# Blocking PDGF-CC signaling ameliorates multiple sclerosis-like neuroinflammation by inhibiting disruption of the blood–brain barrier

**DOI:** 10.1038/s41598-020-79598-z

**Published:** 2020-12-24

**Authors:** Manuel Zeitelhofer, Milena Z. Adzemovic, Christine Moessinger, Christina Stefanitsch, Carina Strell, Lars Muhl, Lou Brundin, Linda Fredriksson, Tomas Olsson, Ulf Eriksson, Ingrid Nilsson

**Affiliations:** 1 Division of Vascular Biology, Department of Medical Biochemistry and Biophysics, Karolinska Institutet, 171 77 Stockholm, Sweden; 2 Neuroimmunology Unit, Department of Clinical Neuroscience, Center for Molecular Medicine, Karolinska University Hospital, 171 76 Stockholm, Sweden; 3 Department of Immunology, Genetics and Pathology, Uppsala University, 75185 Uppsala, Sweden

**Keywords:** Blood-brain barrier, Neuroimmunology

## Abstract

Disruption of blood–brain barrier (BBB) integrity is a feature of various neurological disorders. Here we found that the BBB is differently affected during the preclinical, progression and remission phase of experimental autoimmune encephalomyelitis (EAE), an animal model of multiple sclerosis (MS). We have identified an upregulation of pro-inflammatory and pro-angiogenic factors in the BBB transcriptome and down-regulation of endothelial tight junction members coinciding with elevated BBB leakage specifically during the progression phase. These changes were antagonized by blocking PDGFRα signaling with the small tyrosine kinase inhibitor imatinib. Moreover, targeting the PDGFRα ligand PDGF-CC using a neutralizing antibody, facilitated recovery of BBB integrity and improvement of EAE symptoms. Intracerebroventricular injection of PDGF-CC induced upregulation, whereas blocking PDGF-CC during EAE led to downregulation of *Tnfa* and *Il1a* at the BBB. Our findings suggest that blocking PDGF-CC counteracts fundamental aspects of endothelial cell activation and disruption of the BBB by decreasing *Tnfa* and *Il1a* expression. We also demonstrate that both PDGF-CC and its receptor PDGFRα were upregulated in MS lesions indicating that blocking PDGF-CC may be considered a novel treatment for MS.

## Introduction

The blood–brain barrier (BBB) with counterparts found in the spinal cord (blood-spinal cord barrier) and retina (blood-retinal barrier), represents a dynamic interface between the central nervous system (CNS) and the peripheral immune system. The anatomical basis of this barrier are tight junctions between endothelial cells (ECs). ECs are supported by pericytes and both vascular cell types are surrounded by the basement membrane comprised of extracellular matrix components (ECM). Astrocyte endfeet ensheath blood vessels as well as neuronal synapses. The association of these neuronal cells, vascular cells, and ECM components is termed the neurovascular unit, which is highly responsive to both physiological and pathological changes while tending to maintain regular function and integrity. In vivo demonstration of gadolinium uptake^11,32^ and post-mortem evidence of vascular leakage and tight junction (TJ) degradation^6^ implicate that breach of the BBB is an essential event in a number of neuropathologies, including multiple sclerosis (MS). MS, an inflammatory demyelinating disease characterized by multifocal CNS lesions, represents the leading cause of non‐traumatic disability among young adults in Europe and the USA^19^. Currently available immunomodulatory therapies have a modest effect on disease progression and may even be associated with a risk for developing an opportunistic infection such as progressive multifocal leukoencephalopathy (PML)^26^. Hence, there is a need for developing novel treatments for MS, preferably acting on molecular targets expressed exclusively at the BBB and not on immune cells, which should decrease the risk for adverse complications.

It is believed that in MS, activated T cells, macrophages and brain resident microglia produce cytokines and chemokines that mediate upregulation of respective receptors and adhesion molecules at the BBB^28,54^. For example, TNF-α and IFN-γ promote both adhesion and migration of leucocytes across the BBB by influencing the expression of various chemokines such as CCL2 to CCL5^28^. This leads to endothelial cell activation and subsequent disruption of BBB integrity, resulting in influx of immune cells into the CNS^21,53^. In addition, disturbed balance between pro- and anti-angiogenic chemokines contributes to formation of dysfunctional blood vessels^17^.

Recent studies have shown that the CNS displays a common injury response mechanism involving activation of platelet-derived growth factor receptor α (PDGFRα) signaling, leading to BBB dysfunction and increased vascular permeability^36^. The formal identity of perivascular PDGFRα expressing cells presumed responsible for mediating BBB opening and onset of vascular leakage is still under investigation but suggested to be a distinct arteriolar population of GFAP-positive astrocytes^56,57^ or potentially fibroblast-like cells^61^. PDGF-CC, a ligand of PDGFRα, is a member of the PDGF family consisting of a growth factor domain (GFD) and a N-terminal CUB domain^40^. The latter domain has to be proteolytically removed by tissue plasminogen activator (tPA)^22,23^ in order to release the GFD dimer and subsequently allow the PDGF-CC ligand to activate its receptor^23^. Inhibition of PDGFRα signaling with the small tyrosine kinase inhibitor imatinib has been shown to reduce BBB dysfunction, as demonstrated in animal models of neuroinflammatory and neurodegenerative diseases such as MS^2^ and amyotrophic lateral sclerosis (ALS)^37^, respectively, but also in acute conditions such as stroke^57^, spinal cord injury (SCI)^1^, traumatic brain injury (TBI)^58^ and seizures^25^. Moreover, a recently performed phase II randomized trial in patients with acute ischemic stroke treated with intravenous thrombolysis showed that imatinib significantly improves neurological outcome in comparison to patients treated with tPA only^62^.

The present study is based on yet unreported systematic profiling of transcriptional and phenotypic changes at the BBB during myelin oligodendrocyte glycoprotein (MOG)- induced experimental autoimmune encephalomyelitis (EAE), an animal model of MS. Along with many shared features of the human pathology^55^, this model allows investigation of distinguishable disease phases representing preclinical, progression as well as remission properties^15^. Accordingly, we were able to analyse the disease phase-specific impact on the BBB transcriptome and phenotype and found that blocking PDGFRα leads to downregulation of transcripts important for endothelial cell activation and immune cell transmigration, correlating with restored BBB integrity specifically during disease progression. In order to further investigate the therapeutic potential of PDGFRα modulation in autoimmune neuroinflammation, we utilized a neutralizing PDGF-CC antibody^38^ in a transgenic mouse line expressing PDGF-CC with a humanized growth factor domain (PDGF-CC^hum^)^66^. We could observe that inhibiting PDGFRα signaling by blocking its ligand PDGF-CC indeed preserves BBB integrity, reduces immune cell infiltration and consequently ameliorates EAE. Our data strongly indicate the possibility for establishing a novel treatment specifically targeting disruption of BBB integrity during disease progression.

## Methods

### Ethical statement

All experiments in this study were approved and performed in accordance with the guidelines from the Swedish National Board for Laboratory Animals and the European Union Directive (2010/63/EU) under ethical permits approved by the North Stockholm Animal Ethics Committee.

### Mice

Animals were housed in the animal facility at the Karolinska Institute (Stockholm, Sweden) or at the animal facility at Karolinska Hospital (Stockholm, Sweden) in a climate-controlled environment in polystyrene cages containing aspen wood shavings with free access to standard rodent chow and water with regulated 12-h light/dark cycles. The C57BL/6NTac-Pdgfc^tm3633(K242T, K246R, R299S, K318R, N342S, A343T)Arte^ mice were originally generated by Taconic (Cologne, Germany) and are referred to as PDGF-CC^hum^ throughout the manuscript. PDGF-CC^hum^ mice have been described elsewhere^66^. In short, to exchange the mouse with the human *PDGFC,* six mutations were introduced into the mouse *Pdgfc* sequence. The generation of *Pdgfc*–deficient mice has been described previously^18^. C57BL/6N *Pdgfc*^+*/−*^ and littermate wildtype controls were used in experiments.

### EAE induction and clinical scoring

The N- terminal amino acids 1–125 of myelin oligodendrocyte glycoprotein (MOG) were expressed in *Escherichia coli* and purified to homogeneity by chelate chromatography^7^. The purified protein, dissolved in 6 M urea, was dialyzed against PBS to obtain a physiological preparation that was stored at − 80 °C. Mice at the age of 10 weeks were anaesthetized with isoflurane (Forene, Abbott Laboratories, USA) and injected subcutaneously in the tail base in order to induce EAE with a 100 µl inoculum containing 50 µg MOG in PBS, emulsified 1:1 with complete Freund’s adjuvant (Sigma-Aldrich, USA). Mice were intraperitoneally (i.p.) injected twice with 100 ng pertussis toxin (Sigma-Aldrich, USA), once on the day of immunization and once 2 days post-immunization (p.i.).

Mice were monitored daily for clinical signs of EAE, from day 7 p.i. until they were sacrificed as follows: score 0 = no clinical signs of EAE; 1 = tail paralysis; 2 = hind leg paraparesis or hemiparesis; 3 = hind leg paralysis or hemiparalysis and 4 = tetraplegy or moribund. Statistics were calculated using Kruskal–Wallis test with Dunn's correction for multiple testing (*P < 0.05, **P < 0.01 and ***P < 0.001).

### Antibody and imatinib treatment

To neutralize PDGF-CC in vivo during EAE, PDGF-CC^hum^ mice were injected i.p. twice a week, starting 2 days p.i., with 15 mg/kg/mouse anti-human PDGF-CC monoclonal antibody (mAb) 6B3 or BM4 (IgG mAb control). mAb 6B3 has been described previously^38^. For imatinib treatment, C57BL/6N wildtype mice were oral gavage fed with steel gavage needles with a daily dose of 250 mg/kg/mouse imatinib or phosphate buffered saline (PBS) as control delivered as a morning (1/3) and evening (2/3) dose 8 h later, starting 2 days p.i. Imatinib tablets (Novartis, Switzerland) were crushed into a fine powder, solubilized in sterile PBS, vortexed and incubated at 37 °C (water bath) for 5 min. Insoluble components were spun down in a table microcentrifuge (13,000 rpm) for 2 min. The supernatant was used for oral gavage immediately.

### Tracer injection, immunofluorescence (IF) and histopathological analysis

2.5 mg tetramethylrhodamine-conjugated 70 kDa dextran (dextran-TMR, Invitrogen) dissolved in PBS (0.1 ml) was intravenously infused at the preclinical, progression and remission phase, respectively. The tracer was allowed to circulate for 2 h and thereafter the animals were thoroughly perfused with Hanks’ balanced salt solution (HBSS) followed by 4% paraformaldehyde (PFA). Spinal cords were immediately removed and post-fixed for 4 h in PFA and thereafter cryo-protected in 20% sucrose at 4 °C over night (o/n). The spinal cords were then imaged using a Zeiss Lumar V12 stereo microscope and an Axiocam MRm digital camera (Carl Zeiss microimaging GmbH, Jena, Germany) was used to capture the dorsal view of the entire spinal cords. Epi-fluorescence of the entire dorsal surface was then measured using Image J64 (National Institutes of Health, Bethesda, MD, USA).

To obtain sections from different segments, spinal cords were first cut in 7 mm long pieces, rostral to caudal. Cryosections of 12 μm thickness from the different spinal cord segments were obtained and immunofluorescence was performed as follows: Sections were air-dried for 30 min before permeabilization in PBS/0.2% Triton X-100 for 10 min. Staining against rabbit anti-human ADAMTS9, rabbit anti-mouse CXCL10 (Bioss), monoclonal anti-mouse occludin, monoclonal anti-mouse ZO-1, rabbit anti-mouse claudin-5 (all three from Zymed) and rabbit anti-mouse p65 NFκB (RnD Systems), required antigen retrieval with citrate buffer pH 6.0 (Dako) by warming for 20 min in a steamer device (Braun, Germany). Staining against goat anti-mouse CD31 (RnD systems), rabbit anti-mouse collagen IV (COLLIV, Biorad), goat anti-mouse aquaporin 4 (AQP4, Santa Cruz), rabbit anti-mouse ETS related gene (ERG1, Abcam), rabbit anti-mouse Ki67 (Abcam), goat anti-mouse podocalyxin (PODO, RnD Systems), rabbit anti-mouse CD3 (Abcam), rat anti-mouse CD45 (BD Pharmingen), rat anti-mouse CD68 (Biorad) and rabbit anti-mouse Iba-1 (Wako) did not require antigen retrieval. Blocking was performed in PBS/10%FBS (blocking solution) and primary antibodies diluted in blocking solution were applied o/n at 4 °C. The antibody signal was visualized using Alexa-Fluor conjugated secondary antibodies (Invitrogen). DAPI (4′,6-Diamidino-2-Phenylindole, Dihydrochloride, 0.2 μg/ml) was included in the last PBS wash to visualize the nuclei.

For immunohistochemical stainings (occludin / CD31 co-staining in mouse and PDGF-CC staining in human samples, respectively), paraffin-embedded sections of the spinal cord or brain were treated as previously described^10^. After deparaffinization in x-tra-solv, sections were transferred to 99.5% ethanol. Endogenous peroxidase was blocked by incubation in methanol with 0.02% H_2_O_2_ for 30 min at room temperature (RT) and rehydration to distilled water followed via a 99.5%, 90%, 70%, and 50% ethanol series. Staining against rabbit anti-mouse occludin (Novus Biologicals) and mouse anti-human PDGF-CC mAb 6B3 (provided by Paracrine Therapeutics AB, Sweden) required retrieval with high pH (Envision Flex target retrieval solution, Dako) by warming for 1 h in a steaming device (Braun, Germany). Sections were incubated in 10% FBS in PBS 30 min prior to incubation with primary antibody on 4 °C, o/n. After washing in PBS, sections were incubated with Alexa-Fluor conjugated secondary antibody. DAPI was included in the last PBS wash to visualize the nuclei. All images were acquired with a Zeiss LSM700 confocal microscope and the ZEN 2009 software (Carl Zeiss Microimaging GmbH, Jena, Germany). Representative images shown are 2D renderings of 12 µm thick z-stacks from the lumbar spinal cord region. For all quantifications of antibody immunoreactivity done in this study, images were acquired using the same settings (within each experiment). For quantification of intensity the number of pixels above a set threshold was determined and the quantifications were performed using ImageJ64. For occludin, claudin-5, ZO-1, CD45, CD3, CD68 and Iba-1 evaluation, ten adjacent fields of vision were measured. Whole spinal cord portions were measured to evaluate dextran extravasation. For evaluation of endothelial cell proliferation (Ki67/ERG1 co-staining), all double-positive nuclei were manually counted. Quantifications of immunofluorescence and immunohistochemical stainings, as well as dextran leakage, were calculated using Student´s unpaired *t*-test or one-way ANOVA with Fisher´s LSD (*P < 0.05, **P < 0.01, ***P < 0.001 and ****P < 0.0001). Results are depicted as average ± S.E.M.

PDGFRα staining in human brain samples was performed on the Ventana Discovery Autostainer System (Ventana, Roche, Basel, Switzerland) using PDGFRα antibody (Cell Signaling Technology, Danvers, MA) in Discovery antibody diluent (Ventana, Roche). Extensive antigen retrieval was performed with Discovery Cell Conditioning buffer 1 (Ventana, Roche). Antibodies were incubated for 1 h at room temperature. Detection was performed using the OmniMap DAB anti-rabbit kit (Ventana, Roche) with 30 min incubation of the secondary antibody at room temperature. Representative images shown are from inflammatory demyelinating/remyelinating lesions and normal appearing parenchymal white matter in MS brain parenchyma.

Hematoxylin&eosin (HE) and Luxol Fast Blue (Kluever) were stained according to standard protocol to assess tissue inflammation and demyelination, respectively^2^. HE and Kluever images were acquired with a Zeiss Axio Observer Z1 inverted microscope and the ZEN 2009 software (Carl Zeiss Microimaging GmbH, Jena, Germany). The inflammatory index (I.I.) and demyelination score (DM) were determined from the number and size of demyelinated lesions of each animal on an average of ten complete spinal cord cross-sections as previously described^55^.

### Isolation of vascular fragments

C57BL/6N mice treated with imatinib or PBS were sacrificed during the preclinical, progression or remission phase. Non-immunized C57BL/6N littermate mice were used as naïve controls. PDGF-CC^hum^ mice i.p. injected with anti-PDGF-CC (6B3) or BM4 (IgG control) antibodies were sacrificed during the progression phase. The mice were anesthetized with isoflurane and thereafter perfused with HBSS. Spinal cords were subsequently rapidly dissected out and placed into ice-cold HBSS. The rest of the protocol was performed as described except for the antibody and magnetic beads for pulling out the endothelial vessel fragments^9^. We used biotin rat anti-mouse CD31 (BD Biosciences) together with magnetic beads (Dynabeads biotin binder (Invitrogen Dynal AS, Norway). The purity of vascular fragments was analyzed by real-time qPCR. Markers for endothelial cells (*Pecam1, Cldn5*), pericytes (*Pdgfrb*), astrocyte endfeet (*Aqp4*), neurons (*Dlg4*), microglia (*Iba1*) and immune cells (*Itgam, Cd4 and Lat)* were used to assess the percentage of the distinct cell populations in the vascular fragments. The mean expression level of *Cldn5* was set to 10. The analysis revealed high enrichment of endothelial cells (5.8–7.5 fold for *Pecam1* and tenfold for *Cldn5*), followed by pericytes (0.7–1-fold) and astrocyte endfeet (0.1–0.78 fold). The vascular fragments were depleted of neurons (0.002–0.005 fold), microglia (0.003–0.11 fold) and showed neglectable contamination from immune cells (0.07–0.2 fold for *Cd4*, 0–0.08 fold for *Lat* and 0.07–0.18 fold for *Itgam*) that was similar for EAE and naive vascular fragments (Supplementary Fig. S1). The values represent the relative percentage of each cell type specific marker in the vascular fragments compared to the wash fractions. These results indicate high purity of the vascular fragments with very strong enrichment for endothelial cells and neglectable contaminations from neurons or immune cells in both EAE induced and naïve non-immunized mice.

Total RNA was extracted from both the wash and the eluate fraction using the RNeasy kit (Qiagen, Hilden, Germany) and the QIAcube (Qiagen) including on column DNA-digestion for fully automated sample preparation^2^. RNA concentration and purity were determined through measurement of A260/A280 ratios with a NanoDrop ND-1000 Spectrophotometer (NanoDrop Technologies, Wilmington, DE, USA). Confirmation of RNA quality was assessed using the Agilent 2100 Bioanalyzer (Agilent Technologies, Santa Clara, CA, USA)^2^. Total RNA was subsequently either used for expression array analysis or cDNA generation for qPCR analysis. cDNA was prepared using the iScript kit (Bio-Rad, Hercules, CA, USA).

### Real-time qPCR analysis

Real-time quantitative PCR was performed using KAPA SYBR FAST qPCR Kit Master Mix (2x) Universal (KAPA Biosystems) in Rotor-Gene Q (Qiagen) Real-Time PCR thermal cycler according to the manufacturers’ instructions. Expression levels were normalized to the expression of *Rpl19*. Primers used in this study are described in Supplementary Table S1. For real-time PCR statistics one-way ANOVA with Fisher´s LSD (*P < 0.05, **P < 0.01, ***P < 0.001 and ****P < 0.0001) was used.

### Microarray procedure and data analysis

The procedure and data analysis were done as in^2^. 250 nanograms of total RNA from each sample were used to generate amplified and biotinylated sense-strand cDNA from the entire expressed genome according to the Ambion WT Expression Kit (P/N 4425209 Rev C 09/2009) and Affymetrix GeneChip WT Terminal Labeling and Hybridization User Manual (P/N 702808 Rev. 6, Affymetrix Inc., Santa Clara, CA). GeneChip ST Arrays (GeneChip Gene 2.0 ST Array) were hybridized for 16 h in a 45 °C incubator, rotated at 60 rpm. According to the GeneChip Expression Wash, Stain and Scan Manual (PN 702731 Rev 3, Affymetrix Inc., Santa Clara, CA) the arrays were then washed and stained using the Fluidics Station 450 and finally scanned using the GeneChip Scanner 3000 7G.

The raw data was normalized in the free software Expression Console provided by Affymetrix (http://www.affymetrix.com) using the robust multi-array average (RMA) method first suggested by Li and Wong in 2001^30^. Subsequent analysis of the gene expression data was carried out in the freely available statistical computing language R (http://www.r-project.org) using packages available from the Bioconductor project (www.bioconductor.org). In order to search for the differentially expressed genes between X and the Y groups an empirical Bayes moderated t-test was then applied^51^, using the ‘limma’ package. To address the problem with multiple testing, the p-values were adjusted using the method of Benjamini and Hochberg.

Molecules from the data set that met the log2 fold change >1 cut off (i.e. at least twofold up- or downregulated where a positive log2 fold change indicate an upregulated gene) and an adjusted *P* value < than 0.05 were uploaded to the Ingenuity pathways analysis platform (Ingenuity Systems, CA, USA, www.ingenuity.com). The molecules in this data set were associated with a canonical pathway in Ingenuity’s knowledge base. The significance of the association between the data set and the canonical pathway was measured in 2 ways: (1) A ratio of the number of molecules from the data set that map to the pathway divided by the total number of molecules that map to the canonical pathway is displayed. (2) Fisher’s exact test was used to calculate a *P*-value determining the probability that the association between the genes in the data set and canonical pathway is by chance alone.

The full EAE and healthy naïve microarray data sets of vascular fragments presented in this publication have been deposited to the GEO expression omnibus database with the accession number GSE150562 and GSE157604, respectively.

### Intracerebroventricular (ICV) injections

#### For isolation of vascular fragments

Wildtype female C57BL/6N mice were anesthetized with isoflurane, placed on a stereotactic frame, and thereafter injected with either 4 µl of active PDGF-CC core protein (3 µM) or PBS into the left lateral ventricle (bregma-0.6, mediolateral-1.2 and dorsoventral-2) using an automatic injection pump over 7 min (Robot Stereotaxic, Neurostar, Tubingen, Germany). The recombinant core domain of PDGF-CC was produced in baculo-virus infected insect Sf9 cells and purified as described^40^. For vascular fragment isolation and subsequent qPCR analysis, mice were perfused with HBSS 4 h after ICV injection and the left hemisphere was immediately processed for vascular fragment isolation and quantitative real-time PCR as described above for spinal cords. Statistics were calculated using Student´s unpaired *t*-test (*P < 0.05, **P < 0.01, ***P < 0.001).

#### For Evans blue (EB) dye extravasation

For BBB permeability analysis active PDGF-CC core protein (3 μM) or PBS as control was ICV injected into the dorsal 3rd ventricle (bregma-0.94, mediolateral 0 and dorsoventral-2.45) in mice pre-treated with either imatinib (or PBS as control) or 6B3 antibody (or BM4 as control) 2-5 h before ICV injection. Directly after ICV injection mice were intravenously injected with 0.1 ml of 4% EB dye (Sigma-Aldrich). 2 h later, animals were perfused with HBSS for 8 min and the brains were removed and photographed with a Samsung s4mini mobile phone (Samsung, South Korea). Each brain was then homogenized in *N*,*N*-dimethylformamide (Sigma-Aldrich) and centrifuged for 45 min at 25,000 rcf (Eppendorf centrifuge, model 5417R). The supernatants were collected and quantitation of EB extravasation was performed as described^65^. EB levels in each brain were determined from the formula: (*A*_620_ nm − ((*A*_500_ nm + *A*_740_ nm) ∕ 2)) ∕ mg wet weight. One-way ANOVA with Fisher´s LSD (*P < 0.05, **P < 0.01, ***P < 0.001 and ****P < 0.0001) was used.

#### For ELISA

For enzyme-linked immunosorbent assay (ELISA) active PDGF-CC core protein (3 μM) or PBS as control was ICV injected into the left lateral ventricle (bregma 0.04, mediolateral -0.88 and dorsoventral -2.82) in wildtype C57BL/6N female mice. Eight hours later, cerebrospinal fluid (CSF) was harvested from cisterna magna with a fine glass microcapillary followed by euthanasia of the mice. The CSF was immediately frozen and kept at − 80 °C.

### ELISA

CSF harvested 8 h after PDGF-CC or PBS ICV injection or at the EAE progression phase was subjected to ELISA according to the manufacturer’s protocol (Quantikine HS mouse TNF-α and IL-1α immunoassays, RnD Systems, Minneapolis, MN). Statistics were calculated using Student´s unpaired *t*-test (*P < 0.05, **P < 0.01, ***P < 0.001).

### Human tissue samples

Immunohistochemical staining of PDGF-CC and PDGFRα was performed on brain samples from 12 MS cases supplied by the Multiple Sclerosis Society Tissue Bank, funded by the Multiple Sclerosis Society of Great Britain and Northern Ireland, registered charity 207,495. All participants gave prospective premortem written informed consent for their brains to be banked and used for research. Besides normal appearing white matter (NAWM), the sample collection comprised different lesion phenotypes (active (AL), chronic active (CAL), chronic (CL) and remyelinated lesions (RL).

### Ethical approval

All applicable international, national, and/or institutional guidelines for the care and use of animals were followed. All animal experiments in this study were approved and performed in accordance with the guidelines from the Swedish National Board for Laboratory Animals and the European Union Directive (2010/63/EU) under ethical permits approved by the North Stockholm Animal Ethics Committee. All experiments involving human material in this study were approved by the Regional Ethics Committee in Stockholm (No. 2012/1417–31/1).

The Multiple Sclerosis Society Tissue Bank at the Imperial College London has been approved as a Research Tissue Bank by the Wales Research Ethics Committee (Ref. No. 08/MRE09/31 + 5). All participants gave prospective premortem written informed consent for their brains to be banked and used for research. All procedures performed involving human participants were in line with the ethical standards as laid down in the 1964 Declaration of Helsinki and its later amendments or comparable ethical standards.

## Results

**Figure 1:**
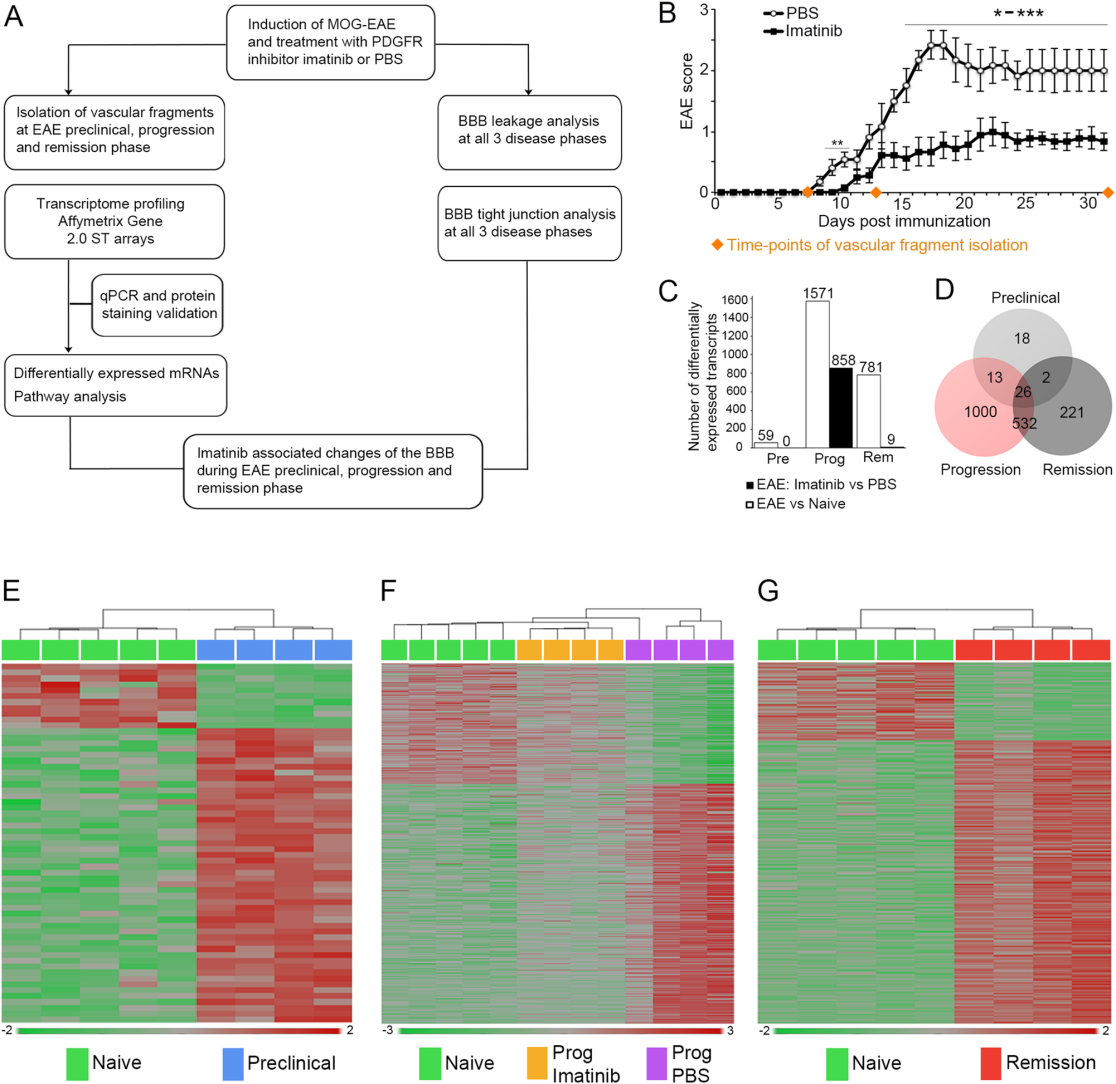
Transcriptome analysis of spinal cord endothelial cell enriched vascular fragments during the preclinical, progression and remission phase of EAE. (**A**) Schematic illustration of study design: C57BL/6N mice were immunized with MOG to induce EAE and treated either with the PDGFRα inhibitor imatinib or PBS from day 2 p.i. until the end of the experiment. At the preclinical, progression and remission phase, spinal cords were harvested and vascular fragments were isolated (n = 4 mice for each disease phase and treatment group, respectively). In addition, vascular fragments from healthy naive (non-immunized) mice were isolated (n = 5). RNA prepared from vascular fragments was hybridized to Affymetrix mouse Gene 2.0 ST arrays and differential gene expression was confirmed with qPCR. Analysis of BBB leakage and tight junctions was done on spinal cord cross-sections. (**B**) EAE induced mice treated with imatinib showed significantly decreased clinical scores compared to PBS treated mice (n = 6 for PBS and n = 9 for imatinib treated mice; 1 representative of 2 independent experiments is shown). (**C**) Representation of number of differentially expressed genes in the BBB transcriptome between naive and EAE induced mice (white bars: 59 at the preclinical phase, 1571 at the progression phase and 781 at the remission phase), and between imatinib and PBS treated EAE mice (black bars: 0 at the preclinical phase, 858 at the progression phase and 9 at the remission phase), respectively. (**D**) Venn diagram showing the distribution of differentially expressed transcripts between naïve and EAE induced mice. 26 transcripts were shared between all three disease phases, 13 between preclinical and progression phases and 532 between progression and remission phases. (**E**–**G**) Heat map diagrams showing differential expression profiles between naïve and EAE induced mice at preclinical (**E**), progression (**F**), and remission (**G**) phases, as well as between imatinib and PBS treated mice during EAE progression (**F**). Adjusted *P* value < 0.05 and a log2 fold change >1 was used as statistical cut off for all analyses. EAE scores are depicted as mean ± SEM. Statistical evaluation for EAE scores was performed using Mann–Whitney (*P < 0.05, **P < 0.01, ***P < 0.001 and ****P < 0.0001). Abbreviations: Pre: preclinical phase, Prog: progression phase, Rem: remission phase, MS: multiple sclerosis.

We have previously shown that imatinib preserves BBB integrity and ameliorates clinical symptoms of EAE in rats^2^. Here we extend our study focusing on the consequences of blocking PDGFRα signaling at the neurovascular unit (NVU) in mouse EAE utilizing imatinib as well as a neutralizing antibody against the PDGFRα ligand PDGF-CC. For this we first performed a genome wide analysis of the BBB transcriptome on endothelial cell enriched vascular fragments isolated from the spinal cords of: i) MOG-immunized, imatinib-treated and ii) MOG-immunized, PBS-treated C57BL/6N mice sacrificed during the preclinical, progression and remission phase of EAE, respectively. As control, we used vascular fragments isolated from naïve (non-immunized) littermates. The purity of the vascular fragments subjected to transcriptome analyses was assessed using cell type specific markers and found to be highly enriched in endothelial cells with minor enrichment of pericytes, while devoid of contaminating non-vascular cells such as neurons, microglia and immune cells (Supplementary Fig. S1). Differential gene expression was analyzed using Affymetrix GeneChip Gene 2.0 ST arrays and validated by qPCR analyses. Functional integrity of the BBB was assessed in vivo using dextran-TMR and by immuno-staining of TJ components at each of the three disease phases, respectively. The experimental design is depicted in Fig. 1A and as previously shown in rats^2^, imatinib was able to ameliorate clinical symptoms of EAE in mice (Fig. 1B). This protective effect was associated with less severe CNS inflammation and demyelination comparing to the PBS-treated group (Supplementary Fig. S2).

### Disease phase-specific transcriptional changes at the BBB are restored by blocking PDGFRα predominantly during disease progression

In line with our hypothesis, endothelial cell enriched vascular fragments isolated from the spinal cord of MOG-immunized, PBS-treated mice displayed marked changes in gene expression at all three phases of EAE compared to the naïve BBB transcriptome, respectively (Fig. 1C–G). Using adjusted *P* value < 0.05 and log twofold change > or < 1 (log twofold change (1)), we detected 59 transcripts that were differentially expressed in the preclinical (Fig. 1E, Supplementary Table S2), 1571 in the progression (Fig. 1F, Supplementary Table S3) and 781 in the remission phase, respectively, (Fig. 1G, Supplementary Table S4) of EAE comparing to the BBB transcriptome of naïve mice (Fig. 1C). The Venn diagram in Fig. 1D shows differentially expressed genes that either overlap or are exclusive for one of the three disease phases. 26 transcripts, mainly related to leucocyte adhesion and endothelial cell activation, were common for all three disease phases (Supplementary Table S5).

Using the same cut-off as above, we detected 0 differentially expressed transcripts in the preclinical, 858 in the progression (Supplementary Table S6) and only 9 in the remission phase of the MOG-immunized, imatinib-treated compared to the MOG-immunized, PBS-treated group (Fig. 1C,G). Hence, treatment with imatinib predominantly affected the BBB transcriptome during the progression phase of EAE. To rule out potential effects of imatinib on the naïve BBB, we compared the transcriptome of the vascular fragments isolated from imatinib- and PBS- treated naïve (non-immunized) mice and could not find any expression changes between the groups (Supplementary Fig. S3).

### Pro-inflammatory and pro-angiogenic mediators upregulated during the progression phase are reduced by blocking PDGFRα

**Figure 2:**
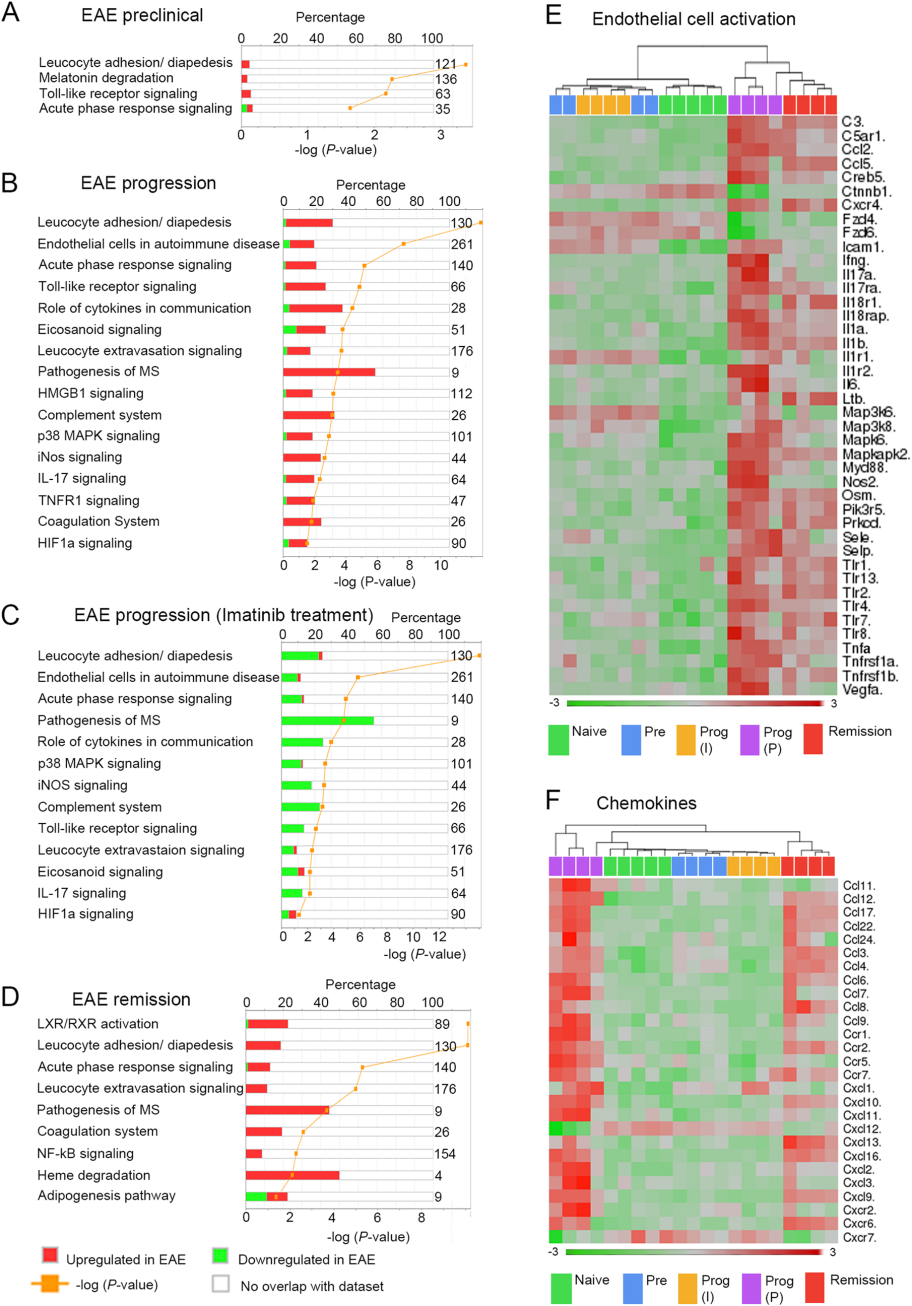
BBB transcriptome pathway analyses across different EAE disease phases and response to imatinib treatment. (**A**–**D**) Representation of selected canonical pathways differentially regulated between naïve and EAE induced mice during the preclinical (**A**), progression (**B**) and remission phase (**D**), or between imatinib- and PBS treated EAE induced mice in progression phase (**C**). The percentage of significantly differentially expressed transcripts of the corresponding pathway is depicted. The numbers on the right represents the number of genes in the respective pathway. For canonical pathway analysis, the significance of the association between the data set and the canonical pathway was measured in 2 ways: (1) A ratio of the number of molecules from the data set that map to the pathway divided by the total number of molecules that map to the canonical pathway is displayed. (2) Fisher’s exact test was used to calculate a *P*-value determining the probability that the association between the genes in the data set and canonical pathway is by chance alone. (**E**–**F**) Heat map diagrams of transcripts implicated in endothelial cell activation (**E**) and of chemokines (**F**) in naïve and EAE induced mice using hierarchical clustering. Abbreviations: Pre: preclinical phase, Prog: progression phase, MS: multiple sclerosis.

In order to identify the most relevant pathways for each of the three phases of EAE, we performed IPA canonical pathway analyses with Ingenuity (Fig. 2A–D). The BBB gene expression profile of the preclinical phase, i.e. prior to manifestation of clinical symptoms, was characterized by upregulation of transcripts important for endothelial cell activation (*Il1b, Il1r1, Tnfa*) and leucocyte adhesion (*Icam1, Cd14*) as well as chemokines *Ccl4* and *Ccl3l3* (Fig. 2A,E and Table 1). In the progression phase, leucocyte adhesion and endothelial cell activation were affected alongside IL-17, Toll-like receptor, iNOS and HIF1α signaling (Fig. 2B,E,F and Table 1 and 2).

**Table 1:** Differentially expressed immune mediators in the BBB transcriptome during the preclinical, progression and remission phase of EAE.

Gene symbol	Entrez gene name	Affymetrix ID	Fold change preclinical	Fold change progression	Fold change progression (I)	Fold change remission
**Proliferation of antibody producing cells and effector T cell proliferation**						
Il2Ra	Interleukin 2 receptor, alpha	17366992		1.63		1.64
Il2Rb	Interleukin 2 receptor, beta	17319009		2.35		2.38
Il2Rg	Interleukin 2 receptor, gamma	17543572		1.22		
Il2	Interleukin 2	1704880		1.84		
Il6	Interleukin 6	17435725		3.20	− 2.73	
**Increased chemokine production**						
Il17A	Interleukin 17A	17211369		2.59	− 2.74	
Il17Ra	Interleukin 17 receptor A	17462351		1.56	− 1.29	
**Activation of macrophages**						
Ifng	Interferon, gamma	17237589		2.76	− 2.66	
**Th1 differentiation**						
Spp1	Secreted phosphoprotein 1	17439830		3.55	3.73	4,99
Il12Rb2	Interleukin 12 receptor, beta 2	17467323		1.42	1.81	
Ifng	Interferon, gamma	17237589		− 2.76	2.66	
**Differentiation, proliferation and activation of NK cells**						
Il2Ra	Interleukin 2 receptor, alpha	17366992		1.63		1.64
Il2Rb interleukin	2 receptor, beta	17319009		2.35	− 2.87	2.38
Il2Rg	Interleukin 2 receptor, gamma	17543572		1.22		
Il15Ra	Interleukin 15 receptor, alpha	17367004		1.01		
Il12Rb2	Interleukin 12 receptor, beta 2	17467323		1.42	− 1.81	
Il2	Interleukin 2	17404880		1.84		
Tnfa	Tumor necrosis factor apha	17344309	1.65	3.59	− 2.63	
**Leucocyte adhesion and transmigration**						
Itgal	Integrin, alpha L	17483264		2.68	− 2.69	2.07
Itgb2	Integrin, beta 2	17234647		2.62	− 2.05	1.95
Mmp8	Matrix metallopeptidase 8	17514553		4.12	− 3.25	1.28
Mmp14	Matrix metallopeptidase14	17300279		2.79	− 2.46	1.64
Osm	Oncostatin M	17246803		2.94	− 2.81	2.38
Selp	Selectin P	17218845		4.54	− 3.42	2.76
Timp1	TIMP metallopeptidase inhibitor 1	17533713		3.64	− 3.33	1.88
Cd44	CD44 molecule (Indian blood group)	17388733		2.74	− 2.86	
Icam1	Intercellular adhesion molecule 1	17515074	1.40	1.71		
Itga5	Integrin, alpha 5	17322369		1.41	− 1.20	
Il17A	Interleukin 17A	17211369		2.59	− 2.74	
Il17Ra	Interleukin 17 receptor A	17462351		1.56	− 1.29	
Mmp19	Matrix metallopeptidase 19	17238558		2.67	− 2.41	
Sele	Selectin E	17218820		2.86	# − 1.85	1.39
Il4R	Interleukin 4 receptor	17482943		2.35	− 1.43	1.11
**Toll like receptors**						
Cd14	CD14 molecule	17353747	1.00	2.19	− 1.26	1.85
Tlr1	Toll-like receptor 1	17448245		1.30	− 1.48	1.19
Tlr2	Toll-like receptor 2	17406279		1.89	− 1.71	1.89
Tlr4	Toll-like receptor 4	17414836		1.37		
Tlr6	Toll-like receptor 6	17448251		1.02		
Tlr7	Toll-like receptor 7	17546109		1.09		1.39
Tlr8	Toll-like receptor 8	17546101		2.17	− 2.09	1.34
Tlr13	Toll-like receptor 13	17537081		1.26		1.15
**Eicosanoid signaling**						
Alox12	Arachidonate 12-lipoxygenase	17265193		− 1.68	1.13	− 1.19
Alox5Ap	Arachidonate 5-lipoxygenase-activating protein	17444961		1.62	− 1.13	
Fpr2	Formyl peptide receptor 2	17333731		3.62	− 3.15	
Ltb4R	Leukotriene B4 receptor	17300666		1.99	− 2.48	
Ltc4S	Leukotriene C4 synthase	17262316		− 1.52	1.35	
Pla2G7	Phospholipase A2, group VII	17337796		1.75	− 2.08	
Pla2G4A	Phospholipase A2, group IVA	17227828		1.06		2.09
Ptger2	Prostaglandin E receptor 2 (subtype EP2)	17299180		1.34		
Ptger4	Prostaglandin E receptor 4 (subtype EP4)	17315718		1.21	− 1.15	
Ptgis	Prostaglandin I2 (prostacyclin) synthase	17394679		− 1.14		
Ptgs2	Prostaglandin-endoperoxide synthase 2	17218060	2.04	3.25		2.10
Tbxa2R	Thromboxane A2 receptor	17235694		− 1.17		− 1.10
Tbxas1	Thromboxane A synthase 1 (platelet)	17457472		1.10		

Differentially expressed genes between naïve mice and MOG-EAE induced mice during preclinical, progression and remission phase as well as from imatinib compared to PBS treated mice during the progression phase, respectively. An adjusted *P* value lower than 0.05 and a log2 fold change >1 was used as statistical cut off except for the transcript indicated with # (adjusted *P* value 0.06). Fold change progression (I) refers to the comparison of imatinib versus PBS treated mice during the progression phase.

**Table 2:** Differentially expressed vascular mediators in the BBB transcriptome during the preclinical, progression and remission phase of EAE.

Gene symbol	Entrez gene name	Affymetrix ID	Fold change preclinical	Fold change progression	Fold change progression (I)	Fold change remission
**Angiogenesis**						
Thbs1	Thrombospondin 1	17374488		3.24	− 3.74	
Hpse	Heparanase	17548411		2.99	− 2.94	4.05
Adamts9	ADAM metallopeptidase with thrombospondin type 1 motif	17469136		2.65	− 2.28	
Ptafr	Platelet-activating factor receptor	17419437		1.87		1.76
Angpt2	Angiopoietin 2	17507799		1.56		
Plaur	Plasminogen activator, urokinase receptor	17474974		2.92	− 2.16	1.43
Plau	Plasminogen activator, urokinase	17297537		1.52		2.15
Ace	Angiotensin I converting enzyme	17257444		1.08		
Pdgfb	Platelet-derived growth factor beta polypeptide	17319380		− 1.24	1.08	
Sdpr	Serum deprivation response	17212719		− 1.56	1.91	
Aplnr	Apelin receptor	17372725	− 1.81	− 1.64		
Apln	Apelin	17541378		− 1.70		− 1.75
Saa3	Serum amyloid A 3	17491193		5.97	− 5.82	4.00
Fzd4	Frizzled class receptor 4	17480036		− 1.03	1.24	
Fzd6	Frizzled class receptor 6	17311179		− 1.44	1.66	
Fzd8	Frizzled class receptor 8	17348276			1.19	
Tfrc	Transferrin receptor	17324835		− 2.07	1.41	
Tie1	Tyrosine kinase with IG-like and EGF-like domains 1	17429206		− 1.58	1.41	
**iNos signaling**						
Cd14	CD14 molecule	17353747	1.00	2.19	− 1.26	1.85
Ifng	Interferon, gamma	17237589		2.76	− 2.66	
Ikbke	Inhibitor of kappa light polypeptide gene enhancer in B-cells	17226771		2.16	− 1.90	1.43
Irf1	Interferon regulatory factor 1	17249593		1.50	− 1.23	
Lbp	Lipopolysaccharide binding protein	17378827		1.64		
Nos2	Nitric oxide synthase 2, inducible	17253707		3.04	# − 2.73	
Stat1	Signal transducer and activator of transcription 1, 91 kDa	17212750		1.73	− 1.14	
Tlr4	Toll-like receptor 4	17414836		1.37	* − 0.81	
**HIF1**a **signaling**						
Egln3	Egl nine homolog 3 (C, elegans)	17281084		2.79	− 2.94	
Hif1A	Hypoxia inducible factor 1, alpha subunit	17276328		1.21	* − 0.71	
Mmp8	Matrix metallopeptidase 8 (neutrophil collagenase)	17514553		4.12	− 3.25	
Mmp14	Matrix metallopeptidase 14 (membrane-inserted)	17300279		2.79	− 2.46	1.64
Mmp19	Matrix metallopeptidase 19	17238558		2.67	− 2.41	
Nos2	Nitric oxide synthase 2, inducible	17253707		3.04	− 2.73	
Tceb1	Transcription elongation factor B (SIII), polypeptide 1	17221497		1.33		
Vegfa	Vascular endothelial growth factor A	17345293		2.36	− 2.27	
Vegfc	Vascular endothelial growth factor C	17501160		− 1.75	1.75	
**Interleukines with important role in angiogenesis**						
Il1A	Interleukin 1, alpha	17391554		2.21	− 1.90	1.22
Il1B	Interleukin 1b	17391565	1.92	5.31	− 4.31	4.25
Il6	Interleukin 6	17435725		3.20	− 2.73	
Ifng	Interferon gamma	17237589		2.76	− 2.66	
Spp1	Secreted phosphoprotein 1	17439830		3.55	− 3.73	4.99
Tgfb2	Transforming growth factor beta	17230830		− 2.32	1.59	− 1.23
**Coagulation**						
F3	Coagulation factor III	17402181		1.30		
F7	Coagulation factor VII	17499212		1.88		
F10	Coagulation factor X	17499224		4.71	− 4.70	
F13a1	Coagulation factor XIII, A1 polypeptide	17291881		2.63	− 2.33	
**Angiogenic chemokines and receptors**						
Ccl2	Chemokine (C–C motif) ligand 2	17254041		5.62	− 4.23	2.87
Ccr2	Chemokine (C–C motif) receptor 2	17523650		3.53	− 3.73	2.80
Ccr1	Chemokine (C–C motif) receptor 1	17532569		3.16	− 2.92	
Cxcr4	Chemokine (C-X-C motif) receptor 4	17226593		1.76	− 2.31	2.20
Ccr5	Chemokine (C–C motif) receptor 5	17523659		1.06	− 1.28	
Cxcl2	Chemokine (C-X-C motif) ligand 2	17438987		1.76		
Cxcl3	Chemokine (C-X-C motif) ligand 3	17438995		3.05		
Cxcl12	Chemokine (C-X-C motif) ligand 12	17462149		− 1.98		
Cxcr2	Chemokine (C-X-C motif) receptor 2	17214142		2.75	− 2.22	
Ccl11	Chemokine (C–C motif) ligand 11	17254053		1.61	− 2.04	
**Angiostatic chemokines and receptors**						
Cxcl9	Chemokine (C-X-C motif) ligand 9	17449710		5.29		
Cxcl10	Chemokine (C-X-C motif) ligand 10	17449718		5.21	− 4.85	3.30
Cxcl11	Chemokine (C-X-C motif) ligand 11	17449725		2.25		

Differentially expressed genes between naïve mice and MOG-EAE induced mice during preclinical, progression and remission phase as well as from imatinib compared to PBS treated mice during the progression phase, respectively. An adjusted *P* value lower than 0.05 and a log2 fold change >1 was used as statistical cut off except for the transcripts indicated with # (adjusted *P* value 0.07) and * (log twofold change 0.81). Fold change progression (I) refers to the comparison of imatinib versus PBS treated mice during the progression phase.

The remission phase was predominantly characterized by activation of the liver X receptor/retinoid X receptor (LXR/RXR) (Fig. 2D and Supplementary Fig. S4) and leucocyte adhesion/diapedesis (Fig. 2D). Thus, cholesterol transporters *Abca1* and *Abcg1,* LDL scavenger receptors *Cd36* and *Msr1,* the triacylglycerol (TAG) hydrolyzing lipase *Lpl* as well as its co-activator *Apoc2,* and *Fabp5*, involved in fatty acid uptake, were specifically upregulated during the remission phase (Supplementary Fig. S4)*.* Further, we observed upregulation of molecules involved in sphingolipid and phosphatidylinositol metabolism such as *Sgpl1*, *Cers6*, *Igf1* and *Inpp5d* (Supplementary Table S4). Notably, transcripts important for endothelial cell activation, immune cell recruitment and angiogenesis were less affected at disease remission compared to the progression phase (Fig. 2E,F, Table 1 and 2).

**Figure 3:**
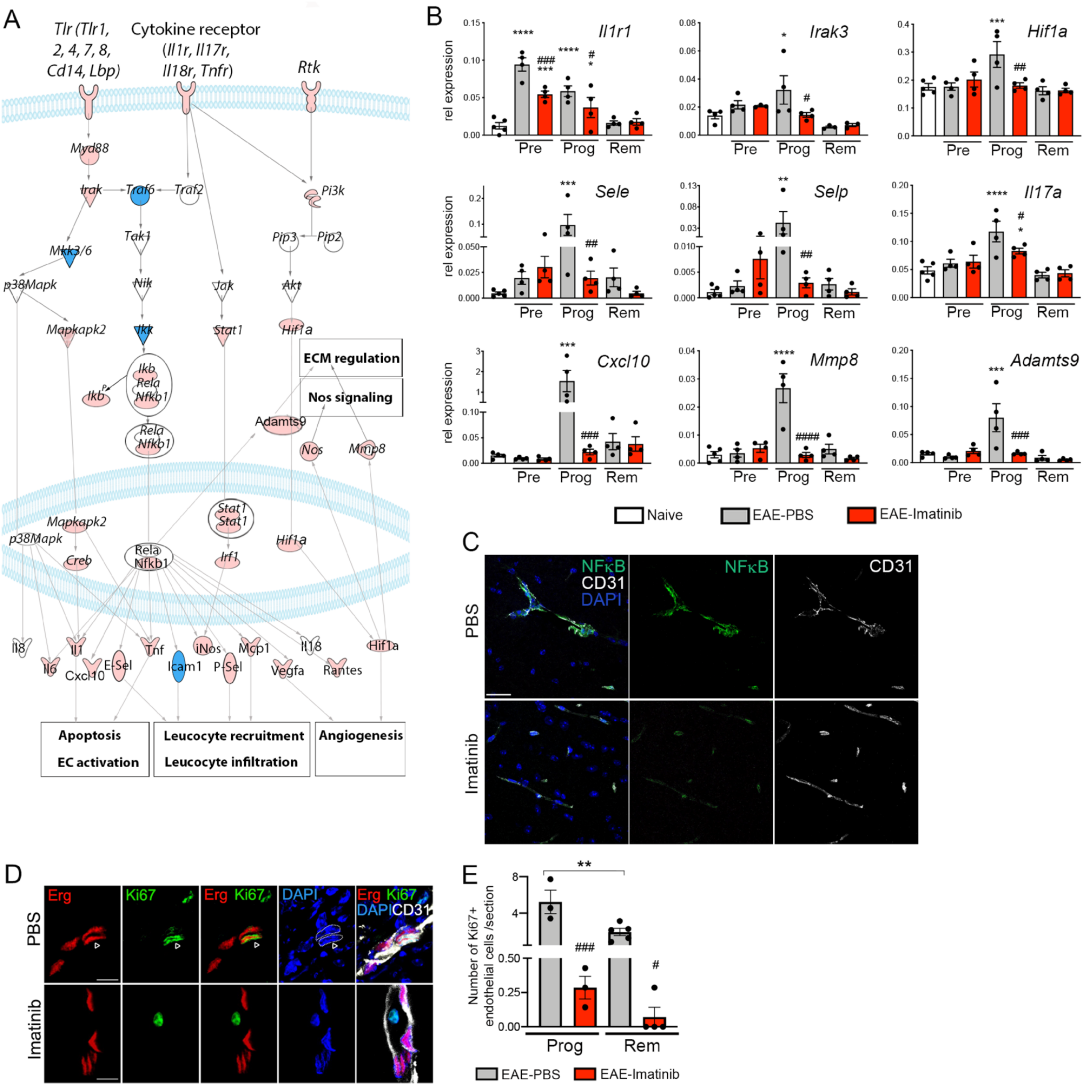
Blocking PDGFRα signaling down-regulates BBB transcripts important for endothelial cell activation and leucocyte recruitment. C57BL/6N mice were immunized with MOG to induce EAE, treated either with imatinib or PBS from day 2 p.i. until the end of the experiment and vascular fragments were isolated from the spinal cord at the preclinical, progression and remission phase. (**A**) Multi-pathway scheme generated by Ingenuity: During the progression phase, genes important for endothelial cell activation, leucocyte recruitment and infiltration, angiogenesis and extracellular matrix regulation are up-regulated via *Mapk* or *NfkB* downstream signaling activated by cytokine and toll-like receptors at the BBB. Molecules in pink indicate that these molecules are upregulated during the EAE progression phase and restored by imatinib treatment. Molecules in blue indicate that these molecules are upregulated during the EAE progression phase but not restored by imatinib treatment, whereas molecules in white are not regulated. (**B**) Quantitative real-time PCR analyses of vascular fragment cDNA from naïve and imatinib or PBS treated EAE induced mice collected at the preclinical, progression and remission phase (n = 4 mice for each treatment group for preclinical, progression and remission phase, n = 5 mice for naive mice). During the progression phase, imatinib significantly down-regulated all transcripts shown. (**C**) Representative images of NfκB p65 subunit expression (green) in spinal cord cross-sections showing expression in endothelial cells and infiltrating immune cells in PBS-treated EAE induced mice in the progression phase, indicative of an inflammatory response at the BBB. Imatinib-treated EAE induced mice exhibit less NfκB p65 immunoreactivity. Anti-CD31 co-staining in white. Scale bar, 20 μm. (**D**) Representative images of the nuclear proliferation marker Ki67 (green) co-stained with the endothelial cell transcription factor ERG (red) and CD31 (white) in spinal cord cross-sections show proliferating angiogenic endothelial cells during disease progression (arrowhead). (**E**) Quantification shows significantly less proliferating endothelial cells during disease remission, compared to progression phase, in the PBS-treated mice. Imatinib-treated mice show less endothelial proliferation during both phases. n = 3 and 4 mice for each treatment group of progression and remission phase, respectively. Results are depicted as mean ± SEM. Statistics were calculated using one-way ANOVA with Fisher´s LSD test and *P* values < 0.05 were considered significant (*^,#^*P* < 0.05; **^,##^*P* < 0.01; ***^,###^*P* < 0.001 and ****^,####^*P* < 0.0001). In B, * significance between naive and EAE PBS treated mice; # significance between EAE PBS and EAE imatinib treated mice. Abbreviations: Pre: preclinical phase, Prog: progression phase, Rem: remission phase.

Blocking PDGFRα signaling with imatinib resulted in downregulation of the following pathways during disease progression (Fig. 2C): Chemokines and chemokine receptors necessary for both angiogenesis and recruitment of immune cells such as *Ccl2*, *Ccl5*, *Ccl7*, *Cxcl2*, *Cxcl10*, *Ccr2* or *Cxcr4* (Fig. 2F, Table 1); transcripts involved in endothelial cell activation such as *Tnfa, Il1a*, *Il1b, Ifng, Vegfa, Il6* and *Il17* (Fig. 2E, Table 1); transcripts important for leucocyte adhesion and transmigration such as *Icam1*, *Sele, Selp*, *Mmp8, Mmp14* and *Mmp19* (Table 1)*;* various toll-like and cytokine receptors such as *Tlr1, Tlr2, Cd14, Il17ra* and *Il1r1* (Table 1); transcription factors such as *Creb5, Mapkapk2* or *Irf1; * molecules involved in the HIF1α signaling pathway such as *Vegfa, Hif1a* and *Egln3* (Table 2). Thus, the expression of genes important for endothelial cell activation, leucocyte recruitment and infiltration, extracellular matrix regulation as well as angiogenesis is downregulated at the BBB by imatinib treatment during the EAE progression phase compared to PBS treated controls (Fig. 3A). As depicted in the multi-pathway scheme all components of the signaling pathways are downregulated: ligands, receptors, intracellular signaling mediators, transcription factors as well as their respective gene products (Fig. 3A). In contrast, monocarboxylate transporters *Slc16a1* and *Slc16a4,* required for pyruvate/lactate and ketone body transport, as well as receptor-mediated transporters of the BBB *Tfrc, Lepr* and *Igf1r* were down-regulated in PBS but not imatinib treated mice (Supplementary Table S7).

The array data were confirmed by qPCR (Fig. 3B) and by immunostaining analysis (Fig. 3C, Supplementary Fig. S5). Finally, reduction of hypoxia and angiogenesis in response to imatinib treatment was confirmed by immunostaining of the proliferation marker Ki67. The analysis showed more Ki67^+^ proliferating endothelial cells in the progression, compared to the remission phase, and the numbers of proliferating endothelial cells were decreased with imatinib-treatment in both phases (Fig. 3D,E).

### Blocking PDGFRα preserves BBB integrity in vivo during disease progression

**Figure 4:**
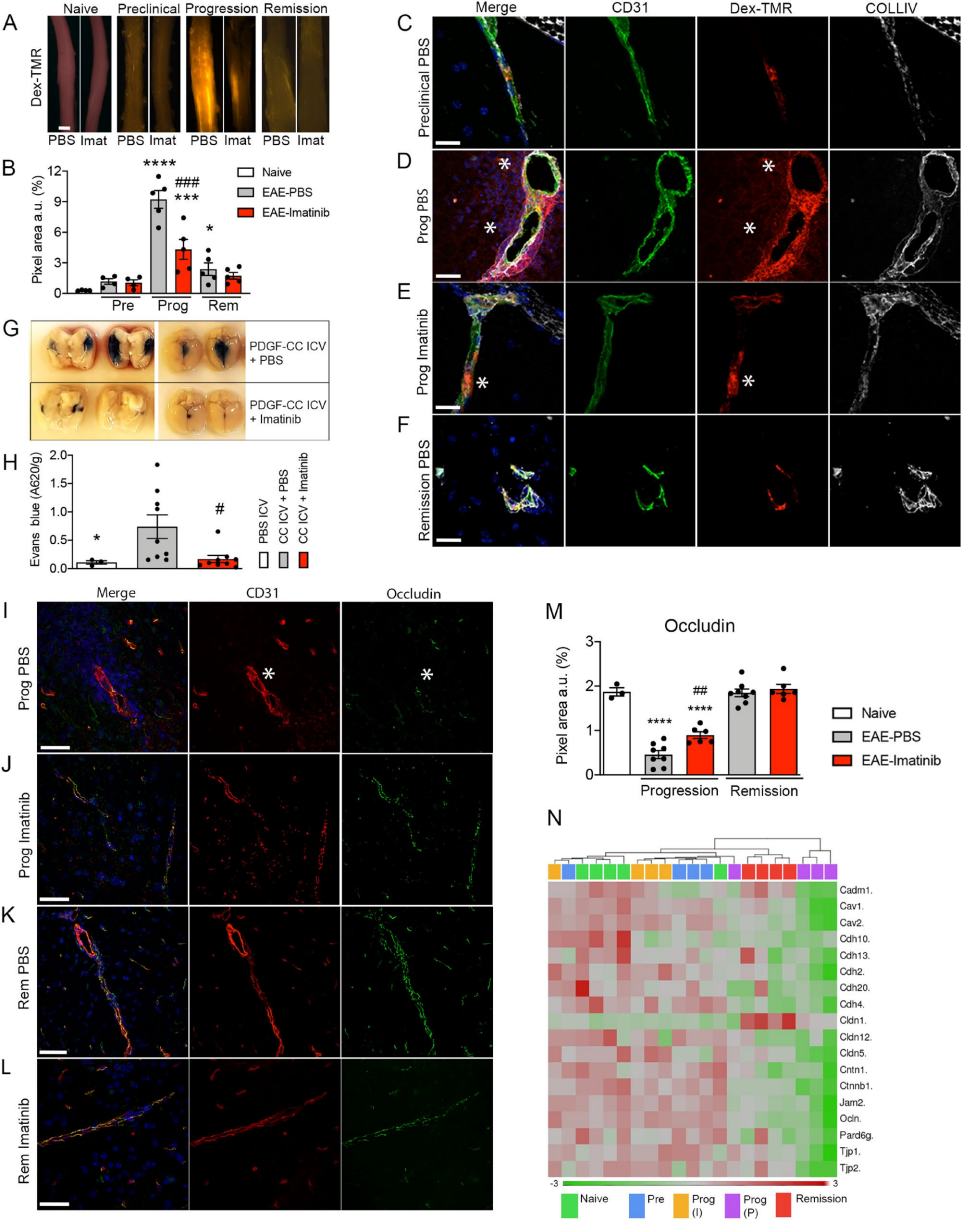
BBB disruption and loss of tight junction integrity in EAE can be rescued by blocking PDGFRα. C57BL/6N mice were immunized with MOG to induce EAE, treated either with imatinib or PBS from day 2 p.i. until the end of the experiment and spinal cords were harvested at the preclinical, progression and remission phase. (**A**, **B**) BBB disruption was evaluated by the amount of extravasation of a circulating 70 kDa dextran tracer (red) into the spinal cord parenchyma. (**A**) Representative images of whole spinal cords. Scale bar: 1 mm. (**A**) The degree of BBB leakage was highest in the progression phase and blocking PDGFRα with imatinib correlated with preservation of BBB integrity in the progression phase. Quantification based on red fluorescent pixel area recorded in spinal cord whole-mounts (n = 4–5 mice per treatment and disease phase). Tracer injected naïve mice served as controls (n = 2 mice). (**C**–**F**) Spinal cord cross-sections from 70 kDa dextran tracer injected mice were co-stained with anti-CD31 (green) and anti-collagen IV (COLLIV, white) to visualize endothelial cells and extracellular matrix (ECM), respectively. Representative images of each disease phase are shown (n =  4 for preclinical phase, n = 5 for progression and remission phase). (**C**) During the preclinical phase the tracer was confined within the luminal portion of the blood vessel wall of the BBB. (**D**) Disease progression resulted in extravasation of the tracer from the endothelial cells, crossing the ECM and diffusing deep into the spinal cord parenchyma (asterisk). (**E**) Imatinib treatment correlated with reduced extravasation of tracer during disease progression. Only a small portion of the tracer was extravasating from the endothelial cells but could not cross the ECM (asterisk). (**F**) During the remission phase the barrier function of the BBB was restored and the tracer was again confined to the blood vessels. Scale bars C-F: 40 µm. (G) ICV injection of active PDGF-CC core protein into the dorsal 3rd ventricle 2–5 h after imatinib or PBS treatment in naïve C57BL/6N mice. BBB leakage was assessed by the degree of Evans Blue extravasation. Images show representative leaky brain regions (dark blue) in separated brain hemispheres (left panels) and in intact brains (dorsal view, panels to the right). (**H**) Quantification of Evans blue extravasation from the PDGF-CC ICV experiment shown in **G** (n = 9 per group receiving PDGF-CC ICV injections, n = 3 for PBS ICV injection controls) shows that blocking PDGFRα with imatinib significantly preserves BBB integrity. (**I**–**L**) Spinal cord cross-sections were co-stained with anti-occludin (green) and anti-CD31 (red) to visualize tight junctions and endothelial cells, respectively (n = 5 for each disease phase and treatment group as well as naive mice). (**I**) Occludin was almost totally lost during the progression phase in PBS treated mice (white asterisk), however blocking PDGFRα with imatinib correlated with increased occludin expression during the progression phase (**J**). Scale bars I and J: 40 µm. (**K**, **L**) During the remission phase, occludin expression was restored also in PBS-treated EAE mice. Scale bars K 60 µm and L 40 µm. (**M**) Quantification of occludin staining during progression and remission phase and in naive mice. (**N**) Heat map diagram with hierarchical clustering showing down-regulation of various BBB structural transcripts during the different EAE disease phases. Imatinib treatment led to up-regulation of BBB structural components such as *Cldn1*, *Cldn5*, *Ocln*, *Tjp1*, *Tjp2*, *Jam2* and various *Cdhs*. For statistical evaluation of dextran, Evans blue and occludin, one-way ANOVA with Fisher´s LSD (*^,#^*P* < 0.05; **^,##^*P* < 0.01; ***^,###^*P* < 0.001 and ****^,####^*P* < 0.0001).) was used. Results are depicted as average  ± SEM. In B and M, * significance between Naive and EAE PBS treated mice; # significance between EAE PBS and EAE imatinib treated mice. In H, * significance between PBS ICV and PDGF-CC ICV + PBS; # significance between PDGF-CC ICV + PBS and PDGF-CC ICV + imatinib. Abbreviations: Pre: preclinical phase, Prog: progression phase, Rem: remission phase, Imat: imatinib, COLLIV: collagen IV, CC ICV: PDGF-CC intracerebroventricular injection.

To correlate the BBB transcriptome data with particular features of BBB integrity, we analyzed the degree of extravasation of fluorescently labeled 70 kDa dextran into the perivascular tissue at the preclinical, progression and remission phases of EAE (Fig. 4A–F). Visualization (Fig. 4A) and quantification (Fig. 4B) of the spinal cord whole-mount preparations demonstrated that discrete leakiness of the BBB was apparent already during the preclinical phase. During the disease progression phase, the BBB was markedly leaky (Fig. 4D) and this overt leaky phenotype had however almost completely restored in the remission phase (Fig. 4F). Immunostaining with a marker for extracellular matrix (ECM), collagen IV (COLLIV), illustrated substantially compromised BBB integrity exclusively during the progression phase with profound extravasation of the dextran tracer beyond the basal lamina, deep into the CNS parenchyma (Fig. 4D). Disruption of BBB integrity during EAE progression was efficiently ameliorated by imatinib (Fig. 4A,B,E).

We have previously shown that imatinib inhibits clonal T cell expansion and downregulates pro-inflammatory mediators in EAE in rats^2^. To assess the ability of imatinib to restore BBB integrity in the absence of inflammation, we administered active PDGF-CC core protein by intracerebroventricular (ICV) injections 2 h after treating C57BL/6N naïve mice with imatinib or PBS, respectively. Indeed, evaluation of Evans Blue (EB) extravasation revealed BBB protection by imatinib (Fig. 4G,H). Hence, we here demonstrate that imatinib can directly target PDGF-CC/PDGFRα signaling in the NVU, conferring BBB protection independently of modulation of the peripheral immune response.

In order to investigate correlation between the transcriptional changes and the functional status of the BBB, we performed immunofluorescence (IF)- analysis of the tight junction (TJ) components occludin, claudin-5 and ZO-1 in spinal cords of mice injected with 70 kDa dextran. During the progression phase, all TJ markers exhibited a discontinuous and aberrant expression pattern (Fig. 4I–L, Supplementary Fig. S6) and quantification of the staining revealed a significant reduction in TJ marker expression compared to naïve controls (Fig. 4M, Supplementary Fig. S6). In line with this observation, several other structural components of the BBB were transcriptionally downregulated during the disease progression phase, including *Jam2*, *Cav1, Cav2, Cldn5* and *Tjp1* (Fig. 4N). In contrast to the PBS-treated group, occludin, claudin-5 and ZO-1 expression in the MOG-immunized, imatinib-treated mice was preserved during the progression phase as judged by a stronger and more continuous expression pattern (Fig. 4J,M, Supplementary Fig. S6). During disease remission, occludin, claudin-5 and ZO-1 levels were restored and comparable to naïve controls (Fig. 4K,L,M, Supplementary Fig. S6). This was in line with the transcription profile from the PBS-treated group in disease remission that showed recovery of various TJ components in contrast to the profound downregulation in the progression phase (Fig. 4N).

### Blocking PDGF-CC preserves BBB integrity and ameliorates EAE

**Figure 5:**
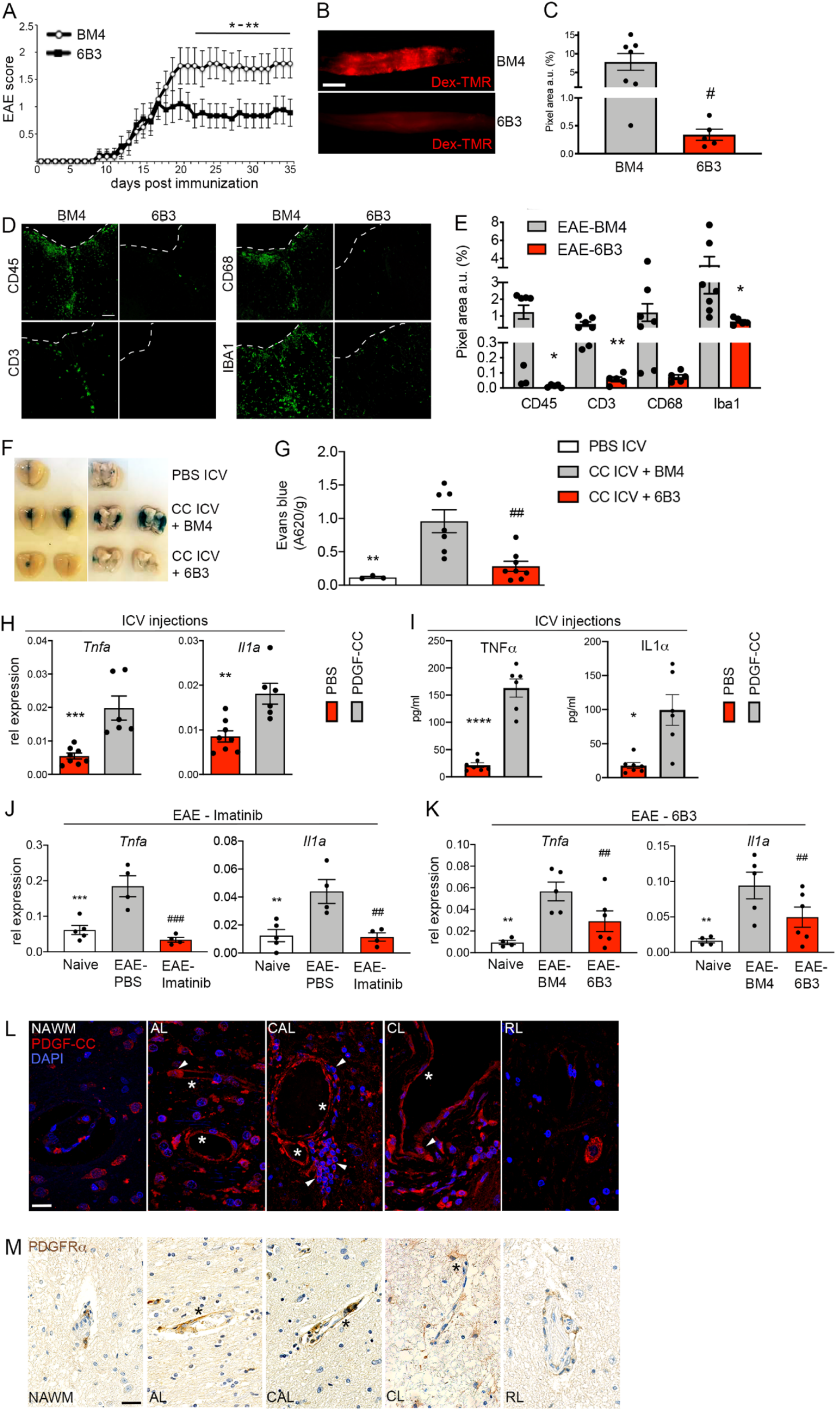
Blocking PDGF-CC down-regulates *Il1a* and *Tnfa* expression at the BBB, correlating with improved BBB integrity and amelioration of EAE. (**A**) PDGF-CC^hum^ mice were treated either with 15 mg/kg anti-PDGF-CC mAb 6B3 or BM4 (IgG control) from day 2 p.i. until the end of the experiment twice per week (n = 9 for 6B3 and n = 10 for BM4 treated mice, respectively, 1 representative of 2 independent experiments shown). 6B3 treated mice developed less severe EAE than BM4 treated controls. (**B**) Representative images showing extravasation of a circulating 70 kDa dextran tracer (red) in whole-mount spinal cord portions at the EAE progression phase. 6B3 treated PDGF-CC^hum^ mice exhibited less extravasation compared to BM4 treated controls. Scale bar: 2 mm. (**C**) Quantification of vascular permeability at the EAE progression phase based on red fluorescent pixel area recorded in spinal cord whole-mounts (n = 5 for 6B3 and n = 7 for BM4 treated mice, respectively). (**D**) Representative images of CD45^+^, CD3^+^, CD68^+^ and IBA1^+^ immune cells in the spinal cord of 6B3 and BM4 treated mice during the progression phase, Scale bar: 50 μm. (**E**) Quantification of immune cell content based on evaluation of IF stainings in D (n = 5 for 6B3 and n = 7 for BM4). (**F**) Active PDGF-CC core protein was stereotactically injected into the dorsal 3rd ventricle of C57BL/6N mice 2–5 h after systemic pre-treatment with 6B3 or BM4 and BBB leakage was assessed by the degree of Evans Blue extravasation (n = 7 for PDGF-CC ICV + BM4 and n = 8 for PDGF-CC ICV + 6B3, n = 3 for PBS ICV controls). Images show representative leaky brain regions (dark blue) in intact brains (dorsal view, left panels) and in separated brain hemispheres (right panels). (**G**) Quantitative evaluation of Evans blue extravasation from the PDGF-CC ICV experiment shown in F. Blocking PDGF-CC with mAb 6B3 could significantly reduce Evans Blue extravasation. (**H**) qPCR analysis of vascular fragment cDNA isolated 4 h after ICV injection into the left lateral ventricle of C57BL/6N mice with active PDGF-CC core protein or PBS (n = 8 for PBS ICV and n = 6 PDGF-CC ICV). (**I**) Active PDGF-CC core protein was stereotactically injected into the left lateral ventricle of C57BL/6N mice. 8 h post ICV, CSF was harvested and TNF-α and IL-1α levels were analyzed using ELISA. (n = 6 for PDGF-CC ICV and n = 7 for PBS-ICV). (**J**) qPCR analysis of vascular fragment cDNA isolated at the progression phase from EAE induced C57BL/6N mice treated with imatinib or PBS (n = 4 for each treatment group, n = 5 for naive mice). (**K**) qPCR analysis of vascular fragment cDNA isolated at the progression phase from EAE induced PDGF-CC^hum^ mice treated with anti-PDGF-CC mAb 6B3 or BM4 (n = 7 for each treatment group, n =  4 for naive littermates). (**L**) Representative images of PDGF-CC immunofluorescence staining on brain autopsies obtained from MS patients. Asterisk point to vascular PDGF-CC expression and arrowheads point to PDGF-CC expression in immune cells. Lesions were classified into active (AL, n = 5), chronic active (CAL, n = 5), chronic (CL, n = 5) and remyelinated (RL, n = 5) lesions. As control, normal appearing white matter areas (NAWM, n = 5) were evaluated. Scale bar: 15 μm. (**M**) Representative images of PDGFRαimmunohistochemistry performed on the same brain autopsies (adjacent tissue sections to those targeted for PDGF-CC) obtained from MS patients. Asterisk point to the perivascular PDGFRα expression (brown). Scale bar: 15 μm. Results are depicted as average ± SEM. Statistical evaluation for A was performed using Mann–Whitney (*P < 0.05, **P < 0.01, ***P < 0.001 and ****P < 0.0001). Statistics for E, G, J, and K were calculated using one-way ANOVA with Fisher´s LSD. Statistics for C, H and I were calculated using Student´s *t*-test. *^,#^*P* < 0.05; **^,##^*P* < 0.01; ***^,###^*P* < 0.001 and ****^,####^*P* < 0.0001). In **J** and **K**, * significance between Naive and EAE PBS treated mice; # significance between EAE PBS and EAE imatinib (**I**) and EAE-6B3 (**J**) treated mice. Abbreviations: CC ICV: PDGF-CC intracerebroventricular injection.

Since PDGFRα is expressed in perivascular cells in the NVU^57,61^ we aimed to investigate whether interfering with the PDGF-CC/PDGFRα axis is responsible for the imatinib-mediated preservation of BBB integrity in EAE by analyzing the effect of genetically reducing PDGF-CC levels utilizing *Pdgfc* deficient mice and neutralizing PDGF-CC monoclonal antibodies (mAb), respectively, in autoimmune neuroinflammation. Since *Pdgfc*^*-/-*^ mice on C57BL/6 background show multiple congenital defects in the CNS we decided to use heterozygous mice^24^. *Pdgfc*^+/-^ mice showed amelioration of disease symptoms compared to wild type littermate controls (Supplementary Fig. S7). We could even observe cases of complete recovery from clinical EAE (2 out of 6 mice in the *Pdgfc*^+/-^ group). For the antibody experiments, we utilized PDGF-CC^hum^ mice, which have a partially humanized PDGF-CC growth factor domain^66^. PDGF-CC^hum^ mice were used in order to allow in vivo antibody blocking with a murine anti-human PDGF-CC mAb. The mice were injected i.p twice weekly with 15 mg/kg anti-PDGF-CC mAb 6B3 or control IgG (BM4) from day 2 post MOG-immunization until the end of the experiment. Notably, in contrast to the control group, PDGF-CC^hum^ mice injected with mAb 6B3 exhibited less severe EAE course from the progression phase until the end of the experiment (Fig. 5A). We hypothesized that the disease amelioration achieved with blocking PDGF-CC was due to decreased disruption of BBB integrity. Indeed, 6B3 injected PDGF-CC^hum^ mice showed preserved BBB integrity as demonstrated by reduced extravasation of fluorescently labeled 70 kDa dextran (Fig. 5B,C) accompanied by decreased immune cell infiltration to the CNS during the progression phase (Fig. 5D,E). To assess whether blocking PDGF-CC acts directly on the BBB we performed ICV injections with active PDGF-CC core protein in naïve (non-immunized) mice 2–5 h post i.p. injection with either anti-PDGF-CC mAb 6B3 or BM4, respectively. Integrity of the BBB was subsequently assessed in all mice by measuring the extravasation of EB 2 h post ICV injection. Indeed, mice which received ICV injections of PDGF-CC in combination with the BM4 control antibody had significantly more EB leakage compared to the group that received the PDGF-CC blocking antibody (Fig. 5F,G). Moreover, extravasation of EB in the latter group was comparable to that in controls, which received ICV injection with PBS only.

To elucidate possible downstream PDGF-CC effectors in the NVU, we performed ICV injections of either active core PDGF-CC or PBS in naïve mice and isolated endothelial cell enriched vascular fragments 4 h later. Interestingly, qPCR analyses revealed upregulation of *Tnfa* and *Il1a*, which are known to be important for endothelial cell activation (Fig. 5H). To investigate if the transcriptional upregulation at the BBB translated into increased protein levels of TNF-α and IL-1α, we performed ICV injections and harvested cerebrospinal fluid (CSF) 8 h later. Indeed PDGF-CC injection triggered upregulation of both TNF-α and IL-1α in CSF (Fig. 5I). Of note, *Tnfa* and *Il1a* were correspondingly downregulated in the BBB from MOG-immunized, imatinib-treated mice (Fig. 5J), consistent with decreased TNF-α and IL-1α protein level in CSF collected from MOG-immunized imatinib-treated mice (Supplementary Fig. S8). In addition, *Tnfa* and *Il1a* were downregulated in MOG-immunized, PDGF-CC^hum^ mice treated with the anti-PDGF-CC blocking mAb 6B3 (Fig. 5K), suggesting that TNF-α and IL-1α may constitute PDGF-CC downstream targets at the BBB.

An extensive overlap of differentially expressed transcripts and disease-promoting pathways at the BBB, including upregulation of *Tnfa* and *Il1a*, exist between progression phase of EAE and an additional dataset acquired 24 h post middle cerebral artery occlusion (MCAO) in mice (Supplementary Fig. S9). In addition, comparisons with published datasets reveal a large overlap of our EAE BBB transcriptomes with BBB transcriptomes from other CNS disease models, including the MOG^35–55^ EAE model^44^ (Supplementary Tables S8 and S9).

In MS, we observed an upregulation of PDGF-CC in the lesion vasculature in contrast to the normal appearing white matter (NAWM) (Fig. 5L, asterisk). This applies to AL, CAL and CL, whereas, remyelinated lesions (RL) exhibited similar expression pattern of PDGF-CC as the NAWM. Interestingly, neuronal expression of PDGF-CC was reduced in MS lesions compared to NAWM. The PDGF-CC ligand was also expressed by immune cells infiltrating MS lesions (arrowheads in Fig. 5L). The PDGF-CC expression analysis was additionally complemented by PDGFRα immunohistochemistry which revealed its concordant upregulation around the lesion vasculature (Fig. 5M, asterisk) in AL, CAL and CL (Fig. 5M).

Taken together, our data show that blocking PDGF-CC in MS-like neuroinflammation decreases endothelial cell activation and downregulates *Tnfa* and *Il1a* at the BBB*,* which in turn preserves the integrity of the BBB and ameliorates the disease. In addition, upregulation of PDGF-CC and its receptor PDGFRα in MS indicates relevance of these targets in the disease treatment.

## Discussion

Loss of BBB integrity and subsequent recruitment of immune cells into the CNS parenchyma is a hallmark of MS and its animal model, EAE^31, 52^. By systematic gene profiling of ex vivo isolated endothelial cell enriched vascular fragments from three subsequent disease phases of EAE (preclinical, progression and remission), we have generated a unique BBB transcriptome database with correlation to structural and functional changes at the BBB. The uncovering of disease phase-related transcriptional differences at the BBB presents a novel source of potential candidate genes that can be targeted at different phases of MS. Combined with data from our previous study^2^, the present study strongly suggests modulation of PDGFRα signaling as a novel therapeutic approach to restore BBB integrity during the progression phase of EAE, which in turn attenuates further disease exacerbation. As initially reported^2^ and now functionally demonstrated, blocking PDGFRα with the small tyrosine kinase inhibitor imatinib or its ligand PDGF-CC with neutralizing antibodies, respectively, resulted in EAE amelioration and a better-preserved BBB.

Based on transcriptome and pathway analyses of endothelial cell enriched vascular fragments of the BBB we here show that blocking PDGFRα in EAE with imatinib resulted in downregulation of multiple signaling pathways critical for endothelial cell activation, immune cell transmigration and angiogenesis during disease progression. Amelioration of the clinical symptoms in the imatinib-treated group was associated with reduced TJ degradation and better preservation of BBB function and integrity. PDGF-CC is the prevailing PDGFRα ligand regulating BBB integrity^57^, and similar protective effect as seen with imatinib was observed upon specifically blocking PDGF-CC in EAE-induced mice. Restoration of BBB function and integrity by blocking PDGF-CC is likely based on downregulation of the endothelial cell activators TNF-α and IL-1α since *Tnfa* and *Il1a* expression were specifically increased in CNS vascular fragment isolates upon PDGF-CC ICV injections and correspondingly found to be downregulated in MOG-immunized, PDGF-CC^hum^ mice treated with the PDGF-CC blocking mAb. Indeed, TNF-α and IL-1 have been shown to potentiate BBB disruption in vivo and endothelial cell permeability in vitro^47^.

Endothelial cells can be activated by proinflammatory cytokines such as TNF-α, IL-6, IL-1 and IFNγ, which facilitates the recruitment and attachment of circulating leucocytes to the vessel wall. For example, knock down of IL-1R1 in endothelial cells delays EAE onset and severity^39^. Endothelial IL-1R1 has an effect on both adhesion and transmigration of leucocytes across the BBB and recruitment of additional leucocytes^45^. TNF-α and IFNγ modulate the expression of a wide variety of chemokines, cytokines and CAMs for example CXCL8, CXCL9, CXCL10, CX3CL1, CCL2, CCL3, CCL4 and CCL5^14,29,59,64^, promoting both adhesion of leucocytes to endothelial cells and migration of leucocytes across the BBB endothelial cells through upregulation of ICAM-1 and VCAM-1^49,64^ as well as E- and P-selectins^12,63^. We found downregulation of *Ccl2, Ccl3, Ccl4, Cxcl9, Cxcl10, Icam1, Sele* and *Selp* in vessel isolates of imatinib-treated mice during disease progression. Thus, our results suggest that the pathway initiated by the PDGF-CC/PDGFRα signaling in the NVU potentially regulates the expression of TNF-α and IL-1α during EAE, and through these factors increase endothelial cell activation and ultimately disruption of BBB integrity. The presumed endothelial source of TNF-α and IL-1α expression in our expression analysis is intriguing and may represent an autocrine mode of action.

Upregulation of proinflammatory markers in endothelial cells in EAE is in line with a recent publication based on a different approach to endothelial cell isolation and high sensitivity RNA sequencing instead of microarray-based analysis, strengthening the concept of BBB dysfunction and an altered BBB immune profile contributing to CNS diseases^44^.

Upregulation of PDGF-CC in the vascular bed of MS lesions was accompanied by PDGF-CC + infiltrating immune cells. Of note, we also detected upregulation of PDGFRα around the vessels in MS lesions in contrast to NAWM. This suggests that the PDGF-CC/PDGFRα axis also plays an important role in MS. Activation of PDGF-CC/PDGFRα signaling during MS is potentially due to tPA-mediated activation of latent PDGF-CC in the CNS. Indeed, elevated tPA levels in CSF have been reported in MS patients^4^. In addition, in MS, an increased tPA expression in neurons and in perivascular inflammatory cells has been documented, and high tPA activity in the circulation has been shown to correlate with disease progression^5,16^. Similarly, data from animal studies show correlation of plasma tPA levels with the clinical signs of EAE^48^. tPA activity is also increased in inflammatory lesions in the EAE model^60^ and *tPA*^-/-^ mice show a delayed onset of EAE; however, symptoms are more severe^42^. The delayed onset can possibly be explained by decreased BBB leakiness in the acute phase due to lack of tPA-mediated PDGF-CC activation, whereas the more severe delayed symptoms can be explained by less regenerative capacity in the absence of tPA. Fibrin is known to limit axonal regeneration^20^ and genetic deficiency of the endogenous tPA inhibitor PAI-1, leading to increased tPA activity, resulted in higher fibrinolysis capacity, accompanied by less axonal damage and brain inflammation^3^. This is in line with our data since we show that imatinib and mAb 6B3 are most effective during the EAE progression phase.

Current knowledge suggests differences in pathological mechanisms underlying early and late stages of MS, which are commonly manifested as relapsing–remitting MS (RRMS) and progressive MS (PMS) clinical course, respectively. Thus early stages of MS are associated with profound BBB leakage, as demonstrated by contrast enhancement on magnet resonance (MR) images, which enables entry of circulating inflammatory cells into the CNS^33^. On the other hand, PMS disease course is characterized by less extensive inflammation behind a nearly intact BBB^27^. While expression profiles of the brain vasculature during the progression phase of EAE exhibit similarities with pathological changes at the BBB during disease relapse in RRMS^55^, it is tempting to speculate analogy between the remission phase of EAE and PMS. In this sense, the remission phase of EAE was characterized by: (i) downregulation of proinflammatory and proangiogenic cytokines at the BBB comparing to the progression phase, (ii) partially restored BBB including upregulation of *Cldn1*, which was previously shown to facilitate resealing of the TJ of the compromised BBB in EAE^46^ and (iii) an upregulation of various transcripts involved in lipid and cholesterol metabolism, especially those important for fatty acid and cholesterol transport. Interestingly, simvastatin, a 3-hydroxy-3-methylglutaryl-CoA (HMG-CoA) reductase inhibitor used for treating hyperlipidaemia, was associated with overall reduced brain atrophy in secondary progressive MS (SPMS)^13^. Increased levels of plasma cholesterol and triglyceride-rich lipoproteins (TGRL) have been associated with cerebrovascular inflammation, vascular dementia and Alzheimer´s disease^35^ and increased TGRL lipolysis were shown to disturb the BBB and induce lipid droplets in astrocytes^34^. The accumulation of lipid droplets was associated with upregulation of the proinflammatory NFκB pathway and secretion of proinflammatory cytokines. Moreover, glial lipid droplets and reactive oxygen species (ROS) formation attributable to mitochondrial dysfunction were shown to promote neurodegeneration^41^. Thus, our data indicating increased cholesterol uptake and esterification, as well as increased lipid uptake during the remission phase of EAE, may implicate accumulation of lipid droplets at the BBB. Thus, a thorough investigation of lipid and cholesterol metabolism during the remission phase of EAE may contribute to further elucidation of MS pathology.

Understanding molecular mechanisms controlling BBB function and integrity in health and disease is a prerequisite for developing novel therapeutic strategies. Unlike currently available MS treatments such as natalizumab, a humanized antibody against the cell adhesion molecule α4-integrin which is associated with developing PML, an opportunistic infection caused by the John Cunningham (JC) virus^26^, imatinib exhibit much less severe side effects and is used for treating cancer since many years^43^. In blood, imatinib is strongly bound to plasma proteins, *e.g.* albumin and alpha-1-acid glycoprotein and will gain access to the CNS parenchyma by co-leakage through a permeable BBB^8^. Imatinib will however also be transported out from CNS via the P-glycoprotein efflux system, probably explaining the higher doses needed for CNS implications^50,62^. Recently, a phase II clinical trial (NCT03674099) to test safety and efficacy of imatinib treatment in RRMS was initiated. In addition to the PDGFRα antagonist imatinib, the anti-PDGF-CC mAb 6B3 represents a prospective therapy for brain disorders characterized by a leaky BBB. Blocking PDGF-CC with mAb 6B3 could provide an even more superior treatment for MS than imatinib, since a mAb likely excludes off-target effects, and thus reduces potential adverse side effects. Importantly, in a long-term toxicology study in mice with mAb 6B3 we could not observe any adverse side effects^66^.

Besides EAE, beneficial therapeutic effects of imatinib treatment were also demonstrated in animal models of other neuropathologies associated with a compromised BBB such as amyotrophic lateral sclerosis (ALS)^37^, spinal cord injury (SCI)^1^, traumatic brain injury (TBI)^58^ and seizures^25^. We have also shown that targeting PDGFRα signalling with imatinib reduces stroke volume and BBB disruption after MCAO in mice^57^. Interestingly, we could detect an extensive overlap of differentially expressed transcripts and disease-promoting pathways at the BBB between the data sets from progression phase of EAE and 24 h post MCAO, as well as with published datasets from various other CNS disease models^44^, which points to a common injury response elicited at the BBB in the respective disease models.

In summary, we here show that blocking either PDGFRα, or its ligand PDGF-CC, leads to amelioration of a MS-like neuroinflammation through restoration of BBB function and integrity. Since PDGF-CC/PDGFRα signaling-related disruption of the BBB and neurovascular dysfunction are common features of several neuropathologies, agents blocking this pathway are likely to attain larger therapeutic range than currently indicated.

## Supplementary Information

Supplementary Information 1.

Supplementary Information 2.

## Data Availability

Raw data are deposited on the NCBI Gene Expression Omnibus database (accession no. GSE150562 and no. GSE157604).
