# Supplementary material for: Blocking PDGF-CC signaling ameliorates multiple sclerosis-like neuroinflammation by inhibiting disruption of the blood–brain barrier
Source: Sci Rep. 2020 Dec 24;10:22383. doi: 10.1038/s41598-020-79598-z (PMC7759579; doi:10.1038/s41598-020-79598-z)
Supplement: Supplementary file 1 — Supplementary Information 1. [file 41598_2020_79598_MOESM1_ESM.docx]

**Blocking PDGF-CC signaling ameliorates multiple sclerosis-like neuroinflammation by inhibiting disruption of the blood-brain barrier**

Manuel Zeitelhofer^1§^, Milena Z. Adzemovic^1,2^, Christine Moessinger^1,#^, Christina Stefanitsch^1,#^, Carina Strell^3^, Lars Muhl^1^, Lou Brundin^2^, Linda Fredriksson^1^, Tomas Olsson^2^, Ulf Eriksson^1^ and Ingrid Nilsson^1§^

^1^ Division of Vascular Biology, Department of Medical Biochemistry and Biophysics, Karolinska Institutet, 171 77 Stockholm, Sweden

^2^ Neuroimmunology Unit, Department of Clinical Neuroscience, Center for Molecular Medicine, Karolinska University Hospital, 171 76 Stockholm, Sweden

^3^ Department of Immunology, Genetics and Pathology, Uppsala University, 75185 Uppsala, Sweden

^#^ equal contribution

^§^To whom correspondence and reprint requests should be addressed:

Manuel Zeitelhofer; e-mail: manuel.zeitelhofer@ki.se

or Ingrid Nilsson; e-mail: ingrid.nilsson@ki.se

**Supplementary legends**

**Supplementary Figure 1. Characterization of the cellular composition of vascular fragments during MOG-EAE.** (A-D) Vascular fragments were isolated from spinal cords with an antibody against CD31 (PECAM1) and subsequently mRNA and cDNA were generated for quantitative real-time PCR analysis. Primers for different cell type specific markers were used to assess the percentage of the distinct cell populations in the vascular fragments isolated from EAE preclinical (B), progression (C) and remission (D) phase (n=4 for each disease phase and treatment group, respectively), as well as from naïve controls (n=5) (A). For enrichment calculation we compared the amount of the respective specific marker in the vascular fragment fraction (CD31 bound fraction) versus the wash fraction (remaining cells from the spinal cord). Cldn5 was set to 10-fold. The analysis revealed high enrichment of endothelial cells (5.8-7.5 fold for *Pecam1* and 10-fold for *Cldn5*), followed by pericytes (0.7-1 fold) and astrocyte endfeet (0.1-0.78 fold). The vascular fragments were depleted of neurons (0.002-0.005 fold), microglia (0.003-0.11 fold) and showed neglectable contamination from immune cells (0.07-0.2 fold for *Cd4*, 0-0.08 fold for *Lat* and 0.07-0.18 fold for *Itgam*) that was similar for EAE and naive vascular fragments. The values represent the relative percentage of each cell type specific marker in the vascular fragments. These results indicate high purity of the vascular fragments with very strong enrichment for endothelial cells and neglectable contaminations from neurons or immune cells in both EAE induced and naïve non-immunized mice. (E) Overview of the enrichment in the 3 EAE disease phases and naive controls. (F-G) Representative images showing CD31 immunofluorescence staining (in white) exclusively in endothelial cells during EAE. No co-localization of CD31 and inflammatory cell infiltrates (visualized by DAPI in blue) was observed. Higher magnification shown in G. n.d., not detectable. Scale bars in F and G: 60 and 15 μm. Results are depicted as average ± SEM.

**Supplementary Figure 2. Imatinib treatment decreases demyelination and inflammatory cell recruitment.** Group representative paraffin embedded spinal cord cross-sections from imatinib (B and D) and PBS (A and C) treated mice during the progression (A and B) and remission phase (C and D) stained for myelin with Kluever (lower row) and Hematoxylin-eosin (HE; upper row), respectively. During progression as well as remission, imatinib-treated mice showed no signs of demyelination (Kluever) and only very weak signs of inflammation (HE), whereas PBS treated mice exhibited moderate loss of myelin and vast immune cell infiltration in the spinal cord white matter lesions (arrows). (E) Graphical representation of the inflammation parameters calculated as inflammatory index (I.I.) and demyelination score (D.M.) for spinal cords, during progression (n=3 mice in each group) and remission phase (n=5 mice in each group). Results are depicted as average ± SEM. Statistics were calculated using one-way ANOVA with Fisher´s LSD *^###^P*<0.0001, *^####^P*<0.00001.

**Supplementary Figure 3. Imatinib treatment does not affect the BBB transcriptome in naïve mice.** Vascular fragments from healthy naïve (non-immunized) mice were isolated after 2 weeks of gavaging with imatinib or PBS (n=3/group). mRNA prepared from vascular fragments was hybridized to Affymetrix mouse Gene 2.0 ST arrays. (A) Heat map diagram of the 2000 most affected transcripts (according to *P*-value) using hierarchical clustering. No significant statistical difference could be detected using an adjusted *P* value *<* 0.05 and a log 2-fold change (1). (B) Heat map diagram of chemokines using hierarchical clustering. No difference between imatinib and PBS gavaged naïve mice.

**Supplementary Figure 4.** **Blocking PDGFR**α **signaling down-regulates transcripts involved in lipid metabolism during the remission phase.** C57BL/6N mice were immunized with MOG to induce EAE, treated either with imatinib or PBS from day 2 p.i. until the end of the experiment and subjected to vascular fragment isolation at the preclinical, progression and remission phase. (A) During the remission phase, genes important for cholesterol and fatty acid transport were up-regulated as indicated in the LXR/RXR pathway scheme which was generated by Ingenuity. Molecules in pink indicate that these molecules are differentially regulated during the remission phase whereas molecules in white are not regulated. (B) Quantitative real-time PCR analyses of vascular fragment cDNA of imatinib or PBS treated EAE induced mice and naive mice at the preclinical, progression and remission phase. The cholesterol transporter *Abca1* and *Apoc1* were down-regulated by imatinib specifically during the remission, whereas *Lpl* and *Fabp5* were down-regulated during both the remission and progression phase in response to imatinib treatment.

Results are depicted as average ± SEM. Statistics were calculated using one-way ANOVA with Fisher´s LSD and *P* values <0.05 were considered significant (*P*<0.05=*^/#^, *P*<0.01=**^/##^, *P*<0.001=*** and *****P* *<* 0.0001). n= 4 mice for each group for onset, progression and remission phase, n=5 mice for naive mice. * significance between naive towards EAE induced mice; # significance between disease phase EAE PBS and EAE imatinib treated mice.

**Supplementary Figure 5. Protein staining confirms transcriptome analysis.** (A and B) Spinal cords of EAE immunized mice were cryo-embedded and immunofluorescent stainings for ADAMTS9 and CXCL10 were performed on spinal cord cross-sections. Confocal analysis showed that ADAMTS9 (A) co-localized with aquaporin 4 (AQP4), a marker for astrocyte endfeet and CXCL10 (B) co-localized with the endothelial cell marker podocalyxin (PODO), confirming the expression in the neurovascular unit. Scale bars in A and B are 10 and 20 μm.

**Supplementary Figure 6. Loss of tight junction integrity in EAE can be rescued by blocking PDGFRα**. C57BL/6N mice were immunized with MOG to induce EAE, treated either with imatinib or PBS from day 2 p.i. until the end of the experiment and spinal cords were harvested at the preclinical, progression and remission phase. Spinal cord cross-sections were stained with anti-occludin (A), anti-claudin-5 (B) or anti-ZO-1 (C) to visualize tight junctions (n=4-5 for each disease phase and treatment group, respectively, and n=3 for naive control mice). For all tight junction markers analysed the quantifications are shown on the left side of each panel. For claudin-5 and ZO-1 high magnification images are shown for all disease phase and treatment settings. Scale bars: 20 μm. Results are depicted as average ± SEM. One-way ANOVA with Fisher´s LSD (*,^#^P<0.05; **,^##^P<0.01; ***,^###^P<0.001 and ****,^####^P< 0.0001) was used.

**Supplementary Figure 7. Reduced *Pdgfc* levels in *Pdgfc^+/-^* mice lead to amelioration of EAE symptoms.** *Pdgfc^+/-^* mice were immunized with recombinant MOG to induce EAE. *Pdgfc^+/-^* mice developed less severe EAE compared to wt littermate controls (n = 6 for *Pdgfc^+/-^* and n=8 for wt, respectively, 1 representative of 2 independent experiments shown). Statistics were performed using Mann–Whitney (**P <* 0.05, ***P <* 0.01, ****P* *<* 0.001 and *****P* *<* 0.0001). Results are depicted as average ± SEM.

**Supplementary Figure 8. Imatinib downregulates IL-1**α **and TNF-**α **in the CSF during EAE.** C57BL/6N mice were immunized with MOG to induce EAE and treated either with the PDGFRα inhibitor imatinib (n=5) or PBS (n=5) from day 2 p.i. until the end of the experiment. CSF was harvested during the progression phase and the quantity of IL-1α and TNF-α was analysed with ELISA. Both IL-1α and TNF-α were significantly downregulated in CSF of imatinib treated mice. Results are depicted as average ± SEM. Statistics were performed using Student´s *t*-test (**P <* 0.05, ***P <* 0.01, ****P* *<* 0.001 and *****P* *<* 0.0001).

**Supplementary Figure 9.** **The BBB transcriptomes during EAE progression and 24h post middle cerebral artery occlusion (MCAO) share a common gene signature suggestive of a common injury response.** (A) 696 shared transcripts were differentially expressed during EAE progression and 24h post MCAO. (B) Selected canonical pathways that are affected during both EAE progression and 24h post MCAO. (C) Molecules important for leucocyte diapedesis and transmigration differentially expressed in EAE progression and 24h post MCAO.

An adjusted *P* value <0.05 and a log 2-fold change (1) was used as statistical cut off for all our analyses. For canonical pathway analyses the significance of the association between the data set and the canonical pathway was measured in 2 ways: 1) A ratio of the number of molecules from the data set that map to the pathway divided by the total number of molecules that map to the canonical pathway is displayed. 2) Fisher’s exact test was used to calculate a *P* value determining the probability that the association between the genes in the data set and canonical pathway is by chance alone.

**Supplementary Table 1. Primers used for real-time quantitative PCR analyses**

**Supplementary Table 2.** All differentially expressed transcripts in vascular fragments from MOG-EAE induced mice during the preclinical phase compared to naive mice are listed. An adjusted *P* value lower than 0.05 and a log 2-fold change (1) was used as statistical cut off.

**Supplementary Table 3.** All differentially expressed transcripts in vascular fragments from MOG-EAE induced mice during the progression phase compared to naive mice are listed. An adjusted *P* value lower than 0.05 and a log 2-fold change (1) was used as statistical cut off.

**Supplementary Table 4.** All differentially expressed transcripts in vascular fragments of MOG-EAE induced mice during the remission phase compared to naive mice are listed. An adjusted *P* value lower than 0.05 and a log 2-fold change (1) was used as statistical cut off.

**Supplementary Table 5.** Differentially expressed transcripts in vascular fragments common during preclinical, progression and remission phase compared to naïve mice. An adjusted *P* value lower than 0.05 and a log 2-fold change (1) was used as statistical cut off.

**Supplementary Table 6.** All differentially expressed transcripts in vascular fragments from PBS compared to imatinib treated MOG-EAE induced mice during the progression phase are listed. An adjusted *P* value lower than 0.05 and a log 2-fold change (1) was used as statistical cut off.

**Supplementary Table 7.** All differentially expressed transporters in vascular fragments from MOG-EAE induced compared to naive mice during preclinical, progression remission phase as well as from imatinib compared to PBS treated mice during the progression phase are listed. An adjusted *P* value lower than 0.05 and a log 2-fold change (1) was used as statistical cut off.

56 transporters were differentially regulated in the progression phase (6 of the ATP binding cassette, 10 of the ATPase and 36 of the Slc family), 29 transporters show differential expression in the remission stage (6 of the ATP binding cassette, 8 of the ATPase and 14 of the Slc family) and 2 Slc type transporters show differential expression in the preclinical phase. Imatinib treatment affected 33 transporters during the progression phase (3 of the ATP binding cassette, 3 of the ATPase and 24 of the Slc family). Notably, monocarboxylate transporters *Slc16a1* and *Slc16a4* required for pyruvate, lactate and ketone body transport as well as receptor-mediated transporters of the BBB *Tfrc, Lepr* and *Igf1r* were down-regulated in PBS but not imatinib treated mice. Already during the preclinical phase, *Slc16a1* was downregulated while none of the monocarboxylate transporters was affected during disease remission. The data indicates that the CNS and especially neurons (as they are the most sensitive cells in terms of constant energy requirement) are exposed to energy deprivation from the preclinical disease stage throughout disease progression.

Fold change progression (I) refers to the comparison of imatinib versus PBS treated mice during the progression phase.

**Supplementary Table 8.** **Comparison of the overlap between the BBB transcriptomes during EAE and the ‘core BBB dysfunction module' gene list from Munji et al. [43]**

The majority (70.4%) of the 54 differentially regulated genes in the BBB common for four different CNS disease models (seizure, EAE, stroke and TBI), termed the ‘BBB dysfunction module’ [43] is also differentially expressed in our EAE BBB transcriptome analysis. A large proportion, 60.5% of those genes showed anticorrelated expression upon imatinib treatment. Differentially expressed genes in the BBB transcriptome from EAE progression PBS treated, EAE remission PBS treated and EAE progression imatinib treated mice are depicted with colored boxes. An adjusted *P* value <0.05 and a log 2-fold change (1) was used as statistical cut off.

**Supplementary Table 9.** **Comparison of the overlap between the BBB transcriptomes during EAE and the 'BBB dysfunction module' from Munji et al. comprising genes in common for at least three out of four CNS disease models [43]**

The majority (67.7%) of the differentially regulated genes in the BBB in common for at least three out of four different CNS disease models (seizure, EAE, stroke and TBI), extracted from Munji et al. [43], are also differentially expressed in our EAE BBB transcriptome analysis. 57.6% of those genes showed anticorrelated expression upon imatinib treatment. The 54 genes from the ‘core BBB dysfunction module’ are not included in the table. Differentially expressed genes in the BBB transcriptome from EAE progression PBS treated, EAE remission PBS treated and EAE progression imatinib treated are depicted with colored boxes. An adjusted *P* value <0.05 and a log 2-fold change (1) was used as statistical cut off.
