# Supplementary material for: Blocking PDGF-CC signaling ameliorates multiple sclerosis-like neuroinflammation by inhibiting disruption of the blood–brain barrier
Source: Sci Rep. 2020 Dec 24;10:22383. doi: 10.1038/s41598-020-79598-z (PMC7759579; doi:10.1038/s41598-020-79598-z)
Supplement: Supplementary file 2 — Supplementary Information 2. [file 41598_2020_79598_MOESM2_ESM.pdf]

Blocking PDGF-CC signaling ameliorates multiple sclerosis-like neuroinflammation by inhibiting disruption of the blood-brain barrier

Manuel Zeitelhofer, Milena Z. Adzemovic, Christine Moessinger, Christina Stefanitsch, Carina Strell, Lars Muhl, Linda Fredriksson, Tomas Olsson, Ulf Eriksson and Ingrid Nilsson

Supplementary Figure 1

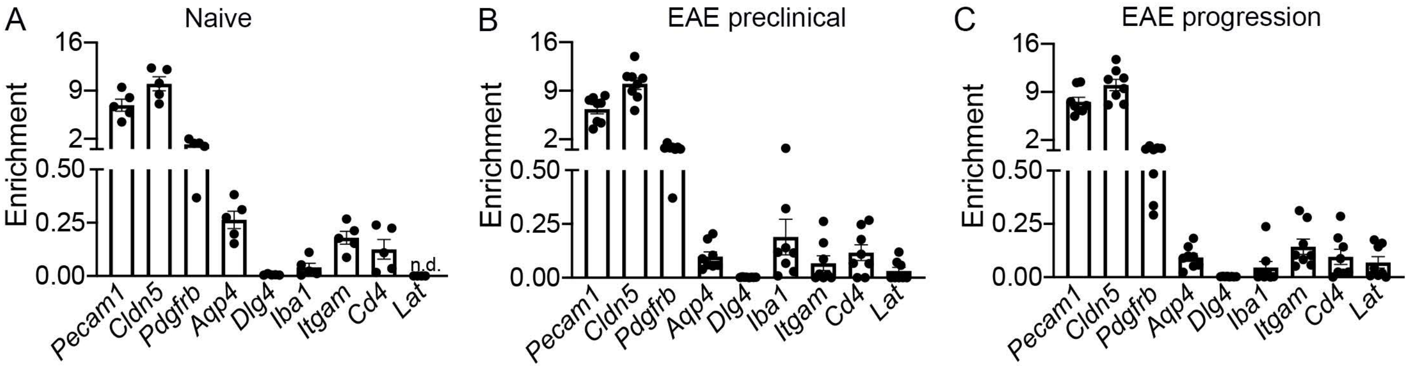

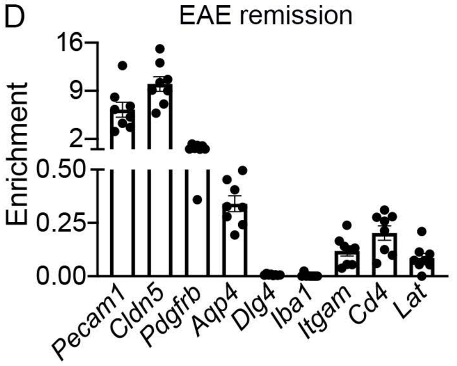

E

	Naive	Preclinical	Progression	Remission
Cldn5	10	10	10	10
Pecam1	7	6	8	6
Pdgfrb	1	0,8	0,7	0,7
Aqp4	0,3	0,1	0,09	0,3
Dlg4	0,006	0,002	0,003	0,007
Iba1	0,04	0,2	0,04	0,003
Itgam	0,2	0,07	0,1	0,1
Cd4	0,1	0,1	0,1	0,2
Lat	0	0,03	0,07	0,09

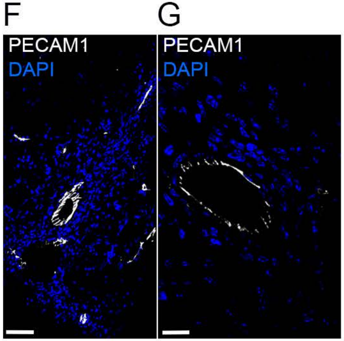

Blocking PDGF-CC signaling ameliorates multiple sclerosis-like neuroinflammation by inhibiting disruption of the blood-brain barrier

Manuel Zeitelhofer, Milena Z. Adzemovic, Christine Moessinger, Christina Stefanitsch, Carina Strell, Lars Muhl, Linda Fredriksson, Tomas Olsson, Ulf Eriksson and Ingrid Nilsson

Supplementary Figure 2

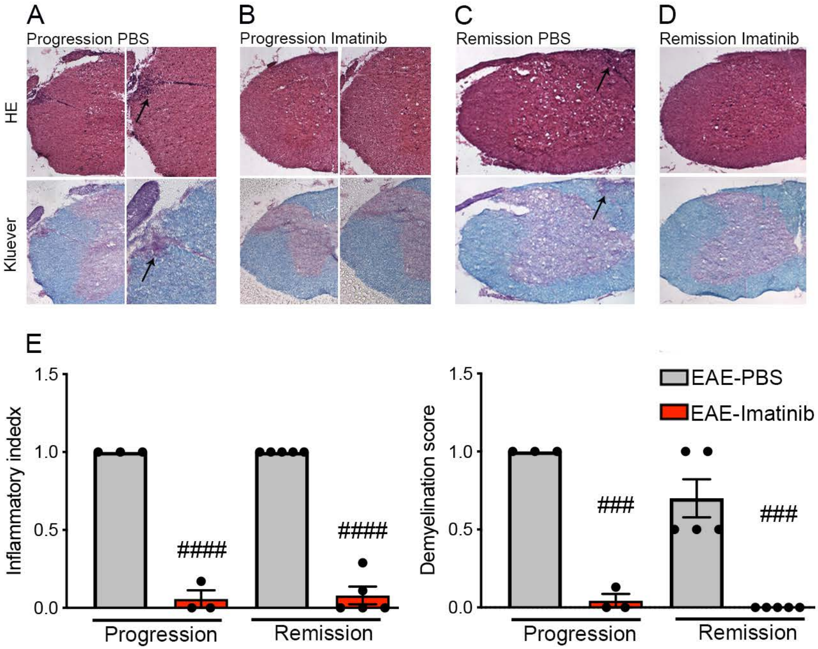

Blocking PDGF-CC signaling ameliorates multiple sclerosis-like neuroinflammation by inhibiting disruption of the blood-brain barrier

Manuel Zeitelhofer, Milena Z. Adzemovic, Christine Moessinger, Christina Stefanitsch, Carina Strell, Lars Muhl, Linda Fredriksson, Tomas Olsson, Ulf Eriksson and Ingrid Nilsson

Supplementary Figure 3

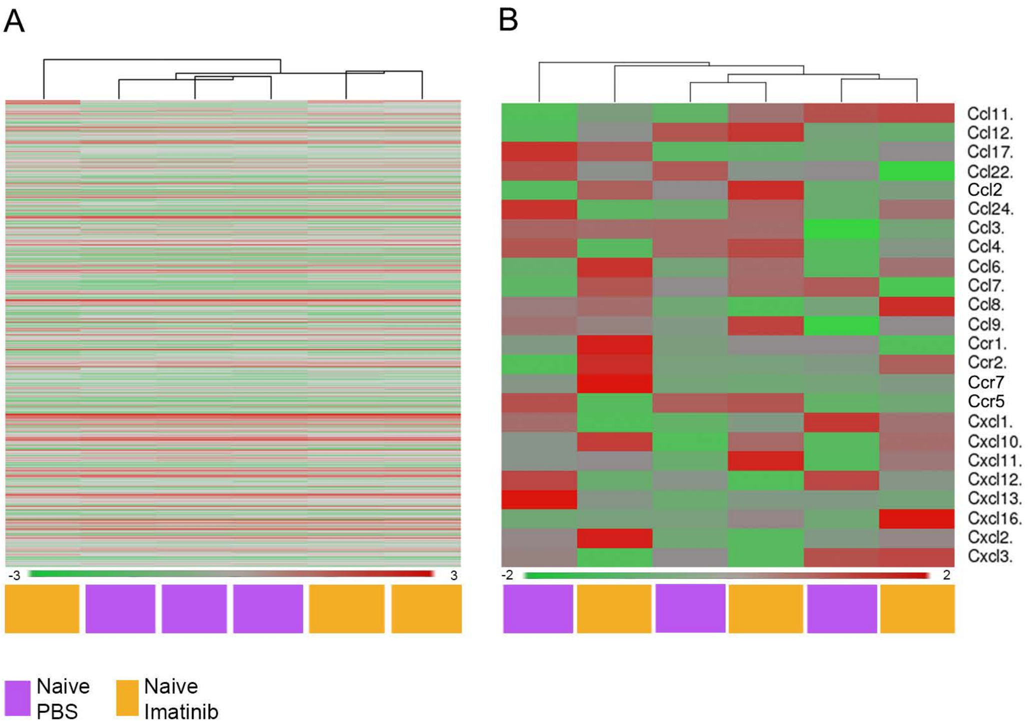

Blocking PDGF-CC signaling ameliorates multiple sclerosis-like neuroinflammation by inhibiting disruption of the blood-brain barrier

Manuel Zeitelhofer, Milena Z. Adzemovic, Christine Moessinger, Christina Stefanitsch, Carina Strell, Lars Muhl, Linda Fredriksson, Tomas Olsson, Ulf Eriksson and Ingrid Nilsson

Supplementary Figure 4

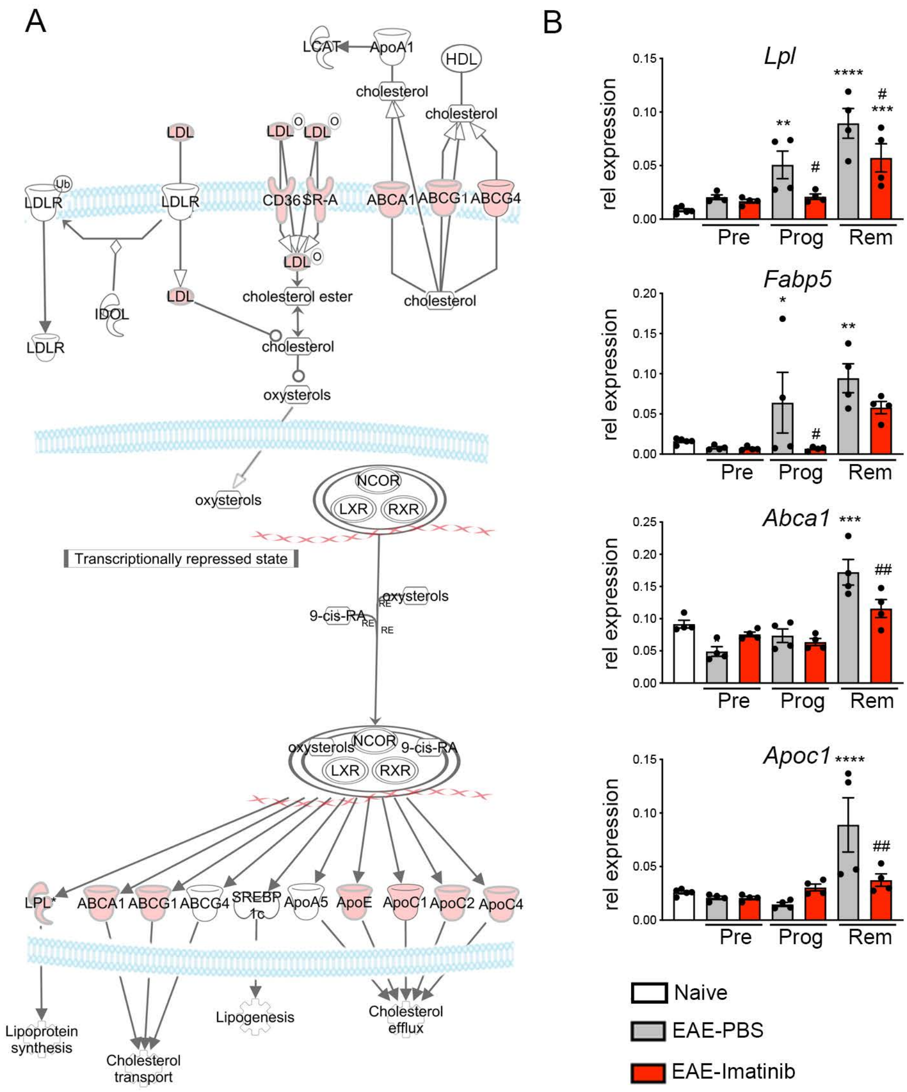

Blocking PDGF-CC signaling ameliorates multiple sclerosis-like neuroinflammation by inhibiting disruption of the blood-brain barrier

Manuel Zeitelhofer, Milena Z. Adzemovic, Christine Moessinger, Christina Stefanitsch, Carina Strell, Lars Muhl, Linda Fredriksson, Tomas Olsson, Ulf Eriksson and Ingrid Nilsson

Supplementary Figure 5

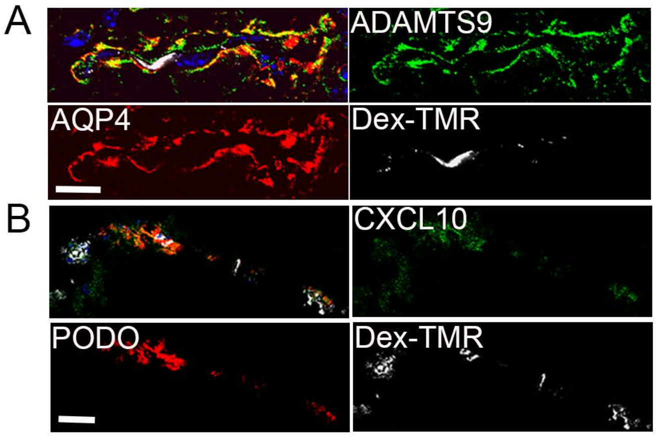

Blocking PDGF-CC signaling ameliorates multiple sclerosis-like neuroinflammation by inhibiting disruption of the blood-brain barrier

Manuel Zeitelhofer, Milena Z. Adzemovic, Christine Moessinger, Christina Stefanitsch, Carina Strell, Lars Muhl, Linda Fredriksson, Tomas Olsson, Ulf Eriksson and Ingrid Nilsson

Supplementary Figure 6

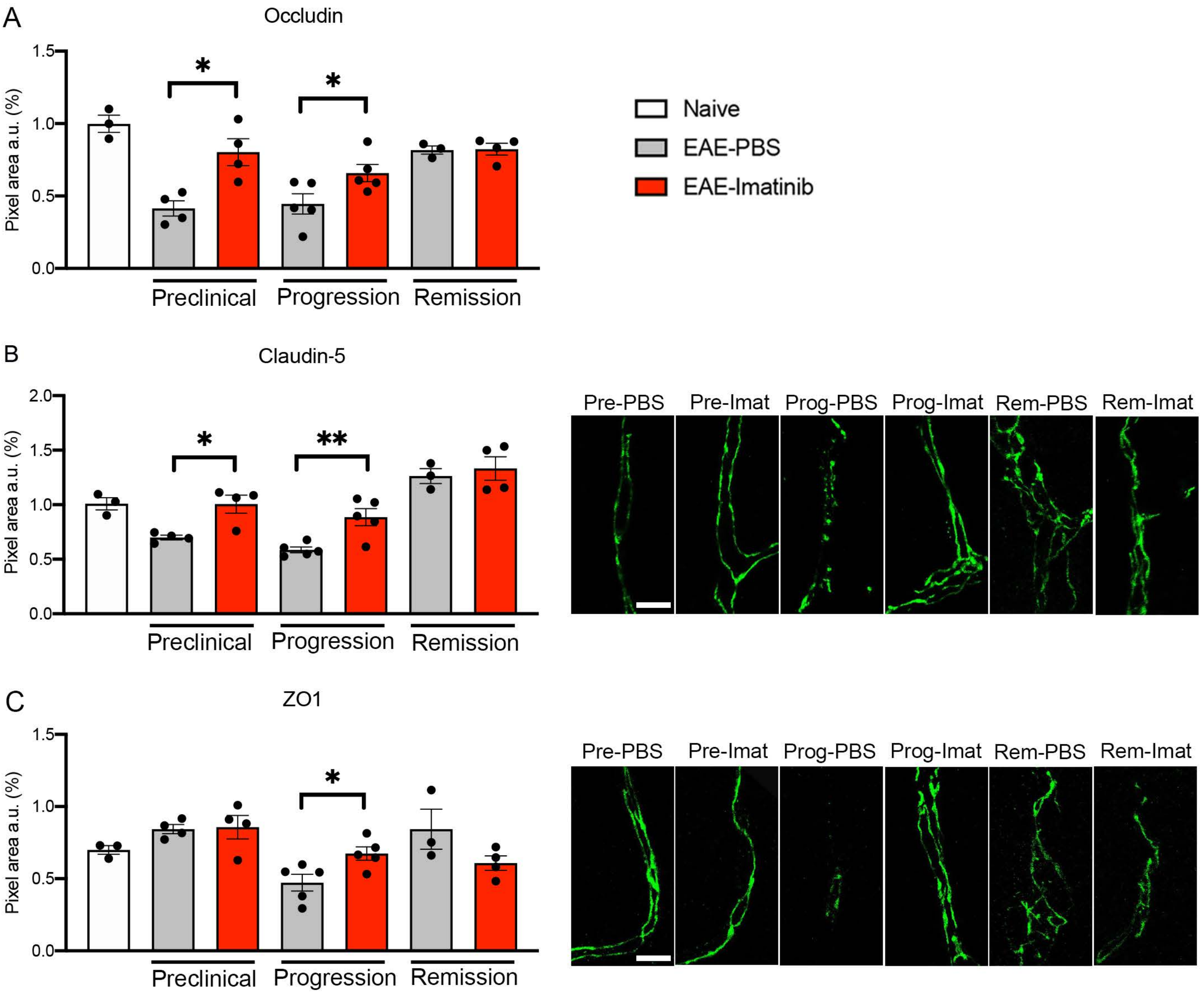

Blocking PDGF-CC signaling ameliorates multiple sclerosis-like neuroinflammation by inhibiting disruption of the blood-brain barrier

Manuel Zeitelhofer, Milena Z. Adzemovic, Christine Moessinger, Christina Stefanitsch, Carina Strell, Lars Muhl, Linda Fredriksson, Tomas Olsson, Ulf Eriksson and Ingrid Nilsson

Supplementary Figure 7

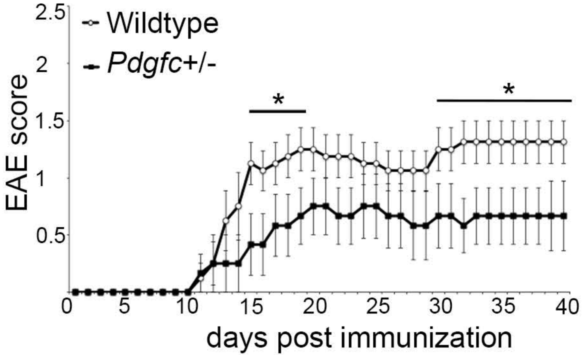

Blocking PDGF-CC signaling ameliorates multiple sclerosis-like neuroinflammation by inhibiting disruption of the blood-brain barrier

Manuel Zeitelhofer, Milena Z. Adzemovic, Christine Moessinger, Christina Stefanitsch, Carina Strell, Lars Muhl, Linda Fredriksson, Tomas Olsson, Ulf Eriksson and Ingrid Nilsson

Supplementary Figure 8

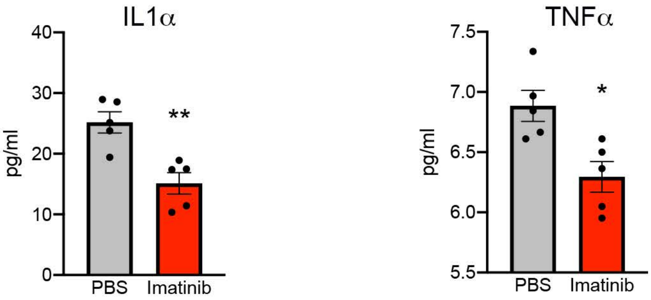

Blocking PDGF-CC signaling ameliorates multiple sclerosis-like neuroinflammation by inhibiting disruption of the blood-brain barrier

Manuel Zeitelhofer, Milena Z. Adzemovic, Christine Moessinger, Christina Stefanitsch, Carina Strell, Lars Muhl, Linda Fredriksson, Tomas Olsson, Ulf Eriksson and Ingrid Nilsson

Supplementary Figure 9

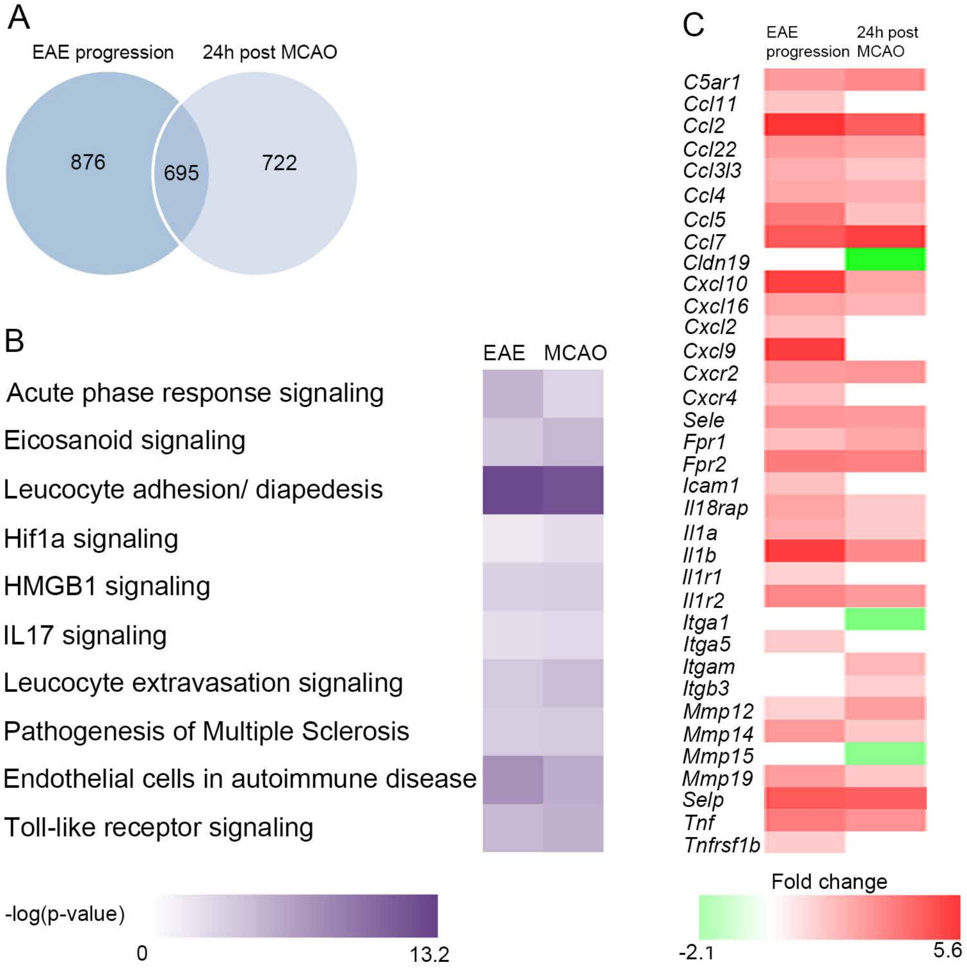

Supplementary Table 1. Primers for expression validation and enrichment analysis

Gene symbol	Primers
Rpl19	GGTGACCTGGATGAGAAGGA
	TTCAAGCTTGTGGATGTGCTC
Cxcl10	AGA ACG GTG CGC TGC AC
	CCT ATG GCC CTG GGTCTCA
Adamts9	TGTTCTGTGACACCCTGTGG
	TACCTTGCCCACAAGTCACG
Lpl	CATGGATGGACGGTAACGGG
	TTCTCTCTTGTACAGGGCGG
Abca1	TGGCAAGGTTGGTGAATGGG
	AGAGCTTTCGTTTGTTGCCG
Apoc1	GACCTTGGAAGGCCCAGC
	GATATGTTCAATGGCTGCCCG
Fabp5	GTCTGCACCTTCCAAGACGG
	ACACACTCCACGATCATCTTCC
Irak3	GCGTAGTGTGACTTTGTGGC
	AGTGCCGGTGGTAATGATGG
Selp	CCTGGCAAGTGGAATGATGA
	AAGCTGCAGACTGACTGGTA
Il17a	CAGAAGGCCCTCAGACTACC
	CTTCATTGCGGTGGAGAGTCC
Tnfa	CTGTAGCCCACGTCGTAGC
	ACAAGGTACAACCCATCGGC
Il1r1	CCTGAACGGGTTTAGTTCGG
	GTACATACGTCAATCTCCAGCG
Mmp8	AAGACAAGTACTTCTGGAGACGG
	CCTGAAGACCGTTGGGTAGG
Il1a	ATCAGCAACGTCAAGCAACG
	AAGGTGCTGATCTGGGTTGG
Sele	AGCTACCCATGGAACACGAC
	ACGCAAGTTCTCCAGCTGTT
Hif1a	GCTTACACACAGAAATGGCCC
	GATGTTCATCGTCCTCCCCC
Pecam1	TACTGGGCTTCGAGAGCATT
	AGAGACGGTCTTGTCGCAGT
Cldn5	GTGGAACGCTCAGATTTCAT
	TGGACATTAAGGCAGCATCT
Pdgfrb	CACCTTCTCCAGTGTGCTGA
	GGAGTCCATAGGGAGGAAGC
Dlg4	CGCCCCCTCTGGAACACAGC
	TGCTGGAGGGCGAAGAAAACCG
Iba1	AGCCGTGGTGATAGGCACCC
	TCCAGCCCTGCTCTTTGCCC
Itgam	AATTGGGGTGGGAAATGCCT
	TAGATGCGATGGTGTCGAGC
Cd4	ATTAGAGGAGGTTCGCCTTCG
	GATCCTTTCTCCCATGCCCC
Lat	AAGACGACTATCCCAACGGC
	CACACGACTCCACAGAGAAGG
Aqp4	ATGGTGGATCCCACACCGAG
	AGGCGGTGGGGTAAGTGTG

Supplementary Table 2. Differentially expressed transcripts in vascular fragments between EAE and naive mice at the preclinical phase

Gene symbol	Entrez gene name	Affymetrix ID	Fold Change	Adj. *P* value
Ackr1	atypical chemokine receptor 1 (Duffy blood group)	17229984	3.190	1.68E-04
Aplnr	apelin receptor	17372725	-1.806	3.23E-03
Atf3	activating transcription factor 3	17231033	1.429	1.68E-04
Bcl6B	B-cell CLL/lymphoma 6, member B	17265164	-1.334	1.90E-02
Ccl3L3	chemokine (C-C motif) ligand 3-like 3	17266967	1.139	2.21E-02
Ccl4	chemokine (C-C motif) ligand 4	17254283	1.288	2.17E-02
Cd14	CD14 molecule	17353747	0.995	3.25E-02
Cdkn1A	cyclin-dependent kinase inhibitor 1A (p21, Cip1)	17335467	1.167	3.79E-04
Ch25H	cholesterol 25-hydroxylase	17364111	1.940	2.89E-04
Cyp2E1	cytochrome P450, family 2, subfamily E, polypeptide 1	17484587	-1.122	1.83E-02
D730005E14Ril	RIKEN cDNA D730005E14 gene	17319364	1.245	2.89E-04
Dcbld2	discoidin, CUB and LCCL domain containing 2	17326405	1.015	1.90E-02
Doc2B	double C2-like domains, beta	17266010	2.420	3.96E-04
Fam107A	family with sequence similarity 107, member A	17303433	1.087	3.25E-02
Fkbp5	FK506 binding protein 5	17342868	1.545	3.79E-04
Fnbp1L	formin binding protein 1-like	17409963	1.005	3.25E-02
Gadd45B	growth arrest and DNA-damage-inducible, beta	17235511	1.315	8.20E-03
Gadd45G	growth arrest and DNA-damage-inducible, gamma	17287361	1.507	1.17E-03
Galnt15	polypeptide N-acetylgalactosaminyltransferase 15	17298473	1.308	3.74E-02
Gm14023	predicted gene 14023	17376153	1.029	3.25E-02
Gm5	predicted gene 5	17455542	1.138	4.92E-02
Gm6614	predicted gene 6614	17472406	1.719	4.92E-02
Gsdmc	gasdermin C	17317472	-1.162	1.28E-02
Hif3A	hypoxia inducible factor 3, alpha subunit	17487001	1.018	3.84E-02
Icam1	intercellular adhesion molecule 1	17515074	1.395	7.62E-06
Ier3	immediate early response 3	17337228	1.047	3.93E-02
Ighm	immunoglobulin heavy constant mu	17284605	-1.415	2.83E-02
Il1B	interleukin 1, beta	17391565	1.915	2.17E-02
Il1R1	interleukin 1 receptor, type I	17212185	1.526	2.24E-03
Inpp5D	inositol polyphosphate-5-phosphatase D	17215309	1.266	1.34E-02
Jade2	jade family PHD finger 2	17262551	1.201	1.68E-04
Lao1	L-amino acid oxidase 1	17417826	1.422	3.53E-03
Lcn2	lipocalin 2	17383892	3.586	1.38E-05
Lrg1	leucine-rich alpha-2-glycoprotein 1	17346150	1.944	3.18E-03
Map3K6	mitogen-activated protein kinase kinase kinase 6	17419553	1.566	3.23E-03
Mir1945	microRNA 1945	17328220	-1.012	3.07E-02
Ndnf	neuron-derived neurotrophic factor	17459207	-1.232	2.36E-02
Nfkbiz	nuclear factor of kappa light polypeptide gene enhancer in B-cells inhibitor, zeta	17330967	1.173	1.42E-04
Pla1A	phospholipase A1 member A	17330359	1.188	1.52E-02
Plxna2	plexin A2	17220974	1.808	2.24E-03
Ppp1R15A	protein phosphatase 1, regulatory subunit 15A	17490878	1.004	5.00E-03
Ptgs2	prostaglandin-endoperoxide synthase 2	17218060	2.038	3.23E-03
Qsox1	quiescin Q6 sulfhydryl oxidase 1	17228407	1.024	1.78E-02
Rab27A	RAB27A, member RAS oncogene family	17519282	1.083	7.33E-03
Ranbp9	RAN binding protein 9	17292122	1.336	5.29E-03
Scgb3A1	secretoglobin, family 3A, member 1	17249028	1.903	5.02E-02
Serpina3	serpin peptidase inhibitor, clade A (alpha-1 antiproteinase, antitrypsin), member 3	17278328	4.219	5.91E-04
Serpina3G	serine (or cysteine) peptidase inhibitor, clade A, member 3G	17278268	2.319	4.89E-03
Serpini1	serpin peptidase inhibitor, clade I (neuroserpin), member 1	17398323	1.126	4.37E-02
Slc16A1	solute carrier family 16 (monocarboxylate transporter), member 1	17401335	-1.034	4.77E-02
Slc6A20	solute carrier family 6 (proline IMINO transporter), member 20	17532509	1.201	6.45E-03
Snora31	small nucleolar RNA, H/ACA box 31	17302054	1.059	1.78E-02
Snord49B	small nucleolar RNA, C/D box 49B	17250740	1.208	3.25E-02
Sult1A1	sulfotransferase family 1A, phenol-preferring, member 1	17496354	1.345	1.69E-03
Tbc1D4	TBC1 domain family, member 4	17309118	1.199	1.28E-02
Tecrl	trans-2,3-enoyl-CoA reductase-like	17449108	1.506	3.05E-02
Tnfa	tumor necrosis factor alpha	17344309	1.649	2.04E-03
Xdh	xanthine dehydrogenase	17347163	1.260	2.27E-03
Zbtb16	zinc finger and BTB domain containing 16	17526707	2.246	1.21E-03

Supplementary Table 3. Differentially expressed transcripts in vascular fragments between EAE and naive mice at the progression phase

Gene symbol	Gene name	Affymetrix ID	Fold Change	Adj. *P* value
Aarsd1	alanyl-tRNA synthetase domain containing 1	17269903	-1.028	5.11E-03
Aatf	apoptosis antagonizing transcription factor	17267021	1.004	2.96E-04
Abca8A	ATP-binding cassette, sub-family A (ABC1), member 8a	17271350	-1.252	9.06E-04
Abca9	ATP-binding cassette, sub-family A (ABC1), member 9	17271399	-1.616	2.72E-06
Abcb1B	ATP-binding cassette, sub-family B (MDR/TAP), member 1B	17434555	1.407	1.16E-04
Abcc6	ATP-binding cassette, sub-family C (CFTR/MRP), member 6	17491035	-1.538	1.64E-07
Abcg1	ATP-binding cassette, sub-family G (WHITE), member 1	17335770	1.087	5.87E-03
Abi3Bp	ABI gene family, member 3 (NESH) binding protein	17326318	-1.131	4.28E-04
Ablim3	actin binding LIM protein family, member 3	17354831	-1.084	1.84E-03
Abracl	ABRA C-terminal like	17548593	1.541	2.72E-05
Acat2	acetyl-CoA acetyltransferase 2	17340953	1.274	6.76E-04
Ace	angiotensin I converting enzyme	17257444	1.084	5.49E-04
Acer3	alkaline ceramidase 3	17493556	1.183	3.14E-04
Ackr1	atypical chemokine receptor 1 (Duffy blood group)	17229984	3.840	1.73E-07
Ackr3	atypical chemokine receptor 3	17215605	-1.021	1.11E-03
Acp5	acid phosphatase 5, tartrate resistant	17524930	2.128	1.20E-03
Acsl4	acyl-CoA synthetase long-chain family member 4	17545051	1.129	1.95E-05
Actr3	ARP3 actin-related protein 3 homolog (yeast)	17226420	1.040	5.53E-07
Acvr1B	activin A receptor, type IB	17315152	1.698	1.30E-04
Acy3	aminoacylase 3	17356041	-1.009	1.00E-04
Adam19	ADAM metallopeptidase domain 19	17248754	1.221	2.40E-03
Adam8	ADAM metallopeptidase domain 8	17497525	2.484	1.12E-05
Adam9	ADAM metallopeptidase domain 9	17508300	1.346	2.31E-07
Adamts4	ADAM metallopeptidase with thrombospondin type 1 motif, 4	17219248	1.512	2.10E-05
Adamts9	ADAM metallopeptidase with thrombospondin type 1 motif, 9	17469136	2.650	3.07E-07
Adap1	ArfGAP with dual PH domains 1	17454382	1.495	9.55E-04
Adgra2	adhesion G protein-coupled receptor A2	17500301	-1.110	1.54E-04
Adgrg6	adhesion G protein-coupled receptor G6	17239435	-1.389	2.81E-03
Adh1C	alcohol dehydrogenase 1C (class I), gamma polypeptide	17403070	-2.490	1.73E-06
Adora2B	adenosine A2b receptor	17250660	1.030	1.69E-04
Adra2A	adrenoceptor alpha 2A	17360440	-1.133	7.73E-04
Adrb2	adrenoceptor beta 2, surface	17354857	-1.280	7.76E-07
Adssl1	adenylosuccinate synthase like 1	17279349	2.463	4.37E-05
Afap1L1	actin filament associated protein 1-like 1	17354810	-1.521	1.35E-05
Agmo	alkylglycerol monooxygenase	17275069	-1.417	2.47E-06
Agrn	agrin	17433977	-1.117	3.76E-05
Ai480526	expressed sequence AI480526	17452562	-1.154	1.07E-04
Ai504432	expressed sequence AI504432	17401530	3.264	1.02E-06
Ai506816	expressed sequence AI506816	17446060	1.308	1.05E-02
Ai662270	expressed sequence AI662270	17254194	2.079	9.98E-06
Ai839979	expressed sequence AI839979	17447013	1.719	2.70E-05
Aif1L	allograft inflammatory factor 1-like	17369613	-1.257	6.06E-05
Aim1	absent in melanoma 1	17240621	1.192	2.79E-04
Ak2	adenylate kinase 2	17418904	1.223	5.61E-04
Akr1B7	aldo-keto reductase family 1, member B7	17457158	-1.037	8.52E-03
Akr1C14	aldo-keto reductase family 1, member C14	17284919	-1.279	2.17E-04
Aldh1A1	aldehyde dehydrogenase 1 family, member A1	17358103	-1.146	5.99E-03
Alg8	ALG8, alpha-1,3-glucosyltransferase	17480327	1.650	5.24E-06
Alox12	arachidonate 12-lipoxygenase	17265193	-1.675	3.66E-06
Alox5Ap	arachidonate 5-lipoxygenase-activating protein	17444961	1.619	2.80E-08
Alpl	alkaline phosphatase, liver/bone/kidney	17431720	-1.144	1.71E-04
Amica1	adhesion molecule, interacts with CXADR antigen 1	17516718	2.172	2.72E-06
Angpt2	angiopoietin 2	17507799	1.557	1.47E-04
Ankh	ANKH inorganic pyrophosphate transport regulator	17310673	-1.094	1.62E-05
Anks1A	ankyrin repeat and sterile alpha motif domain containing 1A	17335204	-1.080	1.40E-05
Anln	anillin actin binding protein	17524969	1.485	2.37E-05
Anpep	alanyl (membrane) aminopeptidase	17492431	2.272	5.55E-04
Antxr1	anthrax toxin receptor 1	17468573	-1.644	6.13E-06
Antxr2	anthrax toxin receptor 2	17449939	1.324	1.26E-03
Anxa1	annexin A1	17363407	1.643	5.83E-05
Anxa2	annexin A2	17518962	1.304	2.94E-07
Anxa4	annexin A4	17468551	1.107	7.01E-05
Anxa6	annexin A6	17262887	1.373	2.02E-06
Aoah	acyloxyacyl hydrolase (neutrophil)	17285586	2.673	1.11E-04
Apaf1	apoptotic peptidase activating factor 1	17244140	1.172	7.62E-05
Apcdd1	adenomatosis polyposis coli down-regulated 1	17351196	-1.130	1.59E-06
Apln	apelin	17541378	-1.700	5.22E-05
Aplnr	apelin receptor	17372725	-1.635	2.47E-04
Apobec1	apolipoprotein B mRNA editing enzyme, catalytic polypeptide 1	17470580	1.457	1.17E-03
Apobec3B	apolipoprotein B mRNA editing enzyme, catalytic polypeptide-like 3B	17313050	1.000	1.06E-05
Apoc2	apolipoprotein C-II	17487361	2.590	2.70E-04
Apol7E (Includ	apolipoprotein L 7e	17318794	-1.275	5.53E-04
Aprt	adenine phosphoribosyltransferase	17513771	1.661	7.84E-07
Aqp11	aquaporin 11	17493461	-1.619	9.56E-07
Arg1	arginase 1	17239845	4.862	2.11E-04
Arg2	arginase 2	17276776	2.015	1.03E-04
Arhgap15	Rho GTPase activating protein 15	17370551	1.534	9.17E-04
Arhgap29	Rho GTPase activating protein 29	17402193	-1.058	2.20E-03
Arhgap30	Rho GTPase activating protein 30	17219324	1.017	6.09E-03
Arhgap9	Rho GTPase activating protein 9	17237984	1.038	2.86E-03
Arhgdib	Rho GDP dissociation inhibitor (GDI) beta	17472210	1.408	1.32E-05

Supplementary Table 3. Differentially expressed transcripts in vascular fragments between EAE and naive mice at the progression phase

Gene symbol	Gene name	Affymetrix ID	Fold Change	Adj. *P* value
Arhgef10	Rho guanine nucleotide exchange factor (GEF) 10	17499447	-1.060	1.27E-03
Arhgef12	Rho guanine nucleotide exchange factor (GEF) 12	17526038	-1.017	4.62E-04
Arhgef9	Cdc42 guanine nucleotide exchange factor (GEF) 9	17543290	-1.286	5.84E-04
Arntl	aryl hydrocarbon receptor nuclear translocator-like	17481960	2.057	3.39E-09
Arrdc4	arrestin domain containing 4	17492051	1.602	7.34E-05
Arxes1/Arxes2	adipocyte-related X-chromosome expressed sequence 2	17537861	-1.012	5.15E-03
Asf1B	anti-silencing function 1B histone chaperone	17503023	1.628	1.14E-06
Aspn	asporin	17287160	-1.385	1.28E-03
Asprv1	aspartic peptidase, retroviral-like 1	17460465	1.660	4.74E-04
Atad2	ATPase family, AAA domain containing 2	17317233	1.440	4.80E-06
Atf1	activating transcription factor 1	17548846	1.198	1.60E-04
Atf3	activating transcription factor 3	17231033	1.676	2.77E-07
Atic	5-aminoimidazole-4-carboxamide ribonucleotide formyltransferase/IMP cyclohydrolase	17213990	1.214	1.18E-04
Atp10A	ATPase, class V, type 10A	17478745	-1.053	8.95E-04
Atp1A3	ATPase, Na+/K+ transporting, alpha 3 polypeptide	17487805	2.406	1.73E-05
Atp2A3	ATPase, Ca++ transporting, ubiquitous	17252497	-1.088	4.74E-04
Atp6Ap2	ATPase, H+ transporting, lysosomal accessory protein 2	17533336	1.022	1.57E-04
Atp6V0C	ATPase, H+ transporting, lysosomal 16kDa, V0 subunit c	17457343	1.649	6.05E-05
Atp6V0D2	ATPase, H+ transporting, lysosomal 38kDa, V0 subunit d2	17423577	1.874	3.68E-02
Atp6V1B2	ATPase, H+ transporting, lysosomal 56/58kDa, V1 subunit B2	17501652	1.216	1.73E-05
Atp7A	ATPase, Cu++ transporting, alpha polypeptide	17537055	1.110	5.88E-05
Atp8B1	ATPase, aminophospholipid transporter, class I, type 8B, member 1	17355026	1.724	3.03E-04
Atp8B4	ATPase, class I, type 8B, member 4	17391056	2.888	6.19E-06
Au021063	expressed sequence AU021063	17315163	-1.185	3.84E-03
Aurka	aurora kinase A	17395016	1.174	6.32E-04
Aurkb	aurora kinase B	17251485	1.385	7.99E-05
Aw549542	expressed sequence AW549542	17451930	-1.095	1.41E-02
Axin2	axin 2	17257801	-1.550	4.45E-06
B230206H07Ri	RIKEN cDNA B230206H07 gene	17497877	-1.346	2.40E-04
B3Gnt3	UDP-GlcNAc:betaGal beta-1,3-N-acetylglucosaminyltransferase 3	17510422	1.127	2.82E-04
B430306N03Ri	RIKEN cDNA B430306N03 gene	17338403	2.052	5.84E-06
B4Galnt1	beta-1,4-N-acetyl-galactosaminyl transferase 1	17237937	1.887	1.59E-06
B4Galt1	UDP-Gal:betaGlcNAc beta 1,4- galactosyltransferase, polypeptide 1	17424077	1.357	8.20E-06
B4Galt4	UDP-Gal:betaGlcNAc beta 1,4- galactosyltransferase, polypeptide 4	17325637	-1.064	2.00E-03
Bak1	BCL2-antagonist/killer 1	17342676	1.276	3.89E-05
Batf	basic leucine zipper transcription factor, ATF-like	17277404	1.416	4.89E-06
Batf2	basic leucine zipper transcription factor, ATF-like 2	17356662	1.500	7.89E-05
Baz1A	bromodomain adjacent to zinc finger domain, 1A	17281148	1.479	7.37E-08
Bc147527	cDNA sequence BC147527	17290205	1.242	3.56E-04
Bcam	basal cell adhesion molecule (Lutheran blood group)	17487422	-1.148	3.67E-05
Bcap31	B-cell receptor-associated protein 31	17542382	1.666	1.89E-05
Bche	butyrylcholinesterase	17405908	-2.131	1.84E-07
Bckdha	branched chain keto acid dehydrogenase E1, alpha polypeptide	17487982	-1.043	1.75E-03
Bcl2A1	BCL2-related protein A1	17520162	2.629	2.40E-07
Bcl3	B-cell CLL/lymphoma 3	17487457	2.082	5.08E-08
Bcl6	B-cell CLL/lymphoma 6	17329433	1.475	8.20E-06
Bcl6B	B-cell CLL/lymphoma 6, member B	17265164	-1.209	1.61E-03
Be692007	expressed sequence BE692007	17362966	1.276	4.68E-03
Best1	bestrophin 1	17362579	1.519	1.08E-05
Bin2	bridging integrator 2	17321768	1.354	1.99E-04
Birc5	baculoviral IAP repeat containing 5	17258867	2.312	3.66E-06
Bloc1S2	biogenesis of lysosomal organelles complex-1, subunit 2	17365072	1.069	6.29E-03
Bmp2K	BMP2 inducible kinase	17439481	1.288	9.86E-06
Bora	bora, aurora kinase A activator	17302362	1.021	1.11E-04
Brca1	breast cancer 1, early onset	17269921	1.046	2.92E-04
Brcc3	BRCA1/BRCA2-containing complex, subunit 3	17535957	1.092	8.94E-04
Bri3Bp	BRI3 binding protein	17442714	1.311	1.62E-04
Brip1	BRCA1 interacting protein C-terminal helicase 1	17267209	1.141	2.68E-03
Brix1	BRX1, biogenesis of ribosomes	17315960	1.481	8.81E-06
Bst1	bone marrow stromal cell antigen 1	17437198	3.164	2.52E-07
Bst2	bone marrow stromal cell antigen 2	17510345	1.183	3.76E-05
Btbd3	BTB (POZ) domain containing 3	17376867	-1.076	8.17E-06
Btg1	B-cell translocation gene 1, anti-proliferative	17236787	1.042	1.60E-04
Bub1	BUB1 mitotic checkpoint serine/threonine kinase	17391376	1.551	4.24E-05
Bub1B	BUB1 mitotic checkpoint serine/threonine kinase B	17374569	1.782	4.24E-05
C130026I21Rik	RIKEN cDNA C130026I21 gene	17366201	2.370	2.28E-06
C15Orf48	chromosome 15 open reading frame 48	17375503	6.492	5.12E-07
C15Orf61	chromosome 15 open reading frame 61	17528079	-1.427	1.33E-04
C19Orf12	chromosome 19 open reading frame 12	17476872	1.140	2.11E-04
C19Orf38	chromosome 19 open reading frame 38	17515238	2.738	3.67E-06
C19Orf70	chromosome 19 open reading frame 70	17346311	1.223	9.49E-04
C1Orf115	chromosome 1 open reading frame 115	17230754	-1.232	8.32E-04
C1Orf131	chromosome 1 open reading frame 131	17514122	1.058	1.10E-05
C1Orf233	chromosome 1 open reading frame 233	17422659	-1.287	1.01E-05
C1S	complement component 1, s subcomponent	17462889	1.869	1.45E-03
C3	complement component 3	17346528	4.652	9.15E-08
C3Ar1	complement component 3a receptor 1	17470616	1.380	1.26E-03
C4A/C4B	complement component 4B (Chido blood group)	17343918	2.332	4.27E-05
C5Ar1	complement component 5a receptor 1	17486864	2.726	1.35E-09
C7Orf50	chromosome 7 open reading frame 50	17454408	1.008	1.26E-02
C8Orf76	chromosome 8 open reading frame 76	17317214	1.054	1.34E-03

Supplementary Table 3. Differentially expressed transcripts in vascular fragments between EAE and naive mice at the progression phase

Gene symbol	Gene name	Affymetrix ID	Fold Change	Adj. *P* value
C920009B18Ri	RIKEN cDNA C920009B18 gene	17232112	1.726	7.65E-05
C9Orf172	chromosome 9 open reading frame 172	17382681	-1.159	2.53E-05
C9Orf91	chromosome 9 open reading frame 91	17414767	1.181	6.32E-04
Ca11	carbonic anhydrase XI	17477968	-1.107	1.13E-04
Ca13	carbonic anhydrase XIII	17396143	1.873	1.04E-06
Ca14	carbonic anhydrase XIV	17407956	-1.209	1.95E-04
Ca4	carbonic anhydrase IV	17254508	-1.380	1.19E-04
Cadm1	cell adhesion molecule 1	17516960	-1.115	8.52E-06
Calr	calreticulin	17511183	1.035	5.73E-07
Camk1D	calcium/calmodulin-dependent protein kinase ID	17381357	1.113	9.03E-04
Camk2N1	calcium/calmodulin-dependent protein kinase II inhibitor 1	17420514	-1.071	2.16E-04
Capg	capping protein (actin filament), gelsolin-like	17459656	1.226	3.93E-06
Card10	caspase recruitment domain family, member 10	17319063	-1.003	7.43E-04
Casc5	cancer susceptibility candidate 5	17374692	2.309	3.99E-07
Casp1	caspase 1, apoptosis-related cysteine peptidase	17514424	2.251	3.23E-07
Casp4	caspase 4, apoptosis-related cysteine peptidase	17514435	2.371	3.82E-08
Casp8Ap2	caspase 8 associated protein 2	17412404	1.194	4.24E-05
Cav1	caveolin 1, caveolae protein, 22kDa	17456161	-1.174	7.59E-05
Cav2	caveolin 2	17456152	-1.274	8.04E-06
Cbfa2T3	core-binding factor, runt domain, alpha subunit 2; translocated to, 3	17513806	-1.230	5.43E-06
Ccdc109B	coiled-coil domain containing 109B	17410332	1.389	1.39E-04
Ccdc25	coiled-coil domain containing 25	17301440	1.029	4.63E-04
Ccdc86	coiled-coil domain containing 86	17362831	1.742	3.72E-05
Ccl11	chemokine (C-C motif) ligand 11	17254053	1.606	1.39E-04
Ccl17	chemokine (C-C motif) ligand 17	17504138	3.488	7.21E-04
Ccl2	chemokine (C-C motif) ligand 2	17254041	5.616	1.06E-09
Ccl22	chemokine (C-C motif) ligand 22	17504122	2.813	1.28E-03
Ccl24	chemokine (C-C motif) ligand 24	17453611	1.490	6.03E-03
Ccl3L3	chemokine (C-C motif) ligand 3-like 3	17266967	2.217	1.27E-07
Ccl4	chemokine (C-C motif) ligand 4	17254283	2.362	2.85E-07
Ccl5	chemokine (C-C motif) ligand 5	17266946	3.661	2.37E-05
Ccl6	chemokine (C-C motif) ligand 6	17266960	2.839	3.48E-06
Ccl7	chemokine (C-C motif) ligand 7	17254047	4.592	3.11E-06
Ccl8	chemokine (C-C motif) ligand 8	17254065	1.357	1.23E-02
Ccl9	chemokine (C-C motif) ligand 9	17266952	1.528	1.71E-04
Ccna2	cyclin A2	17404821	2.080	7.13E-07
Ccnb1	cyclin B1	17295757	1.072	7.16E-04
Ccnb2	cyclin B2	17528586	2.167	3.93E-06
Ccr1	chemokine (C-C motif) receptor 1	17532569	3.156	2.72E-06
Ccr2	chemokine (C-C motif) receptor 2	17523650	3.532	3.69E-07
Ccr5	chemokine (C-C motif) receptor 5 (gene/pseudogene)	17523659	1.057	5.15E-04
Ccr7	chemokine (C-C motif) receptor 7	17268972	1.021	5.28E-03
Ccrl2	chemokine (C-C motif) receptor-like 2	17531705	1.860	6.88E-06
Cct6A	chaperonin containing TCP1, subunit 6A (zeta 1)	17442925	1.113	4.18E-05
Cct8	chaperonin containing TCP1, subunit 8 (theta)	17331774	1.087	4.81E-08
Cd14	CD14 molecule	17353747	2.191	4.42E-08
Cd2	CD2 molecule	17408497	2.262	5.00E-04
Cd200R1L	CD200 receptor 1-like	17325874	1.724	2.62E-03
Cd226	CD226 molecule	17352330	1.133	6.24E-03
Cd244	CD244 molecule, natural killer cell receptor 2B4	17219382	2.674	4.63E-07
Cd248	CD248 molecule, endosialin	17356369	-1.017	1.30E-03
Cd274	CD274 molecule	17358544	3.043	1.84E-05
Cd28	CD28 molecule	17213462	1.352	6.11E-03
Cd300Ld	CD300 molecule-like family member d	17271724	1.746	8.92E-05
Cd300Lf	CD300 molecule-like family member f	17271776	3.691	2.26E-06
Cd33	CD33 antigen	17490149	1.194	2.63E-03
Cd36	CD36 molecule (thrombospondin receptor)	17445715	2.798	5.75E-04
Cd38	CD38 molecule	17437213	1.238	5.05E-04
Cd3D	CD3d molecule, delta (CD3-TCR complex)	17516691	1.906	1.97E-03
Cd3E	CD3e molecule, epsilon (CD3-TCR complex)	17526464	2.634	7.60E-05
Cd3G	CD3g molecule, gamma (CD3-TCR complex)	17526456	2.761	5.53E-04
Cd4	CD4 molecule	17470960	1.256	6.62E-03
Cd40Lg	CD40 ligand	17535048	1.282	1.32E-02
Cd44	CD44 molecule (Indian blood group)	17388733	2.737	2.45E-07
Cd48	CD48 molecule	17219397	1.682	1.14E-05
Cd5	CD5 molecule	17362753	1.440	3.38E-03
Cd52	CD52 antigen	17431174	1.587	5.11E-06
Cd53	CD53 molecule	17408960	1.812	3.94E-07
Cd68	CD68 molecule	17264835	1.249	3.75E-05
Cd69	CD69 molecule	17471502	3.424	4.06E-07
Cd72	CD72 molecule	17424608	1.689	9.34E-04
Cd74	CD74 molecule, major histocompatibility complex, class II invariant chain	17350982	2.475	2.10E-06
Cd79A	CD79a molecule, immunoglobulin-associated alpha	17475127	-1.218	7.96E-03
Cd80	CD80 molecule	17325608	1.187	2.26E-03
Cd86	CD86 molecule	17330203	1.912	2.02E-06
Cdc20	cell division cycle 20	17429177	1.022	9.66E-04
Cdc42Ep1	CDC42 effector protein (Rho GTPase binding) 1	17312774	-1.149	2.97E-03
Cdc45	cell division cycle 45	17328978	1.068	4.95E-04
Cdca5	cell division cycle associated 5	17356622	1.226	1.40E-04
Cdca8	cell division cycle associated 8	17429896	1.072	1.84E-04
Cdk1	cyclin-dependent kinase 1	17241692	2.921	7.13E-07

Supplementary Table 3. Differentially expressed transcripts in vascular fragments between EAE and naive mice at the progression phase

Gene symbol	Gene name	Affymetrix ID	Fold Change	Adj. *P* value
Cdk6	cyclin-dependent kinase 6	17434297	1.231	4.17E-04
Cdkn1A	cyclin-dependent kinase inhibitor 1A (p21, Cip1)	17335467	2.010	2.21E-09
Cdkn2B	cyclin-dependent kinase inhibitor 2B (p15, inhibits CDK4)	17427155	-1.267	1.23E-05
Cdkn3	cyclin-dependent kinase inhibitor 3	17299268	1.463	8.07E-04
Cdt1	chromatin licensing and DNA replication factor 1	17506418	1.375	3.11E-05
Cebpb	CCAAT/enhancer binding protein (C/EBP), beta	17379938	1.739	7.61E-08
Cebpd	CCAAT/enhancer binding protein (C/EBP), delta	17323297	1.193	1.26E-03
Cenpa	centromere protein A	17435825	1.175	2.71E-04
Cenpe	centromere protein E, 312kDa	17402797	1.809	1.82E-06
Cenpf	centromere protein F, 350/400kDa	17230918	1.090	4.67E-04
Cenph	centromere protein H	17295745	1.585	9.57E-06
Cenpk	centromere protein K	17289584	1.770	4.02E-05
Cenpw	centromere protein W	17240022	1.444	2.92E-04
Cep55	centrosomal protein 55kDa	17359098	1.596	2.34E-05
Cept1	choline/ethanolamine phosphotransferase 1	17408945	1.067	8.39E-06
Cers4	ceramide synthase 4	17498847	-1.281	6.34E-06
Cers6	ceramide synthase 6	17371374	2.691	4.82E-06
Ces2G	carboxylesterase 2G	17504572	-1.131	8.52E-03
Cfb	complement factor B	17344064	3.958	2.50E-08
Cfp	complement factor properdin	17540521	1.512	1.08E-05
Ch25H	cholesterol 25-hydroxylase	17364111	2.734	3.82E-08
Chaf1A	chromatin assembly factor 1, subunit A (p150)	17338701	1.001	1.05E-04
Chaf1B	chromatin assembly factor 1, subunit B (p60)	17327331	1.041	4.76E-05
Chd7	chromodomain helicase DNA binding protein 7	17411647	1.193	1.14E-05
Chi3L1	chitinase 3-like 1 (cartilage glycoprotein-39)	17217399	1.201	4.30E-03
Chil3/Chil4	chitinase-like 3	17408897	5.525	1.22E-06
Chst15	carbohydrate (N-acetylgalactosamine 4-sulfate 6-O) sulfotransferase 15	17497076	-1.094	4.74E-04
Ciart	circadian associated repressor of transcription	17407934	-2.619	8.21E-09
Cish	cytokine inducible SH2-containing protein	17521300	1.224	2.21E-03
Ckap2	cytoskeleton associated protein 2	17508025	1.319	1.75E-04
Ckap2L	cytoskeleton associated protein 2-like	17391544	1.304	6.51E-05
Cks2	CDC28 protein kinase regulatory subunit 2	17548955	1.379	2.98E-02
Clcn7	chloride channel, voltage-sensitive 7	17334545	1.323	6.40E-04
Cldn24	claudin 24	17501096	-1.149	1.49E-04
Clec12A	C-type lectin domain family 12, member A	17463509	2.997	1.63E-04
Clec14A	C-type lectin domain family 14, member A	17281350	-1.472	7.25E-06
Clec4A3	C-type lectin domain family 4, member a3	17462738	1.685	2.94E-04
Clec4D	C-type lectin domain family 4, member D	17462796	4.195	5.65E-06
Clec4E	C-type lectin domain family 4, member E	17470627	5.530	2.21E-06
Clec5A	C-type lectin domain family 5, member A	17466228	2.598	2.96E-07
Clec6A	C-type lectin domain family 6, member A	17462788	4.298	5.73E-06
Clec7A	C-type lectin domain family 7, member A	17471541	4.032	3.56E-07
Clec9A	C-type lectin domain family 9, member A	17463530	1.168	3.28E-02
Clic5	chloride intracellular channel 5	17337852	-1.377	4.34E-06
Cln5	ceroid-lipofuscinosis, neuronal 5	17302483	1.004	1.96E-04
Clstn1	calsyntenin 1	17421828	-1.033	7.84E-04
Cmip	c-Maf inducing protein	17505967	1.060	3.80E-04
Cmklr1	chemerin chemokine-like receptor 1	17451390	1.059	4.70E-04
Cml2	camello-like 2	17468444	-1.112	3.73E-03
Cmtm5	CKLF-like MARVEL transmembrane domain containing 5	17300381	-1.449	4.64E-03
Cmtm8	CKLF-like MARVEL transmembrane domain containing 8	17531932	-1.659	7.48E-07
Cndp2	CNDP dipeptidase 2 (metallopeptidase M20 family)	17355825	1.616	5.68E-06
Cntn1	contactin 1	17314387	-1.402	2.12E-03
Cobll1	cordon-bleu WH2 repeat protein-like 1	17385879	-1.568	1.97E-06
Col14A1	collagen, type XIV, alpha 1	17311551	-1.117	4.30E-04
Col4A3Bp	collagen, type IV, alpha 3 (Goodpasture antigen) binding protein	17289304	-1.076	4.09E-06
Col6A6	collagen, type VI, alpha 6	17530503	-1.052	2.02E-03
Colca2	colorectal cancer associated 2	17526982	-1.093	1.70E-03
Colgalt2	collagen beta(1-O)galactosyltransferase 2	17218233	-1.525	5.94E-05
Coro1A	coronin, actin binding protein, 1A	17496376	1.671	3.19E-07
Coro2B	coronin, actin binding protein, 2B	17527996	-1.019	1.82E-05
Cotl1	coactosin-like F-actin binding protein 1	17513491	1.863	1.06E-05
Cox6A2	cytochrome c oxidase subunit VIa polypeptide 2	17496857	1.260	9.50E-03
Cpne8	copine VIII	17320583	1.225	1.90E-03
Creb5	cAMP responsive element binding protein 5	17458682	1.636	2.08E-04
Creld2	cysteine-rich with EGF-like domains 2	17313998	1.043	1.06E-05
Crip2	cysteine rich protein 2	17279499	-1.054	3.27E-04
Cript	cysteine-rich PDZ-binding protein	17340232	1.063	6.14E-06
Crispld1	cysteine-rich secretory protein LCCL domain containing 1	17211313	-1.668	1.22E-06
Cry2	cryptochrome circadian clock 2	17388389	-1.410	7.78E-06
Csf2Rb	colony stimulating factor 2 receptor, beta, low-affinity (granulocyte-macrophage)	17318950	4.005	6.50E-09
Csf3	colony stimulating factor 3 (granulocyte)	17256129	1.714	2.96E-04
Csmd3	CUB and Sushi multiple domains 3	17316878	-1.049	5.65E-05
Cspg4	chondroitin sulfate proteoglycan 4	17517592	-1.218	8.21E-05
Csrnp1	cysteine-serine-rich nuclear protein 1	17532257	1.261	4.18E-04
Cst7	cystatin F (leukocystatin)	17377464	2.341	5.23E-05
Cstb	cystatin B (stefin B)	17234803	1.588	1.07E-04
Cthrc1	collagen triple helix repeat containing 1	17311191	-1.719	1.30E-06
Ctla2A/Ctla2B	cytotoxic T lymphocyte-associated protein 2 alpha	17293362	1.260	1.13E-02
Ctla4	cytotoxic T-lymphocyte-associated protein 4	17213478	1.153	1.02E-02
Ctnnb1	catenin (cadherin-associated protein), beta 1, 88kDa	17523257	-1.010	2.46E-06

Supplementary Table 3. Differentially expressed transcripts in vascular fragments between EAE and naive mice at the progression phase

Gene symbol	Gene name	Affymetrix ID	Fold Change	Adj. *P* value
Ctsb	cathepsin B	17301213	1.354	2.00E-06
Ctsc	cathepsin C	17480018	2.342	2.96E-08
Ctsw	cathepsin W	17361605	1.597	3.39E-04
Ctsz	cathepsin Z	17395155	1.509	5.93E-06
Cttnbp2	cortactin binding protein 2	17465006	-1.011	3.31E-04
Cuedc1	CUE domain containing 1	17254948	-1.042	1.53E-04
Cxcl10	chemokine (C-X-C motif) ligand 10	17449718	5.210	1.51E-08
Cxcl11	chemokine (C-X-C motif) ligand 11	17449725	2.248	3.05E-04
Cxcl12	chemokine (C-X-C motif) ligand 12	17462149	-1.981	1.06E-05
Cxcl13	chemokine (C-X-C motif) ligand 13	17439367	1.056	3.39E-02
Cxcl16	chemokine (C-X-C motif) ligand 16	17265268	2.509	5.17E-07
Cxcl2	chemokine (C-X-C motif) ligand 2	17438987	1.758	1.96E-05
Cxcl3	chemokine (C-X-C motif) ligand 3	17438975	3.051	2.40E-03
Cxcl9	chemokine (C-X-C motif) ligand 9	17449710	5.292	3.71E-08
Cxcr2	chemokine (C-X-C motif) receptor 2	17214142	2.752	5.66E-05
Cxcr4	chemokine (C-X-C motif) receptor 4	17226593	1.764	2.51E-04
Cxcr6	chemokine (C-X-C motif) receptor 6	17523642	1.971	6.17E-03
Cxorf36	chromosome X open reading frame 36	17540436	-1.372	1.22E-06
Cyb5R4	cytochrome b5 reductase 4	17520043	1.400	2.10E-05
Cyba	cytochrome b-245, alpha polypeptide	17513672	1.563	3.68E-05
Cybb	cytochrome b-245, beta polypeptide	17540154	4.044	4.27E-08
Cycs	cytochrome c, somatic	17548541	1.835	5.26E-04
Cyfip2	cytoplasmic FMR1 interacting protein 2	17262065	1.140	3.83E-03
Cygb	cytoglobin	17272461	-1.107	2.94E-03
Cyp2D22	cytochrome P450, family 2, subfamily d, polypeptide 22	17319625	-1.073	1.35E-03
Cyp2E1	cytochrome P450, family 2, subfamily E, polypeptide 1	17484587	-2.330	3.25E-08
Cyp4B1	cytochrome P450, family 4, subfamily B, polypeptide 1	17428477	-1.164	5.38E-03
Cyp4F2	cytochrome P450, family 4, subfamily F, polypeptide 2	17510462	2.412	7.62E-06
Cytip	cytohesin 1 interacting protein	17385405	2.803	7.13E-07
Cytl1	cytokine-like 1	17437043	-1.191	3.06E-02
D17H6S56E-5	DNA segment, Chr 17, human D6S56E 5	17344140	1.960	9.15E-08
D4Ertd617E	DNA segment, Chr 4, ERATO Doi 617, expressed	17417808	-1.262	6.45E-04
D730005E14Ri	RIKEN cDNA D730005E14 gene	17319364	1.626	1.18E-07
Dach1	dachshund family transcription factor 1	17309041	-1.110	7.25E-05
Dapp1	dual adaptor of phosphotyrosine and 3-phosphoinositides	17410617	1.582	2.86E-06
Dazap2	DAZ associated protein 2	17315045	1.149	3.69E-04
Dbf4	DBF4 zinc finger	17445507	1.179	2.62E-04
Dbnl	drebrin-like	17247023	1.607	1.59E-05
Dbp	D site of albumin promoter (albumin D-box) binding protein	17477979	-2.823	4.32E-10
Dcaf13	DDB1 and CUL4 associated factor 13	17311199	1.376	7.79E-06
Dck	deoxycytidine kinase	17438823	1.934	2.58E-06
Dclre1C	DNA cross-link repair 1C	17366399	1.017	5.53E-04
Ddah1	dimethylarginine dimethylaminohydrolase 1	17403439	-1.027	3.63E-04
Ddc	dopa decarboxylase (aromatic L-amino acid decarboxylase)	17260644	-1.383	7.31E-05
Ddhd1	DDHD domain containing 1	17305685	1.080	4.03E-04
Ddn	dendrin	17321346	-1.017	3.16E-03
Ddost	dolichyl-diphosphooligosaccharide--protein glycosyltransferase subunit (non-catalytic)	17420486	1.064	1.45E-04
Ddt	D-dopachrome tautomerase	17241912	1.025	6.37E-04
Ddx21	DEAD (Asp-Glu-Ala-Asp) box helicase 21	17241436	1.137	9.27E-07
Ddx39A	DEAD (Asp-Glu-Ala-Asp) box polypeptide 39A	17502976	1.081	1.01E-04
Degs2	delta(4)-desaturase, sphingolipid 2	17283915	-1.705	1.26E-06
Dennd2D	DENN/MADD domain containing 2D	17401480	1.062	4.51E-04
Dennd5B	DENN/MADD domain containing 5B	17473061	-1.219	1.71E-04
Dera	deoxyribose-phosphate aldolase (putative)	17463898	1.046	3.12E-03
Dgat2	diacylglycerol O-acyltransferase 2	17493632	1.014	2.48E-03
Dhfr	dihydrofolate reductase	17289085	1.042	1.71E-04
Dhrs9	dehydrogenase/reductase (SDR family) member 9	17371427	1.151	8.79E-03
Dhx58	DEXH (Asp-Glu-X-His) box polypeptide 58	17269595	1.261	9.88E-04
Dlgap5	discs, large (Drosophila) homolog-associated protein 5	17305789	1.701	4.92E-06
Dll4	delta-like 4 (Drosophila)	17374792	-1.360	1.02E-03
Dna2	DNA replication helicase/nuclease 2	17233811	1.910	1.96E-06
Dnm3	dynamin 3	17228906	-1.020	4.04E-04
Dnph1	2'-deoxynucleoside 5'-phosphate N-hydrolase 1	17338118	1.003	5.70E-04
Doc2B	double C2-like domains, beta	17266010	1.298	6.56E-03
Dock11	dedicator of cytokinesis 11	17533994	1.206	1.24E-04
Dock2	dedicator of cytokinesis 2	17261650	1.272	1.38E-04
Dock6	dedicator of cytokinesis 6	17524775	-1.198	2.85E-04
Dpep2	dipeptidase 2	17512628	2.136	1.66E-03
Dpp4	dipeptidyl-peptidase 4	17385719	-1.054	1.62E-03
Dram1	DNA-damage regulated autophagy modulator 1	17243868	1.803	2.24E-04
Dtx3L	deltex 3 like, E3 ubiquitin ligase	17330119	1.099	3.02E-04
Dusp10	dual specificity phosphatase 10	17220475	1.379	2.63E-05
Dusp2	dual specificity phosphatase 2	17375859	1.129	6.49E-05
Dyrk1B	dual-specificity tyrosine-(Y)-phosphorylation regulated kinase 1B	17475787	-1.233	3.02E-07
E030003E18Ri	RIKEN cDNA E030003E18 gene	17358098	-1.189	2.60E-03
E230013L22Ri	RIKEN cDNA E230013L22 gene	17499011	1.094	1.13E-02
E2F8	E2F transcription factor 8	17491378	1.949	1.51E-07
Ear2 (Includes	eosinophil-associated, ribonuclease A family, member 2	17305520	-2.692	1.92E-02
Ebf1	early B-cell factor 1	17248691	-1.093	1.18E-03
Ecm1	extracellular matrix protein 1	17407850	1.172	7.00E-06
Ecm2	extracellular matrix protein 2, female organ and adipocyte specific	17287148	-1.482	3.72E-04

Supplementary Table 3. Differentially expressed transcripts in vascular fragments between EAE and naive mice at the progression phase

Gene symbol	Gene name	Affymetrix ID	Fold Change	Adj. *P* value
Edn3	endothelin 3	17380377	-1.694	7.45E-08
Eef1E1	eukaryotic translation elongation factor 1 epsilon 1	17291964	1.720	6.39E-07
Efhd2	EF-hand domain family, member D2	17432341	1.540	1.93E-06
Efnb2	ephrin-B2	17507321	-1.490	5.18E-04
Efr3B	EFR3 homolog B	17279764	-1.397	2.99E-04
Egflam	EGF-like, fibronectin type III and laminin G domains	17315763	-1.677	9.78E-06
Egln3	egl-9 family hypoxia-inducible factor 3	17281084	2.787	1.07E-04
Egr2	early growth response 2	17233993	1.186	3.81E-04
Eid3	EP300 interacting inhibitor of differentiation 3	17235937	1.096	7.55E-04
Eif1Ad	eukaryotic translation initiation factor 1A domain containing	17356401	1.507	3.57E-04
Eif1Ax	eukaryotic translation initiation factor 1A, X-linked	17350356	1.576	1.79E-07
Eif2S1	eukaryotic translation initiation factor 2, subunit 1 alpha, 35kDa	17276732	1.260	2.30E-06
Eif4E3	eukaryotic translation initiation factor 4E family member 3	17469446	1.186	1.65E-04
Eif4Ebp1	eukaryotic translation initiation factor 4E binding protein 1	17500327	1.257	9.06E-05
Elf4	E74-like factor 4 (ets domain transcription factor)	17541404	1.921	1.51E-06
Elovl7	ELOVL fatty acid elongase 7	17289717	-1.512	3.58E-05
Emb	embigin	17290083	4.411	2.10E-07
Emilin2	elastin microfibril interfacer 2	17346975	3.041	1.61E-07
Emp1	epithelial membrane protein 1	17463781	1.434	1.08E-04
Emp2	epithelial membrane protein 2	17328062	-1.172	2.84E-03
Enah	enabled homolog (Drosophila)	17230573	-1.055	4.91E-04
Enho	energy homeostasis associated	17424276	-1.005	1.32E-04
Enpp2	ectonucleotide pyrophosphatase/phosphodiesterase 2	17317056	-1.397	7.50E-03
Enpp5	ectonucleotide pyrophosphatase/phosphodiesterase 5 (putative)	17337844	-1.006	4.10E-04
Eogt	EGF domain-specific O-linked N-acetylglucosamine (GlcNAc) transferase	17469289	-1.139	5.31E-05
Ephx1	epoxide hydrolase 1, microsomal (xenobiotic)	17230484	-2.067	1.97E-06
Epn2	epsin 2	17263837	-1.193	1.00E-05
Epsti1	epithelial stromal interaction 1 (breast)	17302141	2.221	4.94E-06
Ermn	ermin, ERM-like protein	17385398	1.086	6.72E-03
Ero1A	endoplasmic reticulum oxidoreductase alpha	17305636	1.797	9.27E-07
Ero1B	endoplasmic reticulum oxidoreductase beta	17285204	1.267	2.74E-05
Esyt2	extended synaptotagmin-like protein 2	17279584	-1.045	2.03E-04
Etf1	eukaryotic translation termination factor 1	17353541	1.010	3.54E-06
Etv6	ets variant 6	17463673	2.029	9.15E-08
Exo1	exonuclease 1	17219848	1.096	1.31E-04
Ezh2	enhancer of zeste 2 polycomb repressive complex 2 subunit	17466507	1.201	2.88E-07
Ezr	ezrin	17340609	1.325	2.18E-03
F10	coagulation factor X	17499224	4.710	9.86E-06
F13A1	coagulation factor XIII, A1 polypeptide	17291881	2.629	8.83E-06
F3	coagulation factor III (thromboplastin, tissue factor)	17402181	1.303	4.51E-03
F630111L10Ri	RIKEN cDNA F630111L10 gene	17405469	1.100	1.11E-02
F7	coagulation factor VII (serum prothrombin conversion accelerator)	17499212	1.882	5.80E-04
F830016B08Ri	RIKEN cDNA F830016B08 gene	17350921	2.350	7.79E-05
Fabp5	fatty acid binding protein 5 (psoriasis-associated)	17548717	1.883	9.35E-04
Fam111A	family with sequence similarity 111, member A	17357815	1.354	2.68E-06
Fam155A	family with sequence similarity 155, member A	17507347	-1.102	1.15E-05
Fam193B	family with sequence similarity 193, member B	17292941	-1.010	3.47E-05
Fam20C	family with sequence similarity 20, member C	17443901	1.518	2.99E-04
Fam212A	family with sequence similarity 212, member A	17531075	-1.065	4.62E-04
Fam214A	family with sequence similarity 214, member A	17519364	-1.219	3.04E-06
Fam26F	family with sequence similarity 26, member F	17240186	2.426	5.90E-06
Fam65B	family with sequence similarity 65, member B	17285964	1.345	7.45E-05
Far2	fatty acyl CoA reductase 2	17464455	-1.036	3.12E-03
Fbl	fibrillarin	17475777	1.446	2.61E-05
Fbxl5	F-box and leucine-rich repeat protein 5	17447803	1.593	5.66E-08
Fbxo21	F-box protein 21	17441490	-1.046	3.64E-05
Fcgr1A	Fc fragment of IgG, high affinity Ia, receptor (CD64)	17408024	2.036	1.02E-06
Fcgr2A	Fc fragment of IgG, low affinity IIa, receptor (CD32)	17229620	1.463	5.24E-06
Fcgr2B	Fc fragment of IgG, low affinity IIb, receptor (CD32)	17229607	2.217	1.81E-06
Fcgr3A/Fcgr3E	Fc fragment of IgG, low affinity IIIa, receptor (CD16a)	17219199	4.756	4.32E-10
Fcgrt	Fc fragment of IgG, receptor, transporter, alpha	17490589	-1.345	3.26E-07
Fcor	Foxo1 corepressor	17507128	1.494	1.80E-02
Fendrr	Foxf1 adjacent non-coding developmental regulatory RNA	17513550	-1.499	3.57E-04
Fermt3	fermitin family member 3	17362223	1.277	1.60E-03
Fgl2	fibrinogen-like 2	17435089	2.589	5.98E-07
Fgr	FGR proto-oncogene, Src family tyrosine kinase	17419483	2.547	2.23E-05
Filip1	filamin A interacting protein 1	17529185	-1.045	1.15E-03
Fkbp5	FK506 binding protein 5	17342868	1.469	1.62E-05
Flrt2	fibronectin leucine rich transmembrane protein 2	17277788	-2.496	2.53E-10
Fmo1	flavin containing monooxygenase 1	17229020	-1.450	4.51E-05
Fmo2	flavin containing monooxygenase 2 (non-functional)	17229036	-2.667	3.98E-07
Fnbp1	formin binding protein 1	17383748	1.177	4.32E-04
Fosl2	FOS-like antigen 2	17436237	1.411	2.75E-05
Foxc1	forkhead box C1	17286340	-1.044	9.84E-05
Foxo4	forkhead box O4	17536657	-1.020	7.88E-06
Foxq1	forkhead box Q1	17286320	-1.265	1.62E-05
Fpr1	formyl peptide receptor 1	17341276	1.809	7.79E-05
Fpr2	formyl peptide receptor 2	17333731	3.620	1.06E-05
Frrs1	ferric-chelate reductase 1	17402072	1.263	2.95E-03
Fry	furry homolog (Drosophila)	17445160	-1.034	3.81E-03
Ftl	ferritin, light polypeptide	17246505	-1.171	4.95E-03

Supplementary Table 3. Differentially expressed transcripts in vascular fragments between EAE and naive mice at the progression phase

Gene symbol	Gene name	Affymetrix ID	Fold Change	Adj. *P* value
Ftsj3	FtsJ homolog 3 (E. coli)	17270783	1.255	1.50E-06
Fut2	fucosyltransferase 2 (secretor status included)	17490912	1.508	1.66E-05
Fuz	fuzzy planar cell polarity protein	17477619	-1.102	5.36E-07
Fzd4	frizzled class receptor 4	17480036	-1.026	3.64E-04
Fzd6	frizzled class receptor 6	17311179	-1.441	3.36E-05
Gabrg1	gamma-aminobutyric acid (GABA) A receptor, gamma 1	17448565	-1.090	4.33E-03
Gadd45B	growth arrest and DNA-damage-inducible, beta	17235511	1.736	5.69E-06
Gadd45G	growth arrest and DNA-damage-inducible, gamma	17287361	1.886	1.02E-06
Gal3St2	galactose-3-O-sulfotransferase 2	17216120	-1.043	9.12E-04
Galnt6	polypeptide N-acetylgalactosaminyltransferase 6	17321790	1.659	4.69E-05
Gapdh	glyceraldehyde-3-phosphate dehydrogenase	17221432	1.829	2.05E-04
Gars	glycyl-tRNA synthetase	17458771	1.057	3.11E-06
Gart	phosphoribosylglycinamide formyltransferase	17332140	1.371	3.33E-05
Gas5	growth arrest-specific 5 (non-protein coding)	17218680	1.551	1.08E-03
Gas6	growth arrest-specific 6	17507605	-1.473	6.87E-07
Gbp2	guanylate binding protein 2, interferon-inducible	17403268	2.085	3.52E-05
Gbp5	guanylate binding protein 5	17403205	1.150	3.45E-04
Gbp6	guanylate binding protein family, member 6	17450461	1.078	4.86E-02
Gbp7	guanylate binding protein 7	17403224	1.045	1.05E-03
Gbp8	guanylate-binding protein 8	17450434	2.895	8.37E-04
Gda	guanine deaminase	17363470	3.234	1.04E-07
Gdap10	ganglioside-induced differentiation-associated-protein 10	17274889	1.688	9.82E-05
Gemin6	gem (nuclear organelle) associated protein 6	17548523	1.197	2.29E-04
Gen1	GEN1 Holliday junction 5' flap endonuclease	17279962	1.176	1.34E-04
Ggct	gamma-glutamylcyclotransferase	17467031	1.590	2.29E-06
Gimap1-Gimap	GIMAP1-GIMAP5 readthrough	17466618	1.344	3.28E-03
Gins1	GINS complex subunit 1 (Psf1 homolog)	17377525	2.025	6.53E-08
Gins2	GINS complex subunit 2 (Psf2 homolog)	17513525	1.479	4.04E-06
Gip	gastric inhibitory polypeptide	17255487	-1.076	2.12E-04
Gipc3	GIPC PDZ domain containing family, member 3	17243278	-1.197	5.49E-05
Gjc3	gap junction protein, gamma 3, 30.2kDa	17454209	-1.407	2.60E-02
Gk	glycerol kinase	17543045	1.643	1.45E-06
Gkn3	gastrokine 3	17468602	-3.370	1.03E-06
Gla	galactosidase, alpha	17544517	1.421	1.16E-03
Glce	glucuronic acid epimerase	17527982	-1.007	2.61E-02
Glipr1	GLI pathogenesis-related 1	17244949	1.268	4.87E-05
Glipr2	GLI pathogenesis-related 2	17413500	2.391	4.42E-08
Glis2	GLIS family zinc finger 2	17322525	-1.134	6.33E-06
Glrx	glutaredoxin (thioltransferase)	17288716	1.567	1.07E-04
Gm10036	ribosomal protein L11 pseudogene	17348674	1.670	5.47E-04
Gm10921 (Incl	predicted gene 14374	17540902	-1.088	4.97E-03
Gm12250	predicted gene 12250	17249977	3.648	1.15E-06
Gm13150/Znf4	ZNF41, pseudogene	17421444	1.022	9.38E-03
Gm13293	predicted gene 13293	17366985	-1.095	1.31E-02
Gm13363	predicted gene 13363	17367436	1.155	3.27E-05
Gm14023	predicted gene 14023	17376153	1.625	5.83E-06
Gm15056	predicted gene 15056	17507882	2.468	2.02E-03
Gm15704	predicted gene 15704	17472756	-1.004	5.94E-05
Gm15737	predicted gene 15737	17461055	1.020	1.14E-03
Gm16046	predicted gene 16046	17341039	-1.049	3.56E-04
Gm16336	predicted gene 16336	17481797	-1.048	1.85E-05
Gm16381/Gm2	predicted gene 2001	17282920	-1.024	2.69E-02
Gm16548	predicted gene 16548	17229162	1.536	1.12E-04
Gm16894	predicted gene, 16894	17222601	1.005	5.13E-03
Gm19585	predicted gene, 19585	17338540	1.552	4.49E-03
Gm19951	predicted gene, 19951	17277876	1.715	1.14E-04
Gm21188/Gm3	predicted gene, 21188	17296558	1.828	1.84E-04
Gm3002	alpha-takusan pseudogene	17296653	-1.308	4.29E-05
Gm3258	predicted gene 3258	17240098	-1.034	6.39E-03
Gm3383 (Inclu	predicted gene 3383	17303117	-1.811	1.18E-04
Gm35034	predicted gene, 35034	17548321	1.111	5.98E-05
Gm454	predicted gene 454	17454278	-1.004	5.18E-04
Gm4841	predicted gene 4841	17354589	2.330	1.10E-05
Gm4951	predicted gene 4951	17350916	3.296	1.06E-05
Gm5069	glyceraldehyde-3-phosphate dehydrogenase pseudogene	17230451	-1.328	6.75E-04
Gm5093	predicted gene 5093	17345404	1.259	3.34E-02
Gm5150	predicted gene 5150	17404230	1.483	1.11E-03
Gm5424	argininosuccinate synthase pseudogene	17233769	1.851	4.96E-04
Gm5431	predicted gene 5431	17262209	1.610	2.40E-03
Gm5662 (Inclu	predicted gene 5662	17277679	-1.143	7.17E-03
Gm5908	predicted gene 5908	17508626	2.016	3.75E-05
Gm6548	eukaryotic translation elongation factor 1 alpha 1 pseudogene	17347353	1.220	2.70E-04
Gm6594	high mobility group nucleosomal binding domain 2 pseudogene	17548012	1.062	2.82E-03
Gm6614	predicted gene 6614	17472406	2.111	3.28E-04
Gm6625	predicted gene 6625	17548690	1.762	9.64E-03
Gm6665	glutathione S-transferase, mu 2 pseudogene	17353256	-1.033	4.95E-02
Gm7102	predicted gene 7102	17366168	-1.186	7.13E-04
Gm8203	predicted pseudogene 8203	17462057	1.166	2.43E-03
Gm9733	predicted gene 9733	17404195	2.271	1.65E-07
Gm9946	predicted gene 9946	17462162	-1.826	1.53E-04
Gmnn	geminin, DNA replication inhibitor	17291343	1.399	3.31E-04

Supplementary Table 3. Differentially expressed transcripts in vascular fragments between EAE and naive mice at the progression phase

Gene symbol	Gene name	Affymetrix ID	Fold Change	Adj. *P* value
Gna13	guanine nucleotide binding protein (G protein), alpha 13	17257822	1.330	5.90E-06
Gng7	guanine nucleotide binding protein (G protein), gamma 7	17243147	-1.177	1.88E-04
Gnl3	guanine nucleotide binding protein-like 3 (nucleolar)	17304493	1.081	1.03E-06
Gnptab	N-acetylglucosamine-1-phosphate transferase, alpha and beta subunits	17236339	1.173	5.89E-04
Golt1B	golgi transport 1B	17464128	1.221	1.11E-07
Got1	glutamic-oxaloacetic transaminase 1, soluble	17364932	1.280	2.17E-04
Got2	glutamic-oxaloacetic transaminase 2, mitochondrial	17512103	1.154	1.20E-04
Gpat3	glycerol-3-phosphate acyltransferase 3	17439622	1.177	2.59E-03
Gpatch4	G patch domain containing 4	17398915	1.029	6.42E-05
Gpm6A	glycoprotein M6A	17501191	-1.195	1.29E-02
Gpnmb	glycoprotein (transmembrane) nmb	17458439	3.932	1.50E-04
Gpr132	G protein-coupled receptor 132	17284247	1.751	1.84E-03
Gpr141	G protein-coupled receptor 141	17290894	3.848	3.94E-07
Gpr165	G protein-coupled receptor 165	17536420	-2.039	3.90E-06
Gpr171	G protein-coupled receptor 171	17405458	2.755	3.72E-05
Gpr18	G protein-coupled receptor 18	17309644	1.610	1.61E-03
Gpr182	G protein-coupled receptor 182	17246038	1.078	3.54E-03
Gpr34	G protein-coupled receptor 34	17533446	-2.417	4.32E-10
Gpr35	G protein-coupled receptor 35	17215873	2.127	1.59E-06
Gpr65	G protein-coupled receptor 65	17277794	2.574	9.56E-09
Gpr84	G protein-coupled receptor 84	17322355	2.022	1.10E-06
Gprc5B	G protein-coupled receptor, class C, group 5, member B	17495610	-1.070	3.14E-03
Grb14	growth factor receptor-bound protein 14	17385853	-1.119	3.33E-04
Grina	glutamate receptor, ionotropic, N-methyl D-aspartate-associated protein 1 (glutamate binding)	17312341	1.856	2.02E-06
Gsap	gamma-secretase activating protein	17435055	1.833	1.36E-04
Gsdmc	gasdermin C	17317472	-1.174	3.08E-04
Gsr	glutathione reductase	17500478	1.454	1.17E-06
Gsto1	glutathione S-transferase omega 1	17360216	1.590	1.42E-05
Gstt2/Gstt2B	glutathione S-transferase theta 2 (gene/pseudogene)	17241954	-1.469	1.73E-06
Gtf2F1	general transcription factor IIF, polypeptide 1, 74kDa	17346412	1.031	9.04E-07
Gtf2F2	general transcription factor IIF, polypeptide 2, 30kDa	17308734	1.473	7.01E-06
Gtpbp4	GTP binding protein 4	17290398	1.015	5.24E-06
Gusb	glucuronidase, beta	17453160	1.387	6.13E-06
Gvin1 (Includes	GTPase, very large interferon inducible 1	17494677	3.110	3.65E-07
Gzma	granzyme A (granzyme 1, cytotoxic T-lymphocyte-associated serine esterase 3)	17296286	1.253	2.82E-02
Gzmb	granzyme B	17307033	2.336	5.98E-04
H2-K2/H2-Q9	histocompatibility 2, K region locus 2	17337133	1.045	1.43E-02
H2-Q5	histocompatibility 2, Q region locus 5	17337110	1.322	1.61E-03
H2-T10	histocompatibility 2, T region locus 10	17344642	1.501	2.07E-03
H2-T22	histocompatibility 2, T region locus 22	17344593	2.083	9.65E-04
Hacd2	3-hydroxyacyl-CoA dehydratase 2	17325198	1.186	1.48E-05
Hat1	histone acetyltransferase 1	17371739	1.250	1.14E-04
Haus7	HAUS augmin-like complex, subunit 7	17542349	1.087	2.44E-03
Hcar1	hydroxycarboxylic acid receptor 1	17452709	-1.228	2.42E-05
Hcar2	hydroxycarboxylic acid receptor 2	17452705	4.612	1.17E-09
Hck	HCK proto-oncogene, Src family tyrosine kinase	17377870	1.918	1.22E-06
Hcls1	hematopoietic cell-specific Lyn substrate 1	17325438	1.653	1.15E-06
Hcn2	hyperpolarization activated cyclic nucleotide gated potassium channel 2	17234936	-1.025	1.29E-03
Hcst	hematopoietic cell signal transducer	17489046	1.661	1.36E-03
Hdac7	histone deacetylase 7	17321046	-1.113	6.03E-05
Hdc	histidine decarboxylase	17391094	2.698	1.14E-04
Heatr1	HEAT repeat containing 1	17285157	1.653	2.58E-08
Heatr3	HEAT repeat containing 3	17503572	1.101	5.43E-06
Hells	helicase, lymphoid-specific	17359212	1.587	2.78E-06
Herc6	HECT and RLD domain containing E3 ubiquitin protein ligase family member 6	17458962	1.099	3.82E-04
Hes1	hes family bHLH transcription factor 1	17324623	-1.027	5.89E-04
Hey1	hes-related family bHLH transcription factor with YRPW motif 1	17404011	-1.496	5.59E-04
Hgf	hepatocyte growth factor (hepapoietin A; scatter factor)	17434933	1.543	9.13E-03
Hif1A	hypoxia inducible factor 1, alpha subunit (basic helix-loop-helix transcription factor)	17276328	1.208	4.21E-07
Hilpda	hypoxia inducible lipid droplet-associated	17456554	1.307	4.31E-04
Hist1H1B	histone cluster 1, H1b	17291005	2.946	1.20E-04
Hist1H2Aa	histone cluster 1, H2aa	17291001	1.546	8.32E-05
Hist1H2Ad	histone cluster 1, H2ad	17285867	2.633	4.37E-05
Hist1H2Aj	histone cluster 1, H2aj	17285819	1.461	3.86E-04
Hist1H2Bl	histone cluster 1, H2bl	17290997	1.527	5.25E-05
Hist1H3C	histone cluster 1, H3c	17400549	-1.310	7.39E-06
Hist2H2Ac	histone cluster 2, H2ac	17408011	1.366	9.12E-04
Hk2	hexokinase 2	17468018	1.321	8.85E-05
Hk3	hexokinase 3 (white cell)	17292775	2.593	1.04E-06
Hla-A	major histocompatibility complex, class I, A	17337122	1.248	6.29E-03
Hla-Dma	major histocompatibility complex, class II, DM alpha	17336407	1.218	6.21E-04
Hla-Dmb	major histocompatibility complex, class II, DM beta	17336414	1.626	3.32E-03
Hla-Dqa1	major histocompatibility complex, class II, DQ alpha 1	17343813	3.079	1.81E-06
Hla-Dqb1	major histocompatibility complex, class II, DQ beta 1	17336494	3.172	2.34E-07
Hla-Drb5	major histocompatibility complex, class II, DR beta 5	17336502	2.144	2.50E-04
Hlf	hepatic leukemia factor	17267702	-1.956	2.97E-07
Hmcn1	hemicentin 1	17227910	-2.201	6.34E-06
Hmgb2	high mobility group box 2	17501292	1.131	6.33E-05
Hmgcs2	3-hydroxy-3-methylglutaryl-CoA synthase 2 (mitochondrial)	17400862	-1.030	1.31E-02
Hmmr	hyaluronan-mediated motility receptor (RHAMM)	17261840	1.564	1.26E-06
Hmox1	heme oxygenase 1	17502573	1.983	1.96E-05

Supplementary Table 3. Differentially expressed transcripts in vascular fragments between EAE and naive mice at the progression phase

Gene symbol	Gene name	Affymetrix ID	Fold Change	Adj. *P* value
Hn1	hematological and neurological expressed 1	17271920	1.289	2.33E-05
Hn1L	hematological and neurological expressed 1-like	17342101	1.436	1.23E-04
Hnrnpab	heterogeneous nuclear ribonucleoprotein A/B	17262478	1.020	2.47E-07
Hnrnpd	heterogeneous nuclear ribonucleoprotein D (AU-rich element RNA binding protein 1, 37kDa)	17450022	1.231	2.40E-04
Hoxa7	homeobox A7	17466861	-1.075	1.02E-04
Hoxb6	homeobox B6	17255581	-1.225	1.64E-03
Hp	haptoglobin	17512809	3.769	2.40E-07
Hpgd	hydroxyprostaglandin dehydrogenase 15-(NAD)	17501250	-1.418	1.96E-06
Hpse	heparanase	17548411	2.994	9.96E-06
Hs3St3B1	heparan sulfate (glucosamine) 3-O-sulfotransferase 3B1	17264102	1.025	7.53E-04
Hspa1A/Hspa1	heat shock 70kDa protein 1A	17344132	1.479	3.26E-02
Hspa1B	heat shock protein 1B	17344126	2.174	3.63E-03
Hspa5	heat shock 70kDa protein 5 (glucose-regulated protein, 78kDa)	17370103	1.663	1.25E-08
Hspa8	heat shock 70kDa protein 8	17516383	1.637	9.13E-04
Hspe1	heat shock 10kDa protein 1	17212882	1.228	2.14E-05
Hsph1	heat shock 105kDa/110kDa protein 1	17455507	1.158	6.47E-03
Hyal1	hyaluronoglucosaminidase 1	17521428	-1.149	9.89E-04
Hyou1	hypoxia up-regulated 1	17516564	1.080	7.97E-06
Ibtk	inhibitor of Bruton agammaglobulinemia tyrosine kinase	17529322	1.067	6.89E-05
Icam1	intercellular adhesion molecule 1	17515074	1.705	3.84E-09
Icos	inducible T-cell co-stimulator	17213490	1.593	1.22E-02
Id2	inhibitor of DNA binding 2, dominant negative helix-loop-helix protein	17280310	1.790	9.57E-06
Ier3	immediate early response 3	17337228	1.726	5.43E-06
Ifi16	interferon, gamma-inducible protein 16	17230111	5.386	1.59E-06
Ifi27L2A/Ifi27L2	interferon, alpha-inducible protein 27 like 2A	17283549	2.165	6.81E-05
Ifi30	interferon, gamma-inducible protein 30	17510136	2.940	3.90E-08
Ifit1	interferon-induced protein with tetratricopeptide repeats 1	17364126	2.442	9.19E-05
Ifit1B	interferon-induced protein with tetratricopeptide repeats 1B	17358832	1.207	2.31E-04
Ifit2	interferon-induced protein with tetratricopeptide repeats 2	17358815	1.289	1.13E-03
Ifitm1	interferon induced transmembrane protein 1	17484701	1.133	7.16E-04
Ifitm6	interferon induced transmembrane protein 6	17497724	2.447	3.05E-07
Ifna4	interferon, alpha 4	17415359	-1.070	1.89E-03
Ifng	interferon, gamma	17237589	2.755	2.43E-05
Igf1R	insulin-like growth factor 1 receptor	17479099	-1.139	4.48E-04
Igf2	insulin-like growth factor 2	17498142	-1.348	2.18E-05
Igfbp3	insulin-like growth factor binding protein 3	17260474	1.038	7.42E-03
Igfbp4	insulin-like growth factor binding protein 4	17256264	1.571	5.64E-05
Igfbp5	insulin-like growth factor binding protein 5	17224180	-1.072	1.63E-02
Igfbp6	insulin-like growth factor binding protein 6	17315305	1.740	1.31E-05
Ighg3	Immunoglobulin heavy constant gamma 3	17284614	-1.542	1.22E-03
Ighm	immunoglobulin heavy constant mu	17284605	-1.595	2.83E-04
Igkv15-103	immunoglobulin kappa chain variable 15-103	17459347	-1.037	9.38E-04
Igsf6	immunoglobulin superfamily, member 6	17495839	2.120	6.78E-05
Igtp	interferon gamma induced GTPase	17249980	2.179	6.50E-06
Iigp1	interferon inducible GTPase 1	17350925	1.442	1.95E-03
Ikbke	inhibitor of kappa light polypeptide gene enhancer in B-cells, kinase epsilon	17226771	2.158	1.73E-06
Il12Rb2	interleukin 12 receptor, beta 2	17467323	1.424	5.66E-04
Il15Ra	interleukin 15 receptor, alpha	17367004	1.012	2.01E-04
Il17A	interleukin 17A	17211369	2.592	4.82E-05
Il17Ra	interleukin 17 receptor A	17462351	1.562	1.95E-06
Il18Bp	interleukin 18 binding protein	17493995	1.508	1.01E-03
Il18R1	interleukin 18 receptor 1	17212229	1.286	1.12E-02
Il18Rap	interleukin 18 receptor accessory protein	17212252	2.463	1.89E-06
Il1A	interleukin 1, alpha	17391554	2.209	3.82E-08
Il1B	interleukin 1, beta	17391565	5.305	4.32E-10
Il1R1	interleukin 1 receptor, type I	17212185	1.221	6.03E-04
Il1R2	interleukin 1 receptor, type II	17212174	3.259	3.56E-06
Il1Rn	interleukin 1 receptor antagonist	17367686	3.916	1.30E-05
Il2	interleukin 2	17404880	1.840	2.80E-03
Il2Ra	interleukin 2 receptor, alpha	17366992	1.629	3.01E-03
Il2Rb	interleukin 2 receptor, beta	17319009	2.353	1.01E-03
Il2Rg	interleukin 2 receptor, gamma	17543572	1.215	1.59E-06
Il3	interleukin 3	17262823	1.323	2.75E-02
Il36G	interleukin 36, gamma	17367652	3.055	2.40E-04
Il4R	interleukin 4 receptor	17482943	2.350	1.15E-08
Il6	interleukin 6	17435725	3.198	1.06E-05
Il7R	interleukin 7 receptor	17315891	1.568	3.82E-02
Inhba	inhibin, beta A	17285438	3.533	5.69E-05
Inpp5D	inositol polyphosphate-5-phosphatase D	17215309	1.543	3.64E-05
Insl6	insulin-like 6	17363790	1.338	1.14E-03
Iqgap2	IQ motif containing GTPase activating protein 2	17295136	1.433	9.30E-05
Irf1	interferon regulatory factor 1	17249593	1.497	2.47E-06
Irf5	interferon regulatory factor 5	17456692	1.252	1.07E-04
Irf8	interferon regulatory factor 8	17506279	1.420	5.41E-05
Irg1	immunoresponsive 1 homolog (mouse)	17302475	4.930	3.60E-06
Irgm	immunity-related GTPase family, M	17249990	1.042	9.85E-04
Irgm1	immunity-related GTPase family M member 1	17262202	1.795	1.14E-05
Isg15	ISG15 ubiquitin-like modifier	17434023	1.626	2.11E-04
Isg20	interferon stimulated exonuclease gene 20kDa	17479317	1.176	1.69E-04
Itga5	integrin, alpha 5 (fibronectin receptor, alpha polypeptide)	17322369	1.409	3.99E-07
Itgal	integrin, alpha L (antigen CD11A (p180), lymphocyte function-associated antigen 1; alpha poly	17483264	2.677	1.08E-06

Supplementary Table 3. Differentially expressed transcripts in vascular fragments between EAE and naive mice at the progression phase

Gene symbol	Gene name	Affymetrix ID	Fold Change	Adj. *P* value
Itgax	integrin, alpha X (complement component 3 receptor 4 subunit)	17483615	2.667	3.64E-05
Itgb2	integrin, beta 2 (complement component 3 receptor 3 and 4 subunit)	17234647	2.620	1.16E-07
Itgb7	integrin, beta 7	17322163	1.864	1.02E-04
Itgb8	integrin, beta 8	17284839	-1.038	1.10E-02
Itih4	inter-alpha-trypsin inhibitor heavy chain family, member 4	17298267	3.219	2.50E-05
Itih5	inter-alpha-trypsin inhibitor heavy chain family, member 5	17366670	-1.882	6.13E-06
Itk	IL2-inducible T-cell kinase	17262102	1.234	2.90E-03
Itm2A	integral membrane protein 2A	17544078	-1.344	2.01E-04
Jam2	junctional adhesion molecule 2	17326801	-1.038	1.12E-04
Jpx	Jpx transcript, Xist activator (non-protein coding)	17536984	-1.075	1.44E-04
Kank3	KN motif and ankyrin repeat domains 3	17336175	-1.092	3.33E-04
Kcna3	potassium channel, voltage gated shaker related subfamily A, member 3	17401526	1.528	1.90E-03
Kcnj10	potassium channel, inwardly rectifying subfamily J, member 10	17219536	-1.225	4.18E-04
Kcnk2	potassium channel, two pore domain subfamily K, member 2	17230902	-1.292	3.07E-04
Kcnmb4Os2	potassium large conductance calcium-activated channel, subfamily M, beta member 4, opposi	17237496	-1.060	1.59E-04
Kcnn4	potassium channel, calcium activated intermediate/small conductance subfamily N alpha, mer	17474941	1.650	7.36E-04
Kctd12B	potassium channel tetramerisation domain containing 12b	17545488	-1.490	4.02E-05
Kdr	kinase insert domain receptor	17448924	-1.403	1.17E-05
Kiaa0101	KIAA0101	17518636	1.463	7.92E-05
Kiaa1524	KIAA1524	17326081	1.205	7.01E-05
Kif11	kinesin family member 11	17359020	2.410	1.53E-06
Kif15	kinesin family member 15	17523494	1.484	1.45E-04
Kif20B	kinesin family member 20B	17358838	1.434	2.30E-05
Kif23	kinesin family member 23	17527934	1.013	1.71E-04
Kif26A	kinesin family member 26A	17279299	-1.033	2.33E-05
Kif2C	kinesin family member 2C	17428803	1.381	8.20E-06
Kifc3	kinesin family member C3	17511927	-1.223	3.12E-05
Kitlg	KIT ligand	17236900	-1.202	6.81E-05
Klf12	Kruppel-like factor 12	17309099	-1.153	3.60E-04
Klhl38	kelch-like family member 38	17317278	-1.006	8.70E-03
Klhl6	kelch-like family member 6	17329063	1.116	3.09E-05
Klk3	kallikrein-related peptidase 3	17477391	1.250	3.74E-02
Klra2	killer cell lectin-like receptor, subfamily A, member 2	17471828	3.744	5.36E-07
Klrb1	killer cell lectin-like receptor subfamily B, member 1	17471464	3.383	1.10E-05
Klrc1	killer cell lectin-like receptor subfamily C, member 1	17471598	1.413	8.35E-03
Klrk1	killer cell lectin-like receptor subfamily K, member 1	17471565	1.675	3.54E-03
Kmo	kynurenine 3-monooxygenase (kynurenine 3-hydroxylase)	17219789	1.286	8.17E-03
Kpnb1	karyopherin (importin) beta 1	17547634	1.048	2.51E-05
Krt222	keratin 222, type II	17268995	-1.485	5.17E-06
Krtap10-7	keratin associated protein 10-7	17242322	-1.186	4.69E-03
Krtap5-1	keratin associated protein 5-1	17498084	-1.623	3.80E-04
Lacc1	laccase (multicopper oxidoreductase) domain containing 1	17308772	1.341	2.85E-04
Lap3	leucine aminopeptidase 3	17437247	1.687	1.27E-07
Larp1	La ribonucleoprotein domain family, member 1	17249929	1.187	1.12E-06
Lat	linker for activation of T cells	17496150	2.103	1.08E-03
Layn	layilin	17526956	-1.540	4.45E-06
Lbp	lipopolysaccharide binding protein	17378827	1.644	4.73E-03
Lck	LCK proto-oncogene, Src family tyrosine kinase	17430413	1.331	4.05E-03
Lcn2	lipocalin 2	17383892	4.090	3.12E-08
Lcp1	lymphocyte cytosolic protein 1 (L-plastin)	17301968	1.830	2.16E-08
Lcp2	lymphocyte cytosolic protein 2 (SH2 domain containing leukocyte protein of 76kDa)	17248380	1.668	1.61E-07
Ldlrad3	low density lipoprotein receptor class A domain containing 3	17388705	-1.232	3.11E-05
Lef1	lymphoid enhancer-binding factor 1	17402662	-1.060	4.19E-04
Leo1	LEO1 homolog, Paf1/RNA polymerase II complex component	17519509	1.343	2.61E-06
Lepr	leptin receptor	17415979	-1.028	5.43E-03
Lgals1	lectin, galactoside-binding, soluble, 1	17312829	1.735	1.41E-05
Lgals3	lectin, galactoside-binding, soluble, 3	17299329	2.864	2.78E-06
Lgmn	legumain	17283445	1.185	2.09E-05
Lig1	ligase I, DNA, ATP-dependent	17473856	1.492	3.78E-06
Lilrb3	leukocyte immunoglobulin-like receptor, subfamily B (with TM and ITIM domains), member 3	17485589	2.565	4.52E-06
Lilrb4	leukocyte immunoglobulin-like receptor, subfamily B (with TM and ITIM domains), member 4	17233226	5.213	1.33E-09
Limch1	LIM and calponin homology domains 1	17437887	-1.200	2.03E-04
Lims2	LIM and senescent cell antigen-like domains 2	17349209	-1.147	1.56E-04
Litaf	lipopolysaccharide-induced TNF factor	17328124	1.385	5.96E-07
Llph	LLP homolog, long-term synaptic facilitation (Aplysia)	17548354	1.208	5.98E-05
Lmnb1	lamin B1	17350740	1.895	3.56E-06
Lmo7	LIM domain 7	17302429	-1.684	2.76E-05
Lnx2	ligand of numb-protein X 2	17455281	-1.113	3.78E-04
Loc102638993	uncharacterized LOC102638993	17480924	2.493	1.34E-04
Loc102641083	uncharacterized LOC102641083	17472772	-1.139	1.64E-05
Loc73899	uncharacterized LOC73899	17360344	1.028	2.91E-02
Lpar4	lysophosphatidic acid receptor 4	17537112	-1.306	1.11E-04
Lrat	lecithin retinol acyltransferase (phosphatidylcholine--retinol O-acyltransferase)	17406247	1.125	1.52E-02
Lrg1	leucine-rich alpha-2-glycoprotein 1	17346150	2.704	7.13E-07
Lrp12	low density lipoprotein receptor-related protein 12	17316754	1.209	1.35E-04
Lrrc16A	leucine rich repeat containing 16A	17291275	1.027	5.75E-05
Lrrc25	leucine rich repeat containing 25	17501989	1.278	1.92E-03
Lrrc49	leucine rich repeat containing 49	17527883	-1.508	7.37E-08
Lrrc8D	leucine rich repeat containing 8 family, member D	17450529	1.010	3.48E-04
Lrrfip1	leucine rich repeat (in FLII) interacting protein 1	17215650	1.055	3.68E-05
Lrrn3	leucine rich repeat neuronal 3	17280888	-2.530	4.63E-07

Supplementary Table 3. Differentially expressed transcripts in vascular fragments between EAE and naive mice at the progression phase

Gene symbol	Gene name	Affymetrix ID	Fold Change	Adj. *P* value
Lsamp	limbic system-associated membrane protein	17325671	-1.327	2.01E-03
Lsm12	LSM12 homolog	17270071	1.094	3.36E-04
Lsm3	LSM3 homolog, U6 small nuclear RNA and mRNA degradation associated	17460933	1.226	6.80E-04
Ltb	lymphotoxin beta (TNF superfamily, member 3)	17337024	1.374	4.64E-03
Ltb4R	leukotriene B4 receptor	17300666	1.990	1.28E-04
Ltbp4	latent transforming growth factor beta binding protein 4	17488179	-1.851	1.06E-05
Ltc4S	leukotriene C4 synthase	17262316	-1.516	2.08E-07
Ltv1	LTV1 ribosome biogenesis factor	17239371	1.309	2.34E-07
Lurap1L	leucine rich adaptor protein 1-like	17414984	-1.118	3.01E-05
Ly6A (Includes	lymphocyte antigen 6 complex, locus A	17318076	3.994	8.18E-05
Ly86	lymphocyte antigen 86	17286587	1.009	2.72E-05
Ly9	lymphocyte antigen 9	17229767	1.396	5.77E-03
Lynx1	Ly6/neurotoxin 1	17318013	-1.390	1.48E-05
Lyz	lysozyme	17245223	1.738	7.57E-07
Lzts1	leucine zipper, putative tumor suppressor 1	17509801	-1.169	1.73E-03
Lzts2	leucine zipper, putative tumor suppressor 2	17359796	-1.160	8.96E-05
Mad2L1	MAD2 mitotic arrest deficient-like 1 (yeast)	17459236	1.367	6.75E-06
Mageh1	melanoma antigen family H1	17545482	-1.042	2.47E-04
Magohb	mago homolog B, exon junction complex core component	17471840	1.013	8.15E-04
Malt1	MALT1 paracaspase	17351330	1.491	1.02E-04
Mamld1	mastermind-like domain containing 1	17535284	-1.207	2.74E-04
Man2A1	mannosidase, alpha, class 2A, member 1	17339108	1.003	5.57E-05
Maob	monoamine oxidase B	17540378	-1.247	1.44E-03
Map2K6	mitogen-activated protein kinase kinase 6	17257906	-1.693	2.40E-07
Map3K6	mitogen-activated protein kinase kinase kinase 6	17419553	1.064	3.71E-03
Map3K8	mitogen-activated protein kinase kinase kinase 8	17352401	1.520	8.60E-06
Map4K1	mitogen-activated protein kinase kinase kinase kinase 1	17476036	1.368	1.96E-03
Map4K2	mitogen-activated protein kinase kinase kinase kinase 2	17356811	-1.148	6.43E-05
Mapk6	mitogen-activated protein kinase 6	17528836	1.323	2.28E-06
Mapkapk2	mitogen-activated protein kinase-activated protein kinase 2	17226736	1.554	2.58E-08
Mapkapk3	mitogen-activated protein kinase-activated protein kinase 3	17530863	1.179	3.53E-04
Marcksl1-Ps4	MARCKS-like 1, pseudogene 4	17284963	-1.179	2.70E-04
Mat2A	methionine adenosyltransferase II, alpha	17467753	1.114	2.26E-05
Mb21D1	Mab-21 domain containing 1	17529046	1.124	5.97E-04
Mboat2	membrane bound O-acyltransferase domain containing 2	17274558	-1.133	1.76E-03
Mcemp1	mast cell-expressed membrane protein 1	17498730	4.187	2.97E-07
Mcf2L	MCF.2 cell line derived transforming sequence-like	17499155	-1.258	6.70E-05
Mcm2	minichromosome maintenance complex component 2	17468798	1.124	6.93E-05
Mcm3	minichromosome maintenance complex component 3	17221633	1.531	2.63E-06
Mcm5	minichromosome maintenance complex component 5	17502583	1.438	1.69E-05
Mcm6	minichromosome maintenance complex component 6	17226550	1.048	6.33E-05
Mcpt8	mast cell protease 8	17306968	-1.092	3.65E-02
Med11	mediator complex subunit 11	17252032	1.521	4.48E-05
Mefv	Mediterranean fever	17327580	2.294	4.14E-09
Melk	maternal embryonic leucine zipper kinase	17413528	1.037	8.45E-04
Met	MET proto-oncogene, receptor tyrosine kinase	17456176	2.379	1.42E-06
Metap2	methionyl aminopeptidase 2	17244378	1.213	4.99E-06
Metrn	meteorin, glial cell differentiation regulator	17342386	-1.011	2.46E-04
Mfsd2A	major facilitator superfamily domain containing 2A	17429632	-1.546	4.13E-06
Mgat5	mannosyl (alpha-1,6-)-glycoprotein beta-1,6-N-acetyl-glucosaminyltransferase	17216753	1.068	3.33E-05
Mif	macrophage migration inhibitory factor (glycosylation-inhibiting factor)	17241962	1.606	8.15E-07
Milr1	mast cell immunoglobulin like receptor 1	17257599	2.343	1.86E-06
Mir-10	microRNA 100	17326700	-1.524	4.39E-02
Mir-24	microRNA 24-1	17503120	-1.216	1.37E-02
Mir-30	microRNA 30a	17317706	-1.084	7.11E-03
Mir-3065	microRNA 3065	17259262	-1.030	2.49E-04
Mir-3074	microRNA 3074	17511077	-1.672	9.73E-03
Mir-34	microRNA 34a	17526980	-1.115	1.88E-04
Mir-3473	microRNA 3473b	17231114	-1.101	2.61E-03
Mir-450	microRNA 450b	17541719	-1.093	2.81E-03
Mir-598	microRNA 598	17301245	-1.016	2.94E-04
Mir143Hg	Mir143 and Mir145 host gene (non-protein coding)	17354784	-1.024	5.02E-03
Mir17Hg	miR-17-92 cluster host gene	17302632	1.269	4.89E-03
Mir1970	microRNA 1970	17544687	-1.030	2.95E-02
Mir3961	microRNA 3961	17294680	-1.179	1.19E-04
Mir5112	microRNA 5112	17352196	-1.336	4.97E-04
Mirt2	myocardial infraction associated transcript 2	17318422	1.856	9.69E-04
Mki67	marker of proliferation Ki-67	17497334	1.745	1.07E-04
Mlc1	megalencephalic leukoencephalopathy with subcortical cysts 1	17320225	-1.083	2.51E-02
Mlkl	mixed lineage kinase domain-like	17513076	1.481	1.94E-04
Mmp12	matrix metallopeptidase 12	17514495	1.255	1.30E-02
Mmp14	matrix metallopeptidase 14 (membrane-inserted)	17300279	2.792	1.64E-07
Mmp19	matrix metallopeptidase 19	17238558	2.674	1.26E-06
Mmp28	matrix metallopeptidase 28	17266911	-1.070	6.87E-04
Mmp8	matrix metallopeptidase 8	17514553	4.118	7.12E-08
Mocos	molybdenum cofactor sulfurase	17349016	1.212	1.15E-04
Mpeg1	macrophage expressed 1	17357810	1.468	1.79E-04
Mpp6	membrane protein, palmitoylated 6 (MAGUK p55 subfamily member 6)	17458520	1.272	1.82E-03
Mpzl1	myelin protein zero-like 1	17229259	-1.044	8.37E-04
Mrps15	mitochondrial ribosomal protein S15	17418507	1.346	1.06E-05
Mrps16	mitochondrial ribosomal protein S16	17303765	1.629	1.01E-03

Supplementary Table 3. Differentially expressed transcripts in vascular fragments between EAE and naive mice at the progression phase

Gene symbol	Gene name	Affymetrix ID	Fold Change	Adj. *P* value
Mrps18B	mitochondrial ribosomal protein S18B	17385483	1.546	1.75E-05
Mrto4	MRT4 homolog, ribosome maturation factor	17431895	1.253	3.71E-05
Ms4A4A	membrane-spanning 4-domains, subfamily A, member 4A	17357640	3.196	3.33E-07
Ms4A4B (Inclu	membrane-spanning 4-domains, subfamily A, member 4B	17357648	3.518	2.34E-06
Ms4A6A	membrane-spanning 4-domains, subfamily A, member 6A	17362973	2.857	2.35E-07
Ms4A6B	membrane-spanning 4-domains, subfamily A, member 6B	17357688	2.242	4.52E-07
Ms4A6C	membrane-spanning 4-domains, subfamily A, member 6C	17357671	3.978	1.49E-09
Ms4A7	membrane-spanning 4-domains, subfamily A, member 7	17362953	1.678	2.81E-02
Ms4A8	membrane-spanning 4-domains, subfamily A, member 8	17362874	3.666	7.29E-06
Msr1	macrophage scavenger receptor 1	17508850	4.886	2.50E-08
Msrb1	methionine sulfoxide reductase B1	17334419	1.583	2.24E-04
Msx1	msh homeobox 1	17447610	-1.064	1.15E-03
Msx1Os	msh homeobox 1 opposite strand	17437049	-1.028	2.21E-05
Mt2	metallothionein 2	17503937	1.236	4.78E-05
Mthfd1	methylenetetrahydrofolate dehydrogenase (NADP+ dependent) 1	17276520	1.138	3.32E-05
Mthfd2	methylenetetrahydrofolate dehydrogenase (NADP+ dependent) 2	17468143	1.298	5.78E-07
Mtss1	metastasis suppressor 1	17317327	-1.013	1.07E-03
Mtss1L	metastasis suppressor 1-like	17505623	-1.618	1.04E-06
Mturn	maturin, neural progenitor differentiation regulator homolog (Xenopus)	17458752	-1.193	5.16E-04
Mvd	mevalonate (diphospho) decarboxylase	17513681	1.166	1.07E-03
Mvp	major vault protein	17496514	1.180	1.27E-05
Mx1/Mx2	MX dynamin-like GTPase 1	17332531	1.204	5.07E-03
Mxd1	MAX dimerization protein 1	17468511	2.118	6.05E-05
Mxd4	MAX dimerization protein 4	17447218	-1.351	7.01E-07
Myc	v-myc avian myelocytomatosis viral oncogene homolog	17311846	1.511	2.30E-06
Myct1	myc target 1	17231477	-1.948	5.73E-06
Myd88	myeloid differentiation primary response 88	17532137	1.138	1.44E-05
Myl6	myosin, light chain 6, alkali, smooth muscle and non-muscle	17246146	1.304	2.45E-04
Myo1D	myosin ID	17266698	-1.176	3.75E-05
Myo1F	myosin IF	17336114	1.373	5.80E-04
Myo1G	myosin IG	17260369	1.611	7.75E-05
Myo5A	myosin VA	17519394	2.597	7.48E-07
Myo6	myosin VI	17519821	-1.230	2.37E-04
N-R5S136	nuclear encoded rRNA 5S 136	17513976	-1.262	1.71E-02
Naaa	N-acylethanolamine acid amidase	17449673	3.475	3.07E-08
Nabp1	nucleic acid binding protein 1	17222825	2.259	1.85E-06
Naip	NLR family, apoptosis inhibitory protein	17295569	1.197	8.07E-03
Napsa	napsin A aspartic peptidase	17477508	2.940	5.79E-08
Nasp	nuclear autoantigenic sperm protein (histone-binding)	17428659	1.118	8.08E-06
Ncapg	non-SMC condensin I complex, subunit G	17437278	1.539	3.11E-05
Ncapg2	non-SMC condensin II complex, subunit G2	17279608	1.527	1.14E-04
Ncf1	neutrophil cytosolic factor 1	17453288	1.158	4.27E-04
Ncf2	neutrophil cytosolic factor 2	17218261	1.298	1.16E-05
Ncf4	neutrophil cytosolic factor 4, 40kDa	17312700	1.510	2.62E-05
Ndc80	NDC80 kinetochore complex component	17347042	2.733	1.16E-06
Ndnf	neuron-derived neurotrophic factor	17459207	-3.170	1.71E-09
Ndrg2	NDRG family member 2	17306147	-1.199	6.32E-04
Ndufa11	NADH dehydrogenase (ubiquinone) 1 alpha subcomplex, 11, 14.7kDa	17338872	1.215	3.15E-03
Ndufs5	NADH dehydrogenase (ubiquinone) Fe-S protein 5	17325719	1.058	2.53E-03
Nes	nestin	17398903	-1.191	5.53E-04
Neurl2	neuralized E3 ubiquitin protein ligase 2	17394292	-1.015	3.51E-03
Neurl3	neuralized E3 ubiquitin protein ligase 3	17222149	1.055	1.30E-03
Nfam1	NFAT activating protein with ITAM motif 1	17319738	1.224	3.52E-03
Nfat5	nuclear factor of activated T-cells 5, tonicity-responsive	17505260	-1.017	5.87E-06
Nfia	nuclear factor I/A	17415606	-1.056	1.78E-05
Nfil3	nuclear factor, interleukin 3 regulated	17292634	2.861	1.00E-08
Nfkbie	nuclear factor of kappa light polypeptide gene enhancer in B-cells inhibitor, epsilon	17337927	1.135	5.20E-04
Nfkbiz	nuclear factor of kappa light polypeptide gene enhancer in B-cells inhibitor, zeta	17330967	1.147	2.01E-06
Nhp2	NHP2 ribonucleoprotein	17249347	1.476	3.32E-07
Nid2	nidogen 2 (osteonidogen)	17297391	-1.244	3.57E-05
Nkd1	naked cuticle homolog 1 (Drosophila)	17503650	-1.443	1.60E-05
Nkg7	natural killer cell granule protein 7	17477101	1.385	8.65E-03
Nlrc5	NLR family, CARD domain containing 5	17504023	1.780	6.58E-05
Nlrp1A	NLR family, pyrin domain containing 1A	17265473	1.192	4.38E-04
Nlrp3	NLR family, pyrin domain containing 3	17250249	2.299	1.60E-07
Noc4L	nucleolar complex associated 4 homolog	17451043	2.025	7.61E-08
Nolc1	nucleolar and coiled-body phosphoprotein 1	17359902	1.007	8.89E-04
Nop16	NOP16 nucleolar protein	17292712	1.134	1.01E-04
Nop56	NOP56 ribonucleoprotein	17376252	1.181	3.05E-07
Nop58	NOP58 ribonucleoprotein	17213295	1.329	1.93E-07
Nop9	NOP9 nucleolar protein	17300650	1.178	1.69E-05
Nos2	nitric oxide synthase 2, inducible	17253707	3.039	4.48E-05
Notch3	notch 3	17343299	-1.059	1.28E-03
Notum	notum pectinacetylesterase homolog (Drosophila)	17273260	-1.134	2.13E-03
Npl	N-acetylneuraminate pyruvate lyase (dihydrodipicolinate synthase)	17228234	1.837	4.65E-05
Nr1D1	nuclear receptor subfamily 1, group D, member 1	17268884	-2.336	2.52E-07
Nr1D2	nuclear receptor subfamily 1, group D, member 2	17303625	-1.197	6.04E-04
Nr3C2	nuclear receptor subfamily 3, group C, member 2	17502626	-1.250	1.14E-04
Nrep	neuronal regeneration related protein	17353370	-1.692	1.27E-05
Nrg1	neuregulin 1	17508609	1.732	2.08E-04
Nrxn1	neurexin 1	17348016	-1.059	6.03E-04

Supplementary Table 3. Differentially expressed transcripts in vascular fragments between EAE and naive mice at the progression phase

Gene symbol	Gene name	Affymetrix ID	Fold Change	Adj. *P* value
Nt5C3A	5'-nucleotidase, cytosolic IIIA	17467128	1.307	5.34E-05
Nt5Dc2	5'-nucleotidase domain containing 2	17298364	1.234	1.19E-04
Nt5E	5'-nucleotidase, ecto (CD73)	17520073	2.987	4.42E-08
Ntn3	netrin 3	17341758	-1.106	6.03E-04
Ntpcr	nucleoside-triphosphatase, cancer-related	17506891	1.162	5.65E-05
Ntsr2	neurotensin receptor 2	17274195	-1.440	2.67E-03
Nuak1	NUAK family, SNF1-like kinase, 1	17243604	-1.078	4.22E-05
Nuak2	NUAK family, SNF1-like kinase, 2	17217182	1.852	1.04E-05
Nuf2	NUF2, NDC80 kinetochore complex component	17229433	2.446	9.88E-08
Nup210	nucleoporin 210kDa	17468961	1.326	4.77E-05
Nusap1	nucleolar and spindle associated protein 1	17374833	1.155	5.30E-03
Nutf2	nuclear transport factor 2	17504958	1.097	8.53E-03
Nxpe4	neurexophilin and PC-esterase domain family, member 4	17516978	-1.109	2.11E-04
Oas1	2'-5'-oligoadenylate synthetase 1, 40/46kDa	17452115	2.882	1.56E-04
Oas2	2'-5'-oligoadenylate synthetase 2, 69/71kDa	17452054	1.759	1.98E-05
Oas3	2'-5'-oligoadenylate synthetase 3, 100kDa	17452070	1.784	1.27E-04
Oasl	2'-5'-oligoadenylate synthetase-like	17441051	1.524	1.83E-03
Oasl2	2'-5' oligoadenylate synthetase-like 2	17441037	1.469	1.48E-05
Ocln	occludin	17295670	-1.107	1.38E-04
Odc1	ornithine decarboxylase 1	17274294	1.126	1.49E-04
Olfml2A	olfactomedin-like 2A	17370487	-1.040	7.23E-03
Olfr12	olfactory receptor 12	17215817	-1.182	5.80E-05
Olfr126 (Includ	olfactory receptor 126	17337614	-1.034	1.90E-04
Olfr212/Olfr21	olfactory receptor 213	17462139	-1.012	3.55E-03
Olfr338	olfactory receptor 338	17370326	-1.190	8.38E-04
Olr1	oxidized low density lipoprotein (lectin-like) receptor 1	17471550	3.685	3.16E-05
Or10A6	olfactory receptor, family 10, subfamily A, member 6 (gene/pseudogene)	17494820	-1.099	9.68E-03
Or2At4	olfactory receptor, family 2, subfamily AT, member 4	17480596	-1.155	4.86E-03
Or2V1	olfactory receptor, family 2, subfamily V, member 1	17248911	1.610	9.81E-05
Or56A3	olfactory receptor, family 56, subfamily A, member 3	17481350	-1.030	2.81E-03
Or9Q2	olfactory receptor, family 9, subfamily Q, member 2	17363162	-1.028	9.88E-04
Ormdl2	ORMDL sphingolipid biosynthesis regulator 2	17246345	1.925	3.09E-03
Osm	oncostatin M	17246803	2.942	1.65E-07
Osmr	oncostatin M receptor	17315743	1.103	4.47E-03
P2Rx4	purinergic receptor P2X, ligand gated ion channel, 4	17442149	1.436	8.75E-05
P2Ry10	purinergic receptor P2Y, G-protein coupled, 10	17537118	1.515	1.26E-03
P2Ry6	pyrimidinergic receptor P2Y, G-protein coupled, 6	17493869	1.111	7.23E-04
P4Ha1	prolyl 4-hydroxylase, alpha polypeptide I	17233536	1.223	4.84E-07
Papss2	3'-phosphoadenosine 5'-phosphosulfate synthase 2	17358690	1.185	3.93E-03
Parm1	prostate androgen-regulated mucin-like protein 1	17439037	-1.279	1.55E-03
Parp9	poly (ADP-ribose) polymerase family, member 9	17325274	1.048	4.65E-05
Pbk	PDZ binding kinase	17301428	1.977	2.71E-07
Pcbp1	poly(rC) binding protein 1	17468505	1.066	1.12E-04
Pcgf5	polycomb group ring finger 5	17358891	1.476	2.49E-05
Pcp4L1	Purkinje cell protein 4-like 1	17229639	-1.171	1.19E-04
Pdcd1Lg2	programmed cell death 1 ligand 2	17358552	2.102	1.22E-04
Pde2A	phosphodiesterase 2A, cGMP-stimulated	17480880	-1.624	1.96E-06
Pdgfb	platelet-derived growth factor beta polypeptide	17319380	-1.241	1.21E-05
Pdia6	protein disulfide isomerase family A, member 6	17274249	1.279	2.40E-07
Pdk3	pyruvate dehydrogenase kinase, isozyme 3	17543196	1.108	6.81E-05
Pdpn	podoplanin	17432440	1.559	8.30E-03
Peg13	paternally expressed 13	17317835	-1.134	1.58E-04
Per2	period circadian clock 2	17225506	-1.141	9.32E-06
Per3	period circadian clock 3	17433328	-2.242	4.14E-09
Pfkm	phosphofructokinase, muscle	17314577	-1.256	1.70E-04
Pfkp	phosphofructokinase, platelet	17290324	2.045	8.27E-06
Pgd	phosphogluconate dehydrogenase	17433040	1.567	1.14E-05
Pgk1	phosphoglycerate kinase 1	17537088	1.981	7.79E-08
Pgm5	phosphoglucomutase 5	17363626	-1.461	1.37E-04
Phf10	PHD finger protein 10	17341132	1.578	5.46E-04
Phf11	PHD finger protein 11	17307280	2.319	3.56E-06
Phxr1	per-hexamer repeat gene 1	17366423	1.040	3.27E-02
Phyhipl	phytanoyl-CoA 2-hydroxylase interacting protein-like	17241709	-1.041	1.70E-02
Pi4K2A	phosphatidylinositol 4-kinase type 2 alpha	17359520	1.137	2.04E-03
Pigf	phosphatidylinositol glycan anchor biosynthesis, class F	17347888	1.163	1.08E-04
Pign	phosphatidylinositol glycan anchor biosynthesis, class N	17225999	1.024	5.45E-04
Pik3Ap1	phosphoinositide-3-kinase adaptor protein 1	17364642	1.789	4.84E-07
Pik3R5	phosphoinositide-3-kinase, regulatory subunit 5	17251303	1.060	1.15E-04
Pilra	paired immunoglobin-like type 2 receptor alpha	17454166	1.485	2.96E-05
Pilrb	paired immunoglobin-like type 2 receptor beta	17454179	1.383	2.87E-02
Pim1	Pim-1 proto-oncogene, serine/threonine kinase	17335540	2.643	8.10E-08
Pink1	PTEN induced putative kinase 1	17431749	-1.281	7.65E-05
Pinx1	PIN2/TERF1 interacting, telomerase inhibitor 1	17301247	1.307	2.08E-06
Pitpnm3	PITPNM family member 3	17265570	-1.148	1.20E-04
Pla1A	phospholipase A1 member A	17330359	1.055	1.44E-03
Pla2G4A	phospholipase A2, group IVA (cytosolic, calcium-dependent)	17227828	1.058	3.36E-04
Pla2G7	phospholipase A2, group VII (platelet-activating factor acetylhydrolase, plasma)	17337796	1.745	6.15E-04
Plac8	placenta-specific 8	17450121	4.051	3.06E-08
Plau	plasminogen activator, urokinase	17297537	1.523	3.29E-04
Plaur	plasminogen activator, urokinase receptor	17474974	2.917	1.60E-07
Plbd1	phospholipase B domain containing 1	17472114	4.037	2.40E-07

Supplementary Table 3. Differentially expressed transcripts in vascular fragments between EAE and naive mice at the progression phase

Gene symbol	Gene name	Affymetrix ID	Fold Change	Adj. *P* value
Plek	pleckstrin	17260761	1.576	4.52E-07
Plekhh2	pleckstrin homology domain containing, family H (with MyTH4 domain) member 2	17340050	-1.734	1.74E-06
Plekho1	pleckstrin homology domain containing, family O member 1	17407969	1.063	2.51E-04
Plekho2	pleckstrin homology domain containing, family O member 2	17528274	2.206	1.99E-06
Plet1	placenta expressed transcript 1	17517097	1.684	9.79E-04
Plin2	perilipin 2	17426981	1.803	5.53E-05
Plk1	polo-like kinase 1	17482739	1.781	1.92E-05
Plk3	polo-like kinase 3	17428766	1.594	4.65E-07
Plk4	polo-like kinase 4	17397268	1.173	1.14E-03
Pllp	plasmolipin	17511878	-1.396	5.15E-04
Pln	phospholamban	17233323	-1.730	1.34E-04
Plod1	procollagen-lysine, 2-oxoglutarate 5-dioxygenase 1	17432835	1.007	2.36E-05
Pltp	phospholipid transfer protein	17394297	-1.507	1.92E-05
Plxna4	plexin A4	17465636	-1.182	3.13E-05
Plxnc1	plexin C1	17244439	1.438	6.71E-03
Pmp2	peripheral myelin protein 2	17404077	-1.097	4.83E-02
Pnp	purine nucleoside phosphorylase	17299551	1.495	5.01E-07
Pnpla6	patatin-like phospholipase domain containing 6	17498625	-1.546	7.79E-06
Pole	polymerase (DNA directed), epsilon, catalytic subunit	17440465	1.108	2.84E-05
Polk	polymerase (DNA directed) kappa	17295212	-1.443	1.71E-05
Polr1A	polymerase (RNA) I polypeptide A, 194kDa	17459550	1.090	1.96E-06
Pomc	proopiomelanocortin	17273694	-1.392	1.85E-06
Ppa1	pyrophosphatase (inorganic) 1	17233720	1.498	3.48E-06
Ppp1R14B	protein phosphatase 1, regulatory (inhibitor) subunit 14B	17356987	1.388	1.59E-06
Ppp1R3B	protein phosphatase 1, regulatory subunit 3B	17500543	1.033	2.73E-04
Pram1	PML-RARA regulated adaptor molecule 1	17336157	1.155	1.75E-03
Prc1	protein regulator of cytokinesis 1	17479575	1.516	6.35E-05
Prdx1	peroxiredoxin 1	17548658	1.758	3.21E-05
Prdx5	peroxiredoxin 5	17362101	1.570	1.07E-05
Prg2	proteoglycan 2, bone marrow (natural killer cell activator, eosinophil granule major basic prote	17372662	-1.996	1.53E-02
Prickle1	prickle homolog 1	17320720	-1.432	2.93E-05
Prim1	primase, DNA, polypeptide 1 (49kDa)	17238210	1.553	2.36E-05
Prkcd	protein kinase C, delta	17304406	1.130	6.21E-04
Procr	protein C receptor, endothelial	17378440	1.139	2.38E-03
Prom1	prominin 1	17447835	-1.795	2.12E-06
Proser2	proline and serine rich 2	17381448	-1.212	1.97E-05
Prr11	proline rich 11	17267454	2.268	6.55E-07
Prrt2	proline-rich transmembrane protein 2	17496547	-1.028	1.30E-02
Prss23Os	protease, serine 23, opposite strand	17480044	-1.526	5.06E-04
Psat1	phosphoserine aminotransferase 1	17363204	1.383	9.62E-06
Psd2	pleckstrin and Sec7 domain containing 2	17349607	-1.198	3.84E-04
Psd4	pleckstrin and Sec7 domain containing 4	17367698	1.908	9.17E-05
Psma1	proteasome subunit alpha 1	17495297	1.206	1.01E-03
Psmb8	proteasome subunit beta 8	17336446	1.174	6.50E-05
Psmb9	proteasome subunit beta 9	17343789	1.074	7.38E-04
Psmd10	proteasome 26S subunit, non-ATPase 10	17544960	1.196	7.43E-04
Pspc1	paraspeckle component 1	17307080	1.315	1.00E-05
Pstpip1	proline-serine-threonine phosphatase interacting protein 1	17517554	1.509	4.75E-04
Pstpip2	proline-serine-threonine phosphatase interacting protein 2	17352036	2.132	7.99E-05
Ptafr	platelet-activating factor receptor	17419437	1.873	1.29E-03
Ptger2	prostaglandin E receptor 2 (subtype EP2), 53kDa	17299180	1.340	1.84E-03
Ptger4	prostaglandin E receptor 4 (subtype EP4)	17315718	1.206	1.24E-04
Ptgfrn	prostaglandin F2 receptor inhibitor	17408483	1.240	4.57E-05
Ptgis	prostaglandin I2 (prostacyclin) synthase	17394679	-1.136	2.90E-04
Ptgr1	prostaglandin reductase 1	17425770	1.432	6.66E-05
Ptgs2	prostaglandin-endoperoxide synthase 2 (prostaglandin G/H synthase and cyclooxygenase)	17218060	3.250	1.11E-07
Pthlh	parathyroid hormone-like hormone	17472903	-1.079	2.22E-04
Ptk2B	protein tyrosine kinase 2 beta	17307860	1.336	1.27E-04
Ptma (Includes	prothymosin alpha	17215115	1.117	9.70E-03
Ptn	pleiotrophin	17465856	-1.744	1.84E-05
Ptp4A1	protein tyrosine phosphatase type IVA, member 1	17364543	1.175	2.75E-06
Ptpn1	protein tyrosine phosphatase, non-receptor type 1	17379960	1.414	1.60E-05
Ptpn22	protein tyrosine phosphatase, non-receptor type 22 (lymphoid)	17401269	1.776	1.05E-03
Ptpn6	protein tyrosine phosphatase, non-receptor type 6	17470796	1.901	2.12E-06
Ptpn7	protein tyrosine phosphatase, non-receptor type 7	17217566	1.318	7.66E-04
Ptprb	protein tyrosine phosphatase, receptor type, B	17237451	-1.079	7.99E-05
Ptprc	protein tyrosine phosphatase, receptor type, C	17227536	1.710	5.35E-07
Ptprm	protein tyrosine phosphatase, receptor type, M	17346856	-1.173	1.70E-04
Ptprz1	protein tyrosine phosphatase, receptor-type, Z polypeptide 1	17456381	-1.595	1.76E-03
Pum3	pumilio RNA-binding family member 3	17363683	1.088	1.45E-06
Pus7L	pseudouridylate synthase 7-like	17320783	1.052	3.83E-04
Pvr	poliovirus receptor	17487489	1.542	6.55E-07
Pycard	PYD and CARD domain containing	17496839	1.609	3.46E-05
Pygl	phosphorylase, glycogen, liver	17281721	2.154	1.54E-05
Pygm	phosphorylase, glycogen, muscle	17356874	-1.038	4.37E-03
Rab10	RAB10, member RAS oncogene family	17279737	1.095	5.17E-04
Rab20	RAB20, member RAS oncogene family	17507435	1.340	3.38E-03
Rab26Os	RAB26, member RAS oncogene family, opposite strand	17334302	1.789	6.74E-03
Rab27A	RAB27A, member RAS oncogene family	17519282	1.789	1.94E-07
Rab32	RAB32, member RAS oncogene family	17239227	2.020	7.57E-07
Rab6B	RAB6B, member RAS oncogene family	17520922	-1.215	1.27E-03

Supplementary Table 3. Differentially expressed transcripts in vascular fragments between EAE and naive mice at the progression phase

Gene symbol	Gene name	Affymetrix ID	Fold Change	Adj. *P* value
Rab7B	RAB7B, member RAS oncogene family	17217048	1.319	1.01E-02
Rac2	ras-related C3 botulinum toxin substrate 2 (rho family, small GTP binding protein Rac2)	17319037	2.703	5.06E-07
Racgap1	Rac GTPase activating protein 1	17321597	1.406	6.25E-05
Rai14	retinoic acid induced 14	17315993	1.096	6.74E-06
Ralgds	ral guanine nucleotide dissociation stimulator	17368685	1.419	3.31E-06
Ramp2	receptor (G protein-coupled) activity modifying protein 2	17256607	-1.007	5.35E-07
Ranbp9	RAN binding protein 9	17292122	1.011	2.22E-03
Rap2C	RAP2C, member of RAS oncogene family	17541612	1.644	3.77E-06
Rars	arginyl-tRNA synthetase	17261730	1.020	7.48E-07
Rasgef1B	RasGEF domain family, member 1B	17449989	1.672	6.81E-05
Rasgrp1	RAS guanyl releasing protein 1 (calcium and DAG-regulated)	17389647	1.419	2.87E-03
Rassf4	Ras association (RalGDS/AF-6) domain family member 4	17470060	1.308	5.52E-04
Rassf8	Ras association (RalGDS/AF-6) domain family (N-terminal) member 8	17464271	-1.117	3.07E-05
Rbm47	RNA binding motif protein 47	17448389	1.109	1.57E-03
Rbpj	recombination signal binding protein for immunoglobulin kappa J region	17466505	1.505	1.91E-07
Rbpms2	RNA binding protein with multiple splicing 2	17548468	-1.178	6.67E-04
Reck	reversion-inducing-cysteine-rich protein with kazal motifs	17413474	-1.364	1.76E-04
Rel	v-rel avian reticuloendotheliosis viral oncogene homolog	17261107	1.293	3.77E-05
Renbp	renin binding protein	17542501	1.270	3.15E-03
Retnlg	resistin like gamma	17326075	1.849	1.20E-02
Rfc4	replication factor C (activator 1) 4, 37kDa	17329380	1.000	8.72E-05
Rftn1	raftlin, lipid raft linker 1	17345865	1.280	1.47E-03
Rgs1	regulator of G-protein signaling 1	17227780	1.713	1.59E-03
Rgs16	regulator of G-protein signaling 16	17218321	2.161	1.36E-04
Rhobtb1	Rho-related BTB domain containing 1	17234042	-1.007	7.65E-05
Rhobtb3	Rho-related BTB domain containing 3	17294458	-1.185	1.16E-02
Rhoh	ras homolog family member H	17437830	1.501	7.59E-05
Rhou	ras homolog family member U	17506697	1.075	2.17E-04
Rhpn2	rhophilin, Rho GTPase binding protein 2	17476752	-1.349	4.14E-05
Rinl	Ras and Rab interactor-like	17475942	1.379	3.14E-05
Ripk2	receptor-interacting serine-threonine kinase 2	17423490	1.399	8.44E-05
Ripk3	receptor-interacting serine-threonine kinase 3	17306906	1.225	3.05E-05
Rnase6	ribonuclease, RNase A family, k6	17299585	1.146	9.83E-03
Rnasel	ribonuclease L (2',5'-oligoisoadenylate synthetase-dependent)	17218328	1.789	7.58E-05
Rnd1	Rho family GTPase 1	17321307	2.123	6.76E-06
Rnf149	ring finger protein 149	17222549	1.436	3.08E-04
Rnf19B	ring finger protein 19B	17418916	1.430	4.19E-05
Rnh1	ribonuclease/angiogenin inhibitor 1	17497769	1.400	7.37E-06
Rnu73B	U73B small nuclear RNA	17406452	1.033	4.81E-03
Rpf2	ribosome production factor 2 homolog	17240342	1.486	5.75E-05
Rpl11	ribosomal protein L11	17411099	1.906	1.50E-05
Rpl13A	ribosomal protein L13a	17490626	-1.352	3.79E-03
Rpl29 (Include:	ribosomal protein L29	17414299	1.017	8.18E-05
Rpl3	ribosomal protein L3	17319403	1.490	5.21E-04
Rpl34 (Include:	ribosomal protein L34	17410381	1.625	1.15E-04
Rpl6	ribosomal protein L6	17441799	1.533	1.90E-04
Rpph1	ribonuclease P RNA component H1	17305986	-1.259	4.86E-05
Rps15	ribosomal protein S15	17235292	-1.029	1.83E-02
Rps23	ribosomal protein S23	17289031	1.549	2.08E-04
Rps8	ribosomal protein S8	17428797	1.032	1.06E-02
Rpsa	ribosomal protein SA	17411141	1.450	2.40E-06
Rrm1	ribonucleotide reductase M1	17481102	1.090	3.87E-05
Rrm2	ribonucleotide reductase M2	17274540	1.402	9.06E-05
Rrp12	ribosomal RNA processing 12 homolog	17364725	1.261	1.60E-05
Rsad2	radical S-adenosyl methionine domain containing 2	17280327	1.174	7.32E-03
Rsl1D1	ribosomal L1 domain containing 1	17328187	1.128	4.98E-06
Runx1	runt-related transcription factor 1	17332236	1.276	3.53E-05
S100A10	S100 calcium binding protein A10	17400000	1.176	3.63E-04
S100A4	S100 calcium binding protein A4	17399802	1.501	7.86E-05
S100A8	S100 calcium binding protein A8	17399823	3.653	4.02E-04
S100A9	S100 calcium binding protein A9	17407363	2.789	2.96E-04
S100B	S100 calcium binding protein B	17234494	-1.636	1.03E-03
Saa1	serum amyloid A1	17478195	1.173	4.14E-02
Saa3	serum amyloid A 3	17491193	5.968	4.61E-07
Samd12	sterile alpha motif domain containing 12	17317031	-1.097	3.01E-03
Samhd1	SAM domain and HD domain 1	17393658	1.375	2.18E-05
Sap18	Sin3A-associated protein, 18kDa	17504367	1.214	1.60E-03
Sap30	Sin3A-associated protein, 30kDa	17509455	1.710	9.82E-07
Sbno2	strawberry notch homolog 2 (Drosophila)	17242785	1.485	6.30E-07
Scarna17	small Cajal body-specific RNA 17	17355435	-1.219	3.96E-05
Scarna2	small Cajal body-specific RNA 2	17409376	-1.111	8.89E-04
Scd	stearoyl-CoA desaturase (delta-9-desaturase)	17365098	-1.148	1.00E-02
Scd2	stearoyl-Coenzyme A desaturase 2	17359689	-1.119	2.27E-05
Scgb3A1	secretoglobin, family 3A, member 1	17249028	3.739	7.01E-07
Scimp	SLP adaptor and CSK interacting membrane protein	17265386	3.599	4.28E-07
Scn4B	sodium channel, voltage gated, type IV beta subunit	17516740	-1.415	9.33E-06
Scnn1A	sodium channel, non voltage gated 1 alpha subunit	17463169	1.222	1.44E-05
Scpep1	serine carboxypeptidase 1	17267601	2.015	2.46E-06
Scrg1	stimulator of chondrogenesis 1	17501283	-1.333	2.23E-03
Sdad1	SDA1 domain containing 1	17449685	1.653	3.02E-07
Sdc1	syndecan 1	17273948	1.596	2.20E-04

Supplementary Table 3. Differentially expressed transcripts in vascular fragments between EAE and naive mice at the progression phase

Gene symbol	Gene name	Affymetrix ID	Fold Change	Adj. *P* value
Sdf2L1	stromal cell-derived factor 2-like 1	17328625	2.106	1.17E-07
Sdpr	serum deprivation response	17212719	-1.562	5.64E-05
Sele	selectin E	17218820	2.862	5.24E-06
Sell	selectin L	17218835	3.716	2.98E-09
Selp	selectin P (granule membrane protein 140kDa, antigen CD62)	17218845	4.540	7.41E-08
Sema4A	semaphorin 4A	17406760	2.389	4.03E-05
Sephs2	selenophosphate synthetase 2	17496651	1.148	9.60E-03
Serp1	stress-associated endoplasmic reticulum protein 1	17405414	1.205	3.82E-06
Serpina3	serpin peptidase inhibitor, clade A (alpha-1 antiproteinase, antitrypsin), member 3	17278328	6.717	1.58E-08
Serpina3G (Inc	serine (or cysteine) peptidase inhibitor, clade A, member 3G	17278268	4.097	3.83E-08
Serpinb1	serpin peptidase inhibitor, clade B (ovalbumin), member 1	17291694	1.310	2.92E-04
Serpine1	serpin peptidase inhibitor, clade E (nexin, plasminogen activator inhibitor type 1), member 1	17453819	2.504	2.97E-04
Setd8	SET domain containing (lysine methyltransferase) 8	17442507	1.103	3.47E-05
Sfxn1	sideroflexin 1	17287414	1.107	2.03E-04
Sgcb	sarcoglycan, beta (43kDa dystrophin-associated glycoprotein)	17448840	1.118	6.64E-04
Sgpl1	sphingosine-1-phosphate lyase 1	17241162	1.194	3.36E-04
Sgpp2	sphingosine-1-phosphate phosphatase 2	17214665	-2.634	3.77E-07
Sh2D2A	SH2 domain containing 2A	17398853	1.292	2.62E-03
Sh3Bgrl	SH3 domain binding glutamate-rich protein like	17537199	2.556	1.92E-05
Sh3Bp2	SH3-domain binding protein 2	17436607	1.049	1.99E-03
Sh3Pxd2B	SH3 and PX domains 2B	17248288	1.670	2.87E-05
Shcbp1	SHC SH2-domain binding protein 1	17507288	1.995	3.71E-05
Shisa4	shisa family member 4	17227311	-1.039	2.41E-03
Shmt2	serine hydroxymethyltransferase 2 (mitochondrial)	17245902	1.333	4.14E-05
Shtn1	shootin 1	17366012	1.053	7.09E-04
Siglec1	sialic acid binding Ig-like lectin 1, sialoadhesin	17391834	2.353	3.14E-04
Sirpb1	signal-regulatory protein beta 1	17404209	3.129	3.54E-07
Siva1	SIVA1, apoptosis-inducing factor	17279365	1.136	3.17E-04
Skap2	src kinase associated phosphoprotein 2	17466783	1.511	4.40E-06
Skp2	S-phase kinase-associated protein 2, E3 ubiquitin protein ligase	17315878	1.154	2.34E-04
Sla	Src-like-adaptor	17317637	2.002	2.92E-06
Slamf1	signaling lymphocytic activation molecule family member 1	17219407	1.032	4.37E-03
Slamf6	SLAM family member 6	17219435	1.162	5.57E-05
Slamf7	SLAM family member 7	17229782	4.325	2.72E-06
Slamf8	SLAM family member 8	17229931	2.985	1.06E-05
Slc10A6	solute carrier family 10 (sodium/bile acid cotransporter), member 6	17450319	2.078	8.95E-05
Slc11A1	solute carrier family 11 (proton-coupled divalent metal ion transporter), member 1	17214197	1.396	7.95E-04
Slc15A3	solute carrier family 15 (oligopeptide transporter), member 3	17357597	2.409	2.58E-08
Slc16A1	solute carrier family 16 (monocarboxylate transporter), member 1	17401335	-1.212	5.06E-04
Slc16A10	solute carrier family 16 (aromatic amino acid transporter), member 10	17240330	1.184	1.79E-03
Slc16A2	solute carrier family 16, member 2 (thyroid hormone transporter)	17543817	-1.501	4.17E-05
Slc16A3	solute carrier family 16 (monocarboxylate transporter), member 3	17259534	1.677	7.61E-05
Slc16A4	solute carrier family 16, member 4	17401563	-1.926	2.12E-06
Slc19A3	solute carrier family 19 (thiamine transporter), member 3	17224942	-1.623	1.06E-04
Slc1A2	solute carrier family 1 (glial high affinity glutamate transporter), member 2	17373696	-1.037	1.16E-03
Slc1A5	solute carrier family 1 (neutral amino acid transporter), member 5	17474143	1.029	6.53E-04
Slc20A1	solute carrier family 20 (phosphate transporter), member 1	17376124	1.318	2.76E-07
Slc22A8	solute carrier family 22 (organic anion transporter), member 8	17357092	-1.995	3.54E-04
Slc25A13	solute carrier family 25 (aspartate/glutamate carrier), member 13	17464672	1.130	2.17E-04
Slc26A10	solute carrier family 26, member 10	17245729	-2.507	2.98E-09
Slc2A5	solute carrier family 2 (facilitated glucose/fructose transporter), member 5	17421875	-1.199	2.93E-05
Slc2A6	solute carrier family 2 (facilitated glucose transporter), member 6	17383216	1.751	8.20E-06
Slc31A2	solute carrier family 31 (copper transporter), member 2	17414536	1.001	3.61E-04
Slc35F2	solute carrier family 35, member F2	17517349	-1.214	1.27E-05
Slc38A1	solute carrier family 38, member 1	17320907	1.252	1.08E-04
Slc38A11	solute carrier family 38, member 11	17385902	-1.015	5.19E-03
Slc38A3	solute carrier family 38, member 3	17530967	-1.555	1.69E-05
Slc39A12	solute carrier family 39 (zinc transporter), member 12	17367139	-1.000	7.77E-03
Slc39A14	solute carrier family 39 (zinc transporter), member 14	17308299	1.372	1.49E-06
Slc39A6	solute carrier family 39 (zinc transporter), member 6	17353153	1.009	2.33E-05
Slc40A1	solute carrier family 40 (iron-regulated transporter), member 1	17222777	-2.618	2.58E-08
Slc43A3	solute carrier family 43, member 3	17372644	1.316	3.30E-03
Slc4A7	solute carrier family 4, sodium bicarbonate cotransporter, member 7	17297227	1.013	5.80E-03
Slc6A11	solute carrier family 6 (neurotransmitter transporter), member 11	17461852	-1.042	3.55E-02
Slc7A1	solute carrier family 7 (cationic amino acid transporter, y+ system), member 1	17455401	-1.202	2.72E-07
Slc7A11	solute carrier family 7 (anionic amino acid transporter light chain, xc- system), member 11	17405082	2.445	2.58E-04
Slc7A2	solute carrier family 7 (cationic amino acid transporter, y+ system), member 2	17500716	1.949	1.36E-04
Slc7A5	solute carrier family 7 (amino acid transporter light chain, L system), member 5	17513641	-1.344	8.43E-06
Slc7A6	solute carrier family 7 (amino acid transporter light chain, y+L system), member 6	17505069	1.144	1.45E-06
Slc7A8	solute carrier family 7 (amino acid transporter light chain, L system), member 8	17306477	1.391	1.69E-04
Slc9A3R2	solute carrier family 9, subfamily A (NHE3, cation proton antiporter 3), member 3 regulator 2	17341963	-1.015	5.99E-04
Slco1C1	solute carrier organic anion transporter family, member 1C1	17464063	-1.157	4.55E-04
Slfn1	schlafen 1	17254171	4.537	6.09E-07
Slfn12L	schlafen family member 12-like	17254176	4.287	7.14E-06
Slfn13	schlafen family member 13	17266851	2.521	4.88E-05
Slfn2	schlafen 2	17254166	2.144	1.15E-06
Slpi	secretory leukocyte peptidase inhibitor	17394153	3.719	8.92E-07
Smarcc1	SWI/SNF related, matrix associated, actin dependent regulator of chromatin, subfamily c, men	17522284	1.002	5.97E-05
Smc2	structural maintenance of chromosomes 2	17414114	1.673	5.01E-07
Smco4	single-pass membrane protein with coiled-coil domains 4	17514841	-1.298	5.11E-06
Smcr8	Smith-Magenis syndrome chromosome region, candidate 8	17250511	1.052	2.28E-04

Supplementary Table 3. Differentially expressed transcripts in vascular fragments between EAE and naive mice at the progression phase

Gene symbol	Gene name	Affymetrix ID	Fold Change	Adj. *P* value
Smim3	small integral membrane protein 3	17354595	1.113	2.96E-05
Smpdl3B	sphingomyelin phosphodiesterase, acid-like 3B	17430894	1.610	1.05E-03
Smtn	smoothelin	17259831	-1.126	5.83E-04
Snai2	snail family zinc finger 2	17323192	-1.993	1.82E-06
Sncaip	synuclein, alpha interacting protein	17350591	-1.407	5.42E-06
Sned1	sushi, nidogen and EGF-like domains 1	17215932	-1.364	1.58E-04
Snhg18	small nucleolar RNA host gene 18	17316345	-1.065	7.85E-03
Snhg3	small nucleolar RNA host gene 3	17430833	1.006	3.28E-04
Snhg4	small nucleolar RNA host gene 4	17349549	2.135	2.37E-07
Snhg6	small nucleolar RNA host gene 6	17221186	1.302	3.57E-06
Snora20	small nucleolar RNA, H/ACA box 20	17333344	2.660	1.33E-05
Snora23	small nucleolar RNA, H/ACA box 23	17481723	1.201	2.51E-02
Snora33	small nucleolar RNA, H/ACA box 33	17239751	1.054	2.96E-03
Snora73B	small nucleolar RNA, H/ACA box 73b	17430831	2.003	3.00E-05
Snora75	small nucleolar RNA, H/ACA box 75	17225169	1.388	1.34E-02
Snord118	small nucleolar RNA, C/D box 118	17251514	1.887	1.37E-04
Snord22	small nucleolar RNA, C/D box 22	17357126	1.159	7.98E-07
Snord35B	small nucleolar RNA, C/D box 35B	17490606	-3.027	5.25E-07
Snord42B	small nucleolar RNA, C/D box 42B	17266372	-1.011	3.31E-02
Snord43	small nucleolar RNA, C/D box 43	17319405	1.034	1.67E-02
Snord65	small nucleolar RNA, C/D box 65	17250744	1.235	1.24E-02
Snord72	small nucleolar RNA, C/D box 72	17309905	1.594	2.13E-03
Snord87	small nucleolar RNA, C/D box 87	17221191	1.936	4.70E-04
Snord91A	small nucleolar RNA, C/D box 91A	17252845	-1.210	9.74E-03
Snord99	small nucleolar RNA, C/D box 99	17419411	1.255	1.31E-04
Snrpa1	small nuclear ribonucleoprotein polypeptide A'	17478985	1.217	7.65E-05
Snrpf	small nuclear ribonucleoprotein polypeptide F	17244362	1.204	1.73E-05
Snx10	sorting nexin 10	17458573	1.428	1.88E-05
Snx20	sorting nexin 20	17511534	1.171	6.69E-04
Soat1	sterol O-acyltransferase 1	17228544	1.726	4.37E-05
Soat2	sterol O-acyltransferase 2	17315312	1.517	1.70E-06
Socs1	suppressor of cytokine signaling 1	17328104	2.561	5.09E-06
Socs2	suppressor of cytokine signaling 2	17274184	1.261	1.91E-02
Sod2	superoxide dismutase 2, mitochondrial	17333347	1.890	3.90E-08
Sorl1	sortilin-related receptor, L(DLR class) A repeats containing	17525894	1.392	3.67E-06
Sox13	SRY (sex determining region Y)-box 13	17226974	-1.598	1.65E-07
Sox17	SRY (sex determining region Y)-box 17	17221071	-1.421	1.48E-05
Sox18	SRY (sex determining region Y)-box 18	17395928	-1.236	8.04E-06
Sox4	SRY (sex determining region Y)-box 4	17291525	-1.737	3.05E-07
Sox7	SRY (sex determining region Y)-box 7	17301256	-1.496	9.50E-05
Sp110	SP110 nuclear body protein	17366258	1.481	1.93E-05
Sp140	SP140 nuclear body protein	17214924	1.502	3.40E-03
Spc24	SPC24, NDC80 kinetochore complex component	17524752	1.116	1.78E-03
Spdl1	spindle apparatus coiled-coil protein 1	17261710	1.441	6.79E-05
Spi1	Spi-1 proto-oncogene	17373177	1.269	5.32E-04
Spib	Spi-B transcription factor (Spi-1/PU.1 related)	17490274	-1.085	1.24E-02
Spint1	serine peptidase inhibitor, Kunitz type 1	17374765	2.000	1.08E-06
Spock2	sparc/osteonectin, cwcv and kazal-like domains proteoglycan (testican) 2	17233613	-2.004	1.22E-06
Spp1	secreted phosphoprotein 1	17439830	3.551	1.05E-06
Srm	spermidine synthase	17421694	1.060	3.73E-05
Sstr4	somatostatin receptor 4	17377344	-1.078	1.72E-03
St14	suppression of tumorigenicity 14 (colon carcinoma)	17525240	1.336	4.24E-05
St6Galnac3	ST6 (alpha-N-acetyl-neuraminyl-2,3-beta-galactosyl-1,3)-N-acetylgalactosaminide alpha-2,6-s	17411262	-1.145	2.24E-03
St8Sia4	ST8 alpha-N-acetyl-neuraminide alpha-2,8-sialyltransferase 4	17225815	-1.491	1.04E-06
Stard8	StAR-related lipid transfer (START) domain containing 8	17536463	-1.019	8.24E-05
Stat1	signal transducer and activator of transcription 1, 91kDa	17212750	1.730	5.51E-06
Stat2	signal transducer and activator of transcription 2, 113kDa	17238367	1.476	1.02E-05
Stat4	signal transducer and activator of transcription 4	17212724	1.690	7.30E-03
Steap4	STEAP family member 4	17434490	2.023	4.99E-04
Stfa1 (Includes	stefin A1	17325347	1.130	2.14E-02
Stfa2/Stfa2L1	stefin A2	17325324	4.356	5.01E-07
Stip1	stress-induced phosphoprotein 1	17362240	1.113	1.90E-05
Stk17B	serine/threonine kinase 17b	17223069	1.239	9.74E-05
Stmn2	stathmin 2	17396024	-1.245	4.75E-04
Stxbp2	syntaxin binding protein 2	17498699	1.053	2.84E-03
Sulf2	sulfatase 2	17394538	1.404	2.75E-04
Synpo2	synaptopodin 2	17410031	-1.228	5.87E-04
Taar7D	trace amine-associated receptor 7D	17232189	-1.090	7.41E-03
Tacc3	transforming, acidic coiled-coil containing protein 3	17436457	1.790	2.95E-06
Taf1D	TATA box binding protein (TBP)-associated factor, RNA polymerase I, D, 41kDa	17514832	1.759	5.85E-07
Tank	TRAF family member-associated NFKB activator	17371101	1.031	1.40E-04
Tap1	transporter 1, ATP-binding cassette, sub-family B (MDR/TAP)	17336432	2.226	3.63E-06
Tapbpl	TAP binding protein-like	17471062	1.006	2.85E-04
Tarm1	T cell-interacting, activating receptor on myeloid cells 1	17485510	4.179	1.10E-06
Tars	threonyl-tRNA synthetase	17316021	1.125	3.06E-05
Tbc1D9	TBC1 domain family, member 9 (with GRAM domain)	17502874	1.277	1.35E-03
Tbx3	T-box 3	17441595	-1.151	8.70E-04
Tbxa2R	thromboxane A2 receptor	17235694	-1.173	1.06E-05
Tbxas1	thromboxane A synthase 1 (platelet)	17457472	1.098	2.47E-04
Tceb1	transcription elongation factor B (SIII), polypeptide 1 (15kDa, elongin C)	17221497	1.334	5.00E-03
Tcf7	transcription factor 7, T cell specific	17262600	-1.174	4.04E-06

Supplementary Table 3. Differentially expressed transcripts in vascular fragments between EAE and naive mice at the progression phase

Gene symbol	Gene name	Affymetrix ID	Fold Change	Adj. *P* value
Tcf7L1	transcription factor 7-like 1 (T-cell specific, HMG-box)	17467806	-1.022	5.52E-05
Tcp1	t-complex 1	17333323	1.106	1.62E-07
Tcrg-V4	T cell receptor gamma, variable 4	17285523	1.563	1.35E-02
Tdgf1	teratocarcinoma-derived growth factor 1	17531685	-1.183	5.04E-04
Tdrp	testis development related protein	17507673	-1.245	1.69E-06
Tecrl	trans-2,3-enoyl-CoA reductase-like	17449108	-1.253	5.28E-03
Tef	thyrotrophic embryonic factor	17313376	-1.953	4.32E-10
Tfdp1	transcription factor Dp-1	17499310	1.189	5.84E-04
Tfec	transcription factor EC	17464950	2.829	2.80E-05
Tfrc	transferrin receptor	17324835	-2.067	1.58E-08
Tgfb2	transforming growth factor, beta 2	17230830	-2.317	1.02E-07
Tgfbi	transforming growth factor, beta-induced, 68kDa	17287827	4.191	4.76E-08
Tgfbr3	transforming growth factor, beta receptor III	17450618	-1.277	6.41E-05
Tgm1	transglutaminase 1	17306816	1.354	1.14E-06
Tgm2	transglutaminase 2	17393789	1.165	9.44E-06
Tgtp1/Tgtp2	T cell specific GTPase 1	17262250	1.195	3.77E-02
Thbs1	thrombospondin 1	17374488	3.236	1.74E-06
Themis2	thymocyte selection associated family member 2	17430906	2.191	5.49E-06
Thra	thyroid hormone receptor, alpha	17256138	-1.159	2.18E-05
Thsd1	thrombospondin, type I, domain containing 1	17499874	-1.236	9.44E-06
Thy1	Thy-1 cell surface antigen	17516462	1.825	1.78E-03
Tie1	tyrosine kinase with immunoglobulin-like and EGF-like domains 1	17429206	-1.582	1.99E-06
Tigit	T cell immunoreceptor with Ig and ITIM domains	17330478	1.382	2.23E-02
Timm23	translocase of inner mitochondrial membrane 23 homolog (yeast)	17304860	1.265	7.12E-08
Timm8A	translocase of inner mitochondrial membrane 8 homolog A (yeast)	17231676	1.474	2.97E-07
Timp1	TIMP metallopeptidase inhibitor 1	17533713	3.635	7.40E-08
Tiparp	TCDD-inducible poly(ADP-ribose) polymerase	17398082	2.008	4.80E-08
Tlr1	toll-like receptor 1	17448245	1.298	1.55E-03
Tlr13	toll-like receptor 13	17537081	1.260	5.62E-04
Tlr2	toll-like receptor 2	17406279	1.887	1.73E-07
Tlr4	toll-like receptor 4	17414836	1.372	5.06E-06
Tlr6	toll-like receptor 6	17448251	1.021	1.28E-02
Tlr7	toll-like receptor 7	17546109	1.093	1.41E-03
Tlr8	toll-like receptor 8	17546101	2.172	8.53E-04
Tma16	translation machinery associated 16 homolog	17509697	1.413	1.35E-04
Tmc7	transmembrane channel-like 7	17495566	-1.136	7.77E-04
Tmed3	transmembrane p24 trafficking protein 3	17529634	1.283	1.08E-05
Tmem104	transmembrane protein 104	17258140	1.087	6.32E-04
Tmem106A	transmembrane protein 106A	17256784	2.310	1.84E-05
Tmem150C	transmembrane protein 150C	17450049	-1.096	5.75E-04
Tmem156	transmembrane protein 156	17448256	1.045	1.76E-04
Tmem173	transmembrane protein 173	17353663	1.263	5.06E-06
Tmem176A	transmembrane protein 176A	17458393	1.396	2.00E-04
Tmem189	transmembrane protein 189	17394727	1.124	8.92E-04
Tmem229A	transmembrane protein 229A	17465229	-1.006	2.69E-03
Tmem252	transmembrane protein 252	17358266	1.319	4.05E-04
Tmem44	transmembrane protein 44	17329675	-1.256	3.96E-05
Tmem45A	transmembrane protein 45A	17331078	1.111	8.66E-04
Tmem47	transmembrane protein 47	17536067	-1.033	1.77E-03
Tmem97	transmembrane protein 97	17266489	1.374	3.00E-05
Tmem98	transmembrane protein 98	17253996	-1.118	3.57E-04
Tmtc2	transmembrane and tetratricopeptide repeat containing 2	17244661	-1.628	2.08E-05
Tnfa	tumor necrosis factor alpha	17344309	3.587	4.32E-10
Tnfaip2	tumor necrosis factor, alpha-induced protein 2	17279131	2.867	1.35E-05
Tnfaip3	tumor necrosis factor, alpha-induced protein 3	17239597	1.142	2.16E-05
Tnfrsf17	tumor necrosis factor receptor superfamily, member 17	17322944	-1.451	1.29E-04
Tnfrsf19	tumor necrosis factor receptor superfamily, member 19	17307433	-1.551	9.45E-06
Tnfrsf1B	tumor necrosis factor receptor superfamily, member 1B	17432674	1.400	3.56E-06
Tnfrsf22/Tnfrsf	tumor necrosis factor receptor superfamily, member 23	17498323	2.088	1.62E-04
Tnfrsf26	tumor necrosis factor receptor superfamily, member 26	17498301	1.960	1.10E-03
Tnfrsf4	tumor necrosis factor receptor superfamily, member 4	17422859	1.704	1.06E-03
Tnfrsf9	tumor necrosis factor receptor superfamily, member 9	17421981	2.000	1.71E-05
Tnfsf13B	tumor necrosis factor (ligand) superfamily, member 13b	17498897	1.513	4.62E-05
Tnfsf8	tumor necrosis factor (ligand) superfamily, member 8	17426356	3.077	2.46E-06
Tnfsf9	tumor necrosis factor (ligand) superfamily, member 9	17338959	1.508	3.77E-03
Tomm5	translocase of outer mitochondrial membrane 5 homolog (yeast)	17548454	1.469	2.83E-05
Top2A	topoisomerase (DNA) II alpha	17268909	2.922	1.15E-08
Topbp1	topoisomerase (DNA) II binding protein 1	17520932	1.237	3.47E-06
Tor3A	torsin family 3, member A	17228563	1.050	8.21E-05
Tox3	TOX high mobility group box family member 3	17511550	-1.092	6.81E-05
Tp53Inp2	tumor protein p53 inducible nuclear protein 2	17378348	-1.097	3.24E-05
Tpcn1	two pore segment channel 1	17451987	-1.086	5.43E-06
Tpd52L1	tumor protein D52-like 1	17240089	-1.345	2.64E-05
Tpi1	triosephosphate isomerase 1	17470879	1.726	1.73E-07
Tpx2	TPX2, microtubule-associated	17377793	1.382	4.25E-07
Traf3Ip3	TRAF3 interacting protein 3	17231203	1.025	3.54E-03
Trafd1	TRAF-type zinc finger domain containing 1	17452178	1.315	1.12E-05
Trav14-1	T cell receptor alpha variable 14-1	17300165	2.279	1.15E-04
Trav7-4	T cell receptor alpha variable 7-4	17300155	1.192	1.57E-02
Trbv1	T cell receptor beta, variable 1	17457804	2.724	8.88E-04
Trbv13-2	T cell receptor beta, variable 13-2	17457694	1.080	2.29E-02

Supplementary Table 3. Differentially expressed transcripts in vascular fragments between EAE and naive mice at the progression phase

Gene symbol	Gene name	Affymetrix ID	Fold Change	Adj. *P* value
Trem1	triggering receptor expressed on myeloid cells 1	17338364	3.051	6.83E-07
Trem3	triggering receptor expressed on myeloid cells 3	17338371	1.310	7.44E-05
Treml2	triggering receptor expressed on myeloid cells-like 2	17338388	2.378	2.72E-06
Trex1	three prime repair exonuclease 1	17531260	1.802	2.72E-06
Trib1	tribbles pseudokinase 1	17311831	1.328	5.06E-06
Trib2	tribbles pseudokinase 2	17280054	-1.100	1.24E-04
Tril	TLR4 interactor with leucine-rich repeats	17466932	-2.217	2.76E-08
Trim30A/Trim3	tripartite motif-containing 30A	17494408	1.277	9.34E-04
Trim30B	tripartite motif-containing 30B	17494370	1.399	4.01E-04
Trim30C	tripartite motif-containing 30C	17494386	2.492	4.61E-07
Trim59	tripartite motif containing 59	17405789	1.192	2.30E-04
Trip13	thyroid hormone receptor interactor 13	17294302	1.177	2.17E-06
Tspan13	tetraspanin 13	17280749	-1.068	1.54E-04
Tsta3	tissue specific transplantation antigen P35B	17318232	1.048	3.18E-04
Ttc39B	tetratricopeptide repeat domain 39B	17426855	1.611	5.42E-06
Ttc39C	tetratricopeptide repeat domain 39C	17348570	1.152	8.91E-04
Ttyh1	tweety family member 1	17473248	-1.349	2.59E-03
Tuba4A	tubulin, alpha 4a	17224540	2.234	1.60E-07
Tubb6	tubulin, beta 6 class V	17351465	1.880	2.68E-07
Tufm	Tu translation elongation factor, mitochondrial	17509721	1.088	1.86E-03
Twf2	twinfilin actin binding protein 2	17521159	1.083	6.92E-04
Txnrd1	thioredoxin reductase 1	17235915	1.224	8.41E-06
Ubb	ubiquitin B	17299247	1.346	1.28E-02
Ubd	ubiquitin D	17337545	2.744	2.37E-04
Ube2C	ubiquitin-conjugating enzyme E2C	17379523	2.627	4.42E-08
Ube2L6	ubiquitin-conjugating enzyme E2L 6	17372604	1.059	8.45E-04
Uchl5	ubiquitin carboxyl-terminal hydrolase L5	17218027	1.262	9.86E-06
Uck2	uridine-cytidine kinase 2	17229391	2.095	5.34E-07
Ugcg	UDP-glucose ceramide glucosyltransferase	17414434	1.130	1.35E-05
Ugp2	UDP-glucose pyrophosphorylase 2	17260916	1.039	3.27E-04
Uhrf1	ubiquitin-like with PHD and ring finger domains 1	17338747	2.458	2.91E-08
Umps	uridine monophosphate synthetase	17329937	1.537	1.03E-05
Unc13C	unc-13 homolog C (C. elegans)	17528800	-1.170	4.51E-03
Unc5B	unc-5 netrin receptor B	17241137	-1.230	2.80E-03
Upp1	uridine phosphorylase 1	17247225	3.820	2.38E-10
Uqcrhl	ubiquinol-cytochrome c reductase hinge protein like	17428545	1.171	2.02E-04
Usb1	U6 snRNA biogenesis 1	17504281	1.207	5.13E-04
Ushbp1	Usher syndrome 1C binding protein 1	17510295	-1.637	5.83E-07
Utrn	utrophin	17239268	-1.007	1.02E-03
Vav1	vav 1 guanine nucleotide exchange factor	17338982	1.348	5.75E-05
Vav3	vav 3 guanine nucleotide exchange factor	17401846	1.084	1.74E-04
Vcan	versican	17294738	1.928	1.06E-09
Vcpkmt	valosin containing protein lysine (K) methyltransferase	17281575	1.169	3.69E-04
Vdr	vitamin D (1,25- dihydroxyvitamin D3) receptor	17321078	1.910	6.81E-05
Vegfa	vascular endothelial growth factor A	17345293	2.363	1.05E-04
Vegfc	vascular endothelial growth factor C	17501160	-1.753	2.21E-04
Vmn1R20/Vmr	vomeronasal 1 receptor 27	17458960	-1.420	6.86E-04
Vmn2R55 (Incl	vomeronasal 2, receptor 55	17486549	-1.032	2.77E-03
Vmp1	vacuole membrane protein 1	17267329	1.306	3.25E-08
Wars	tryptophanyl-tRNA synthetase	17283930	1.387	3.32E-05
Was	Wiskott-Aldrich syndrome	17539997	1.212	1.22E-03
Wdhd1	WD repeat and HMG-box DNA binding protein 1	17305757	1.593	2.76E-07
Wdr43	WD repeat domain 43	17339476	1.035	1.40E-04
Wdr46	WD repeat domain 46	17336268	1.437	3.71E-05
Wdr74	WD repeat domain 74	17357150	1.487	2.28E-06
Wfdc1	WAP four-disulfide core domain 1	17506137	-1.164	3.98E-04
Wfdc17	WAP four-disulfide core domain 17	17254289	3.935	2.02E-06
Wfdc21	WAP four-disulfide core domain 21	17254300	2.373	2.77E-05
Whsc1	Wolf-Hirschhorn syndrome candidate 1	17436507	1.203	3.71E-06
Wwtr1	WW domain containing transcription regulator 1	17405365	-1.047	2.83E-04
Xbp1	X-box binding protein 1	17246967	1.450	4.54E-06
Xist	X inactive specific transcript (non-protein coding)	17543785	-1.820	4.42E-02
Yae1D1	Yae1 domain containing 1	17404474	1.137	1.46E-02
Zap70	zeta-chain (TCR) associated protein kinase 70kDa	17211867	1.231	1.37E-04
Zbp1	Z-DNA binding protein 1	17395079	4.550	4.42E-08
Zbtb4	zinc finger and BTB domain containing 4	17251728	-1.223	1.13E-06
Zc3H12C	zinc finger CCCH-type containing 12C	17527016	1.156	1.90E-03
Zc3H7B	zinc finger CCCH-type containing 7B	17313350	-1.214	2.77E-06
Zcchc6	zinc finger, CCHC domain containing 6	17293280	1.106	1.93E-03
Zdhhc2	zinc finger, DHHC-type containing 2	17500662	1.027	2.15E-02
Zdhhc21	zinc finger, DHHC-type containing 21	17426791	1.023	1.40E-04
Zfp108/Zfp93	zinc finger protein 93	17474916	-1.414	1.85E-03
Zfp119B	zinc finger protein 119b	17346069	-1.352	5.13E-03
Zfp442/Zfp937	zinc finger protein 442	17377448	-1.050	1.09E-03
Zfp532	zinc finger protein 532	17351351	-1.028	3.85E-04
Zfp759	zinc finger protein 759	17288294	-1.167	7.64E-03
Zfp763	zinc finger protein 763	17343527	-1.005	9.71E-05
Zic2	Zic family member 2	17302878	-1.296	4.95E-05
Zic3	Zic family member 3	17535076	-1.357	2.43E-05
Znf354A	zinc finger protein 354A	17249262	-1.123	7.45E-04
Znf358	zinc finger protein 358	17498602	-1.358	4.76E-07

Supplementary Table 3. Differentially expressed transcripts in vascular fragments between EAE and naive mice at the progression phase

Gene symbol	Gene name	Affymetrix ID	Fold Change	Adj. *P* value
Znf366	zinc finger protein 366	17289432	-1.095	5.16E-04
Znf546	zinc finger protein 546	17453106	-1.031	1.54E-03
Znf593	zinc finger protein 593	17431259	1.014	3.36E-04
Znf638	zinc finger protein 638	17460152	1.150	5.92E-03
Znf705A	zinc finger protein 705A	17462729	1.956	2.30E-06
Znf784	zinc finger protein 784	17485993	-1.096	3.10E-05
Zyx	zyxin	17457942	1.382	8.16E-06
1110038B12Ri	RIKEN cDNA 1110038B12 gene	17344114	1.010	1.08E-04
1500012F01Ri	RIKEN cDNA 1500012F01 gene	17379871	1.646	4.79E-05
1700042G15Ri	RIKEN cDNA 1700042G15 gene	17425597	-1.018	2.36E-05
1700054O19Ri	RIKEN cDNA 1700054O19 gene	17304145	1.084	3.10E-02
1700071M16Ri	RIKEN cDNA 1700071M16 gene	17345089	1.334	3.95E-04
1700084C06Ri	RIKEN cDNA 1700084C06 gene	17515103	1.109	4.46E-04
1700109K24Ri	RIKEN cDNA 1700109K24 gene	17312653	-1.103	1.81E-03
2200002D01Ri	RIKEN cDNA 2200002D01 gene	17488799	1.086	2.30E-04
2810029C07Ri	RIKEN cDNA 2810029C07 gene	17284111	-1.099	6.08E-03
3110021N24Ri	RIKEN cDNA 3110021N24 gene	17416743	-1.086	5.27E-04
4930429F24Ri	RIKEN cDNA 4930429F24 gene	17548765	-1.027	3.76E-03
4930430E12Ri	RIKEN cDNA 4930430E12 gene	17423060	1.164	3.82E-04
4930486L24Ril	RIKEN cDNA 4930486L24 gene	17293338	1.054	4.89E-04
4930515G16Ri	myc induced nuclear antigen pseudogene	17467363	-1.022	3.56E-03
5430402E10Ri	predicted gene 14744	17536028	-1.096	3.73E-03
5730408K05Ri	RIKEN cDNA 5730408K05 gene	17362521	1.523	1.92E-04
8030442B05Ri	RIKEN cDNA 8030442B05 gene	17381626	-1.431	1.57E-04
9430037G07Ri	RIKEN cDNA 9430037G07 gene	17529555	1.899	4.32E-05
9930111J21Ril	predicted gene 12185	17262218	1.035	1.55E-03
A530040E14Ri	RIKEN cDNA A530040E14 gene	17214892	1.556	5.04E-04
A630001O12R	RIKEN cDNA A630001O12 gene	17506942	1.016	1.08E-02
A630077J23Ri	RIKEN cDNA A630077J23 gene	17413466	1.030	8.40E-03
A730049H05R	RIKEN cDNA A730049H05 gene	17461045	1.023	5.47E-04
A730082K24Ri	RIKEN cDNA A730082K24 gene	17482072	-1.058	4.18E-04
A930004D18R	RIKEN cDNA A930004D18 gene	17381992	-1.068	9.21E-05

Supplementary Table 4. Differentially expressed transcripts in vascular fragments between EAE and naive mice at the remission phase

Gene symbol	Gene name	Affymetrix ID	Fold Change	Adj. palue
Abca1	ATP-binding cassette, sub-family A (ABC1), member 1	17425301	1.145	4.11E-04
Abcb1B	ATP-binding cassette, sub-family B (MDR/TAP), member 1B	17434555	1.696	3.67E-05
Abcc6	ATP-binding cassette, sub-family C (CFTR/MRP), member 6	17491035	-1.076	8.54E-05
Abcd2	ATP-binding cassette, sub-family D (ALD), member 2	17320652	1.444	2.66E-03
Abcg1	ATP-binding cassette, sub-family G (WHITE), member 1	17335770	1.999	3.19E-05
Abracl	ABRA C-terminal like	17548593	1.044	4.29E-03
Acap1	ArfGAP with coiled-coil, ankyrin repeat and PH domains 1	17265030	1.714	5.88E-05
Ackr1	atypical chemokine receptor 1 (Duffy blood group)	17229984	2.935	3.04E-05
Acp5	acid phosphatase 5, tartrate resistant	17524930	1.924	6.03E-03
Adam8	ADAM metallopeptidase domain 8	17497525	1.088	3.73E-02
Adap1	ArfGAP with dual PH domains 1	17454382	1.196	1.15E-02
Adcy7	adenylate cyclase 7	17503616	1.008	1.68E-03
Adgre1	adhesion G protein-coupled receptor E1	17339013	1.327	6.47E-04
Adssl1	adenylosuccinate synthase like 1	17279349	2.014	1.16E-03
Af067061	cDNA sequence AF067061	17290199	-1.364	1.50E-02
Ahnak2	AHNAK nucleoprotein 2	17547719	1.759	3.08E-06
Ai504432	expressed sequence AI504432	17401530	2.419	1.93E-04
Ai662270	expressed sequence AI662270	17254194	1.343	3.49E-03
Aif1L	allograft inflammatory factor 1-like	17369613	-1.023	1.56E-03
Alox12	arachidonate 12-lipoxygenase	17265193	-1.188	8.03E-04
Amica1	adhesion molecule, interacts with CXADR antigen 1	17516718	1.439	1.26E-03
Ano1	anoctamin 1, calcium activated chloride channel	17498467	-1.048	2.41E-02
Anxa1	annexin A1	17363407	1.284	2.18E-03
Anxa6	annexin A6	17262887	1.075	1.79E-04
Aoah	acyloxyacyl hydrolase (neutrophil)	17285586	2.112	3.10E-03
Apln	apelin	17541378	-1.746	1.19E-04
Apobec1	apolipoprotein B mRNA editing enzyme, catalytic polypeptide '	17470580	2.072	6.89E-05
Apoc1	apolipoprotein C-I	17487374	3.839	1.90E-08
Apoc2	apolipoprotein C-II	17487361	2.495	1.05E-03
Apoc4	apolipoprotein C-IV	17487369	4.499	5.87E-08
Apoe	apolipoprotein E	17487381	1.308	1.70E-04
Apol7E (Includes Others)	apolipoprotein L 7e	17312679	1.981	2.40E-03
Aprt	adenine phosphoribosyltransferase	17513771	1.167	2.91E-04
Arhgap15	Rho GTPase activating protein 15	17370551	1.688	9.19E-04
Arhgap25	Rho GTPase activating protein 25	17468612	1.404	8.01E-06
Arhgap9	Rho GTPase activating protein 9	17237984	1.026	6.31E-03
Arl11	ADP-ribosylation factor-like 11	17301108	1.448	9.58E-03
Arl4C	ADP-ribosylation factor-like 4C	17225360	1.257	5.92E-04
Arl5C	ADP-ribosylation factor-like 5C	17268681	1.338	9.02E-03
Arntl	aryl hydrocarbon receptor nuclear translocator-like	17481960	1.144	6.46E-05
Arrdc4	arrestin domain containing 4	17492051	1.402	9.19E-04
Arxes1/Arxes2	adipocyte-related X-chromosome expressed sequence 2	17537861	-1.224	2.39E-03
Aspn	asporin	17287160	-1.067	1.69E-02
Atad2	ATPase family, AAA domain containing 2	17317233	1.064	6.44E-04
Atf3	activating transcription factor 3	17231033	2.375	7.76E-09
Atp1A3	ATPase, Na+/K+ transporting, alpha 3 polypeptide	17487805	2.925	4.24E-06
Atp1B2	ATPase, Na+/K+ transporting, beta 2 polypeptide	17264792	-1.191	1.28E-03
Atp6V0C	ATPase, H+ transporting, lysosomal 16kDa, V0 subunit c	17457343	1.246	2.98E-03
Atp6V0D2	ATPase, H+ transporting, lysosomal 38kDa, V0 subunit d2	17423577	2.705	7.97E-03
Atp6V1B2	ATPase, H+ transporting, lysosomal 56/58kDa, V1 subunit B2	17501652	1.249	4.15E-05
Atp6V1C1	ATPase, H+ transporting, lysosomal 42kDa, V1 subunit C1	17311157	1.234	3.59E-05
Atp8B1	ATPase, aminophospholipid transporter, class I, type 8B, mem	17355026	1.037	2.92E-02
Atp8B4	ATPase, class I, type 8B, member 4	17391056	1.447	1.51E-02
Au020206	expressed sequence AU020206	17492239	1.106	9.39E-04
Au022793	expressed sequence AU022793	17311263	1.069	2.47E-02
Aw549542	expressed sequence AW549542	17451930	-1.140	1.85E-02
B430306N03Rik	RIKEN cDNA B430306N03 gene	17338403	1.487	9.09E-04
B4Galnt1	beta-1,4-N-acetyl-galactosaminyl transferase 1	17237937	1.844	8.69E-06
Bc021614	cDNA sequence BC021614	17361090	1.292	1.42E-02
Bcap31	B-cell receptor-associated protein 31	17542382	1.524	1.87E-04
Bche	butyrylcholinesterase	17405908	-1.503	8.54E-05
Bcl2A1	BCL2-related protein A1	17520162	2.158	1.44E-05
Bcl3	B-cell CLL/lymphoma 3	17487457	1.125	6.77E-04
Bcl6	B-cell CLL/lymphoma 6	17329433	1.140	6.14E-04
Bcl6B	B-cell CLL/lymphoma 6, member B	17265164	-1.257	2.60E-03
Be692007	expressed sequence BE692007	17362966	1.564	1.89E-03
Best1	bestrophin 1	17362579	1.165	8.03E-04
Blnk	B-cell linker	17364565	1.846	1.48E-05
Blvra	biliverdin reductase A	17375767	1.206	1.37E-03
Bmp2K	BMP2 inducible kinase	17439481	1.039	4.47E-04
Borcs6	BLOC-1 related complex subunit 6	17251500	1.106	4.73E-05
Bphl	biphenyl hydrolase-like (serine hydrolase)	17286487	1.229	2.38E-05
Btk	Bruton agammaglobulinemia tyrosine kinase	17544491	1.026	1.58E-03
C12Orf57	chromosome 12 open reading frame 57	17470829	-1.222	1.09E-02
C130026I21Rik (Includes	RIKEN cDNA C130026I21 gene	17366201	2.045	6.00E-05
C15Orf48	chromosome 15 open reading frame 48	17375503	3.863	1.17E-03
C15Orf61	chromosome 15 open reading frame 61	17528079	-1.066	5.36E-03

Supplementary Table 4. Differentially expressed transcripts in vascular fragments between EAE and naive mice at the remission phase

Gene symbol	Gene name	Affymetrix ID	Fold Change	Adj. palue
C17Orf100	chromosome 17 open reading frame 100	17252329	-1.226	1.07E-03
C19Orf38	chromosome 19 open reading frame 38	17515238	2.439	6.00E-05
C19Orf70	chromosome 19 open reading frame 70	17346311	1.192	2.83E-03
C1S	complement component 1, s subcomponent	17462889	1.530	1.29E-02
C2	complement component 2	17344086	1.083	3.27E-03
C230073G13Rik	RIKEN cDNA C230073G13 gene	17339383	1.034	3.43E-02
C3	complement component 3	17346528	3.925	4.24E-06
C3Ar1	complement component 3a receptor 1	17470616	2.047	4.47E-05
C4A/C4B	complement component 4B (Chido blood group)	17343918	2.991	5.39E-06
C5Ar1	complement component 5a receptor 1	17486864	1.541	2.79E-05
C8Orf4	chromosome 8 open reading frame 4	17508170	1.003	2.85E-02
C920009B18Rik	RIKEN cDNA C920009B18 gene	17232112	1.243	4.99E-03
Camk1D	calcium/calmodulin-dependent protein kinase ID	17381357	1.252	7.35E-04
Card11	caspase recruitment domain family, member 11	17454627	1.215	1.81E-02
Casp1	caspase 1, apoptosis-related cysteine peptidase	17514424	1.614	1.17E-04
Casp4	caspase 4, apoptosis-related cysteine peptidase	17514435	1.473	1.13E-04
Casq2	calsequestrin 2 (cardiac muscle)	17401041	-1.116	2.90E-03
Ccdc43	coiled-coil domain containing 43	17270305	1.001	9.66E-04
Ccl17	chemokine (C-C motif) ligand 17	17504138	3.267	3.11E-03
Ccl19	chemokine (C-C motif) ligand 19	17434280	1.615	5.57E-04
Ccl2	chemokine (C-C motif) ligand 2	17254041	2.870	7.31E-05
Ccl22	chemokine (C-C motif) ligand 22	17504122	2.410	9.02E-03
Ccl3L3	chemokine (C-C motif) ligand 3-like 3	17266967	2.357	1.88E-07
Ccl4	chemokine (C-C motif) ligand 4	17254283	2.525	3.44E-07
Ccl5	chemokine (C-C motif) ligand 5	17266946	4.285	9.87E-06
Ccl6	chemokine (C-C motif) ligand 6	17266960	2.135	4.25E-04
Ccl8	chemokine (C-C motif) ligand 8	17254065	2.255	3.98E-04
Ccr2	chemokine (C-C motif) receptor 2	17523650	2.798	3.69E-05
Ccr8	chemokine (C-C motif) receptor 8	17523158	1.216	4.94E-03
Ccrl2	chemokine (C-C motif) receptor-like 2	17531705	1.778	4.44E-05
Cd14	CD14 molecule	17353747	1.850	2.23E-06
Cd2	CD2 molecule	17408497	2.716	1.93E-04
Cd200R1L	CD200 receptor 1-like	17325874	1.882	2.73E-03
Cd22	CD22 molecule	17489320	2.769	1.90E-08
Cd226	CD226 molecule	17352330	2.178	1.92E-05
Cd248	CD248 molecule, endosialin	17356369	-1.604	2.16E-05
Cd28	CD28 molecule	17213462	1.386	9.60E-03
Cd300C	CD300c molecule	17271733	1.438	2.79E-04
Cd300Ld	CD300 molecule-like family member d	17271751	1.358	2.18E-03
Cd300Lf	CD300 molecule-like family member f	17271776	1.970	6.55E-03
Cd33	CD33 antigen	17490149	1.177	5.98E-03
Cd36	CD36 molecule (thrombospondin receptor)	17445715	2.502	3.75E-03
Cd3D	CD3d molecule, delta (CD3-TCR complex)	17516691	2.403	5.34E-04
Cd3E	CD3e molecule, epsilon (CD3-TCR complex)	17526464	3.319	1.25E-05
Cd3G	CD3g molecule, gamma (CD3-TCR complex)	17526456	3.952	2.55E-05
Cd4	CD4 molecule	17470960	1.296	9.90E-03
Cd40Lg	CD40 ligand	17535048	1.494	8.94E-03
Cd44	CD44 molecule (Indian blood group)	17388733	1.668	5.61E-04
Cd48	CD48 molecule	17219397	2.515	1.20E-07
Cd5	CD5 molecule	17362753	1.855	8.03E-04
Cd52	CD52 antigen	17431174	1.480	4.57E-05
Cd53	CD53 molecule	17408960	1.392	5.75E-05
Cd63	CD63 molecule	17238594	1.498	2.72E-07
Cd68	CD68 molecule	17264835	1.671	2.54E-06
Cd69	CD69 molecule	17471502	2.371	2.08E-04
Cd72	CD72 molecule	17424608	2.281	9.85E-05
Cd74	CD74 molecule, major histocompatibility complex, class II inva	17350982	3.005	3.97E-07
Cd83	CD83 molecule	17286905	1.081	6.44E-04
Cd84	CD84 molecule	17219418	1.372	3.93E-04
Cd86	CD86 molecule	17330203	1.504	1.69E-04
Cd8B	CD8b molecule	17459474	1.153	1.72E-02
Cd96	CD96 molecule	17330751	1.806	2.18E-03
Cdh19	cadherin 19, type 2	17226127	1.498	1.12E-02
Cdk1	cyclin-dependent kinase 1	17241692	1.317	1.18E-02
Cdkn2B	cyclin-dependent kinase inhibitor 2B (p15, inhibits CDK4)	17427155	-1.032	4.79E-04
Cdo1	cysteine dioxygenase type 1	17354282	2.014	1.05E-07
Cebpa	CCAAT/enhancer binding protein (C/EBP), alpha	17476728	1.213	7.31E-05
Cebpb	CCAAT/enhancer binding protein (C/EBP), beta	17379938	1.041	2.98E-04
Cers6	ceramide synthase 6	17371374	1.938	8.26E-04
Ces2G	carboxylesterase 2G	17504572	-1.176	1.19E-02
Cfb	complement factor B	17344064	2.198	2.63E-04
Ch25H	cholesterol 25-hydroxylase	17364111	2.097	7.29E-06
Chd7	chromodomain helicase DNA binding protein 7	17411647	1.070	1.55E-04
Chil3/Chil4	chitinase-like 3	17408897	2.496	1.43E-02
Chst1	carbohydrate (keratan sulfate Gal-6) sulfotransferase 1	17373521	1.010	7.77E-03
Ciart	circadian associated repressor of transcription	17407934	-2.011	1.67E-06
Clca3A1/Clca3A2	chloride channel accessory 3A1	17410863	1.809	1.82E-03

Supplementary Table 4. Differentially expressed transcripts in vascular fragments between EAE and naive mice at the remission phase

Gene symbol	Gene name	Affymetrix ID	Fold Change	Adj. palue
Cldn1	claudin 1	17329479	1.507	2.67E-03
Clec12A	C-type lectin domain family 12, member A	17463509	1.748	2.60E-02
Clec4A3	C-type lectin domain family 4, member a3	17462738	2.350	1.50E-05
Clec4E	C-type lectin domain family 4, member E	17470627	2.015	4.91E-02
Clec4M	C-type lectin domain family 4, member M	17507161	1.388	5.98E-03
Clec5A	C-type lectin domain family 5, member A	17466228	1.404	1.99E-03
Clec7A	C-type lectin domain family 7, member A	17471541	3.915	2.23E-06
Clec9A	C-type lectin domain family 9, member A	17463530	1.263	3.42E-02
Cln3	ceroid-lipofuscinosis, neuronal 3	17496310	1.026	5.42E-03
Cmtm3	CKLF-like MARVEL transmembrane domain containing 3	17504444	1.050	2.56E-04
Cndp2	CNDP dipeptidase 2 (metallopeptidase M20 family)	17355825	1.150	1.05E-03
Cntn1	contactin 1	17314387	-1.187	1.34E-02
Colec12	collectin sub-family member 12	17348282	1.152	3.79E-04
Colgalt2	collagen beta(1-O)galactosyltransferase 2	17218233	-1.038	6.40E-03
Coro1A	coronin, actin binding protein, 1A	17496376	1.199	1.14E-04
Cotl1	coactosin-like F-actin binding protein 1	17513491	1.837	4.47E-05
Cox6A2	cytochrome c oxidase subunit VIa polypeptide 2	17496857	1.732	1.68E-03
Cpa3	carboxypeptidase A3 (mast cell)	17404337	1.020	9.93E-03
Creg1	cellular repressor of E1A-stimulated genes 1	17219005	1.112	4.70E-04
Cryba4	crystallin, beta A4	17451195	1.138	2.79E-02
Csf2Rb	colony stimulating factor 2 receptor, beta, low-affinity (granuloc	17318950	2.715	8.08E-06
Cspg4	chondroitin sulfate proteoglycan 4	17517592	-1.319	9.78E-05
Cst7	cystatin F (leukocystatin)	17377464	4.600	1.61E-08
Cstb	cystatin B (stefin B)	17234803	1.028	1.21E-02
Cthrc1	collagen triple helix repeat containing 1	17311191	-1.284	2.18E-04
Ctla4	cytotoxic T-lymphocyte-associated protein 4	17213478	1.219	1.26E-02
Ctns	cystinosin, lysosomal cystine transporter	17265733	1.026	3.27E-04
Ctsb	cathepsin B	17301213	1.715	2.19E-07
Ctsc	cathepsin C	17480018	1.568	3.40E-05
Ctse	cathepsin E	17217035	2.436	9.69E-06
Ctsw	cathepsin W	17361605	2.112	3.72E-05
Ctsz	cathepsin Z	17395155	1.899	7.64E-07
Cxcl10	chemokine (C-X-C motif) ligand 10	17449718	3.296	3.90E-05
Cxcl13	chemokine (C-X-C motif) ligand 13	17439367	2.521	5.26E-05
Cxcl16	chemokine (C-X-C motif) ligand 16	17265268	2.425	3.43E-06
Cxcl9	chemokine (C-X-C motif) ligand 9	17449710	4.314	2.97E-06
Cxcr3	chemokine (C-X-C motif) receptor 3	17543625	1.006	2.29E-02
Cxcr4	chemokine (C-X-C motif) receptor 4	17226593	2.195	5.69E-05
Cxcr6	chemokine (C-X-C motif) receptor 6	17523642	2.944	4.11E-04
Cxorf21	chromosome X open reading frame 21	17536191	1.363	1.87E-04
Cyba	cytochrome b-245, alpha polypeptide	17513672	1.404	4.03E-04
Cybb	cytochrome b-245, beta polypeptide	17540154	3.383	2.40E-06
Cycs	cytochrome c, somatic	17548541	1.177	2.75E-02
Cyfip2	cytoplasmic FMR1 interacting protein 2	17262065	1.057	1.21E-02
Cygb	cytoglobin	17272461	-1.213	2.97E-03
Cyp27A1	cytochrome P450, family 27, subfamily A, polypeptide 1	17214368	1.246	1.14E-03
Cyp2E1	cytochrome P450, family 2, subfamily E, polypeptide 1	17484587	-1.199	7.84E-04
Cyp4F2	cytochrome P450, family 4, subfamily F, polypeptide 2	17510462	1.322	9.69E-03
Cyr61	cysteine-rich, angiogenic inducer, 61	17410974	-1.164	1.31E-02
Cytip	cytohesin 1 interacting protein	17385405	1.912	3.83E-04
Dapp1	dual adaptor of phosphotyrosine and 3-phosphoinositides	17410617	1.225	2.71E-04
Dazap2	DAZ associated protein 2	17315045	1.083	1.67E-03
Dbp	D site of albumin promoter (albumin D-box) binding protein	17477979	-1.767	2.08E-06
Dclre1C	DNA cross-link repair 1C	17366399	1.210	2.39E-04
Dcn	decorin	17236800	1.327	1.95E-04
Ddhd1	DDHD domain containing 1	17305685	1.158	5.33E-04
Dguok	deoxyguanosine kinase	17468172	1.088	8.81E-04
Dhrs3	dehydrogenase/reductase (SDR family) member 3	17421312	1.472	1.96E-06
Dna2	DNA replication helicase/nuclease 2	17233811	1.382	4.18E-04
Dock11	dedicator of cytokinesis 11	17533994	1.376	7.76E-05
Dock2	dedicator of cytokinesis 2	17261650	1.735	8.76E-06
Donson	downstream neighbor of SON	17332166	1.026	3.10E-03
Dpp7	dipeptidyl-peptidase 7	17382533	1.383	8.83E-07
Dusp2	dual specificity phosphatase 2	17375859	1.014	6.44E-04
E230013L22Rik	RIKEN cDNA E230013L22 gene	17499011	1.196	1.14E-02
Ebi3	Epstein-Barr virus induced 3	17338642	1.014	1.02E-03
Efhd2	EF-hand domain family, member D2	17432341	1.082	5.61E-04
Egln3	egl-9 family hypoxia-inducible factor 3	17281084	1.755	1.42E-02
Egr2	early growth response 2	17233993	1.050	2.95E-03
Eid3	EP300 interacting inhibitor of differentiation 3	17235937	1.078	2.17E-03
Eif5	eukaryotic translation initiation factor 5	17279167	1.323	1.43E-02
Elf4	E74-like factor 4 (ets domain transcription factor)	17541404	1.849	9.69E-06
Eln	elastin	17453454	-1.220	5.25E-03
Emb	embigin	17290083	3.201	6.46E-05
Emilin2	elastin microfibril interfacer 2	17346975	1.247	1.10E-02
Enho	energy homeostasis associated	17424276	-1.179	5.88E-05
Enpp1	ectonucleotide pyrophosphatase/phosphodiesterase 1	17239787	1.030	5.96E-03

Supplementary Table 4. Differentially expressed transcripts in vascular fragments between EAE and naive mice at the remission phase

Gene symbol	Gene name	Affymetrix ID	Fold Change	Adj. palue
Epb41L3	erythrocyte membrane protein band 4.1-like 3	17339313	1.833	2.69E-05
Ephx3	epoxide hydrolase 3	17343334	-1.746	4.43E-06
Epsti1	epithelial stromal interaction 1 (breast)	17302141	2.254	1.44E-05
Ero1B	endoplasmic reticulum oxidoreductase beta	17285204	1.083	5.40E-04
Etv6	ets variant 6	17463673	1.276	1.95E-04
Evi2A	ecotropic viral integration site 2A	17266590	1.172	9.13E-04
Eya4	EYA transcriptional coactivator and phosphatase 4	17239719	1.182	2.43E-03
F13A1	coagulation factor XIII, A1 polypeptide	17291881	1.072	4.64E-02
F630111L10Rik	RIKEN cDNA F630111L10 gene	17405469	1.118	1.70E-02
F830016B08Rik	RIKEN cDNA F830016B08 gene	17350921	1.497	1.15E-02
F9	coagulation factor IX	17535098	1.460	2.04E-04
Fabp4	fatty acid binding protein 4, adipocyte	17404091	2.263	3.84E-02
Fabp5	fatty acid binding protein 5 (psoriasis-associated)	17548717	2.931	1.54E-05
Fam105A	family with sequence similarity 105, member A	17316197	1.014	7.56E-03
Fam20C	family with sequence similarity 20, member C	17443901	1.721	2.13E-04
Fam26F	family with sequence similarity 26, member F	17240186	1.680	1.38E-03
Fam46C	family with sequence similarity 46, member C	17408414	1.674	1.25E-04
Fam50A	family with sequence similarity 50, member A	17535826	1.034	3.04E-05
Fcgr1A	Fc fragment of IgG, high affinity Ia, receptor (CD64)	17408024	1.300	9.25E-04
Fcgr2A	Fc fragment of IgG, low affinity IIa, receptor (CD32)	17229620	1.338	6.00E-05
Fcgr2B	Fc fragment of IgG, low affinity IIb, receptor (CD32)	17229607	1.781	1.19E-04
Fcgr3A/Fcgr3B	Fc fragment of IgG, low affinity IIIa, receptor (CD16a)	17219199	3.381	2.72E-07
Fermt3	fermitin family member 3	17362223	1.131	8.39E-03
Fgl2	fibrinogen-like 2	17435089	1.610	8.72E-04
Fgr	FGR proto-oncogene, Src family tyrosine kinase	17419483	1.194	3.40E-02
Fjx1	four jointed box 1	17388725	-1.007	1.57E-02
Fkbp5	FK506 binding protein 5	17342868	1.096	1.35E-03
Flrt2	fibronectin leucine rich transmembrane protein 2	17277788	-1.066	1.42E-04
Flt3	fms-related tyrosine kinase 3	17455319	1.097	2.05E-02
Fmn1	formin 1	17374332	1.293	4.56E-05
Frrs1	ferric-chelate reductase 1	17402072	1.555	1.05E-03
Fyb	FYN binding protein	17309981	1.558	1.44E-05
Gab2	GRB2-associated binding protein 2	17480312	1.086	3.67E-05
Gabrg1	gamma-aminobutyric acid (GABA) A receptor, gamma 1	17448565	-1.137	6.20E-03
Gadd45B	growth arrest and DNA-damage-inducible, beta	17235511	1.515	1.18E-04
Gadd45G	growth arrest and DNA-damage-inducible, gamma	17287361	1.223	7.94E-04
Galr3	galanin receptor 3	17312899	-1.057	2.59E-02
Gapdh	glyceraldehyde-3-phosphate dehydrogenase	17221432	1.045	3.14E-02
Gart	phosphoribosylglycinamide formyltransferase, phosphoribosylg	17547960	1.003	4.59E-02
Gas2L3	growth arrest-specific 2 like 3	17244057	1.047	3.38E-02
Gbp8	guanylate-binding protein 8	17450434	2.018	2.30E-02
Gda	guanine deaminase	17363470	1.108	2.56E-02
Gimap1-Gimap5	GIMAP1-GIMAP5 readthrough	17466618	1.841	4.18E-04
Gimap4	GTPase, IMAP family member 4	17458362	1.198	4.22E-05
Gimap7	GTPase, IMAP family member 7	17458372	1.531	2.64E-03
Gipc3	GIPC PDZ domain containing family, member 3	17243278	-1.124	3.48E-04
Gja1	gap junction protein, alpha 1, 43kDa	17233347	-1.161	1.43E-02
Gk	glycerol kinase	17543045	1.141	5.20E-04
Gla	galactosidase, alpha	17544517	1.189	9.80E-03
Glce	glucuronic acid epimerase	17527982	-1.366	7.49E-03
Glipr1	GLI pathogenesis-related 1	17244949	1.362	6.24E-05
Glycam1	glycosylation dependent cell adhesion molecule 1	17322437	2.213	1.93E-05
Gm10036	ribosomal protein L11 pseudogene	17348674	1.757	8.63E-04
Gm10192	predicted gene 10192	17427441	-1.017	2.78E-04
Gm10308	predicted gene 10308	17340393	-1.313	8.77E-05
Gm10921 (Includes Other	predicted gene 14374	17540902	-1.217	4.33E-03
Gm12250	predicted gene 12250	17249977	1.977	4.21E-03
Gm12474	predicted gene 12474	17400994	1.206	1.93E-02
Gm13710	predicted gene 13710	17387426	1.613	3.23E-03
Gm14023	predicted gene 14023	17376153	1.231	5.86E-04
Gm14055	predicted gene 14055	17376856	-1.021	1.64E-03
Gm16336	predicted gene 16336	17481797	-1.030	7.58E-05
Gm19585	predicted gene, 19585	17338540	1.625	6.27E-03
Gm21115 (Includes Other	predicted gene 9271	17489965	-1.002	5.42E-03
Gm3383 (Includes Others)	predicted gene 3383	17303018	-1.136	3.67E-03
Gm4841	predicted gene 4841	17354589	1.199	1.66E-02
Gm4951	predicted gene 4951	17350916	2.131	3.59E-03
Gm5086	predicted gene 5086	17289388	1.369	3.00E-03
Gm5547	predicted gene 5547	17401414	1.169	2.59E-04
Gm5908	predicted gene 5908	17508626	2.153	5.29E-05
Gmnn	geminin, DNA replication inhibitor	17291343	1.019	1.09E-02
Gnb2L1	guanine nucleotide binding protein (G protein), beta polypeptid	17262174	1.117	6.15E-06
Gnptab	N-acetylglucosamine-1-phosphate transferase, alpha and beta	17243884	1.112	2.14E-03
Gns	glucosamine (N-acetyl)-6-sulfatase	17237715	1.064	6.14E-04
Gpnmb	glycoprotein (transmembrane) nmb	17458439	6.261	9.63E-07
Gpr137B-Ps	G protein-coupled receptor 137B, pseudogene	17290655	1.201	4.29E-03
Gpr165	G protein-coupled receptor 165	17536420	-2.668	2.72E-07

Supplementary Table 4. Differentially expressed transcripts in vascular fragments between EAE and naive mice at the remission phase

Gene symbol	Gene name	Affymetrix ID	Fold Change	Adj. palue
Gpr171	G protein-coupled receptor 171	17405458	1.841	5.69E-03
Gpr18	G protein-coupled receptor 18	17309644	1.456	7.36E-03
Gpr65	G protein-coupled receptor 65	17277794	2.555	3.43E-08
Gpr84	G protein-coupled receptor 84	17322355	1.187	2.13E-03
Gpx3	glutathione peroxidase 3	17249787	1.231	5.44E-03
Grap2	GRB2-related adaptor protein 2	17313147	1.048	4.31E-03
Grina	glutamate receptor, ionotropic, N-methyl D-aspartate-associate	17312341	1.233	1.02E-03
Grm3	glutamate receptor, metabotropic 3	17445596	-1.119	1.87E-02
Gsap	gamma-secretase activating protein	17435055	1.576	1.74E-03
Gsdmc	gasdermin C	17317472	-1.047	2.36E-03
Gsto1	glutathione S-transferase omega 1	17360216	1.768	1.21E-05
Gusb	glucuronidase, beta	17453160	1.330	3.90E-05
Gvin1 (Includes Others)	GTPase, very large interferon inducible 1	17494677	2.210	1.40E-04
H2-K2/H2-Q9	histocompatibility 2, K region locus 2	17337133	1.102	1.74E-02
H2-M2	histocompatibility 2, M region locus 2	17344873	1.130	5.20E-03
H2-Q5	histocompatibility 2, Q region locus 5	17337110	1.985	5.29E-05
H2-T22	histocompatibility 2, T region locus 22	17344593	1.639	1.27E-02
Hamp/Hamp2	hepcidin antimicrobial peptide	17489363	1.888	1.61E-02
Hcar1	hydroxycarboxylic acid receptor 1	17452709	-1.450	9.11E-06
Hcar2	hydroxycarboxylic acid receptor 2	17452705	3.585	2.19E-07
Hck	HCK proto-oncogene, Src family tyrosine kinase	17377870	1.219	1.10E-03
Hcls1	hematopoietic cell-specific Lyn substrate 1	17325438	1.273	1.37E-04
Hcst	hematopoietic cell signal transducer	17489046	2.454	5.29E-05
Hebp1	heme binding protein 1	17472063	1.322	1.94E-03
Hexa	hexosaminidase A (alpha polypeptide)	17517947	1.038	1.56E-03
Heyl	hes-related family bHLH transcription factor with YRPW motif-l	17418177	-1.058	1.06E-02
Hist1H1B	histone cluster 1, H1b	17291005	1.861	1.47E-02
Hist1H2Aa	histone cluster 1, H2aa	17285938	-1.123	1.61E-03
Hla-A	major histocompatibility complex, class I, A	17337122	1.519	2.88E-03
Hla-Dma	major histocompatibility complex, class II, DM alpha	17336407	1.030	6.05E-03
Hla-Dmb	major histocompatibility complex, class II, DM beta	17336414	1.917	1.81E-03
Hla-Doa	major histocompatibility complex, class II, DO alpha	17336396	1.685	2.30E-03
Hla-Dob	major histocompatibility complex, class II, DO beta	17336476	1.115	2.19E-04
Hla-Dqa1	major histocompatibility complex, class II, DQ alpha 1	17343813	3.761	3.31E-07
Hla-Dqb1	major histocompatibility complex, class II, DQ beta 1	17336494	3.697	7.62E-08
Hla-Drb5	major histocompatibility complex, class II, DR beta 5	17336502	3.055	9.59E-06
Hlf	hepatic leukemia factor	17267702	-1.626	1.53E-05
Hmcn1	hemicentin 1	17227910	-1.574	1.08E-03
Hmox1	heme oxygenase 1	17502573	1.377	2.90E-03
Hp	haptoglobin	17512809	1.373	2.48E-02
Hpse	heparanase	17548411	4.046	4.08E-07
Hspa1A/Hspa1B	heat shock 70kDa protein 1A	17344132	1.458	5.00E-02
Hspa1B	heat shock protein 1B	17344126	1.589	3.94E-02
Hspa4L	heat shock 70kDa protein 4-like	17397240	1.029	8.73E-04
Hspa8	heat shock 70kDa protein 8	17516383	1.536	3.69E-03
Hspe1	heat shock 10kDa protein 1	17212882	1.069	3.62E-04
Hsph1	heat shock 105kDa/110kDa protein 1	17455507	1.220	8.41E-03
Hvcn1	hydrogen voltage gated channel 1	17442046	1.200	1.87E-03
Icos	inducible T-cell co-stimulator	17213490	1.528	2.51E-02
Id2	inhibitor of DNA binding 2, dominant negative helix-loop-helix p	17280310	1.981	8.79E-06
Ier3	immediate early response 3	17337228	1.202	1.24E-03
Ifi16	interferon, gamma-inducible protein 16	17230045	4.036	2.14E-07
Ifi27L2A/Ifi27L2B	interferon, alpha-inducible protein 27 like 2A	17283549	1.566	4.53E-03
Ifi30	interferon, gamma-inducible protein 30	17510136	2.155	1.40E-05
Ifit1	interferon-induced protein with tetratricopeptide repeats 1	17364126	1.320	2.96E-02
Igf1	insulin-like growth factor 1 (somatomedin C)	17236288	2.926	6.27E-07
Ighg	Immunoglobulin heavy chain (gamma polypeptide)	17284334	3.279	2.52E-03
Ighg2C	immunoglobulin heavy constant gamma 2C	17284327	1.141	2.64E-02
Ighg3	Immunoglobulin heavy constant gamma 3	17284614	-1.237	1.31E-02
Ighm	immunoglobulin heavy constant mu	17284339	2.831	2.29E-03
Igk	immunoglobulin kappa chain complex	17459338	2.240	1.73E-03
Iglv1	immunoglobulin lambda variable 1	17329009	1.142	4.62E-02
Igsf6	immunoglobulin superfamily, member 6	17495839	1.873	7.90E-04
Ikbke	inhibitor of kappa light polypeptide gene enhancer in B-cells, ki	17226771	1.426	9.51E-04
Il12Rb2	interleukin 12 receptor, beta 2	17467323	1.018	1.62E-02
Il18R1	interleukin 18 receptor 1	17212229	1.837	1.51E-03
Il18Rap	interleukin 18 receptor accessory protein	17212252	1.062	2.17E-02
Il1A	interleukin 1, alpha	17391554	1.220	4.31E-04
Il1B	interleukin 1, beta	17391565	4.254	5.84E-08
Il1Rn	interleukin 1 receptor antagonist	17367686	1.671	4.33E-02
Il21R	interleukin 21 receptor	17482958	1.013	1.33E-02
Il2Ra	interleukin 2 receptor, alpha	17366992	1.640	5.82E-03
Il2Rb	interleukin 2 receptor, beta	17319009	2.378	2.19E-03
Il4R	interleukin 4 receptor	17482943	1.105	9.63E-04
Inpp5D	inositol polyphosphate-5-phosphatase D	17215309	1.720	3.04E-05
Iqgap2	IQ motif containing GTPase activating protein 2	17295136	1.382	4.18E-04
Irf5	interferon regulatory factor 5	17456692	1.087	1.32E-03

Supplementary Table 4. Differentially expressed transcripts in vascular fragments between EAE and naive mice at the remission phase

Gene symbol	Gene name	Affymetrix ID	Fold Change	Adj. palue
Irf8	interferon regulatory factor 8	17506279	1.121	1.88E-03
Itgae	integrin, alpha E (antigen CD103, human mucosal lymphocyte	17252574	1.795	1.86E-03
Itgal	integrin, alpha L (antigen CD11A (p180), lymphocyte function-a	17483264	2.073	1.22E-04
Itgax	integrin, alpha X (complement component 3 receptor 4 subunit	17483615	4.002	3.60E-07
Itgb2	integrin, beta 2 (complement component 3 receptor 3 and 4 su	17234647	1.945	3.04E-05
Itgb7	integrin, beta 7	17322163	1.676	9.15E-04
Itih4	inter-alpha-trypsin inhibitor heavy chain family, member 4	17298267	2.547	1.07E-03
Itk	IL2-inducible T-cell kinase	17262102	1.368	2.66E-03
Jchain	joining chain of multimeric IgA and IgM	17449447	1.394	8.71E-03
Kcna3	potassium channel, voltage gated shaker related subfamily A,	17401526	1.011	4.33E-02
Kcnj10	potassium channel, inwardly rectifying subfamily J, member 1C	17219536	-1.457	1.73E-04
Kcnj12	potassium channel, inwardly rectifying subfamily J, member 12	17250549	-1.196	9.85E-05
Kif1A	kinesin family member 1A	17225630	1.054	3.84E-02
Kifc3	kinesin family member C3	17511927	-1.238	8.77E-05
Kir3Dl3	killer cell immunoglobulin-like receptor, three domains, long cy	17537906	-1.059	2.36E-02
Klk3	kallikrein-related peptidase 3	17477391	1.566	1.91E-02
Klra2	killer cell lectin-like receptor, subfamily A, member 2	17471828	1.411	2.79E-02
Klrb1	killer cell lectin-like receptor subfamily B, member 1	17471464	3.674	1.30E-05
Klrc1	killer cell lectin-like receptor subfamily C, member 1	17471598	1.514	9.66E-03
Klrd1	killer cell lectin-like receptor subfamily D, member 1	17463567	1.787	5.65E-03
Klrk1	killer cell lectin-like receptor subfamily K, member 1	17471565	1.418	1.90E-02
Kmo	kynurenine 3-monooxygenase (kynurenine 3-hydroxylase)	17219789	1.758	1.44E-03
Krtap10-7	keratin associated protein 10-7	17242322	-1.128	1.21E-02
Krtap5-1	keratin associated protein 5-1	17498084	-1.108	1.68E-02
Lacc1	laccase (multicopper oxidoreductase) domain containing 1	17308772	1.057	5.91E-03
Lag3	lymphocyte-activation gene 3	17470976	1.334	3.30E-05
Lao1	L-amino acid oxidase 1	17417826	1.263	8.86E-04
Lap3	leucine aminopeptidase 3	17437247	1.005	4.43E-04
Lat	linker for activation of T cells	17496150	2.659	2.56E-04
Lck	LCK proto-oncogene, Src family tyrosine kinase	17430413	1.801	6.23E-04
Lcn2	lipocalin 2	17383892	2.340	2.39E-04
Lcp1	lymphocyte cytosolic protein 1 (L-plastin)	17301968	1.429	2.97E-06
Lgals1	lectin, galactoside-binding, soluble, 1	17312829	1.634	1.02E-04
Lgals3	lectin, galactoside-binding, soluble, 3	17299329	2.420	9.48E-05
Lgals3Bp	lectin, galactoside-binding, soluble, 3 binding protein	17272785	1.448	3.66E-05
Lgmn	legumain	17283445	1.100	1.70E-04
Lilrb3	leukocyte immunoglobulin-like receptor, subfamily B (with TM a	17485589	1.663	2.11E-03
Lilrb4	leukocyte immunoglobulin-like receptor, subfamily B (with TM a	17233226	4.848	1.90E-08
Lpl	lipoprotein lipase	17501633	3.372	1.90E-08
Lpxn	leupaxin	17357872	1.301	1.87E-03
Lrg1	leucine-rich alpha-2-glycoprotein 1	17346150	1.887	2.94E-04
Lrguk	leucine-rich repeats and guanylate kinase domain containing	17457092	1.191	1.58E-05
Lrp12	low density lipoprotein receptor-related protein 12	17316754	1.249	2.75E-04
Lrrc25	leucine rich repeat containing 25	17501989	1.283	4.04E-03
Lsamp	limbic system-associated membrane protein	17325671	-1.820	2.16E-04
Lsp1	lymphocyte-specific protein 1	17485226	1.269	1.32E-04
Lst1	leukocyte specific transcript 1	17344303	1.028	5.03E-03
Ltb	lymphotoxin beta (TNF superfamily, member 3)	17337024	2.271	7.82E-05
Lurap1L	leucine rich adaptor protein 1-like	17414984	-1.100	1.21E-04
Ly9	lymphocyte antigen 9	17229767	3.137	1.96E-06
Lynx1	Ly6/neurotoxin 1	17318013	-1.069	9.70E-04
Lyz	lysozyme	17245223	2.000	3.31E-07
Mafb	v-maf avian musculoaponeurotic fibrosarcoma oncogene homo	17393868	1.556	2.63E-05
Map3K7Cl	Map3k7 C-terminal like	17326887	-1.088	5.84E-03
Map3K8	mitogen-activated protein kinase kinase kinase 8	17352401	1.067	1.58E-03
Map4K1	mitogen-activated protein kinase kinase kinase kinase 1	17476036	1.290	6.39E-03
Mapkapk2	mitogen-activated protein kinase-activated protein kinase 2	17226736	1.117	1.13E-05
Matn2	matrilin 2	17310922	1.275	1.18E-02
Mboat2	membrane bound O-acyltransferase domain containing 2	17274558	-1.083	5.42E-03
Mcemp1	mast cell-expressed membrane protein 1	17498730	2.508	7.78E-04
Mcf2L	MCF.2 cell line derived transforming sequence-like	17499155	-1.194	3.72E-04
Mcpt2	mast cell protease 2	17300725	1.706	2.97E-02
Mctp1	multiple C2 domains, transmembrane 1	17288780	1.155	5.61E-06
Med11	mediator complex subunit 11	17252032	1.474	2.06E-04
Mettl23	methyltransferase like 23	17258653	1.040	1.48E-03
Mgat5	mannosyl (alpha-1,6-)-glycoprotein beta-1,6-N-acetyl-glucosan	17216753	1.030	1.66E-04
Mif	macrophage migration inhibitory factor (glycosylation-inhibiting	17241962	1.213	1.31E-04
Milr1	mast cell immunoglobulin like receptor 1	17257599	1.549	9.98E-04
Mir-10	microRNA 100	17333705	-1.414	2.52E-04
Mir-149	microRNA 149	17215832	-1.014	6.72E-03
Mir-34	microRNA 34a	17526980	-1.001	1.51E-03
Mir143Hg	Mir143 and Mir145 host gene (non-protein coding)	17354784	-1.029	9.12E-03
Mirg	miRNA containing gene	17278854	-1.162	2.94E-03
Mitf	microphthalmia-associated transcription factor	17461132	1.063	1.28E-02
Mixl1	Mix paired-like homeobox	17230463	-1.082	1.55E-04
Mlc1	megalencephalic leukoencephalopathy with subcortical cysts 1	17320225	-1.059	4.17E-02
Mmp14	matrix metallopeptidase 14 (membrane-inserted)	17300279	1.644	6.00E-04

Supplementary Table 4. Differentially expressed transcripts in vascular fragments between EAE and naive mice at the remission phase

Gene symbol	Gene name	Affymetrix ID	Fold Change	Adj. palue
Mmp8	matrix metallopeptidase 8	17514553	1.284	3.50E-02
Mpeg1	macrophage expressed 1	17357810	1.538	3.11E-04
Mpp6	membrane protein, palmitoylated 6 (MAGUK p55 subfamily me	17458520	1.387	1.92E-03
Mrps15	mitochondrial ribosomal protein S15	17418507	1.168	2.15E-04
Mrps16	mitochondrial ribosomal protein S16	17303765	1.699	1.65E-03
Ms4A4A	membrane-spanning 4-domains, subfamily A, member 4A	17357640	1.237	2.06E-02
Ms4A4B (Includes Others)	membrane-spanning 4-domains, subfamily A, member 4B	17357659	2.039	1.96E-04
Ms4A6A	membrane-spanning 4-domains, subfamily A, member 6A	17362973	1.670	8.05E-04
Ms4A6B	membrane-spanning 4-domains, subfamily A, member 6B	17357688	1.278	1.62E-03
Ms4A6C	membrane-spanning 4-domains, subfamily A, member 6C	17357671	2.785	1.60E-06
Ms4A7	membrane-spanning 4-domains, subfamily A, member 7	17362953	3.233	4.15E-04
Msr1	macrophage scavenger receptor 1	17508850	2.710	2.67E-04
Myc	v-myc avian myelocytomatosis viral oncogene homolog	17311846	1.267	8.77E-05
Myo1D	myosin ID	17266698	-1.064	3.82E-04
Myo1F	myosin IF	17336114	1.479	7.23E-04
Myo5A	myosin VA	17519394	3.354	5.86E-08
N-R5S136	nuclear encoded rRNA 5S 136	17513976	-1.177	3.75E-02
N4Bp2L1	NEDD4 binding protein 2-like 1	17455554	1.013	2.17E-03
Naaa	N-acylethanolamine acid amidase	17449673	1.744	9.25E-04
Nabp1	nucleic acid binding protein 1	17222825	1.526	8.03E-04
Naglu	N-acetylglucosaminidase, alpha	17256502	1.571	6.77E-04
Naip	NLR family, apoptosis inhibitory protein	17295569	1.255	1.08E-02
Naip1 (Includes Others)	NLR family, apoptosis inhibitory protein 1	17295607	1.473	1.20E-02
Napsa	napsin A aspartic peptidase	17477508	2.315	7.76E-06
Ncan	neurocan	17509944	-1.018	2.75E-03
Ncf1	neutrophil cytosolic factor 1	17453288	1.062	2.40E-03
Ncf2	neutrophil cytosolic factor 2	17218261	1.350	2.57E-05
Ndc80	NDC80 kinetochore complex component	17347042	1.201	1.63E-02
Ndrg2	NDRG family member 2	17306147	-1.233	1.22E-03
Ndufa4L2	NADH dehydrogenase (ubiquinone) 1 alpha subcomplex, 4-like	17238054	-1.143	2.76E-03
Neurl3	neuralized E3 ubiquitin protein ligase 3	17222149	1.605	3.51E-05
Nfam1	NFAT activating protein with ITAM motif 1	17319738	1.303	4.45E-03
Nfil3	nuclear factor, interleukin 3 regulated	17292634	1.785	3.56E-05
Nfkbiz	nuclear factor of kappa light polypeptide gene enhancer in B-c	17330967	1.254	2.23E-06
Nkg7	natural killer cell granule protein 7	17477101	1.512	8.83E-03
Nlrp3	NLR family, pyrin domain containing 3	17250249	1.520	1.58E-04
Notch3	notch 3	17343299	-1.287	4.79E-04
Npl	N-acetylneuraminate pyruvate lyase (dihydrodipicolinate synth	17228234	1.600	6.72E-04
Nptx2	neuronal pentraxin II	17444498	-1.013	1.29E-04
Nr1D1	nuclear receptor subfamily 1, group D, member 1	17268884	-2.485	3.31E-07
Nt5E	5'-nucleotidase, ecto (CD73)	17520073	1.848	1.35E-04
Ntsr2	neurotensin receptor 2	17274195	-1.731	1.17E-03
Nuak2	NUAK family, SNF1-like kinase, 2	17217182	1.365	1.12E-03
Oas1	2'-5'-oligoadenylate synthetase 1, 40/46kDa	17452115	2.087	7.26E-03
Oas2	2'-5'-oligoadenylate synthetase 2, 69/71kDa	17452054	1.374	1.02E-03
Olfr1129/Olfr1130	olfactory receptor 1130	17372870	-1.025	5.48E-04
Olfr338	olfactory receptor 338	17370326	-1.169	2.39E-03
Olfr99	olfactory receptor 99	17344860	1.337	4.62E-04
Olig1	oligodendrocyte transcription factor 1	17327035	-1.215	2.33E-04
Or10A6	olfactory receptor, family 10, subfamily A, member 6 (gene/pse	17494820	-1.008	2.60E-02
Or2At4	olfactory receptor, family 2, subfamily AT, member 4	17480596	-1.175	8.15E-03
Or4L1	olfactory receptor, family 4, subfamily L, member 1	17305929	-1.030	8.40E-03
Or51E1	olfactory receptor, family 51, subfamily E, member 1	17481144	-1.151	1.18E-02
Or56A3	olfactory receptor, family 56, subfamily A, member 3	17481350	-1.123	2.98E-03
Or5V1	olfactory receptor, family 5, subfamily V, member 1	17337580	2.218	1.04E-03
Ormdl2	ORMDL sphingolipid biosynthesis regulator 2	17246345	1.599	1.92E-02
Osm	oncostatin M	17246803	2.377	1.31E-05
P2Rx4	purinergic receptor P2X, ligand gated ion channel, 4	17442149	1.963	4.86E-06
P2Rx7	purinergic receptor P2X, ligand gated ion channel, 7	17442128	1.006	1.38E-03
P2Ry10	purinergic receptor P2Y, G-protein coupled, 10	17537118	1.726	9.03E-04
P2Ry6	pyrimidinergic receptor P2Y, G-protein coupled, 6	17493869	1.144	1.35E-03
P4Ha1	prolyl 4-hydroxylase, alpha polypeptide I	17233536	1.156	4.24E-06
Paox	polyamine oxidase (exo-N4-amino)	17484484	1.008	8.99E-03
Paqr8	progestin and adipoQ receptor family member VIII	17211375	-1.037	1.05E-02
Pcdh7	protocadherin 7	17437558	1.086	5.70E-03
Pfkm	phosphofructokinase, muscle	17314577	-1.173	9.70E-04
Pgd	phosphogluconate dehydrogenase	17433040	1.015	3.69E-03
Pgk1	phosphoglycerate kinase 1	17537088	1.097	6.94E-04
Phf10	PHD finger protein 10	17341132	1.251	8.50E-03
Phf11	PHD finger protein 11	17307280	1.405	3.24E-03
Phxr1	per-hexamer repeat gene 1	17366423	1.453	8.38E-03
Pianp	PILR alpha associated neural protein	17462987	1.415	3.04E-05
Pik3Ap1	phosphoinositide-3-kinase adaptor protein 1	17364642	1.298	1.38E-04
Pim1	Pim-1 proto-oncogene, serine/threonine kinase	17335540	1.107	7.29E-03
Pitpnm3	PITPNM family member 3	17265570	-1.089	6.23E-04
Plac8	placenta-specific 8	17450121	2.192	4.33E-04
Plau	plasminogen activator, urokinase	17297537	2.152	1.44E-05

Supplementary Table 4. Differentially expressed transcripts in vascular fragments between EAE and naive mice at the remission phase

Gene symbol	Gene name	Affymetrix ID	Fold Change	Adj. palue
Plaur	plasminogen activator, urokinase receptor	17474974	1.434	3.00E-03
Plbd1	phospholipase B domain containing 1	17472114	3.058	4.26E-05
Plcb2	phospholipase C, beta 2	17389740	1.002	5.77E-03
Pld3	phospholipase D family, member 3	17488292	1.653	3.75E-06
Plek	pleckstrin	17260761	1.488	4.24E-06
Plekho1	pleckstrin homology domain containing, family O member 1	17407969	1.155	2.86E-04
Plekho2	pleckstrin homology domain containing, family O member 2	17528274	1.400	1.54E-03
Plin2	perilipin 2	17426981	1.098	1.26E-02
Pln	phospholamban	17233323	-1.037	2.06E-02
Plod1	procollagen-lysine, 2-oxoglutarate 5-dioxygenase 1	17432835	1.012	7.31E-05
Plxnc1	plexin C1	17244439	2.610	4.57E-05
Pnpla7	patatin-like phospholipase domain containing 7	17367765	1.212	8.81E-05
Postn	periostin, osteoblast specific factor	17397575	2.845	2.72E-07
Ppp1R14A	protein phosphatase 1, regulatory (inhibitor) subunit 14A	17476119	-1.080	5.43E-03
Prdx1	peroxiredoxin 1	17548658	1.505	6.01E-04
Prkcd	protein kinase C, delta	17304406	1.174	1.09E-03
Prrt2	proline-rich transmembrane protein 2	17496547	-1.165	1.06E-02
Psat1	phosphoserine aminotransferase 1	17363204	1.163	2.75E-04
Psd2	pleckstrin and Sec7 domain containing 2	17349607	-1.079	2.57E-03
Psd4	pleckstrin and Sec7 domain containing 4	17367698	1.443	3.86E-03
Ptafr	platelet-activating factor receptor	17419437	1.755	4.97E-03
Ptger4	prostaglandin E receptor 4 (subtype EP4)	17315718	2.086	2.19E-07
Ptgs2	prostaglandin-endoperoxide synthase 2 (prostaglandin G/H syı	17218060	2.101	1.60E-04
Ptk2B	protein tyrosine kinase 2 beta	17307860	1.401	2.18E-04
Ptpn22	protein tyrosine phosphatase, non-receptor type 22 (lymphoid)	17401269	2.273	2.16E-04
Ptpn6	protein tyrosine phosphatase, non-receptor type 6	17470796	1.880	9.59E-06
Ptpn7	protein tyrosine phosphatase, non-receptor type 7	17217566	1.104	7.39E-03
Ptprc	protein tyrosine phosphatase, receptor type, C	17227536	1.513	1.20E-05
Ptprz1	protein tyrosine phosphatase, receptor-type, Z polypeptide 1	17456381	-1.486	6.39E-03
Pycard	PYD and CARD domain containing	17496839	1.257	1.51E-03
Pygl	phosphorylase, glycogen, liver	17281721	1.458	3.10E-03
Pygm	phosphorylase, glycogen, muscle	17356874	-1.191	3.10E-03
Pygo1	pygopus family PHD finger 1	17519238	-1.013	1.23E-02
Rab27A	RAB27A, member RAS oncogene family	17519282	1.286	6.91E-05
Rab32	RAB32, member RAS oncogene family	17239227	1.667	4.10E-05
Rab7B	RAB7B, member RAS oncogene family	17217048	2.011	6.92E-04
Rac2	ras-related C3 botulinum toxin substrate 2 (rho family, small G	17319037	2.378	1.21E-05
Rap2C	RAP2C, member of RAS oncogene family	17541612	1.007	3.08E-03
Rasa3	RAS p21 protein activator 3	17507637	1.005	1.38E-03
Rasgef1B	RasGEF domain family, member 1B	17449989	2.268	4.24E-06
Rasgrp1	RAS guanyl releasing protein 1 (calcium and DAG-regulated)	17389647	1.439	5.33E-03
Rassf4	Ras association (RalGDS/AF-6) domain family member 4	17470060	2.072	6.29E-06
Rbpms2	RNA binding protein with multiple splicing 2	17548468	-1.142	2.20E-03
Rel	v-rel avian reticuloendotheliosis viral oncogene homolog	17261107	1.197	2.95E-04
Renbp	renin binding protein	17542501	1.712	4.69E-04
Rftn1	raftlin, lipid raft linker 1	17345865	1.522	6.94E-04
Rgs1	regulator of G-protein signaling 1	17227780	2.036	7.55E-04
Rgs10	regulator of G-protein signaling 10	17496898	1.087	6.79E-03
Rgs4	regulator of G-protein signaling 4	17229454	-1.051	2.84E-02
Rhoh	ras homolog family member H	17437830	1.342	7.73E-04
Rhox2A (Includes Others)	reproductive homeobox 2A	17534280	-1.053	5.10E-03
Rinl	Ras and Rab interactor-like	17475942	1.224	4.05E-04
Ripk2	receptor-interacting serine-threonine kinase 2	17423490	1.033	4.42E-03
Rnase4	ribonuclease, RNase A family, 4	17299575	1.243	7.28E-04
Rnase6	ribonuclease, RNase A family, k6	17299585	1.723	7.64E-04
Rnf19B	ring finger protein 19B	17418916	1.000	4.35E-03
Rpl11	ribosomal protein L11	17411099	1.769	1.29E-04
Rpl29 (Includes Others)	ribosomal protein L29	17414299	1.255	1.84E-05
Rpl34 (Includes Others)	ribosomal protein L34	17410381	1.039	1.35E-02
Rps12	ribosomal protein S12	17360842	1.087	3.08E-06
Rps15A-Ps4	ribosomal protein S15A, pseudogene 4	17430775	-1.031	7.86E-03
Rps23	ribosomal protein S23	17289031	1.267	3.59E-03
Rpsa	ribosomal protein SA	17411141	1.309	3.66E-05
Rragd	Ras-related GTP binding D	17412539	1.276	8.54E-03
Rreb1	ras responsive element binding protein 1	17286595	1.163	3.04E-05
Rtcb	RNA 2',3'-cyclic phosphate and 5'-OH ligase	17243678	1.139	8.80E-06
Runx1	runt-related transcription factor 1	17332236	1.067	8.05E-04
Rybp	RING1 and YY1 binding protein	17288231	-1.102	7.40E-03
S100A4	S100 calcium binding protein A4	17399802	1.209	2.07E-03
Saa3	serum amyloid A 3	17491193	4.004	3.24E-04
Sap18	Sin3A-associated protein, 18kDa	17504367	1.149	5.42E-03
Sap30	Sin3A-associated protein, 30kDa	17509455	1.215	2.99E-04
Scarna17	small Cajal body-specific RNA 17	17355435	-1.103	3.98E-04
Scgb3A1	secretoglobin, family 3A, member 1	17249028	2.591	3.15E-04
Scimp	SLP adaptor and CSK interacting membrane protein	17265386	2.159	9.70E-04
Scn4B	sodium channel, voltage gated, type IV beta subunit	17516740	-1.138	4.42E-04
Scpep1	serine carboxypeptidase 1	17267601	1.970	1.25E-05

Supplementary Table 4. Differentially expressed transcripts in vascular fragments between EAE and naive mice at the remission phase

Gene symbol	Gene name	Affymetrix ID	Fold Change	Adj. palue
Scrg1	stimulator of chondrogenesis 1	17501283	-1.651	7.28E-04
Sdf2L1	stromal cell-derived factor 2-like 1	17328625	1.654	1.40E-05
Sele	selectin E	17218820	1.393	1.66E-02
Sell	selectin L	17218835	1.663	6.99E-04
Selp	selectin P (granule membrane protein 140kDa, antigen CD62)	17218845	2.760	2.43E-04
Sema4A	sema domain, immunoglobulin domain (Ig), transmembrane do	17406760	1.122	4.12E-02
Sema6A	sema domain, transmembrane domain (TM), and cytoplasmic	17354299	1.119	1.92E-03
Serpina3	serpin peptidase inhibitor, clade A (alpha-1 antiproteinase, anti	17278328	4.695	1.09E-05
Serpina3G (Includes Othe	serine (or cysteine) peptidase inhibitor, clade A, member 3G	17278268	2.060	1.10E-03
Serpinb1	serpin peptidase inhibitor, clade B (ovalbumin), member 1	17291694	1.256	1.16E-03
Serpine1	serpin peptidase inhibitor, clade E (nexin, plasminogen activato	17453819	1.449	3.44E-02
Serpinf1	serpin peptidase inhibitor, clade F (alpha-2 antiplasmin, pigme	17265958	-1.039	3.66E-05
Sgpl1	sphingosine-1-phosphate lyase 1	17241162	1.440	1.18E-04
Sgpp2	sphingosine-1-phosphate phosphatase 2	17214665	-1.305	4.71E-03
Siglec1	sialic acid binding Ig-like lectin 1, sialoadhesin	17391834	1.851	6.27E-03
Sik1	salt inducible kinase 1	17343263	-1.026	1.55E-03
Sirpb1	signal-regulatory protein beta 1	17404200	1.134	1.91E-02
Sla	Src-like-adaptor	17317637	1.057	7.98E-03
Slamf1	signaling lymphocytic activation molecule family member 1	17219407	1.100	5.42E-03
Slamf6	SLAM family member 6	17219435	1.190	1.32E-04
Slamf7	SLAM family member 7	17229782	4.371	9.10E-06
Slamf8	SLAM family member 8	17229931	1.697	8.89E-03
Slamf9	SLAM family member 9	17219546	1.327	6.06E-05
Slc11A1	solute carrier family 11 (proton-coupled divalent metal ion trans	17214197	1.579	6.15E-04
Slc15A3	solute carrier family 15 (oligopeptide transporter), member 3	17357597	1.323	3.18E-04
Slc1A2	solute carrier family 1 (glial high affinity glutamate transporter),	17373696	-1.432	1.00E-04
Slc25A33	solute carrier family 25 (pyrimidine nucleotide carrier), member	17433221	-1.051	7.06E-03
Slc26A10	solute carrier family 26, member 10	17245729	-1.419	4.73E-05
Slc2A5	solute carrier family 2 (facilitated glucose/fructose transporter),	17421875	-1.105	2.47E-04
Slc30A10	solute carrier family 30, member 10	17220584	-1.168	7.91E-03
Slc37A2	solute carrier family 37 (glucose-6-phosphate transporter), mer	17525578	1.395	4.41E-03
Slc38A1	solute carrier family 38, member 1	17320907	1.441	6.00E-05
Slc40A1	solute carrier family 40 (iron-regulated transporter), member 1	17222777	-1.347	6.31E-04
Slc6A11	solute carrier family 6 (neurotransmitter transporter), member 1	17461852	-1.351	1.47E-02
Slc7A7	solute carrier family 7 (amino acid transporter light chain, y+L s	17306344	1.113	9.70E-04
Slc9A7	solute carrier family 9, subfamily A (NHE7, cation proton antipo	17540465	1.078	1.22E-02
Slc9A9	solute carrier family 9, subfamily A (NHE9, cation proton antipo	17520396	1.359	2.45E-04
Slfn1	schlafen 1	17254171	2.357	4.22E-03
Slfn13	schlafen family member 13	17266851	1.277	3.17E-02
Slfn2	schlafen 2	17254166	1.271	2.03E-03
Slpi	secretory leukocyte peptidase inhibitor	17394153	1.756	9.63E-03
Snai2	snail family zinc finger 2	17323192	-1.347	7.94E-04
Snhg6	small nucleolar RNA host gene 6	17221186	1.237	2.71E-05
Snora73B	small nucleolar RNA, H/ACA box 73b	17430831	1.179	1.16E-02
Snord35B	small nucleolar RNA, C/D box 35B	17490606	-1.024	4.47E-02
Snord49B	small nucleolar RNA, C/D box 49B	17250740	1.153	4.26E-03
Snord61	small nucleolar RNA, C/D box 61	17541917	1.147	4.32E-02
Snord65	small nucleolar RNA, C/D box 65	17250744	1.154	2.87E-02
Snord72	small nucleolar RNA, C/D box 72	17309905	1.252	2.05E-02
Snord87	small nucleolar RNA, C/D box 87	17221191	1.691	3.86E-03
Snx30	sorting nexin family member 30	17414482	1.189	1.12E-03
Snx8	sorting nexin 8	17454551	1.094	6.92E-03
Soat1	sterol O-acyltransferase 1	17228544	1.944	3.28E-05
Sorl1	sortilin-related receptor, L(DLR class) A repeats containing	17525894	1.697	7.42E-07
Sowahc	sosondowah ankyrin repeat domain family member C	17233534	1.365	6.00E-05
Sp110	SP110 nuclear body protein	17366258	1.164	9.48E-04
Spi1	Spi-1 proto-oncogene	17373177	1.151	3.18E-03
Spn	sialophorin	17496599	1.030	1.01E-02
Spp1	secreted phosphoprotein 1	17439830	4.985	2.13E-08
Sprr2E	small proline-rich protein 2E	17399872	-1.021	6.15E-03
Spry2	sprouty RTK signaling antagonist 2	17309340	-1.006	2.67E-03
Spry4	sprouty RTK signaling antagonist 4	17353957	-1.517	2.56E-03
St14	suppression of tumorigenicity 14 (colon carcinoma)	17525240	1.385	8.77E-05
St6Galnac3	ST6 (alpha-N-acetyl-neuraminyl-2,3-beta-galactosyl-1,3)-N-ace	17411262	-1.030	9.75E-03
Stap1	signal transducing adaptor family member 1	17438584	1.680	4.23E-05
Stat1	signal transducer and activator of transcription 1, 91kDa	17212750	1.106	2.69E-03
Stat4	signal transducer and activator of transcription 4	17212724	1.600	1.77E-02
Stxbp2	syntaxin binding protein 2	17498699	1.184	2.34E-03
Sulf2	sulfatase 2	17394538	1.094	6.15E-03
Syngr1	synaptogyrin 1	17313064	1.047	4.65E-04
Taar7D	trace amine-associated receptor 7D	17232189	-1.313	3.73E-03
Tap1	transporter 1, ATP-binding cassette, sub-family B (MDR/TAP)	17336432	1.241	6.10E-03
Tbc1D9	TBC1 domain family, member 9 (with GRAM domain)	17502874	1.212	4.69E-03
Tbxa2R	thromboxane A2 receptor	17235694	-1.095	8.81E-05
Tcrg-V1	T cell receptor gamma, variable 1	17285539	1.069	6.10E-03
Tcrg-V4	T cell receptor gamma, variable 4	17285523	2.020	4.52E-03
Tep1	telomerase-associated protein 1	17305988	1.147	3.27E-04

Supplementary Table 4. Differentially expressed transcripts in vascular fragments between EAE and naive mice at the remission phase

Gene symbol	Gene name	Affymetrix ID	Fold Change	Adj. palue
Tgfb2	transforming growth factor, beta 2	17230830	-1.229	1.21E-03
Tgfbi	transforming growth factor, beta-induced, 68kDa	17287827	2.394	3.60E-04
Thbs1	thrombospondin 1	17374488	1.567	1.10E-02
Themis2	thymocyte selection associated family member 2	17430906	1.616	7.28E-04
Thra	thyroid hormone receptor, alpha	17256138	-1.123	1.07E-04
Thy1	Thy-1 cell surface antigen	17516462	2.832	4.06E-05
Tigit	T cell immunoreceptor with Ig and ITIM domains	17330478	1.746	9.78E-03
Timp1	TIMP metallopeptidase inhibitor 1	17533713	1.880	1.26E-03
Tiparp	TCDD-inducible poly(ADP-ribose) polymerase	17398082	1.311	7.58E-05
Tlr1	toll-like receptor 1	17448245	1.186	6.68E-03
Tlr13	toll-like receptor 13	17537081	1.153	3.10E-03
Tlr2	toll-like receptor 2	17406279	1.891	5.70E-07
Tlr7	toll-like receptor 7	17546109	1.389	3.27E-04
Tlr8	toll-like receptor 8	17546101	1.337	4.12E-02
Tmed3	transmembrane p24 trafficking protein 3	17529634	1.279	3.99E-05
Tmem104	transmembrane protein 104	17258140	1.026	2.62E-03
Tmem106A	transmembrane protein 106A	17256784	1.778	1.11E-03
Tmem150C	transmembrane protein 150C	17450049	-1.030	2.52E-03
Tmem176A	transmembrane protein 176A	17458393	1.053	6.30E-03
Tmem47	transmembrane protein 47	17536067	-1.111	2.13E-03
Tmem68	transmembrane protein 68	17422959	1.055	1.70E-04
Tmtc2	transmembrane and tetratricopeptide repeat containing 2	17244661	-1.119	3.25E-03
Tnfa	tumor necrosis factor alpha	17344309	2.162	3.82E-06
Tnfaip3	tumor necrosis factor, alpha-induced protein 3	17239597	1.759	1.59E-07
Tnfaip8L2	tumor necrosis factor, alpha-induced protein 8-like 2	17407716	1.239	5.99E-03
Tnfrsf1B	tumor necrosis factor receptor superfamily, member 1B	17432674	1.032	5.43E-04
Tnfrsf22/Tnfrsf23	tumor necrosis factor receptor superfamily, member 23	17498323	1.297	1.87E-02
Tnfrsf4	tumor necrosis factor receptor superfamily, member 4	17422859	1.810	1.47E-03
Tnfsf13B	tumor necrosis factor (ligand) superfamily, member 13b	17498897	1.084	3.81E-03
Tnfsf8	tumor necrosis factor (ligand) superfamily, member 8	17426356	2.015	1.32E-03
Tnfsf9	tumor necrosis factor (ligand) superfamily, member 9	17338959	2.383	9.85E-05
Tomm5	translocase of outer mitochondrial membrane 5 homolog (yeas	17548454	1.319	3.25E-04
Traf3Ip3	TRAF3 interacting protein 3	17231203	1.286	1.09E-03
Trav12N-2	T cell receptor alpha variable 12N-2	17300225	2.382	5.13E-04
Trav14-1	T cell receptor alpha variable 14-1	17300165	2.469	1.37E-04
Trav14D-3-Dv8	T cell receptor alpha variable 14D-3-DV8	17300207	2.100	1.32E-02
Trav3N-3	T cell receptor alpha variable 3N-3	17300197	1.272	2.22E-02
Trav7-4	T cell receptor alpha variable 7-4	17300155	1.877	1.03E-03
Trbv1	T cell receptor beta, variable 1	17457804	3.479	1.82E-04
Trem2	triggering receptor expressed on myeloid cells 2	17338416	1.181	1.19E-04
Treml2	triggering receptor expressed on myeloid cells-like 2	17338388	1.862	2.27E-04
Trgv2	T cell receptor gamma variable 2	17290846	1.397	1.94E-02
Tril	TLR4 interactor with leucine-rich repeats	17466932	-1.169	5.18E-04
Tspan32	tetraspanin 32	17485282	1.101	1.56E-02
Ttyh1	tweety family member 1	17473248	-1.338	5.69E-03
Tuba4A	tubulin, alpha 4a	17224540	2.084	1.70E-06
Twf2	twinfilin actin binding protein 2	17521159	1.058	2.13E-03
Uap1L1	UDP-N-acetylglucosamine pyrophosphorylase 1 like 1	17382557	1.020	2.05E-03
Uba52	ubiquitin A-52 residue ribosomal protein fusion product 1	17423006	1.039	3.67E-05
Ubd	ubiquitin D	17337545	2.453	1.87E-03
Ugcg	UDP-glucose ceramide glucosyltransferase	17414434	1.080	8.23E-05
Umps	uridine monophosphate synthetase	17329937	1.079	1.76E-03
Unc5B	unc-5 netrin receptor B	17241137	-1.009	1.93E-02
Upk1B	uroplakin 1B	17330427	-1.168	6.43E-03
Upp1	uridine phosphorylase 1	17247225	1.249	1.65E-03
Uqcrhl	ubiquinol-cytochrome c reductase hinge protein like	17428545	1.227	3.48E-04
Ushbp1	Usher syndrome 1C binding protein 1	17510295	-1.123	3.10E-04
Usp12	ubiquitin specific peptidase 12	17455254	1.172	1.55E-04
Usp17La (Includes Others	ubiquitin specific peptidase 17-like A	17494162	-1.048	1.25E-03
Vav1	vav 1 guanine nucleotide exchange factor	17338982	1.175	7.85E-04
Vegfc	vascular endothelial growth factor C	17501160	-1.129	1.77E-02
Vmp1	vacuole membrane protein 1	17267329	1.052	3.08E-06
Vps50	VPS50 EARP/GARPII complex subunit	17455752	1.009	3.47E-03
Was	Wiskott-Aldrich syndrome	17539997	1.178	3.54E-03
Wasf3	WAS protein family, member 3	17444730	-1.020	4.83E-03
Wdfy4	WDFY family member 4	17304906	1.392	8.77E-05
Wdr46	WD repeat domain 46	17336268	1.225	6.98E-04
Wfdc17	WAP four-disulfide core domain 17	17254289	2.386	2.35E-03
Xcl1	chemokine (C motif) ligand 1	17229187	2.183	2.43E-04
Xlr4A (Includes Others)	X-linked lymphocyte-regulated 4A	17542254	1.291	3.10E-03
Zbp1	Z-DNA binding protein 1	17395079	2.922	8.77E-05
Zcchc6	zinc finger, CCHC domain containing 6	17293280	1.099	4.39E-03
Zdhhc2	zinc finger, DHHC-type containing 2	17500662	1.080	2.62E-02
Znf385A	zinc finger protein 385A	17322359	1.166	2.64E-03
Znf638	zinc finger protein 638	17460152	1.283	5.31E-03
Znf705A	zinc finger protein 705A	17462729	1.072	5.58E-03
1110038B12Rik	RIKEN cDNA 1110038B12 gene	17344114	1.004	3.42E-04

Supplementary Table 4. Differentially expressed transcripts in vascular fragments between EAE and naive mice at the remission phase

Gene symbol	Gene name	Affymetrix ID	Fold Change	Adj. palue
1500012F01Rik	RIKEN cDNA 1500012F01 gene	17379864	1.000	2.59E-03
1700084C06Rik	RIKEN cDNA 1700084C06 gene	17515103	1.122	1.02E-03
2410006H16Rik	RIKEN cDNA 2410006H16 gene	17250734	1.814	2.23E-06
5033404E19Rik	NSA2 ribosome biogenesis homolog pseudogene	17220591	-1.114	3.93E-02
5430402E10Rik/Gm14744	predicted gene 14744	17536028	-1.187	4.14E-03
5730408K05Rik	RIKEN cDNA 5730408K05 gene	17362521	1.416	1.10E-03
9430037G07Rik	RIKEN cDNA 9430037G07 gene	17529590	1.350	6.10E-03
9930111J21Rik2	RIKEN cDNA 9930111J21 gene 2	17262241	1.039	6.96E-03
A130077B15Rik	RIKEN cDNA A130077B15 gene	17245581	1.395	6.53E-04
A630001O12Rik	RIKEN cDNA A630001O12 gene	17506942	1.078	1.30E-02
A730082K24Rik	RIKEN cDNA A730082K24 gene	17482072	-1.052	1.14E-03

Supplementary Table 5. Differentially expressed transcripts in vascular fragments from EAE treated versus naive mice at all 3 disease phases

Gene Symbo	Entrez Gene Name	Affymetrix ID	Fold change preclinical	Fold change progression	Fold change remission
Ackr1	atypical chemokine receptor 1 (Duffy blood group)	17229984	3,19	3,84	2,94
Atf3	activating transcription factor 3	17231033	1,43	1,68	2,38
Bcl6B	B-cell CLL/lymphoma 6, member B	17265164	-1,33	-1,21	-1,26
Ccl4	chemokine (C-C motif) ligand 4	17254283	1,29	2,36	2,53
Ccl3L3	chemokine (C-C motif) ligand 3-like 3	17266967	1,14	2,22	2,36
Cd14	CD14 molecule	17353747	1,00	2,19	1,85
Ch25H	cholesterol 25-hydroxylase	17364111	1,94	2,73	2,10
Cyp2E1	cytochrome P450, family 2, subfamily E, polypeptide 1	17484587	-1,12	-2,33	-1,20
Fkbp5	FK506 binding protein 5	17342868	1,55	1,47	1,10
Gadd45B	growth arrest and DNA-damage-inducible, beta	17235511	1,32	1,74	1,52
Gadd45G	growth arrest and DNA-damage-inducible, gamma	17287361	1,51	1,89	1,22
Gm14023	predicted gene 14023	17376153	1,03	1,63	1,23
Gsdmc	gasdermin C	17317472	-1,16	-1,17	-1,05
Ier3	immediate early response 3	17337228	1,05	1,73	1,20
Ighm	immunoglobulin heavy constant mu	17284605	-1,42	-1,60	2,83
Il1B	interleukin 1, beta	17391565	1,92	5,31	4,25
Inpp5D	inositol polyphosphate-5-phosphatase D	17215309	1,27	1,54	1,72
Lcn2	lipocalin 2	17383892	3,59	4,09	2,34
Lrg1	leucine-rich alpha-2-glycoprotein 1	17346150	1,94	2,70	1,89
Nfkbiz	nuclear factor of kappa light polypeptide gene enhancer in B-cells inhibitor, zeta	17330967	1,17	1,15	1,25
Ptgs2	prostaglandin-endoperoxide synthase 2	17218060	2,04	3,25	2,10
Rab27A	RAB27A, member RAS oncogene family	17519282	1,08	1,79	1,29
Scgb3A1	secretoglobin, family 3A, member 1	17249028	1,90	3,74	2,59
Serpina3	serpin peptidase inhibitor, clade A (alpha-1 antiproteinase, antitrypsin), member	17278328	4,22	6,72	4,70
Serpina3G	serine (or cysteine) peptidase inhibitor, clade A, member 3G	17278268	2,32	4,10	2,06
Tnfa	tumor necrosis factor alpha	17344309	1,65	3,59	2,16

Supplementary Table 6. Differentially expressed transcripts in vascular fragments in PBS compared to imatinib treated EAE immunized mice at the progression phase

Gene symbol	Entrez Gene name	Affymetrix ID	Fold change	Adj. pvalue
Abca9	ATP-binding cassette, sub-family A (ABC1), member 9	17271399	1.785	1.83E-02
Abcg2	ATP-binding cassette, sub-family G (WHITE), member 2 (Junior blood group)	17459014	1.241	2.35E-02
Abracl	ABRA C-terminal like	17548593	-1.261	4.55E-02
Acp5	acid phosphatase 5, tartrate resistant	17524930	-2.109	4.65E-02
Acvr1B	activin A receptor, type IB	17315152	-1.479	1.41E-02
Adam8	ADAM metallopeptidase domain 8	17497525	-2.554	2.94E-02
Adamts4	ADAM metallopeptidase with thrombospondin type 1 motif, 4	17219248	-1.098	1.05E-02
Adamts9	ADAM metallopeptidase with thrombospondin type 1 motif, 9	17469136	-2.281	3.78E-03
Adgra2	adhesion G protein-coupled receptor A2	17500301	1.164	2.77E-02
Adgrg6	adhesion G protein-coupled receptor G6	17239435	1.260	4.86E-02
Adh1C	alcohol dehydrogenase 1C (class I), gamma polypeptide	17403070	1.884	2.66E-02
Adrb2	adrenoceptor beta 2, surface	17354857	1.000	1.05E-02
Afap1L1	actin filament associated protein 1-like 1	17354810	1.335	3.60E-02
Agmo	alkylglycerol monooxygenase	17275069	1.361	1.28E-02
Agrn	agrin	17433977	1.045	4.65E-02
Ai504432	expressed sequence AI504432	17401530	-2.908	9.87E-03
Ai506816	expressed sequence AI506816	17446060	-1.268	4.04E-02
Ai662270	expressed sequence AI662270	17254194	-1.893	2.79E-02
Ai839979	expressed sequence AI839979	17447013	-1.507	1.80E-02
Aldh1A1	aldehyde dehydrogenase 1 family, member A1	17358103	1.423	4.89E-02
Alg8	ALG8, alpha-1,3-glucosyltransferase	17480327	-1.174	5.50E-03
Alox12	arachidonate 12-lipoxygenase	17265193	1.125	3.12E-02
Alox5Ap	arachidonate 5-lipoxygenase-activating protein	17444961	-1.131	1.52E-02
Alpl	alkaline phosphatase, liver/bone/kidney	17431720	1.694	3.82E-02
Amica1	adhesion molecule, interacts with CXADR antigen 1	17516718	-2.247	2.35E-02
Anpep	alanyl (membrane) aminopeptidase	17492431	-2.229	4.56E-02
Antxr1	anthrax toxin receptor 1	17468573	1.204	3.75E-02
Anxa1	annexin A1	17363407	-1.301	2.15E-02
Aoah	acyloxyacyl hydrolase (neutrophil)	17285586	-2.812	4.10E-02
Apobec1	apolipoprotein B mRNA editing enzyme, catalytic polypeptide 1	17470580	-1.425	4.70E-02
Aprt	adenine phosphoribosyltransferase	17513771	-1.560	1.19E-02
Aqp11	aquaporin 11	17493461	1.486	1.69E-02
Arg1	arginase 1	17239845	-4.798	3.94E-02
Arg2	arginase 2	17276776	-2.009	3.38E-02
Arhgap30	Rho GTPase activating protein 30	17219324	-1.325	3.12E-02
Arhgef12	Rho guanine nucleotide exchange factor (GEF) 12	17526038	1.265	4.14E-02
Arl4A	ADP-ribosylation factor-like 4A	17280810	1.224	4.01E-02
Arrdc4	arrestin domain containing 4	17492051	-1.357	4.11E-02
Arxes1/Arxes2	adipocyte-related X-chromosome expressed sequence 2	17537861	1.135	4.72E-02
Asf1B	anti-silencing function 1B histone chaperone	17503023	-1.538	1.19E-02
Asna1	arsA arsenite transporter, ATP-binding, homolog 1 (bacterial)	17511277	-1.097	2.35E-02
Asprv1	aspartic peptidase, retroviral-like 1	17460465	-1.635	4.65E-02
Atic	5-aminoimidazole-4-carboxamide ribonucleotide formyltransferase/IMP cyclohydrolase	17213990	-1.331	8.07E-03
Atp1A3	ATPase, Na+/K+ transporting, alpha 3 polypeptide	17487805	-2.235	2.41E-02
Atp6V0C	ATPase, H+ transporting, lysosomal 16kDa, V0 subunit c	17457343	-1.711	3.63E-02
Atp8B4	ATPase, class I, type 8B, member 4	17391056	-2.897	2.94E-02
Aurkb	aurora kinase B	17251485	-1.325	4.55E-02
Axin2	axin 2	17257801	1.075	4.71E-02
B3Gnt5	UDP-GlcNAc:betaGal beta-1,3-N-acetylglucosaminyltransferase 5	17323828	-1.003	2.91E-02
B430306N03Rik	RIKEN cDNA B430306N03 gene	17338403	-2.008	1.47E-02
B4Galnt1	beta-1,4-N-acetyl-galactosaminyl transferase 1	17237937	-1.774	1.42E-02
B4Galt1	UDP-Gal:betaGlcNAc beta 1,4- galactosyltransferase, polypeptide 1	17424077	-1.046	2.48E-02
B4Galt4	UDP-Gal:betaGlcNAc beta 1,4- galactosyltransferase, polypeptide 4	17325637	1.425	3.12E-02
Bak1	BCL2-antagonist/killer 1	17342676	-1.247	1.77E-02
Batf	basic leucine zipper transcription factor, ATF-like	17277404	-1.285	7.91E-03
Batf3	basic leucine zipper transcription factor, ATF-like 3	17220787	-1.193	1.23E-02
Bc147527	cDNA sequence BC147527	17290205	-1.205	4.90E-02
Bche	butyrylcholinesterase	17405908	2.210	1.09E-02
Bcl2A1	BCL2-related protein A1	17520162	-2.403	1.05E-02
Bcl2A1C	B cell leukemia/lymphoma 2 related protein A1c	17522726	-1.297	2.39E-02
Bcl3	B-cell CLL/lymphoma 3	17487457	-1.009	1.15E-02
Be692007	expressed sequence BE692007	17362966	-1.548	2.75E-02
Bend7	BEN domain containing 7	17366484	1.040	1.80E-02
Bin2	bridging integrator 2	17321768	-1.117	1.42E-02
Birc5	baculoviral IAP repeat containing 5	17258867	-1.583	3.89E-02
Bri3Bp	BRI3 binding protein	17442714	-1.233	2.20E-02
Bst1	bone marrow stromal cell antigen 1	17437198	-2.218	8.07E-03
Btg1	B-cell translocation gene 1, anti-proliferative	17236787	-1.048	2.47E-02
Bub1	BUB1 mitotic checkpoint serine/threonine kinase	17391376	-1.383	4.99E-02
Bub1B	BUB1 mitotic checkpoint serine/threonine kinase B	17374569	-1.603	2.84E-02
C130026I21Rik (In	RIKEN cDNA C130026I21 gene	17366201	-1.939	2.64E-02
C15Orf48	chromosome 15 open reading frame 48	17375503	-6.191	8.36E-03
C16Orf89	chromosome 16 open reading frame 89	17327950	1.004	3.12E-02
C19Orf12	chromosome 19 open reading frame 12	17476872	-1.161	2.61E-02
C19Orf38	chromosome 19 open reading frame 38	17515238	-3.048	1.23E-02
C3	complement component 3	17346528	-4.295	7.42E-03
C4A/C4B	complement component 4B (Chido blood group)	17343918	-2.110	1.77E-02
C5Ar1	complement component 5a receptor 1	17486864	-2.180	3.02E-03
C7Orf50	chromosome 7 open reading frame 50	17454408	-1.271	2.36E-02
C920009B18Rik	RIKEN cDNA C920009B18 gene	17232112	-1.514	4.06E-02
Ca13	carbonic anhydrase XIII	17396143	-1.952	5.76E-03

Supplementary Table 6. Differentially expressed transcripts in vascular fragments in PBS compared to imatinib treated EAE immunized mice at the progression phase

Gene symbol	Entrez Gene name	Affymetrix ID	Fold change	Adj. pvalue
Ca14	carbonic anhydrase XIV	17407956	1.049	1.90E-02
Ca2	carbonic anhydrase II	17396162	1.187	3.80E-02
Ca4	carbonic anhydrase IV	17254508	1.505	2.87E-02
Cables1	Cdk5 and Abl enzyme substrate 1	17348435	1.182	1.46E-02
Caskin2	CASK interacting protein 2	17272019	1.008	3.97E-02
Casp1	caspase 1, apoptosis-related cysteine peptidase	17514424	-2.242	7.71E-03
Casp4	caspase 4, apoptosis-related cysteine peptidase	17514435	-1.205	2.92E-02
Cav1	caveolin 1, caveolae protein, 22kDa	17456161	1.157	4.53E-02
Cav2	caveolin 2	17456152	1.257	2.81E-02
Ccdc86	coiled-coil domain containing 86	17362831	-1.641	2.42E-02
Ccl11	chemokine (C-C motif) ligand 11	17254053	-2.036	1.42E-02
Ccl19	chemokine (C-C motif) ligand 19	17434280	-1.116	4.56E-02
Ccl2	chemokine (C-C motif) ligand 2	17254041	-4.233	3.15E-04
Ccl22	chemokine (C-C motif) ligand 22	17504122	-2.895	4.53E-02
Ccl27A	chemokine (C-C motif) ligand 27A	17413137	1.004	2.75E-02
Ccl3L3	chemokine (C-C motif) ligand 3-like 3	17266967	-1.937	4.61E-03
Ccl4	chemokine (C-C motif) ligand 4	17254283	-2.270	5.16E-03
Ccl5	chemokine (C-C motif) ligand 5	17266946	-3.544	3.23E-02
Ccl6	chemokine (C-C motif) ligand 6	17266960	-3.092	1.19E-02
Ccl7	chemokine (C-C motif) ligand 7	17254047	-4.868	5.57E-03
Ccl8	chemokine (C-C motif) ligand 8	17254065	-1.773	2.93E-02
Ccl9	chemokine (C-C motif) ligand 9	17266952	-1.413	1.92E-02
Ccna2	cyclin A2	17404821	-1.320	4.82E-02
Ccnb1	cyclin B1	17295757	-1.343	3.49E-02
Ccr1	chemokine (C-C motif) receptor 1	17532569	-2.920	1.88E-02
Ccr2	chemokine (C-C motif) receptor 2	17523650	-3.728	3.64E-03
Ccr5	chemokine (C-C motif) receptor 5 (gene/pseudogene)	17523659	-1.275	1.24E-02
Cd14	CD14 molecule	17353747	-1.255	6.38E-03
Cd177	CD177 molecule	17487759	-1.055	4.11E-02
Cd180	CD180 molecule	17289527	-1.455	2.01E-02
Cd2	CD2 molecule	17408497	-2.553	4.14E-02
Cd244	CD244 molecule, natural killer cell receptor 2B4	17219382	-2.323	6.13E-03
Cd274	CD274 molecule	17358544	-2.494	4.37E-02
Cd300Ld	CD300 molecule-like family member d	17271724	-1.681	1.23E-02
Cd300Lf	CD300 molecule-like family member f	17271776	-4.054	1.28E-02
Cd36	CD36 molecule (thrombospondin receptor)	17445715	-3.774	1.35E-02
Cd38	CD38 molecule	17437213	-1.334	3.74E-02
Cd3E	CD3e molecule, epsilon (CD3-TCR complex)	17526464	-2.538	3.57E-02
Cd40Lg	CD40 ligand	17535048	-1.623	4.52E-02
Cd44	CD44 molecule (Indian blood group)	17388733	-2.864	6.74E-03
Cd48	CD48 molecule	17219397	-1.380	4.17E-02
Cd52	CD52 antigen	17431174	-1.504	1.49E-02
Cd53	CD53 molecule	17408960	-1.226	1.09E-02
Cd68	CD68 molecule	17264835	-1.132	3.89E-02
Cd69	CD69 molecule	17471502	-3.777	7.06E-03
Cd72	CD72 molecule	17424608	-2.363	2.84E-03
Cd74	CD74 molecule, major histocompatibility complex, class II invariant chain	17350982	-3.127	8.32E-03
Cd86	CD86 molecule	17330203	-1.315	4.17E-02
Cdc14A	cell division cycle 14A	17409668	1.021	3.34E-02
Cdca5	cell division cycle associated 5	17356622	-1.238	4.43E-02
Cdca8	cell division cycle associated 8	17429896	-1.037	4.71E-02
Cdh2	cadherin 2, type 1, N-cadherin (neuronal)	17352884	1.062	3.51E-02
Cdk1	cyclin-dependent kinase 1	17241692	-1.752	9.57E-03
Cdt1	chromatin licensing and DNA replication factor 1	17506418	-1.196	1.91E-02
Cebpb	CCAAT/enhancer binding protein (C/EBP), beta	17379938	-1.287	1.43E-02
Cenph	centromere protein H	17295745	-1.385	2.37E-02
Cenpk	centromere protein K	17289584	-1.235	3.97E-02
Cenpq	centromere protein Q	17344990	-1.286	3.76E-02
Cers6	ceramide synthase 6	17371374	-2.516	2.09E-02
Cfb	complement factor B	17344064	-2.723	1.53E-02
Cfp	complement factor properdin	17540521	-1.650	2.22E-02
Chaf1B	chromatin assembly factor 1, subunit B (p60)	17327331	-1.167	1.97E-02
Chil3/Chil4	chitinase-like 3	17408897	-3.747	8.07E-03
Chst15	carbohydrate (N-acetylgalactosamine 4-sulfate 6-O) sulfotransferase 15	17497076	1.146	1.42E-02
Ckap2	cytoskeleton associated protein 2	17508025	-1.231	2.74E-02
Ckap2L	cytoskeleton associated protein 2-like	17391544	-1.043	1.88E-02
Clcn7	chloride channel, voltage-sensitive 7	17334545	-1.725	3.08E-02
Clec12A	C-type lectin domain family 12, member A	17463509	-3.861	1.90E-02
Clec14A	C-type lectin domain family 14, member A	17281350	1.082	4.37E-02
Clec4D	C-type lectin domain family 4, member D	17462796	-4.496	1.75E-02
Clec4E	C-type lectin domain family 4, member E	17470627	-4.796	1.10E-02
Clec5A	C-type lectin domain family 5, member A	17466228	-2.084	1.55E-02
Clec6A	C-type lectin domain family 6, member A	17462788	-4.187	1.88E-02
Clec7A	C-type lectin domain family 7, member A	17471541	-4.978	6.96E-03
Clic5	chloride intracellular channel 5	17337852	1.442	1.42E-02
Clip1	CAP-GLY domain containing linker protein 1	17452640	1.025	3.55E-02
Clstn1	calsyntenin 1	17421828	1.157	4.56E-02
Cmtm3	CKLF-like MARVEL transmembrane domain containing 3	17504444	-1.175	1.90E-02
Cmtm8	CKLF-like MARVEL transmembrane domain containing 8	17531932	1.788	8.35E-03
Cndp2	CNDP dipeptidase 2 (metallopeptidase M20 family)	17355825	-1.619	1.16E-02
Cobll1	cordon-bleu WH2 repeat protein-like 1	17385879	1.327	3.08E-02

Supplementary Table 6. Differentially expressed transcripts in vascular fragments in PBS compared to imatinib treated EAE immunized mice at the progression phase

Gene symbol	Entrez Gene name	Affymetrix ID	Fold change	Adj. pvalue
Col4A3Bp	collagen, type IV, alpha 3 (Goodpasture antigen) binding protein	17289304	1.115	1.88E-02
Colca2	colorectal cancer associated 2	17526982	1.132	3.38E-02
Coro1A	coronin, actin binding protein, 1A	17496376	-1.347	8.35E-03
Cotl1	coactosin-like F-actin binding protein 1	17513491	-1.814	1.32E-02
Cox6A2	cytochrome c oxidase subunit VIa polypeptide 2	17496857	-1.507	4.86E-02
Creb5	cAMP responsive element binding protein 5	17458682	-2.206	3.23E-02
Crip2	cysteine rich protein 2	17279499	1.157	4.25E-02
Crispld1	cysteine-rich secretory protein LCCL domain containing 1	17211313	1.631	1.39E-02
Crtap	cartilage associated protein	17531877	1.005	2.84E-02
Csf2Rb	colony stimulating factor 2 receptor, beta, low-affinity (granulocyte-macrophage)	17318950	-3.321	5.50E-03
Csmd3	CUB and Sushi multiple domains 3	17316878	1.352	6.74E-03
Cst7	cystatin F (leukocystatin)	17377464	-2.280	2.84E-02
Cthrc1	collagen triple helix repeat containing 1	17311191	1.352	1.59E-02
Ctrcos	chymotrypsin C (caldecrin), opposite strand	17421138	-1.157	3.36E-02
Ctsc	cathepsin C	17480018	-1.788	5.76E-03
Ctsz	cathepsin Z	17395155	-1.269	2.75E-02
Cttnbp2	cortactin binding protein 2	17465006	1.153	3.57E-02
Cuedc1	CUE domain containing 1	17254948	1.071	3.49E-02
Cxcl10	chemokine (C-X-C motif) ligand 10	17449718	-4.850	3.64E-03
Cxcl11	chemokine (C-X-C motif) ligand 11	17449725	-2.444	3.21E-02
Cxcl16	chemokine (C-X-C motif) ligand 16	17265268	-2.295	1.40E-02
Cxcl9	chemokine (C-X-C motif) ligand 9	17449710	-5.798	6.29E-03
Cxcr2	chemokine (C-X-C motif) receptor 2	17214142	-2.223	3.57E-02
Cxcr4	chemokine (C-X-C motif) receptor 4	17226593	-2.311	2.49E-02
Cxorf36	chromosome X open reading frame 36	17540436	1.178	1.52E-02
Cybb	cytochrome b-245, beta polypeptide	17540154	-4.091	6.31E-03
Cycs	cytochrome c, somatic	17445624	-1.028	1.77E-02
Cyp2E1	cytochrome P450, family 2, subfamily E, polypeptide 1	17484587	1.088	8.98E-03
Cyp4F2	cytochrome P450, family 4, subfamily F, polypeptide 2	17510462	-2.313	1.81E-02
Cyth3	cytohesin 3	17444372	1.085	3.03E-02
Cytip	cytohesin 1 interacting protein	17385405	-2.532	7.06E-03
D17H6S56E-5	DNA segment, Chr 17, human D6S56E 5	17344140	-1.115	1.07E-02
D730003I15Rik	RIKEN cDNA D730003I15 gene	17220794	1.228	1.28E-02
Dach1	dachshund family transcription factor 1	17309041	1.315	1.23E-02
Dbnl	drebrin-like	17247023	-1.387	2.85E-02
Dbp	D site of albumin promoter (albumin D-box) binding protein	17477979	1.324	1.06E-02
Dck	deoxycytidine kinase	17438823	-1.466	3.49E-02
Ddah1	dimethylarginine dimethylaminohydrolase 1	17403439	1.199	3.12E-02
Ddc	dopa decarboxylase (aromatic L-amino acid decarboxylase)	17260644	1.449	2.63E-02
Degs2	delta(4)-desaturase, sphingolipid 2	17283915	1.725	1.83E-02
Dennd5B	DENN/MADD domain containing 5B	17473061	1.503	2.72E-02
Dgat2	diacylglycerol O-acyltransferase 2	17493632	-1.147	2.79E-02
Dgkh	diacylglycerol kinase, eta	17308842	1.212	1.88E-02
Dna2	DNA replication helicase/nuclease 2	17233811	-1.861	1.64E-02
Dnm3	dynamin 3	17228906	1.187	2.14E-02
Dtl	denticleless E3 ubiquitin protein ligase homolog (Drosophila)	17231066	-1.049	2.82E-02
Dusp10	dual specificity phosphatase 10	17220475	-1.104	4.11E-02
Dut	deoxyuridine triphosphatase	17375641	-1.128	1.09E-02
E030002O03Rik	RIKEN cDNA E030002O03 gene	17494307	1.080	1.66E-02
E2F8	E2F transcription factor 8	17491378	-1.557	1.28E-02
Ebf1	early B-cell factor 1	17248691	1.298	4.97E-02
Ecm2	extracellular matrix protein 2, female organ and adipocyte specific	17287148	1.659	3.74E-02
Edn3	endothelin 3	17380377	1.402	1.80E-02
Eef1E1	eukaryotic translation elongation factor 1 epsilon 1	17291964	-1.203	8.35E-03
Efhc2	EF-hand domain (C-terminal) containing 2	17540402	1.269	4.16E-02
Efhd2	EF-hand domain family, member D2	17432341	-1.412	7.47E-03
Egflam	EGF-like, fibronectin type III and laminin G domains	17315763	1.508	1.88E-02
Egln3	egl-9 family hypoxia-inducible factor 3	17281084	-2.935	3.83E-02
Elf4	E74-like factor 4 (ets domain transcription factor)	17541404	-1.286	1.45E-02
Elovl7	ELOVL fatty acid elongase 7	17289717	1.553	3.93E-02
Emb	embigin	17290083	-3.873	7.67E-03
Emilin2	elastin microfibril interfacer 2	17346975	-3.015	8.07E-03
Emp1	epithelial membrane protein 1	17463781	-1.022	3.03E-02
Emp2	epithelial membrane protein 2	17328062	1.499	4.50E-02
Emp3	epithelial membrane protein 3	17491026	-1.127	3.51E-02
Enpep	glutamyl aminopeptidase (aminopeptidase A)	17410251	1.042	4.81E-02
Enpp2	ectonucleotide pyrophosphatase/phosphodiesterase 2	17317056	1.733	2.11E-02
Enpp5	ectonucleotide pyrophosphatase/phosphodiesterase 5 (putative)	17337844	1.013	3.04E-02
Eogt	EGF domain-specific O-linked N-acetylglucosamine (GlcNAc) transferase	17469289	1.114	3.54E-02
Epsti1	epithelial stromal interaction 1 (breast)	17302141	-1.444	4.77E-02
Ero1A	endoplasmic reticulum oxidoreductase alpha	17305636	-1.100	3.27E-02
Esyt2	extended synaptotagmin-like protein 2	17279584	1.265	3.37E-02
Etv6	ets variant 6	17463673	-1.396	8.07E-03
Evi2A	ecotropic viral integration site 2A	17266590	-1.096	2.75E-02
Ezh1	enhancer of zeste 1 polycomb repressive complex 2 subunit	17269809	1.099	8.64E-03
F10	coagulation factor X	17499224	-4.702	1.38E-02
F13A1	coagulation factor XIII, A1 polypeptide	17291881	-2.327	1.88E-02
F830016B08Rik	RIKEN cDNA F830016B08 gene	17350921	-2.427	4.71E-02
Fam111A	family with sequence similarity 111, member A	17357815	-1.090	7.06E-03
Fam13A	family with sequence similarity 13, member A	17467209	1.237	2.60E-02
Fam214A	family with sequence similarity 214, member A	17519364	1.082	8.07E-03

Supplementary Table 6. Differentially expressed transcripts in vascular fragments in PBS compared to imatinib treated EAE immunized mice at the progression phase

Gene symbol	Entrez Gene name	Affymetrix ID	Fold change	Adj. pvalue
Fam26F	family with sequence similarity 26, member F	17240186	-2.616	1.96E-02
Fam65B	family with sequence similarity 65, member B	17285964	-1.047	2.74E-02
Far2	fatty acyl CoA reductase 2	17464455	1.417	3.39E-02
Fat4	FAT atypical cadherin 4	17397185	1.113	4.80E-02
Fbl	fibrillarin	17475777	-1.214	8.40E-03
Fbxl5	F-box and leucine-rich repeat protein 5	17447803	-1.381	1.42E-02
Fcgr1A	Fc fragment of IgG, high affinity Ia, receptor (CD64)	17408024	-1.798	6.74E-03
Fcgr2A	Fc fragment of IgG, low affinity IIa, receptor (CD32)	17229620	-1.179	1.07E-02
Fcgr2B	Fc fragment of IgG, low affinity IIb, receptor (CD32)	17229607	-1.810	9.36E-03
Fcgr3A/Fcgr3B	Fc fragment of IgG, low affinity IIIa, receptor (CD16a)	17219199	-4.032	3.78E-03
Fcgrt	Fc fragment of IgG, receptor, transporter, alpha	17490589	1.222	1.90E-02
Fendrr	Foxf1 adjacent non-coding developmental regulatory RNA	17513550	1.402	1.96E-02
Fgl2	fibrinogen-like 2	17435089	-2.770	7.71E-03
Fgr	FGR proto-oncogene, Src family tyrosine kinase	17419483	-2.594	1.59E-02
Filip1	filamin A interacting protein 1	17529185	1.184	4.14E-02
Flrt2	fibronectin leucine rich transmembrane protein 2	17277788	1.515	8.98E-03
Fmo1	flavin containing monooxygenase 1	17229020	1.930	1.05E-02
Fmo2	flavin containing monooxygenase 2 (non-functional)	17229036	2.499	1.42E-02
Fmo5	flavin containing monooxygenase 5	17400773	1.197	2.56E-02
Foxl2Os	forkhead box L2, opposite strand	17529981	1.059	2.21E-02
Foxq1	forkhead box Q1	17286320	1.109	4.49E-02
Fpr2	formyl peptide receptor 2	17333731	-3.152	3.12E-02
Frem2	FRAS1 related extracellular matrix protein 2	17405208	1.364	9.67E-03
Frk	fyn-related Src family tyrosine kinase	17232534	1.085	2.26E-02
Fry	furry homolog (Drosophila)	17445160	1.159	1.97E-02
Ftl	ferritin, light polypeptide	17246505	1.314	2.24E-02
Fzd4	frizzled class receptor 4	17480036	1.237	3.76E-02
Fzd6	frizzled class receptor 6	17311179	1.662	2.57E-02
Fzd8	frizzled class receptor 8	17348276	1.185	5.76E-03
Gadd45B	growth arrest and DNA-damage-inducible, beta	17235511	-1.110	2.35E-02
Galnt6	polypeptide N-acetylgalactosaminyltransferase 6	17321790	-1.696	3.45E-02
Gapdh	glyceraldehyde-3-phosphate dehydrogenase	17260881	-1.103	7.31E-03
Gas5	growth arrest-specific 5 (non-protein coding)	17218680	-1.024	2.50E-02
Gas6	growth arrest-specific 6	17507605	1.069	4.06E-02
Gatsl3	GATS protein-like 3	17246790	1.074	1.28E-02
Gbp2	guanylate binding protein 2, interferon-inducible	17403268	-1.800	3.32E-02
Gda	guanine deaminase	17363470	-2.513	8.44E-03
Gdap10	ganglioside-induced differentiation-associated-protein 10	17274889	-1.338	4.67E-02
Ggct	gamma-glutamylcyclotransferase	17467031	-1.147	1.06E-02
Gimap1-Gimap5	GIMAP1-GIMAP5 readthrough	17466618	-1.679	2.97E-02
Gins1	GINS complex subunit 1 (Psf1 homolog)	17377525	-1.653	9.42E-03
Gins2	GINS complex subunit 2 (Psf2 homolog)	17513525	-1.086	2.03E-02
Gk	glycerol kinase	17543045	-1.207	3.30E-02
Gkn3	gastrokine 3	17468602	2.493	4.06E-02
Glipr2	GLI pathogenesis-related 2	17413500	-1.748	2.29E-02
Glrx	glutaredoxin (thioltransferase)	17288716	-1.671	3.38E-02
Gm10790	predicted gene 10790	17286808	1.511	9.56E-03
Gm11110	predicted gene 11110	17346510	-1.007	4.83E-02
Gm11974	predicted gene 11974	17260396	-1.206	6.46E-03
Gm12250	predicted gene 12250	17249977	-3.056	1.24E-02
Gm12264	predicted gene 12264	17263456	1.131	4.60E-02
Gm13293	predicted gene 13293	17366985	1.728	6.74E-03
Gm14023	predicted gene 14023	17376153	-1.048	2.31E-02
Gm15523	predicted gene 15523	17278183	1.188	1.88E-02
Gm16046	predicted gene 16046	17341039	1.421	1.32E-02
Gm16894	predicted gene, 16894	17222601	-1.077	3.94E-02
Gm19951	predicted gene, 19951	17277876	-1.656	4.58E-02
Gm20300	predicted gene, 20300	17240043	1.123	2.85E-02
Gm3383 (Includes	predicted gene 3383	17303368	1.753	5.57E-03
Gm35034	predicted gene, 35034	17548321	-1.096	1.40E-02
Gm4841	predicted gene 4841	17354589	-2.988	1.42E-02
Gm4951	predicted gene 4951	17350916	-2.644	2.96E-02
Gm5069	glyceraldehyde-3-phosphate dehydrogenase pseudogene	17230451	1.658	1.54E-02
Gm5086	predicted gene 5086	17289388	1.079	1.42E-02
Gm5150	predicted gene 5150	17404230	-1.466	1.72E-02
Gm5423	transmembrane protein 229B pseudogene	17233329	-1.059	1.57E-02
Gm7265	predicted gene 7265	17548726	1.116	1.03E-02
Gm9733	predicted gene 9733	17404195	-2.335	7.69E-03
Gm9946	predicted gene 9946	17462162	1.950	2.81E-02
Gna13	guanine nucleotide binding protein (G protein), alpha 13	17257822	-1.119	2.33E-02
Gnb4	guanine nucleotide binding protein (G protein), beta polypeptide 4	17404601	1.082	2.32E-02
Got1	glutamic-oxaloacetic transaminase 1, soluble	17364932	-1.374	1.95E-02
Gpnmb	glycoprotein (transmembrane) nmb	17458439	-4.235	4.45E-02
Gpr141	G protein-coupled receptor 141	17290894	-3.459	1.26E-02
Gpr165	G protein-coupled receptor 165	17536420	2.078	5.76E-03
Gpr171	G protein-coupled receptor 171	17405458	-3.235	1.40E-02
Gpr18	G protein-coupled receptor 18	17309644	-1.768	4.28E-02
Gpr34	G protein-coupled receptor 34	17533446	1.804	6.38E-03
Gpr35	G protein-coupled receptor 35	17215873	-2.287	9.95E-03
Gpr65	G protein-coupled receptor 65	17277794	-1.978	3.97E-03
Gpr84	G protein-coupled receptor 84	17322355	-1.764	3.64E-03

Supplementary Table 6. Differentially expressed transcripts in vascular fragments in PBS compared to imatinib treated EAE immunized mice at the progression phase

Gene symbol	Entrez Gene name	Affymetrix ID	Fold change	Adj. pvalue
Grb14	growth factor receptor-bound protein 14	17385853	1.120	3.71E-02
Gsap	gamma-secretase activating protein	17435055	-2.100	2.72E-02
Gsr	glutathione reductase	17500478	-1.004	2.29E-02
Gstk1	glutathione S-transferase kappa 1	17457876	1.100	3.65E-02
Gstm2	glutathione S-transferase mu 2 (muscle)	17409099	1.069	1.77E-02
Gsto1	glutathione S-transferase omega 1	17360216	-1.373	2.75E-02
Gusb	glucuronidase, beta	17453160	-1.142	2.13E-02
Gvin1 (Includes Ot	GTPase, very large interferon inducible 1	17494677	-2.526	8.07E-03
Gzmb	granzyme B	17307033	-2.480	4.51E-02
H2Afx	H2A histone family, member X	17516558	-1.357	1.80E-02
Hcar2	hydroxycarboxylic acid receptor 2	17452705	-4.321	3.11E-03
Hck	HCK proto-oncogene, Src family tyrosine kinase	17377870	-1.641	1.17E-02
Hcls1	hematopoietic cell-specific Lyn substrate 1	17325438	-1.431	1.05E-02
Heatr1	HEAT repeat containing 1	17285157	-1.268	3.70E-03
Hells	helicase, lymphoid-specific	17359212	-1.304	1.87E-02
Hey1	hes-related family bHLH transcription factor with YRPW motif 1	17404011	1.911	3.55E-02
Hist1H2Aa	histone cluster 1, H2aa	17291001	-1.339	3.76E-02
Hist1H2Ac	histone cluster 1, H2ac	17291012	-1.073	3.08E-02
Hist1H2Aj	histone cluster 1, H2aj	17285819	-1.247	3.55E-02
Hist1H2Bd	histone cluster 1, H2bd	17250162	1.606	1.26E-02
Hist1H2Bh	histone cluster 1, H2bh	17285691	-1.095	2.64E-02
Hist1H2Bl	histone cluster 1, H2bl	17290997	-1.169	2.20E-02
Hist1H3G	histone cluster 1, H3g	17408015	1.297	3.02E-02
Hist3H2A	histone cluster 3, H2a	17250178	1.226	6.74E-03
Hk2	hexokinase 2	17468018	-1.193	4.32E-02
Hk3	hexokinase 3 (white cell)	17292775	-2.434	1.81E-02
Hla-Dma	major histocompatibility complex, class II, DM alpha	17336407	-1.350	4.89E-02
Hla-Dqa1	major histocompatibility complex, class II, DQ alpha 1	17343813	-3.469	9.57E-03
Hla-Dqb1	major histocompatibility complex, class II, DQ beta 1	17336494	-3.422	8.07E-03
Hla-Drb5	major histocompatibility complex, class II, DR beta 5	17336502	-2.930	2.58E-02
Hmcn1	hemicentin 1	17227910	2.397	1.80E-02
Hmga1	high mobility group AT-hook 1	17259507	-1.536	4.17E-02
Hmgcs2	3-hydroxy-3-methylglutaryl-CoA synthase 2 (mitochondrial)	17400862	1.935	4.86E-02
Hmox1	heme oxygenase 1	17502573	-1.615	3.15E-02
Hn1	hematological and neurological expressed 1	17271920	-1.073	2.99E-02
Hoxb6	homeobox B6	17255581	1.187	2.64E-02
Hp	haptoglobin	17512809	-3.202	5.76E-03
Hpgd	hydroxyprostaglandin dehydrogenase 15-(NAD)	17501250	1.123	1.53E-02
Hpse	heparanase	17450142	-2.943	3.49E-02
Hs3St3B1	heparan sulfate (glucosamine) 3-O-sulfotransferase 3B1	17264102	-1.608	9.05E-03
Htr7	5-hydroxytryptamine (serotonin) receptor 7, adenylate cyclase-coupled	17364169	-1.283	4.11E-02
Hyal1	hyaluronoglucosaminidase 1	17521428	1.522	3.39E-02
Icos	inducible T-cell co-stimulator	17213490	-2.041	4.43E-02
Ifi16	interferon, gamma-inducible protein 16	17230111	-5.944	6.38E-03
Ifi27L2A/Ifi27L2B	interferon, alpha-inducible protein 27 like 2A	17283549	-2.096	2.40E-02
Ifi30	interferon, gamma-inducible protein 30	17510136	-2.432	6.38E-03
Ifit2	interferon-induced protein with tetratricopeptide repeats 2	17358815	-1.177	3.32E-02
Ifitm1	interferon induced transmembrane protein 1	17484701	-1.123	4.55E-02
Ifitm6	interferon induced transmembrane protein 6	17497724	-1.867	5.16E-03
Ifng	interferon, gamma	17237589	-2.663	4.30E-02
Ifngr2	interferon gamma receptor 2 (interferon gamma transducer 1)	17327084	-1.027	1.17E-02
Igf1R	insulin-like growth factor 1 receptor	17479099	1.560	3.93E-02
Igfbp4	insulin-like growth factor binding protein 4	17256264	-1.884	3.70E-03
Igsf6	immunoglobulin superfamily, member 6	17495839	-2.031	1.96E-02
Igtp	interferon gamma induced GTPase	17249980	-1.264	2.54E-02
Ikbke	inhibitor of kappa light polypeptide gene enhancer in B-cells, kinase epsilon	17226771	-1.904	1.79E-02
Il10Ra	interleukin 10 receptor, alpha	17526492	-1.179	4.01E-02
Il12Rb2	interleukin 12 receptor, beta 2	17467323	-1.809	3.00E-02
Il17A	interleukin 17A	17211369	-2.737	3.52E-02
Il17Ra	interleukin 17 receptor A	17462351	-1.293	3.64E-03
Il17Rd	interleukin 17 receptor D	17298021	1.022	2.46E-02
Il18Rap	interleukin 18 receptor accessory protein	17212252	-2.823	8.07E-03
Il1A	interleukin 1, alpha	17391554	-1.902	8.32E-03
Il1B	interleukin 1, beta	17391565	-4.310	3.25E-03
Il1R2	interleukin 1 receptor, type II	17212174	-2.926	2.82E-02
Il1Rn	interleukin 1 receptor antagonist	17367686	-4.187	1.80E-02
Il4R	interleukin 4 receptor	17482943	-1.433	3.78E-03
Il6	interleukin 6	17435725	-2.727	4.37E-02
Inhba	inhibin, beta A	17285438	-3.680	2.73E-02
Iqgap2	IQ motif containing GTPase activating protein 2	17295136	-1.717	2.78E-02
Irf1	interferon regulatory factor 1	17249593	-1.226	2.37E-02
Irf8	interferon regulatory factor 8	17506279	-1.874	8.53E-03
Irg1	immunoresponsive 1 homolog (mouse)	17302475	-5.200	1.25E-02
Irgm1	immunity-related GTPase family M member 1	17262202	-1.477	2.48E-02
Ism1	isthmin 1, angiogenesis inhibitor	17376908	1.246	3.89E-02
Itga1	integrin, alpha 1	17296388	1.053	2.81E-02
Itga5	integrin, alpha 5 (fibronectin receptor, alpha polypeptide)	17322369	-1.204	1.02E-02
Itgal	integrin, alpha L (antigen CD11A (p180), lymphocyte function-associated antigen 1; alpha polypeptide	17483264	-2.688	1.81E-02
Itgax	integrin, alpha X (complement component 3 receptor 4 subunit)	17483615	-3.078	2.55E-02
Itgb2	integrin, beta 2 (complement component 3 receptor 3 and 4 subunit)	17234647	-2.054	1.42E-02
Itgb7	integrin, beta 7	17322163	-2.077	2.62E-02

Supplementary Table 6. Differentially expressed transcripts in vascular fragments in PBS compared to imatinib treated EAE immunized mice at the progression phase

Gene symbol	Entrez Gene name	Affymetrix ID	Fold change	Adj. pvalue
Itih4	inter-alpha-trypsin inhibitor heavy chain family, member 4	17298267	-3.153	6.74E-03
Itih5	inter-alpha-trypsin inhibitor heavy chain family, member 5	17366670	1.695	4.22E-02
Itm2A	integral membrane protein 2A	17544078	1.315	4.56E-02
Jmy	junction mediating and regulatory protein, p53 cofactor	17294991	1.356	1.31E-02
Kank3	KN motif and ankyrin repeat domains 3	17336175	1.425	2.84E-02
Kcna3	potassium channel, voltage gated shaker related subfamily A, member 3	17401526	-1.788	3.39E-02
Kcnj8	potassium channel, inwardly rectifying subfamily J, member 8	17472530	1.229	3.73E-02
Kcnrg	potassium channel regulator	17301123	1.146	1.09E-02
Kctd12B	potassium channel tetramerisation domain containing 12b	17545488	1.412	1.90E-02
Kdr	kinase insert domain receptor	17448924	1.388	2.81E-02
Kiaa0101	KIAA0101	17518636	-1.191	3.27E-02
Kiaa1217	KIAA1217	17367390	1.266	3.63E-02
Kiaa1524	KIAA1524	17326081	-1.050	3.39E-02
Kif11	kinesin family member 11	17359020	-1.337	4.58E-02
Kif26A	kinesin family member 26A	17279299	1.049	1.24E-02
Kif2C	kinesin family member 2C	17428803	-1.194	1.90E-02
Kitlg	KIT ligand	17236900	1.556	1.94E-02
Klf12	Kruppel-like factor 12	17309099	1.388	2.79E-02
Klhl38	kelch-like family member 38	17317278	1.253	1.34E-02
Klhl4	kelch-like family member 4	17537306	1.328	4.63E-02
Klra2	killer cell lectin-like receptor, subfamily A, member 2	17471828	-3.910	9.42E-03
Klrb1	killer cell lectin-like receptor subfamily B, member 1	17471464	-3.647	1.14E-02
Kmo	kynurenine 3-monooxygenase (kynurenine 3-hydroxylase)	17219789	-1.353	3.73E-02
Kntc1	kinetochore associated 1	17442332	-1.193	1.66E-02
Krt222	keratin 222, type II	17268995	1.069	8.07E-03
Krtap28-13	keratin associated protein 28-13	17548922	1.126	1.06E-02
Lama3	laminin, alpha 3	17348492	1.454	3.63E-02
Lancl3	LanC lantibiotic synthetase component C-like 3 (bacterial)	17533199	1.182	1.02E-02
Layn	layilin	17526956	1.344	3.20E-02
Lcp1	lymphocyte cytosolic protein 1 (L-plastin)	17301968	-1.273	8.07E-03
Lcp2	lymphocyte cytosolic protein 2 (SH2 domain containing leukocyte protein of 76kDa)	17248380	-1.395	1.66E-02
Ldlrad3	low density lipoprotein receptor class A domain containing 3	17388705	1.106	1.45E-02
Lepr	leptin receptor	17415979	1.290	3.37E-02
Letm2	leucine zipper-EF-hand containing transmembrane protein 2	17508380	1.124	2.33E-02
Lgals1	lectin, galactoside-binding, soluble, 1	17312829	-1.600	1.24E-02
Lgals3	lectin, galactoside-binding, soluble, 3	17299329	-2.820	1.42E-02
Lifr	leukemia inhibitory factor receptor alpha	17310044	1.023	1.63E-02
Lilrb3	leukocyte immunoglobulin-like receptor, subfamily B (with TM and ITIM domains), member 3	17485589	-2.180	2.31E-02
Lilrb4	leukocyte immunoglobulin-like receptor, subfamily B (with TM and ITIM domains), member 4	17233226	-5.250	1.51E-03
Litaf	lipopolysaccharide-induced TNF factor	17328124	-1.148	8.07E-03
Lmnb1	lamin B1	17350740	-1.559	3.12E-02
Lmo7	LIM domain 7	17302429	1.737	2.04E-02
Lnx2	ligand of numb-protein X 2	17455281	1.446	1.28E-02
Loc102635638	uncharacterized LOC102635638	17517215	1.197	3.08E-02
Loc102638993	uncharacterized LOC102638993	17480924	-2.713	2.75E-02
Lpar4	lysophosphatidic acid receptor 4	17537112	1.084	1.81E-02
Lpl	lipoprotein lipase	17501633	-1.276	1.83E-02
Lrp12	low density lipoprotein receptor-related protein 12	17316754	-1.250	1.64E-02
Lrrc25	leucine rich repeat containing 25	17501989	-1.530	4.40E-02
Lrrc49	leucine rich repeat containing 49	17527883	1.449	1.07E-02
Lrrn3	leucine rich repeat neuronal 3	17280888	2.050	3.05E-02
Ltb4R	leukotriene B4 receptor	17300666	-2.475	2.23E-02
Ltc4S	leukotriene C4 synthase	17262316	1.350	1.37E-02
Ly6A (Includes Oth	lymphocyte antigen 6 complex, locus A	17318076	-3.682	3.60E-02
Lyz	lysozyme	17245223	-1.391	8.07E-03
Maob	monoamine oxidase B	17540378	1.312	4.01E-02
Map4K1	mitogen-activated protein kinase kinase kinase kinase 1	17476036	-1.621	3.08E-02
Map4K2	mitogen-activated protein kinase kinase kinase kinase 2	17356811	1.051	2.62E-02
Mapkapk2	mitogen-activated protein kinase-activated protein kinase 2	17226736	-1.236	8.35E-03
Mapkapk3	mitogen-activated protein kinase-activated protein kinase 3	17530863	-1.257	4.55E-02
Mast4	microtubule associated serine/threonine kinase family member 4	17295817	1.084	4.12E-02
Mb21D1	Mab-21 domain containing 1	17529046	-1.284	7.31E-03
Mcemp1	mast cell-expressed membrane protein 1	17498730	-3.648	1.09E-02
Mcf2L	MCF.2 cell line derived transforming sequence-like	17499155	1.095	4.00E-02
Mcm3	minichromosome maintenance complex component 3	17221633	-1.143	1.39E-02
Mcm5	minichromosome maintenance complex component 5	17502583	-1.105	3.45E-02
Mcoln2	mucolipin 2	17403490	-1.024	3.05E-02
Mecom	MDS1 and EVI1 complex locus	17404478	1.092	3.69E-02
Mefv	Mediterranean fever	17327580	-2.429	3.25E-03
Met	MET proto-oncogene, receptor tyrosine kinase	17456176	-2.293	2.70E-02
Mgll	monoglyceride lipase	17460665	1.065	2.78E-02
Micb	MHC class I polypeptide-related sequence B	17474363	1.449	3.95E-02
Mif	macrophage migration inhibitory factor (glycosylation-inhibiting factor)	17241962	-1.295	9.05E-03
Milr1	mast cell immunoglobulin like receptor 1	17257599	-1.997	7.47E-03
Mir-181	microRNA 181a-1	17370485	1.042	2.36E-02
Mir-194	microRNA 194-1	17220529	1.025	3.05E-02
Mir-1957	microRNA 1957a	17417858	1.054	4.75E-02
Mir1192	microRNA 1192	17358219	1.379	1.81E-02
Mirt2	myocardial infraction associated transcript 2	17318422	-2.237	4.64E-02
Mki67	marker of proliferation Ki-67	17497334	-1.263	4.57E-02
Mmp14	matrix metallopeptidase 14 (membrane-inserted)	17300279	-2.462	1.17E-02

Supplementary Table 6. Differentially expressed transcripts in vascular fragments in PBS compared to imatinib treated EAE immunized mice at the progression phase

Gene symbol	Entrez Gene name	Affymetrix ID	Fold change	Adj. pvalue
Mmp15	matrix metallopeptidase 15 (membrane-inserted)	17504293	1.111	4.40E-02
Mmp19	matrix metallopeptidase 19	17238558	-2.411	2.35E-02
Mmp28	matrix metallopeptidase 28	17266911	1.043	1.78E-02
Mmp8	matrix metallopeptidase 8	17514553	-3.247	1.53E-02
Mrps18B	mitochondrial ribosomal protein S18B	17385483	-1.004	1.17E-02
Ms4A4A	membrane-spanning 4-domains, subfamily A, member 4A	17357640	-2.761	1.47E-02
Ms4A4B (Includes	membrane-spanning 4-domains, subfamily A, member 4B	17357648	-3.215	1.94E-02
Ms4A6A	membrane-spanning 4-domains, subfamily A, member 6A	17362973	-2.627	7.71E-03
Ms4A6B	membrane-spanning 4-domains, subfamily A, member 6B	17357688	-2.115	7.31E-03
Ms4A6C	membrane-spanning 4-domains, subfamily A, member 6C	17357671	-3.148	6.38E-03
Ms4A8	membrane-spanning 4-domains, subfamily A, member 8	17362874	-3.742	2.42E-02
Msr1	macrophage scavenger receptor 1	17508850	-4.812	4.10E-03
Msrb1	methionine sulfoxide reductase B1	17334419	-1.563	4.16E-02
Mthfd2	methylenetetrahydrofolate dehydrogenase (NADP+ dependent) 2	17468143	-1.280	6.48E-03
Mturn	maturin, neural progenitor differentiation regulator homolog (Xenopus)	17458752	1.101	1.59E-02
Mvd	mevalonate (diphospho) decarboxylase	17513681	-1.075	2.87E-02
Mx1/Mx2	MX dynamin-like GTPase 1	17332531	-1.428	4.90E-02
Mxd1	MAX dimerization protein 1	17468511	-1.552	2.93E-02
Mxd4	MAX dimerization protein 4	17447218	1.173	1.15E-02
Myc	v-myc avian myelocytomatosis viral oncogene homolog	17311846	-1.095	1.16E-02
Myct1	myc target 1	17231477	1.339	2.22E-02
Myd88	myeloid differentiation primary response 88	17532137	-1.094	1.23E-02
Mylpf	myosin light chain, phosphorylatable, fast skeletal muscle	17483242	1.125	3.20E-02
Myo10	myosin X	17310589	1.125	2.28E-02
Myo1D	myosin ID	17266698	1.117	3.13E-02
Myo1F	myosin IF	17336114	-1.175	4.55E-02
Myo1G	myosin IG	17260369	-1.716	4.46E-02
Myo5A	myosin VA	17519394	-2.152	2.30E-02
Naaa	N-acylethanolamine acid amidase	17449673	-2.690	4.03E-03
Nabp1	nucleic acid binding protein 1	17222825	-1.523	1.88E-02
Napsa	napsin A aspartic peptidase	17477508	-2.268	7.71E-03
Ncf4	neutrophil cytosolic factor 4, 40kDa	17312700	-1.742	9.36E-03
Ndc80	NDC80 kinetochore complex component	17347042	-1.576	4.37E-02
Ndnf	neuron-derived neurotrophic factor	17459207	1.663	3.05E-02
Neurl2	neuralized E3 ubiquitin protein ligase 2	17394292	1.323	1.23E-02
Nfib	nuclear factor I/B	17426765	1.096	2.44E-02
Nfil3	nuclear factor, interleukin 3 regulated	17292634	-1.861	5.76E-03
Nhp2	NHP2 ribonucleoprotein	17249347	-1.244	4.79E-03
Nid2	nidogen 2 (osteonidogen)	17297391	1.238	1.06E-02
Ninj1	ninjurin 1	17287107	-1.337	1.92E-02
Nlrc5	NLR family, CARD domain containing 5	17504023	-1.476	3.20E-02
Nlrp3	NLR family, pyrin domain containing 3	17250249	-1.791	1.07E-02
Noc4L	nucleolar complex associated 4 homolog	17451043	-1.600	8.40E-03
Nostrin	nitric oxide synthase trafficking	17371390	1.371	2.04E-02
Npl	N-acetylneuraminate pyruvate lyase (dihydrodipicolinate synthase)	17228234	-1.247	1.10E-02
Nr1D1	nuclear receptor subfamily 1, group D, member 1	17268884	1.924	1.55E-02
Nr3C2	nuclear receptor subfamily 3, group C, member 2	17502626	1.260	2.51E-02
Nrg1	neuregulin 1	17508609	-1.982	3.02E-02
Nrxn1	neurexin 1	17348016	1.057	2.46E-02
Nt5E	5'-nucleotidase, ecto (CD73)	17520073	-2.648	7.71E-03
Nubp1	nucleotide binding protein 1	17322829	-1.009	3.12E-02
Nuf2	NUF2, NDC80 kinetochore complex component	17229433	-1.465	3.29E-02
Nup210	nucleoporin 210kDa	17468961	-1.210	2.75E-02
Nxpe4	neurexophilin and PC-esterase domain family, member 4	17516978	1.268	3.64E-02
Oas3	2'-5'-oligoadenylate synthetase 3, 100kDa	17452070	-2.485	1.61E-02
Ocln	occludin	17295670	1.267	3.57E-02
Olfr33	olfactory receptor 33	17494126	1.108	3.74E-02
Olr1	oxidized low density lipoprotein (lectin-like) receptor 1	17471550	-3.966	3.15E-02
Osm	oncostatin M	17246803	-2.811	6.48E-03
P2Rx4	purinergic receptor P2X, ligand gated ion channel, 4	17442149	-1.242	3.32E-02
P2Ry10	purinergic receptor P2Y, G-protein coupled, 10	17537118	-1.812	3.08E-02
P2Ry6	pyrimidinergic receptor P2Y, G-protein coupled, 6	17493869	-1.185	2.17E-02
Pbk	PDZ binding kinase	17301428	-1.325	1.90E-02
Pdcd1Lg2	programmed cell death 1 ligand 2	17358552	-2.029	3.57E-02
Pde2A	phosphodiesterase 2A, cGMP-stimulated	17480880	1.711	2.06E-02
Pdgfb	platelet-derived growth factor beta polypeptide	17319380	1.081	4.67E-02
Peg13	paternally expressed 13	17317835	1.321	3.02E-02
Per3	period circadian clock 3	17433328	1.009	2.78E-02
Pfkp	phosphofructokinase, platelet	17290324	-1.489	4.04E-02
Pgd	phosphogluconate dehydrogenase	17433040	-1.731	1.14E-02
Pgk1	phosphoglycerate kinase 1	17537088	-1.473	2.08E-02
Pgm5	phosphoglucomutase 5	17363626	1.818	3.74E-02
Phactr1	phosphatase and actin regulator 1	17286838	1.020	3.74E-02
Phf11	PHD finger protein 11	17307280	-2.102	1.23E-02
Pik3Ap1	phosphoinositide-3-kinase adaptor protein 1	17364642	-1.561	6.74E-03
Pilra	paired immunoglobin-like type 2 receptor alpha	17454166	-1.538	1.35E-02
Pilrb	paired immunoglobin-like type 2 receptor beta	17454179	-1.813	2.19E-02
Pim1	Pim-1 proto-oncogene, serine/threonine kinase	17335540	-2.409	9.56E-03
Pla2G7	phospholipase A2, group VII (platelet-activating factor acetylhydrolase, plasma)	17337796	-2.076	3.54E-02
Plac8	placenta-specific 8	17450121	-3.285	3.97E-03
Plaur	plasminogen activator, urokinase receptor	17474974	-2.162	1.77E-02

Supplementary Table 6. Differentially expressed transcripts in vascular fragments in PBS compared to imatinib treated EAE immunized mice at the progression phase

Gene symbol	Entrez Gene name	Affymetrix ID	Fold change	Adj. pvalue
Plbd1	phospholipase B domain containing 1	17472114	-3.972	5.57E-03
Plek	pleckstrin	17260761	-1.454	8.74E-03
Plekhh2	pleckstrin homology domain containing, family H (with MyTH4 domain) member 2	17340050	1.377	1.37E-02
Plekho2	pleckstrin homology domain containing, family O member 2	17528274	-2.111	4.10E-03
Plet1	placenta expressed transcript 1	17517097	-1.844	2.28E-02
Plin2	perilipin 2	17426981	-1.526	3.74E-02
Plk1	polo-like kinase 1	17482739	-1.686	3.00E-02
Plk3	polo-like kinase 3	17428766	-1.011	1.56E-02
Pllp	plasmolipin	17511878	1.788	3.31E-02
Plscr4	phospholipid scramblase 4	17520353	1.362	4.79E-02
Pltp	phospholipid transfer protein	17394297	1.461	4.33E-02
Plxna4	plexin A4	17465636	1.042	2.55E-02
Pmf1/Pmf1-Bglap	polyamine-modulated factor 1	17406745	-1.219	4.16E-02
Pnpla6	patatin-like phospholipase domain containing 6	17498625	1.320	2.36E-02
Polk	polymerase (DNA directed) kappa	17295212	1.447	3.39E-02
Pot1	protection of telomeres 1	17465246	1.000	1.05E-02
Ppa1	pyrophosphatase (inorganic) 1	17233720	-1.248	1.42E-02
Ppp1R14B	protein phosphatase 1, regulatory (inhibitor) subunit 14B	17356987	-1.384	4.03E-03
Ppp1R16B	protein phosphatase 1, regulatory subunit 16B	17378942	1.077	2.28E-02
Ppp1R3B	protein phosphatase 1, regulatory subunit 3B	17500543	-1.093	4.01E-02
Prdx5	peroxiredoxin 5	17362101	-1.387	2.58E-02
Prickle1	prickle homolog 1	17320720	1.660	1.92E-02
Prom1	prominin 1	17447835	1.723	2.16E-02
Proser2	proline and serine rich 2	17381448	1.507	1.49E-02
Prrg1	proline rich Gla (G-carboxyglutamic acid) 1	17542948	1.226	1.72E-02
Prss23	protease, serine, 23	17493212	1.597	3.45E-02
Prss23Os	protease, serine 23, opposite strand	17480044	1.815	4.16E-02
Psat1	phosphoserine aminotransferase 1	17363204	-1.164	2.97E-02
Psmb8	proteasome subunit beta 8	17336446	-1.048	5.00E-02
Pstpip1	proline-serine-threonine phosphatase interacting protein 1	17517554	-1.826	2.22E-02
Pstpip2	proline-serine-threonine phosphatase interacting protein 2	17352036	-2.185	4.96E-02
Ptger4	prostaglandin E receptor 4 (subtype EP4)	17315718	-1.151	1.04E-02
Ptk2B	protein tyrosine kinase 2 beta	17307860	-1.308	3.45E-02
Ptn	pleiotrophin	17465856	1.636	4.21E-02
Ptpn1	protein tyrosine phosphatase, non-receptor type 1	17379960	-1.336	1.09E-02
Ptpn6	protein tyrosine phosphatase, non-receptor type 6	17470796	-1.532	1.78E-02
Ptprc	protein tyrosine phosphatase, receptor type, C	17227536	-1.545	8.07E-03
Ptprm	protein tyrosine phosphatase, receptor type, M	17346856	1.162	4.06E-02
Ptprz1	protein tyrosine phosphatase, receptor-type, Z polypeptide 1	17456381	1.240	4.85E-02
Pycard	PYD and CARD domain containing	17496839	-1.479	2.05E-02
Pygl	phosphorylase, glycogen, liver	17281721	-1.852	2.62E-02
Rab11Fip1	RAB11 family interacting protein 1 (class I)	17508523	1.063	1.02E-02
Rab20	RAB20, member RAS oncogene family	17507435	-2.022	1.86E-02
Rab26Os	RAB26, member RAS oncogene family, opposite strand	17334302	-1.298	2.85E-02
Rab27A	RAB27A, member RAS oncogene family	17519282	-1.020	2.03E-02
Rab32	RAB32, member RAS oncogene family	17239227	-1.911	1.45E-02
Rab6B	RAB6B, member RAS oncogene family	17520922	1.359	3.20E-02
Rac2	ras-related C3 botulinum toxin substrate 2 (rho family, small GTP binding protein Rac2)	17319037	-2.378	3.64E-03
Ralgds	ral guanine nucleotide dissociation stimulator	17368685	-1.149	1.07E-02
Rap2C	RAP2C, member of RAS oncogene family	17541612	-1.130	1.12E-02
Rapgef4Os3	Rap guanine nucleotide exchange factor (GEF) 4, opposite strand 3	17386451	1.286	1.78E-02
Rasgrp2	RAS guanyl releasing protein 2 (calcium and DAG-regulated)	17356897	1.086	1.90E-02
Rasgrp3	RAS guanyl releasing protein 3 (calcium and DAG-regulated)	17339772	1.094	3.45E-02
Rbm38	RNA binding motif protein 38	17380199	-1.001	2.81E-02
Rbp1	retinol binding protein 1, cellular	17520624	1.196	1.14E-02
Rbpj	recombination signal binding protein for immunoglobulin kappa J region	17466505	-1.097	1.66E-02
Rgs12	regulator of G-protein signaling 12	17436761	1.455	1.28E-02
Rhobtb1	Rho-related BTB domain containing 1	17234042	1.108	1.88E-02
Rhoh	ras homolog family member H	17437830	-1.151	1.37E-02
Rhpn2	rhophilin, Rho GTPase binding protein 2	17476752	1.339	2.74E-02
Rinl	Ras and Rab interactor-like	17475942	-1.137	4.06E-02
Ripk3	receptor-interacting serine-threonine kinase 3	17306906	-1.210	4.06E-02
Rnasel	ribonuclease L (2',5'-oligoisoadenylate synthetase-dependent)	17218328	-1.654	4.19E-02
Rnd1	Rho family GTPase 1	17321307	-1.149	3.71E-02
Rnf149	ring finger protein 149	17222549	-1.514	3.76E-02
Rnf19B	ring finger protein 19B	17418916	-1.348	3.65E-02
Rnh1	ribonuclease/angiogenin inhibitor 1	17497769	-1.213	3.97E-02
Rnu3A	U3A small nuclear RNA	17232731	-1.238	4.61E-02
Rpf2	ribosome production factor 2 homolog	17240342	-1.224	8.07E-03
Rpl3	ribosomal protein L3	17319403	-1.159	6.74E-03
Rpl6	ribosomal protein L6	17441799	-1.067	3.44E-02
Rps11	ribosomal protein S11	17490599	1.291	3.93E-02
Rrm2	ribonucleotide reductase M2	17274540	-1.218	2.62E-02
Rrp12	ribosomal RNA processing 12 homolog	17364725	-1.077	7.94E-03
Rtp3	receptor transporter protein 3	17531693	1.168	8.07E-03
Runx1	runt-related transcription factor 1	17332236	-1.079	2.58E-02
Runx3	runt-related transcription factor 3	17419840	-1.105	1.81E-02
S100A4	S100 calcium binding protein A4	17399802	-1.977	5.76E-03
S100A6	S100 calcium binding protein A6	17399812	-1.194	3.11E-02
Saa3	serum amyloid A 3	17491193	-5.821	1.51E-03
Samd12	sterile alpha motif domain containing 12	17317031	1.508	2.34E-02

Supplementary Table 6. Differentially expressed transcripts in vascular fragments in PBS compared to imatinib treated EAE immunized mice at the progression phase

Gene symbol	Entrez Gene name	Affymetrix ID	Fold change	Adj. pvalue
Scgb3A1	secretoglobin, family 3A, member 1	17249028	-2.008	3.03E-02
Scimp	SLP adaptor and CSK interacting membrane protein	17265386	-3.830	6.74E-03
Scpep1	serine carboxypeptidase 1	17267601	-1.468	4.94E-02
Sdad1	SDA1 domain containing 1	17449685	-1.260	1.12E-02
Sdc1	syndecan 1	17273948	-1.966	1.39E-02
Sdf2L1	stromal cell-derived factor 2-like 1	17328625	-1.198	8.50E-03
Sdpr	serum deprivation response	17212719	1.914	1.96E-02
Sell	selectin L	17218835	-3.442	3.25E-03
Selp	selectin P (granule membrane protein 140kDa, antigen CD62)	17218845	-3.416	3.64E-03
Sema4A	semaphorin 4A	17406760	-2.675	2.20E-02
Serp1	stress-associated endoplasmic reticulum protein 1	17405414	-1.024	1.08E-02
Sesn1	sestrin 1	17232912	1.076	3.30E-02
Sgpp2	sphingosine-1-phosphate phosphatase 2	17214665	2.204	2.22E-02
Sh3Bgrl	SH3 domain binding glutamate-rich protein like	17537199	-2.426	2.22E-02
Sh3Bp2	SH3-domain binding protein 2	17436607	-1.592	2.28E-02
Shcbp1	SHC SH2-domain binding protein 1	17507288	-1.770	3.95E-02
She	Src homology 2 domain containing E	17399533	1.158	4.61E-02
Shmt2	serine hydroxymethyltransferase 2 (mitochondrial)	17245902	-1.274	4.10E-03
Siglec1	sialic acid binding Ig-like lectin 1, sialoadhesin	17391834	-2.545	2.21E-02
Sirpb1	signal-regulatory protein beta 1	17404209	-3.626	7.60E-03
Sla	Src-like-adaptor	17317637	-1.774	1.28E-02
Slamf6	SLAM family member 6	17219435	-1.016	4.10E-02
Slamf7	SLAM family member 7	17229782	-4.549	2.50E-02
Slamf8	SLAM family member 8	17229931	-3.235	2.09E-02
Slc10A6	solute carrier family 10 (sodium/bile acid cotransporter), member 6	17450319	-1.497	4.16E-02
Slc15A3	solute carrier family 15 (oligopeptide transporter), member 3	17357597	-2.777	3.02E-03
Slc16A1	solute carrier family 16 (monocarboxylate transporter), member 1	17401335	1.347	3.45E-02
Slc16A10	solute carrier family 16 (aromatic amino acid transporter), member 10	17240330	-1.444	1.36E-02
Slc16A3	solute carrier family 16 (monocarboxylate transporter), member 3	17259534	-1.588	2.10E-02
Slc16A4	solute carrier family 16, member 4	17401563	1.752	2.78E-02
Slc19A3	solute carrier family 19 (thiamine transporter), member 3	17224942	1.989	3.08E-02
Slc1A5	solute carrier family 1 (neutral amino acid transporter), member 5	17474143	-1.034	2.77E-02
Slc26A10	solute carrier family 26, member 10	17245729	1.959	8.07E-03
Slc2A5	solute carrier family 2 (facilitated glucose/fructose transporter), member 5	17421875	1.209	1.24E-02
Slc2A6	solute carrier family 2 (facilitated glucose transporter), member 6	17383216	-1.477	2.45E-02
Slc35F2	solute carrier family 35, member F2	17517349	1.198	2.03E-02
Slc38A1	solute carrier family 38, member 1	17320907	-1.277	9.42E-03
Slc38A3	solute carrier family 38, member 3	17530967	1.443	4.03E-02
Slc39A12	solute carrier family 39 (zinc transporter), member 12	17367139	1.072	4.71E-02
Slc39A14	solute carrier family 39 (zinc transporter), member 14	17308299	-1.478	7.06E-03
Slc40A1	solute carrier family 40 (iron-regulated transporter), member 1	17222777	1.998	2.26E-02
Slc43A3	solute carrier family 43, member 3	17372644	-1.413	1.09E-02
Slc7A1	solute carrier family 7 (cationic amino acid transporter, y+ system), member 1	17455401	1.012	1.39E-02
Slc7A11	solute carrier family 7 (anionic amino acid transporter light chain, xc- system), member 11	17405082	-2.936	1.19E-02
Slc7A2	solute carrier family 7 (cationic amino acid transporter, y+ system), member 2	17500716	-2.050	2.84E-02
Slc7A5	solute carrier family 7 (amino acid transporter light chain, L system), member 5	17513641	1.207	2.75E-02
Slc7A8	solute carrier family 7 (amino acid transporter light chain, L system), member 8	17306477	-1.177	2.51E-02
Slc8B1	solute carrier family 8 (sodium/lithium/calcium exchanger), member B1	17441671	-1.017	2.84E-02
Slc9A3R2	solute carrier family 9, subfamily A (NHE3, cation proton antiporter 3), member 3 regulator 2	17341963	1.231	3.55E-02
Slco2A1	solute carrier organic anion transporter family, member 2A1	17520905	-1.473	1.34E-02
Slfn1	schlafen 1	17254171	-4.422	9.56E-03
Slfn12L	schlafen family member 12-like	17254176	-4.252	1.53E-02
Slfn13	schlafen family member 13	17266851	-2.254	9.20E-03
Slfn2	schlafen 2	17254166	-1.452	3.48E-02
Slpi	secretory leukocyte peptidase inhibitor	17394153	-3.443	1.09E-02
Smarca2	SWI/SNF related, matrix associated, actin dependent regulator of chromatin, subfamily a, member 2	17358375	1.179	2.79E-02
Smco4	single-pass membrane protein with coiled-coil domains 4	17514841	1.041	2.23E-02
Snai2	snail family zinc finger 2	17323192	1.562	2.50E-02
Sncaip	synuclein, alpha interacting protein	17350591	1.057	3.23E-02
Sned1	sushi, nidogen and EGF-like domains 1	17215932	1.190	4.94E-02
Snhg4	small nucleolar RNA host gene 4	17349549	-1.933	8.07E-03
Snora20	small nucleolar RNA, H/ACA box 20	17333344	-1.904	2.62E-02
Snora73B	small nucleolar RNA, H/ACA box 73b	17430831	-1.377	2.83E-02
Snord16A	small nucleolar RNA, C/D box 16A	17518312	-1.285	2.32E-02
Snord35B	small nucleolar RNA, C/D box 35B	17490606	3.026	1.80E-02
Snord91A	small nucleolar RNA, C/D box 91A	17252845	1.252	3.39E-02
Snrk	SNF related kinase	17523398	1.224	2.39E-02
Snx20	sorting nexin 20	17511534	-1.211	4.30E-02
Soat1	sterol O-acyltransferase 1	17228544	-1.741	2.03E-02
Soat2	sterol O-acyltransferase 2	17315312	-1.334	5.56E-03
Socs1	suppressor of cytokine signaling 1	17328104	-2.177	3.03E-02
Socs3	suppressor of cytokine signaling 3	17272619	-1.226	1.03E-02
Sod2	superoxide dismutase 2, mitochondrial	17333347	-1.540	1.18E-02
Sorl1	sortilin-related receptor, L(DLR class) A repeats containing	17525894	-1.142	1.81E-02
Sox17	SRY (sex determining region Y)-box 17	17221071	1.376	2.80E-02
Sox18	SRY (sex determining region Y)-box 18	17395928	1.146	1.88E-02
Sp110	SP110 nuclear body protein	17366271	-1.368	2.58E-02
Spi1	Spi-1 proto-oncogene	17373177	-1.419	1.50E-02
Spint1	serine peptidase inhibitor, Kunitz type 1	17374765	-1.654	5.76E-03
Spock2	sparc/osteonectin, cwcv and kazal-like domains proteoglycan (testican) 2	17233613	1.707	3.65E-02
Spp1	secreted phosphoprotein 1	17439830	-3.729	1.76E-02

Supplementary Table 6. Differentially expressed transcripts in vascular fragments in PBS compared to imatinib treated EAE immunized mice at the progression phase

Gene symbol	Entrez Gene name	Affymetrix ID	Fold change	Adj. pvalue
Srgap2	SLIT-ROBO Rho GTPase activating protein 2	17226798	1.080	1.09E-02
Srm	spermidine synthase	17421694	-1.011	1.45E-02
St14	suppression of tumorigenicity 14 (colon carcinoma)	17525240	-1.307	9.59E-03
St6Galnac3	ST6 (alpha-N-acetyl-neuraminyl-2,3-beta-galactosyl-1,3)-N-acetylgalactosaminide alpha-2,6-sialyltrar	17411262	1.462	8.07E-03
St8Sia4	ST8 alpha-N-acetyl-neuraminide alpha-2,8-sialyltransferase 4	17225815	1.388	1.45E-02
St8Sia6	ST8 alpha-N-acetyl-neuraminide alpha-2,8-sialyltransferase 6	17381879	1.388	2.54E-02
Stard13	StAR-related lipid transfer (START) domain containing 13	17455578	1.345	4.40E-02
Stard8	StAR-related lipid transfer (START) domain containing 8	17536463	1.182	1.12E-02
Stat1	signal transducer and activator of transcription 1, 91kDa	17212750	-1.139	3.05E-02
Stfa2/Stfa2L1	stefin A2	17325324	-4.055	1.28E-02
Stmn2	stathmin 2	17396024	1.733	1.84E-02
Stra6	stimulated by retinoic acid 6	17517848	1.306	3.60E-02
Sulf2	sulfatase 2	17394538	-1.619	1.24E-02
Sult1A1	sulfotransferase family 1A, phenol-preferring, member 1	17496354	1.083	4.83E-02
Syne1	spectrin repeat containing, nuclear envelope 1	17238938	1.306	4.23E-02
Syne2	spectrin repeat containing, nuclear envelope 2	17276396	1.172	4.17E-02
Tacc3	transforming, acidic coiled-coil containing protein 3	17436457	-1.202	2.89E-02
Taf1D	TATA box binding protein (TBP)-associated factor, RNA polymerase I, D, 41kDa	17514834	-1.688	1.14E-02
Tap1	transporter 1, ATP-binding cassette, sub-family B (MDR/TAP)	17336432	-1.789	2.36E-02
Tarm1	T cell-interacting, activating receptor on myeloid cells 1	17485510	-4.139	1.27E-02
Tcf15	transcription factor 15 (basic helix-loop-helix)	17377672	1.214	2.20E-02
Tdrp	testis development related protein	17507673	1.227	1.40E-02
Tecrl	trans-2,3-enoyl-CoA reductase-like	17449108	2.604	1.59E-02
Tek	TEK tyrosine kinase, endothelial	17415469	1.336	4.70E-02
Tfec	transcription factor EC	17464950	-2.736	2.64E-02
Tfrc	transferrin receptor	17324835	1.408	1.42E-02
Tgfb2	transforming growth factor, beta 2	17230830	1.585	2.40E-02
Tgfbi	transforming growth factor, beta-induced, 68kDa	17287827	-3.503	7.06E-03
Tgm1	transglutaminase 1	17306816	-1.436	1.28E-02
Thbs1	thrombospondin 1	17374488	-3.740	1.51E-03
Themis2	thymocyte selection associated family member 2	17430906	-1.734	3.98E-02
Thra	thyroid hormone receptor, alpha	17256138	1.065	1.94E-02
Thsd1	thrombospondin, type I, domain containing 1	17499874	1.210	2.00E-02
Thy1	Thy-1 cell surface antigen	17516462	-2.146	4.68E-02
Tie1	tyrosine kinase with immunoglobulin-like and EGF-like domains 1	17429206	1.408	2.38E-02
Timm8A	translocase of inner mitochondrial membrane 8 homolog A (yeast)	17231676	-1.228	3.78E-03
Timp1	TIMP metallopeptidase inhibitor 1	17533713	-3.325	5.57E-03
Tlr1	toll-like receptor 1	17448245	-1.477	2.33E-02
Tlr2	toll-like receptor 2	17406279	-1.705	5.57E-03
Tma16	translation machinery associated 16 homolog	17509697	-1.085	1.49E-02
Tmc7	transmembrane channel-like 7	17495566	1.752	2.24E-02
Tmem104	transmembrane protein 104	17258140	-1.110	4.21E-02
Tmem106A	transmembrane protein 106A	17256784	-2.069	2.00E-02
Tmem156	transmembrane protein 156	17448256	-1.120	4.48E-02
Tmem173	transmembrane protein 173	17353663	-1.394	4.36E-03
Tmem253	transmembrane protein 253	17299703	1.038	2.79E-02
Tmem44	transmembrane protein 44	17329675	1.166	3.19E-02
Tmtc1	transmembrane and tetratricopeptide repeat containing 1	17472955	1.219	3.81E-02
Tmtc2	transmembrane and tetratricopeptide repeat containing 2	17244661	1.564	2.61E-02
Tnfa	tumor necrosis factor alpha	17344309	-2.625	1.51E-03
Tnfaip2	tumor necrosis factor, alpha-induced protein 2	17279131	-2.557	3.48E-02
Tnfrsf17	tumor necrosis factor receptor superfamily, member 17	17322944	1.458	1.05E-02
Tnfrsf19	tumor necrosis factor receptor superfamily, member 19	17307433	1.669	3.03E-02
Tnfrsf26	tumor necrosis factor receptor superfamily, member 26	17498301	-2.136	4.80E-02
Tnfrsf9	tumor necrosis factor receptor superfamily, member 9	17421981	-2.042	9.50E-03
Tnfsf8	tumor necrosis factor (ligand) superfamily, member 8	17426356	-1.569	5.57E-03
Tnfsf9	tumor necrosis factor (ligand) superfamily, member 9	17338959	-1.273	2.88E-02
Tob1	transducer of ERBB2, 1	17255210	1.158	1.92E-02
Top2A	topoisomerase (DNA) II alpha	17268909	-1.740	1.17E-02
Tp53Inp1	tumor protein p53 inducible nuclear protein 1	17411751	1.226	2.22E-02
Tpd52L1	tumor protein D52-like 1	17240089	1.389	2.01E-02
Tpi1	triosephosphate isomerase 1	17470879	-1.418	1.83E-02
Trafd1	TRAF-type zinc finger domain containing 1	17452178	-1.018	3.76E-02
Trav12N-2	T cell receptor alpha variable 12N-2	17300225	-1.799	2.88E-02
Trav14-1	T cell receptor alpha variable 14-1	17300165	-2.447	1.17E-02
Trbv1	T cell receptor beta, variable 1	17457804	-2.813	3.44E-02
Trem1	triggering receptor expressed on myeloid cells 1	17338364	-2.945	1.59E-02
Treml2	triggering receptor expressed on myeloid cells-like 2	17338388	-2.366	9.05E-03
Trex1	three prime repair exonuclease 1	17531260	-1.430	1.73E-02
Trib2	tribbles pseudokinase 2	17280054	1.053	1.67E-02
Tril	TLR4 interactor with leucine-rich repeats	17466932	1.919	1.17E-02
Trim30B	tripartite motif-containing 30B	17494370	-1.595	2.84E-02
Trim30C	tripartite motif-containing 30C	17494386	-2.605	1.07E-02
Trim59	tripartite motif containing 59	17405789	-1.053	3.12E-02
Trip13	thyroid hormone receptor interactor 13	17294302	-1.125	2.09E-02
Tshz1	teashirt zinc finger homeobox 1	17355790	1.091	1.35E-02
Tspan13	tetraspanin 13	17280749	1.130	3.49E-02
Tsr1	TSR1, 20S rRNA accumulation, homolog (S. cerevisiae)	17252827	-1.074	8.98E-03
Ttc39B	tetratricopeptide repeat domain 39B	17426855	-1.633	1.05E-02
Tuba4A	tubulin, alpha 4a	17224540	-1.429	3.23E-02
Tubb6	tubulin, beta 6 class V	17351465	-2.001	2.84E-03

Supplementary Table 6. Differentially expressed transcripts in vascular fragments in PBS compared to imatinib treated EAE immunized mice at the progression phase

Gene symbol	Entrez Gene name	Affymetrix ID	Fold change	Adj. pvalue
Ubd	ubiquitin D	17337545	-3.128	3.63E-02
Ube2C	ubiquitin-conjugating enzyme E2C	17379523	-1.482	2.48E-02
Uck2	uridine-cytidine kinase 2	17229391	-1.436	1.28E-02
Ugcg	UDP-glucose ceramide glucosyltransferase	17414434	-1.085	9.36E-03
Uhrf1	ubiquitin-like with PHD and ring finger domains 1	17338747	-1.722	7.47E-03
Unc13B	unc-13 homolog B (C. elegans)	17413221	1.263	2.74E-02
Upk1B	uroplakin 1B	17330427	1.289	4.79E-03
Upp1	uridine phosphorylase 1	17247225	-3.142	3.22E-03
Ushbp1	Usher syndrome 1C binding protein 1	17510295	1.387	1.88E-02
Utrn	utrophin	17239268	1.273	4.23E-02
Vav1	vav 1 guanine nucleotide exchange factor	17338982	-1.187	1.32E-02
Vav3	vav 3 guanine nucleotide exchange factor	17401846	-1.211	4.67E-02
Vcan	versican	17294738	-1.686	1.51E-03
Vdr	vitamin D (1,25- dihydroxyvitamin D3) receptor	17321078	-2.241	1.37E-02
Vegfa	vascular endothelial growth factor A	17345293	-2.271	3.57E-02
Vegfc	vascular endothelial growth factor C	17501160	1.752	3.39E-02
Vmn1R180 (Includ	vomeronasal 1 receptor 180	17487570	1.055	2.16E-02
Vwa3A	von Willebrand factor A domain containing 3A	17482508	1.437	9.05E-03
Wars	tryptophanyl-tRNA synthetase	17283930	-1.078	3.82E-02
Wdhd1	WD repeat and HMG-box DNA binding protein 1	17305757	-1.270	1.11E-02
Wdr74	WD repeat domain 74	17357150	-1.039	2.09E-02
Wfdc1	WAP four-disulfide core domain 1	17506137	1.519	2.89E-02
Wfdc17	WAP four-disulfide core domain 17	17254289	-3.716	6.74E-03
Wfdc21	WAP four-disulfide core domain 21	17254300	-1.660	4.23E-02
Yae1D1	Yae1 domain containing 1	17290823	1.145	4.20E-02
Zak	sterile alpha motif and leucine zipper containing kinase AZK	17371912	1.025	2.94E-02
Zbp1	Z-DNA binding protein 1	17395079	-3.603	1.03E-02
Zbtb20	zinc finger and BTB domain containing 20	17325686	1.213	2.55E-02
Zc3H12D	zinc finger CCCH-type containing 12D	17231591	-1.006	3.82E-02
Zfp108/Zfp93	zinc finger protein 93	17474916	1.106	8.07E-03
Zfp119B	zinc finger protein 119b	17346069	1.241	2.63E-02
Zfp759	zinc finger protein 759	17288294	1.210	2.75E-02
Zfp760	zinc finger protein 760	17333966	1.135	2.33E-02
Znf358	zinc finger protein 358	17498602	1.321	1.06E-02
Znf366	zinc finger protein 366	17289432	1.535	2.36E-02
Znf705A	zinc finger protein 705A	17462729	-2.094	8.50E-03
Zyx	zyxin	17457942	-1.142	1.90E-02
1110038B12Rik	RIKEN cDNA 1110038B12 gene	17344114	-1.065	7.06E-03
1700099I09Rik	RIKEN cDNA 1700099I09 gene	17289539	1.322	9.44E-03
1700109K24Rik	RIKEN cDNA 1700109K24 gene	17312653	1.069	1.31E-02
2810029C07Rik	RIKEN cDNA 2810029C07 gene	17284111	1.747	8.74E-03
3222401L13Rik	RIKEN cDNA 3222401L13 gene	17349956	1.144	1.09E-02
4930429F24Rik	RIKEN cDNA 4930429F24 gene	17548765	1.293	3.02E-02
4930430E12Rik	RIKEN cDNA 4930430E12 gene	17423060	-1.551	1.78E-02
5033404E19Rik	NSA2 ribosome biogenesis homolog pseudogene	17220591	1.048	3.95E-02
5730408K05Rik	RIKEN cDNA 5730408K05 gene	17362521	-1.280	1.92E-02
9430037G07Rik	RIKEN cDNA 9430037G07 gene	17529590	-1.147	8.07E-03
9830107B12Rik/A5	RIKEN cDNA A530064D06 gene	17345775	-1.085	3.12E-02

Supplementary Table 7. Differentially expressed transporters in vascular fragments at preclinical, progression and remission phase of EAE

Gene Symbol	Entrez Gene Name	Fold change Preclinical	Fold change Progression	Fold change Progression (I)	Fold change Remission
**ABC (ATP-binding cassette) transporters**					
Abca8A	ATP-binding cassette, sub-family A (ABC1), member 8a		-1,25		
Abca9	ATP-binding cassette, sub-family A (ABC1), member 9		-1,62	1,79	
Abcb1B	ATP-binding cassette, sub-family B (MDR/TAP), member 1B		1,41		1,70
Abcc6	ATP-binding cassette, sub-family C (CFTR/MRP), member 6		-1,54		-1,08
Abcg1	ATP-binding cassette, sub-family G (WHITE), member 1		1,09		2,00
Tap1	transporter 1, ATP-binding cassette, sub-family B (MDR/TAP)		2,23	-1,79	1,24
Abca1	ATP-binding cassette, sub-family A (ABC1), member 1				1,15
Abcd2	ATP-binding cassette, sub-family D (ALD), member 2				1,44
Abcg2	ATP-binding cassette, sub-family G (WHITE), member 2			1,24	
**Solute carrier-mediated transport**					
	**Sodium bile salt cotransporters**				
Slc10A6	solute carrier family 10 (sodium/bile acid cotransporter), member 6		2,08	-1,50	
	**Proton coupled metal ion transporters**				
Slc11A1	solute carrier family 11 (proton-coupled divalent metal ion transporter), member 1		1,40		1,58
	**Proton oligopeptide cotransporters**				
Slc15A3	solute carrier family 15 (oligopeptide transporter), member 3		2,41	-2,78	1,32
	**Monocarboxylate transporter**				
Slc16A1	solute carrier family 16 (monocarboxylate transporter), member 1	-1,03	-1,21	1,35	
Slc16A10	solute carrier family 16 (aromatic amino acid transporter), member 10		1,18	-1,44	
Slc16A2	solute carrier family 16, member 2 (thyroid hormone transporter)		-1,50		
Slc16A3	solute carrier family 16 (monocarboxylate transporter), member 3		1,68	-1,59	
Slc16A4	solute carrier family 16, member 4		-1,93	1,75	
	**Folate/thiamine transporters**				
Slc19A3	solute carrier family 19 (thiamine transporter), member 3		-1,62	1,99	
	**High affinity glutamate and amino acids transporters**				
Slc1A2	solute carrier family 1 (glial high affinity glutamate transporter), member 2		-1,04		-1,43
Slc1A5	solute carrier family 1 (neutral amino acid transporter), member 5		1,03	-1,03	
	**Type III Na+-phosphate cotransporters**				
Slc20A1	solute carrier family 20 (phosphate transporter), member 1		1,32		
	**Organic cation/anion zwitterion transporters**				
Slc22A8	solute carrier family 22 (organic anion transporter), member 8		-2,00		
	**Mitochondrial carriers**				
Slc25A13	solute carrier family 25 (aspartate/glutamate carrier), member 13		1,13		
	solute carrier family 25 (pyrimidine nucleotide carrier), member 33				-1,05
	**Multifunctional anion exchangers**				
Slc26A10	solute carrier family 26, member 10		-2,51	1,96	-1,42
	**Facilitative GLUT transporters**				
Slc2A5	solute carrier family 2 (facilitated glucose/fructose transporter), member 5		-1,20	1,21	-1,11
Slc2A6	solute carrier family 2 (facilitated glucose transporter), member 6		1,75	-1,48	
	**Cu2+ transporters**				
Slc31A2	solute carrier family 31 (copper transporter), member 2		1,00		
	**Zn2+ efflux transporters**				
	solute carrier family 30, member 10				-1,17
	**Nucleoside-sugar transporters**				
Slc35F2	solute carrier family 35, member F2		-1,21	1,20	
	**Sugar-phosphate/ phosphate exchangers**				
Slc38A1	solute carrier family 38, member 1		1,25	-1,28	1,44
Slc38A11	solute carrier family 38, member 11		-1,02		
Slc38A3	solute carrier family 38, member 3		-1,56	1,44	
	solute carrier family 37 (glucose-6-phosphate transporter), member 2				1,40
	**Metal ion transporters**				
Slc39A12	solute carrier family 39 (zinc transporter), member 12		-1,00	1,07	
Slc39A14	solute carrier family 39 (zinc transporter), member 14		1,37	-1,48	
Slc39A6	solute carrier family 39 (zinc transporter), member 6		1,01		
	**Basolateral Fe2+ transporters**				
Slc40A1	solute carrier family 40 (iron-regulated transporter), member 1		-2,62	2,00	-1,35
	**L-like amino acid transporters**				
Slc43A3	solute carrier family 43, member 3		1,32	-1,41	
	**Bicarbonate transporters**				
Slc4A7	solute carrier family 4, sodium bicarbonate cotransporter, member 7		1,01		
	**Sodium and chloride dependent neurtransmitter transporters**				
Slc6A11	solute carrier family 6 (neurotransmitter transporter), member 11		-1,04		-1,35
	solute carrier family 6 (proline IMINO transporter), member 20	1,20			
	**Cationic amino acid transporter**				
Slc7A1	solute carrier family 7 (cationic amino acid transporter, y+ system), member 1		-1,20	1,01	
Slc7A11	solute carrier family 7 (anionic amino acid transporter light chain), member 11		2,45	-2,94	
Slc7A2	solute carrier family 7 (cationic amino acid transporter, y+ system), member 2		1,95	-2,05	
Slc7A5	solute carrier family 7 (amino acid transporter light chain, L system), member 5		-1,34	1,21	
Slc7A6	solute carrier family 7 (amino acid transporter light chain, y+L system), member 6		1,14		
Slc7A8	solute carrier family 7 (amino acid transporter light chain, L system), member 8		1,39	-1,18	
	solute carrier family 7 (amino acid transporter light chain, y+L system)				1,11
	**Na+/ H+ exchanger**				
Slc9A3R2	solute carrier family 9, subfamily A (NHE3, cation proton antiporter 3)		-1,02	1,23	
Slc9A7	solute carrier family 9, subfamily A (NHE7, cation proton antiporter 7),				1,08
Slc9A9	solute carrier family 9, subfamily A (NHE9, cation proton antiporter 9)				1,36
**ATPase Transporters**					
Atp10A	ATPase, class V, type 10A		-1,05		
Atp1A3	ATPase, Na+/K+ transporting, alpha 3 polypeptide		2,41	2,24	2,93
Atp2A3	ATPase, Ca++ transporting, ubiquitous		-1,09		
Atp6Ap2	ATPase, H+ transporting, lysosomal accessory protein 2		1,02		
Atp6V0C	ATPase, H+ transporting, lysosomal 16kDa, V0 subunit c		1,65	1,71	1,25
Atp6V0D2	ATPase, H+ transporting, lysosomal 38kDa, V0 subunit d2		1,87		2,71
Atp6V1B2	ATPase, H+ transporting, lysosomal 56/58kDa, V1 subunit B2		1,22		1,25
Atp6V1C1	ATPase, H+ transporting, lysosomal 42kDa, V1 subunit C1				1,23
Atp8B1	ATPase, aminophospholipid transporter, class I, type 8B, member 1		1,72		1,04
Atp8B4	ATPase, class I, type 8B, member 4		2,89	2,90	1,45
Atp1B2	ATPase, Na+/K+ transporting, beta 2 polypeptide				-1,19
Atp7A	ATPase, Cu++ transporting, alpha polypeptide		1,11		
**Receptor-Mediated Transport**					
Tfrc	transferrin receptor		-2,07	1,41	
Lepr	leptin receptor		-1,03	1,29	
Igf1R	insulin-like growth factor 1 receptor		-1,14	1,56	
**Major Facilitators**					
Mfsd2A	major facilitator superfamily domain containing 2A		-1,55	MFSD2A	

Supplementary Table 8. Comparison of the overlap between the BBB transcriptomes during EAE and the 'core BBB dysfunction module' gene list from Munji et al.

Colored boxes represent shared genes significantly regulated
(P value <0.05; log 2-fold change >1) in the EAE BBB transcriptomes.
* Log 2-fold change <1

Gene Symbol	Progression (PBS)	Remission	Progression (Imatinib)
Adamts4			
Adamts8			
Anln			
Atp8b1			
Bmp1			
C330027C09Rik	*		*
Ccl2			
Ccna2			
Cd14			
Cenpe			
Ch25h			
Cks1b			
Col3a1	*		
Cxcl10			
D17H6S56E-5			
Darc			
Dcn			
Emilin1			
Fbln1	*		
Gm6169			
Hmmr			
Igfbp4			
Igfbp5			
Itgb3			
Kit			
Lamb1			
Lbp			
Lgals1			
Lox	*		
Lrg1			
Mcm5			
Mki67			
Myc			
Pdlim1			
Plekho1			
Plekho2			
Postn			
Rrm2			
S100a6			
Scgb3a1			
Sele			
Selp			
Serping1			
Spp1			
Thbs1			
Thbs2			
Timp1			
Tmem173			
Tnc			
Top2a			
Tpx2			
Trp53i11			
Tubb6			
Upp1			

Supplementary Table 9. Comparison of overlap between the EAE BBB transcriptomes and 'BBB dysfunction module' from Munji et al. comprising common genes for at least 3 of 4 CNS disease models

The list excludes genes included in the 'core BBB dysfunction module'
Colored boxes represent shared genes significantly regulated
(P value <0.05; log 2-fold change >1) in the EAE BBB transcriptomes.
* Log 2-fold change <1

Gene Symbol	Progression (PBS)	Remission	Progression (Imatinib)
Acot7			
Adam12	*		
Adam19			*
Aebp1			
Aldh18a1	*		*
Aldh1a2	*		
Anxa1			
Apln			*
Aplnr			
Aprt			
Aspm			
B3gnt3			
Bicc1	*		
Bub1b			
Casp4			
Ccnb2			
Cd44			
Cdk1			
Cenpf			
Chst1			
Ckap2			
Cmtm3			
Col12a1	*		
Col1a1			
Col1a2	*		
Col5a1	*		
Col5a2	*		
Cyp1b1			
Dkk3	*		
Dpysl3			
Fblim1			
Fbn1			
Fkbp10			
Flnc	*		
Frrs1			
Fscn1			
Gjb2			
Ifitm1			
Igsf10			
Islr			
Kif11			
Kif15			
Kif20b			
Lcp2			
Lgmn			*
Loxl2			
Loxl3			
Lum			
Marcksl1			
Meox1	*		
Mmp14			
Ndc80			
Nfil3			
Nt5dc2			
Parp3			
Pgf			
Prc1			
Ptgfrn			
Ptgs2			*
Pvrl2			
Pxdn			
Robo1			
Runx1			
Serpine1			
Slc16a6			
Slc43a3			
Slc7a11			
Slc7a7			
Slfn9			
Sulf1			
Sulf2			
Synpo			
Tacc3			
Thy1			
Tlr2			
Tmem176a			
Tnfrsf23			
Tnfsf8			
Ube2c			
Vcan			
Vwf			
Zfp521	*		
